# Dibenzyl isophthalates as versatile hosts in room temperature phosphorescence host–guest systems†

**DOI:** 10.1039/d4sc07768g

**Published:** 2025-01-07

**Authors:** Martin Molkenthin, Emanuel Hupf, Boris J. Nachtsheim

**Affiliations:** a University of Bremen, Institute for Organic and Analytical Chemistry 28359 Bremen Germany nachtsheim@uni-bremen.de; b University of Bremen, Institute of Inorganic Chemistry and Crystallography 28359 Bremen Germany hupf@uni-bremen.de

## Abstract

We report a series of dibenzyl isophthalates (DBIs) as novel hosts for room-temperature phosphorescence (RTP) host–guest systems, achieving RTP quantum yields (QY) of up to 77% or lifetimes of up to 21.0 s with the guest coronene-*d*_12_. Furthermore, a 4,4′-Br substituted DBI was used to form host–guest RTP systems with 15 different aromatic guest molecules, to tune the phosphorescence emission color from blue to red and to demonstrate the versatility of the host. Mechanistic insights were gained through a host–guest–matrix system which shows RTP by trace combinations of a 4,4′-Br DBI host (0.10 wt%) and a pyrene-*d*_10_ guest (0.01 wt%) in an otherwise non-RTP-emissive aromatic matrix. This work establishes DBIs as readily available and versatile, tunable hosts for RTP host–guest systems, posing an alternative to polymeric hosts.

## Abstract 2

Simple yet powerful: dibenzyl isophthalates are easy to modify hosts for room-temperature phosphorescence. These systems achieve record-setting quantum yields and lifetimes while offering unprecedented color tunability across the visible spectrum.

## Graphical abstract

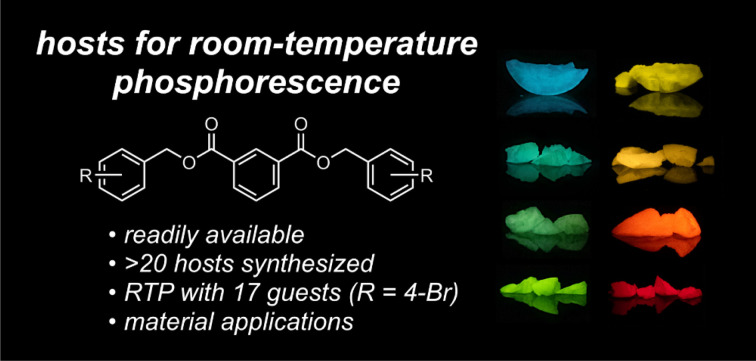

## Introduction

The design of efficient phosphorescent emitters frequently involves the incorporation of heavy transition metals, such as d^8^ or d^10^ metals.^1,2^ The development of purely organic materials showing room temperature phosphorescence (RTP) is more challenging, but significant progress has been achieved in recent years including phosphorescence with ultra-long lifetimes in the range of seconds.^3–5^ Due to their unique afterglow properties, RTP materials found applications in encryption, anti-counterfeiting and high-resolution bio-imaging, amongst others.^6,7^ The development of new, efficient RTP materials still remains challenging and repeatedly consists of trial-and-error approaches.^8,9^

Key challenges in realizing organic RTP are the efficient population and stabilization of triplet excited states for high quantum yields and long phosphorescence lifetimes.^10–12^ Synthetic strategies to achieve RTP compounds and materials are the use of polymers,^13–16^ supramolecular assemblies,^17^ molecular aggregates,^18–20^ carbon dots^21–23^ or through crystallization.^24,25^

Host–guest RTP systems are promising candidates for realizing organic RTP, generally consisting of a solid compound that acts as a matrix (host) and a phosphorescent dopant (guest) in high dilution.^26–29^ However, the variety of studied hosts and/or guests in these literature examples is generally small and the general design of most of these systems leave little room for straightforward variations.^30–39^ For this reason, there is a need for host compounds being highly modifiable with the potential of enabling RTP for structurally divers guests.

In an increasing number of cases, the RTP emission of a compound was found to arise from incorporated trace impurities, making them in reality host–guest RTP systems.^40–44^ Identifying these impurities, or even just proving their existence, can be very challenging as RTP may arise even already at ppb levels of contamination.^45^ Therefore, host–guest systems steadily gain in importance among purely-organic RTP materials. Among purely organic RTP materials, isophthalic acid is a compound with outstanding RTP properties as a crystalline solid.^46,47^ It is also capable of serving as a host compound for host–guest RTP.^48^ Polymeric isophthalic acid esters have also been shown to be capable of RTP.^49^

In this study, we show that a series of simple, substituted dibenzyl isophthalates (DBIs) serve as versatile hosts to enable color-tunable RTP across the spectrum from blue to red. A mechanistic analysis further provides insights into the RTP process, supporting the potential of DBIs for future development as minimalistic RTP host–guest materials.

## Results and discussion

### Initial discovery

Intrigued by the known photophysical properties of isophthalic acid and its derivatives, we investigated isophthalic acid esters as possible hosts for RTP host–guest systems and found DBIs as promising host compounds. DBIs combine several advantages, such as the synthesis in high purity at low costs and chemical robustness. DBIs offer a plethora of options for different substituent patterns and post-functionalizations, which allow for the efficient synthesis and screening of many different host compounds. Moreover, the benzylic design of these hosts was chosen to achieve effective molecular packing in the solid state to suppress nonradiative decay rates of the excited triplet states of guests and to restrict oxygen diffusion.^50^

**Scheme 1:**
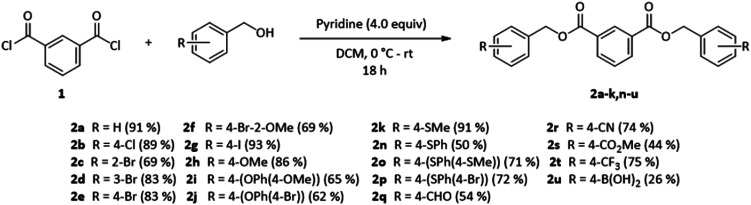
Scope of the dibenzyl isophthalates synthesized in this work *via* nucleophilic substitution of isophthaloyl dichloride (1) with substituted benzyl alcohols. The esters 2l (R = 4-S(O)Me) and 2m (R = 4-SO_2_Me) were synthesized by oxidation of 2k. For detailed data, see ESI Chapter 5.2.†

We synthesized a diverse set of 21 DBIs (2a–2u) in yields of 50% to 91% to gauge the effect of functional groups at the benzylic ester moiety on the RTP properties of the studied host–guest systems. DBIs could be synthesized using isophthaloyl dichloride (1) in a simple, pyridine-catalyzed one-step substitution reaction (Scheme 1) and free of RTP-causing trace impurities (see ESI Chapter 5.2† for details).

### DBI host–guest systems with coronene

As a benchmark, we doped 0.1 wt% of coronene guest (3) into the observed hosts (Table 1).^37^ This wt% induced the most intense RTP-emission from all tested percentages (see ESI Chapter 3.4† for details). Melt-cast samples of host–guest systems were formed by addition of a solution of the guest to the host, followed by solvent evaporation and melting of the mixture under vacuum. After cooling to rt, solid host–guest systems were obtained (see ESI Chapter 1.4† for details). We analyzed the thermal stability of selected DBIs which showed degradation only above 200 °C, demonstrating the thermal stability (see ESI Chapter 8.1† for details). All samples were photoactivated by irradiation with UV-lamp before measurement (see ESI Chapter 1.2† for details). The time needed for the photoactivation correlated with the RTP lifetime. The process is thought to either remove residual oxygen from the solids or increase intermolecular interactions, which both limit nonradiative transitions.^51,52^

**Table 1:** Phosphorescence lifetimes and quantum yields of DBIs with 0.1 wt% coronene (3); melt-cast samples under ambient conditions.^a^ The three highest phosphorescence lifetimes and quantum yields have been highlighted. For more details, see ESI Chapter 3.2

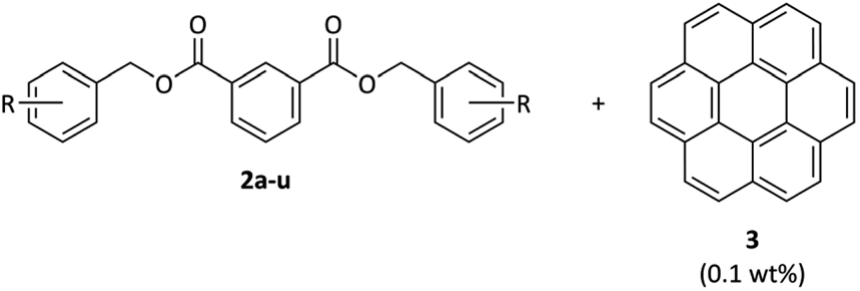					
No.	R =	*τ*_P_ b ^,^ c ^,^ d	*φ*_total_ [%]	*φ*_Fl_ [%]	*φ*_Phos_ [%]
2a	All H	5.11 s	13.2	12.8	0.4
2b	4-Cl	6.20 s	17.8	11.6	6.2
2c	2-Br	1.58 s	19.4	5.8	13.6
2d	3-Br	1.77 s	10.2	7.2	3.0
2e	4-Br	3.61 s	16.5	6.0	10.5
2f	4-Br-2-OMe	1.29 s	31.0	4.5	**26.5**
2g	4-I	65 ms	42.5	<0.1	**42.5**
2h	4-OMe	5.70 s	24.6	22.2	2.4
2i	4-(OPh(4-OMe))	3.08 s	11.0	9.9	1.1
2j	4-(OPh(4-Br))	0.94 s	18.9	9.2	9.7
2k	4-SMe	5.13 s	13.0	8.8	4.2
2l	4-S(O)Me	5.36 s	33.8	31.9	1.9
2m	4-SO_2_Me	4.91 s	8.4	6.9	1.5
2n	4-SPh	**6.46 s**	23.3	19.6	3.7
2o	4-(SPh(4-SMe))	2.12 s	21.7	20.7	1.0
2p	4-(SPh(4-Br))	1.95 s	23.6	3.8	**19.8**
2q	4-CHO	4.57 s	3.6	2.6	1.0
2r	4-CN	**6.39 s**	16.0	13.2	2.8
2s	4-CO_2_Me	5.17 s	23.8	19.0	4.8
2t	4-CF_3_	3.27 s	42.3	42.3	<0.1
2u	4-B(OH)_2_	**6.32 s**	12.5	11.5	1.0

a Excitation at 350 or 345 nm.

b Average lifetimes from multi-exponential decays (mono-exponential exceptions: 2b, 2n, 2r).

c Emission measurement at 570 nm for RTP lifetime determination.

d See ESI Chapter 1.2 for details on the determination of the quantum yields and lifetimes.

**Fig. 1:**
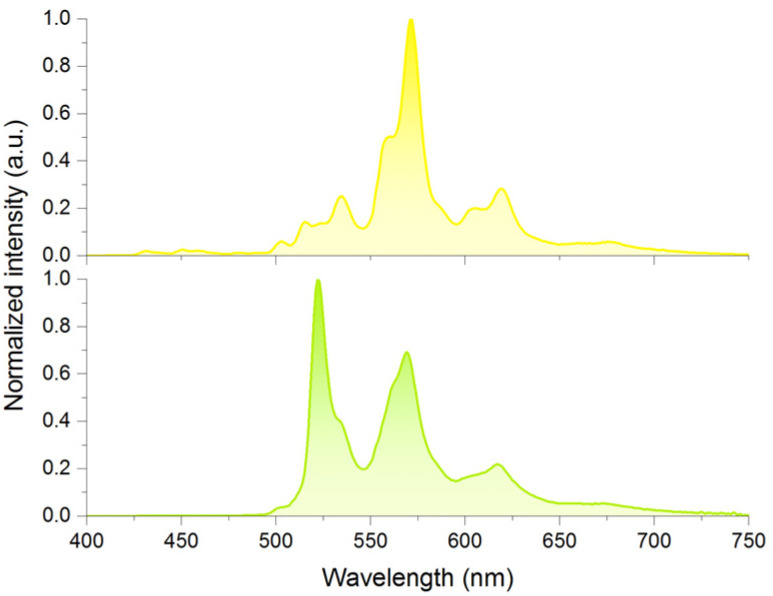
Phosphorescence spectra of the (top) coronene (3)@4-CN DBI (2r) and (bottom) coronene (3)@4-Br-2-OMe DBI (2f) host–guest systems. Excitation at 350 nm.

To our delight, all coronene (3) doped DBIs showed RTP. To the best of our knowledge, this presents the first study of a host being functionalized with a multitude of functional groups (>20) and still showing RTP in almost all cases. Phosphorescence spectra of these composites with 0.1 wt% coronene (3) showed emission from 500–700 nm, with the main emission features being slightly hypsochromically shifted for bromine and iodine substituted DBIs (see Fig. 1 for representative examples). The T_1_–S_0_ transition that leads to RTP emission at ∼523 nm is thought to be enabled by out-of-plane spin–orbit interactions of 3.^53^ Therefore, the transition might be more susceptible to the external heavy-atom effect of a host compound than the transition related to the emission at 569 nm, explaining the change in intensity ratios. The same effect was found with another iodine-substituted host.^54^

Aside from the phosphorescence, a small amount of delayed fluorescence was also present in the RTP-spectra from *ca.* 430–500 nm since its lifetime is similar to that of the phosphorescence for 3.^55^ However, in comparison with the RTP, its intensity was low, and lowered further with increasing RTP quantum yields of the host–guest systems.

As expected, the presence of a bromine or iodine substituent significantly increased RTP quantum yields due to the heavy-atom effect.^56,57^ This trend can be rationalized by comparing RTP quantum yields of the unsubstituted parent dibenzyl isophthalate 2a (*φ*_Phos_ = 0.4%) to the *para*-substituted halide analogues 4-Cl (2b, 6.2%) < 4-Br (2e, 10.5%) < 4-I (2g, 42.5%). Phosphorescent lifetimes decreased from 2b to 2g (Table 1).

We also measured the photostability of 2b, 2e and 2g in their host–guests systems with 3, and that of 2e in solution without guest (see ESI Chapter 8.2† for details). 2e showed only minor degradation after 7.5 h of continuous irradiation at 345 nm under air, while host–guest systems showed persistent lowering of RTP-emission over several hours. The 4-Cl DBI (2b) host showed the slowest decline with *ca.* 75% RTP intensity remaining after 24 h, while 4-Br DBI (2e) had approximately 50% intensity remaining. 4-I DBI (2g) also had *ca.* 50% remaining RTP-intensity, but after 14 h of irradiation. Notably, the photostability is also highly guest dependent and only rarely investigated in the literature for purely organic host–guest systems.

The influence of the substitution position on the phosphorescence was probed by synthesizing the *ortho*- and *meta*-analogs of the 4-Br substituted DBI (2e). The 2-Br DBI (2c, *φ*_Phos_ = 13.6%) showed a comparable RTP quantum yield to 2e, while that of 3-Br DBI (2d, *φ*_Phos_ = 3.0%) was significantly lower. Interestingly, inserting a methoxy group as in 4-Br-2-OMe DBI (2f, *φ*_Phos_ = 26.5%), the quantum yield increased significantly compared to 2e. These findings illustrate that a small variation in the substitution pattern of the benzyl esters can lead to strong changes in *φ*_Phos_ and highlight the potential of DBIs as tunable hosts for organic RTP materials.

Incorporation of a thioether slightly raised *φ*_Phos_ of 4-SMe DBI (2k, *φ*_Phos_ = 4.2%) in comparison to the oxygen analogue 4-OMe DBI (2h, *φ*_Phos_ = 2.4%). An extension of the π-system to 4-(OPh(4-OMe)) DBI (2i) and 4-(SPh(4-SMe)) DBI (2o) lowered both RTP quantum yields and lifetimes. On the other hand, substitution of the methyl ether or thioether with a *p*-bromophenol- or *p*-thiophenol-(thio)ether strongly increased RTP quantum yields up to *φ*_Phos_ = 19.8% (2p). Interestingly, the 4-(SPh(4-SMe)) DBI (2o) showed the highest ratio of delayed fluorescence to phosphorescence in its RTP spectrum, which might be due to the presence of sulfur atoms facilitating ISC, coupled with a low RTP quantum yield (*φ*_Phos_ = 1.0%). Oxidation of the sulfur atom of 2k led to comparably low RTP quantum yields for 4-S(O)Me DBI (2l, *φ*_Phos_ = 1.9%) and 4-SO_2_Me DBI (2m, *φ*_Phos_ = 1.5%), although lifetimes remained very similar between *τ* = 4.91 s and *τ* = 5.36 s. Oxidation of the sulfur atom led to lower quantum yields potentially through less effective host–guest complexation by the induced steric demand of the oxygen atoms. The effect might be balanced by stronger, polar intermolecular interactions between the host molecules, leading to more stabilized triplet states and the observed similar lifetimes. Similarly, 4-CHO DBI (2q) and 4-CF_3_ DBI (2t) showed comparably low RTP quantum yields (*φ*_Phos_ = 1.0% and <0.1%) likely through steric effects, while the planar cyano group in 4-CN DBI (2r) showed a higher RTP quantum yield of *φ*_Phos_ = 2.8% and the second longest RTP lifetime with *τ* = 6.39 s. The RTP lifetime of this host–guest system was re-analyzed after the sample was stored for two years (see ESI Fig. S43†). It remained practically unchanged at *τ* = 6.44 s, demonstrating long-term stability of the DBI-based host–guest system. Furthermore, the RTP lifetime under argon atmosphere barely increased to *τ* = 6.48 s, demonstrating efficient restriction of oxygen diffusion in the solid. In 4-CO_2_Me DBI (2s), the bulkiness of the group likely induces a more efficient packing in the crystal with better host–guest complexation, leading to a comparably high RTP quantum yield of *φ*_Phos_ = 4.8%. In 4-B(OH)_2_ DBI (2u), a very high RTP lifetime of *τ* = 6.32 s results from the formation of boroxines, creating a glassy matrix,^58^ as evidenced by loss of water from thermogravimetric analysis (TGA) (see ESI Fig. S191†).

When the methyl thioether of 2k was changed to a phenyl thioether (4-SPh DBI, 2n) *φ*_Phos_ decreased slightly to 3.7% compared to 2k. However, the RTP lifetime increased to the highest of all investigated DBIs with *τ* = 6.46 s. To the best of our knowledge, this is the longest measured RTP lifetime of coronene (3) in a purely organic host–guest RTP system, longer than 3 in a poly(methyl methacrylate) (PMMA) matrix^59^ or in β-estradiol.^60^ Both have reported RTP lifetimes of *τ*_Phos_ = 6.0 s (see also ESI Table S3† for a comparison).

In conclusion, while there was no superior host compound for 3 both in terms of RTP lifetime and quantum yield due to their inverse relationship, several compounds showed exceptional performance for one of these categories, or a balanced mixture of the two. Selected host compounds were then further analyzed by employing the guest coronene-*d*_12_.

### DBI host–guest systems with coronene-*d*_12_

**Fig. 2:**
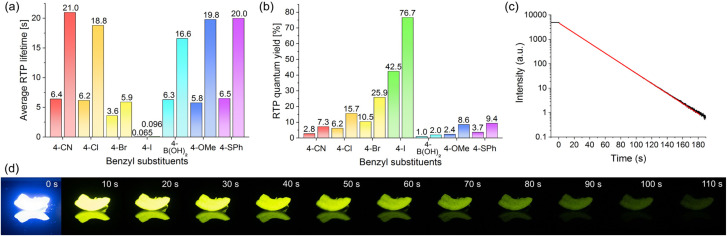
(a) Comparison of the lifetimes of DBI host–guest systems with coronene (3) as a guest (0.1 wt%, left columns) and with coronene-*d*_12_ as a guest (0.1 wt%, right columns). (b) Comparison of the RTP quantum yields of DBI host–guest systems with coronene (3) as a guest (0.1 wt%, left columns) and with coronene-*d*_12_ as a guest (0.1 wt%, right columns). (c) Lifetime decay curve (black line) and exponential tail fit (red line) of the coronene-*d*_12_@4-CN DBI (2r) host–guest system (emission at 570 nm). Excitation at 350 nm for (a)–(c). (d) Photographs of the luminescence of the coronene-*d*_12_@4-CN DBI (2r) host–guest system under irradiation with a 365 nm flashlight and after its removal. For detailed data, see ESI Chapter 3.3.†

The observed long lifetime of coronene@4-SPh DBI (2n), prompted us to investigated the influence of deuterated coronene (3) on the RTP lifetimes and quantum yields for selected hosts (Fig. 2), as deuteration of guests can drastically improve RTP lifetimes and quantum yields.^39,61,62^ Host–guest systems with coronene-*d*_12_ have been reported to exhibit RTP lifetimes of close to or over 20 s, which are among the longest measured lifetimes for organic host–guest systems.^55,59,63^ For comparison, we chose four DBIs with the longest lifetimes from Table 1, as well as 4-Br DBI (2e) for its balanced RTP lifetime and quantum yield. 4-I DBI (2g) was selected due to its high RTP quantum yield.

Coronene-*d*_12_ doped (0.1 wt%) 4-Br DBI (2e) and 4-I DBI (2g), showed an increase in RTP lifetime compared to the non-deuterated host–guest systems of up to 60%. For the other DBIs, RTP lifetime was increased approximately 2.5 fold in each case (Fig. 2a). The coronene-*d*_12_@4-CN DBI (2r) host–guest system showed the longest RTP lifetime of *τ* = 21.0 s (Fig. 2c and d). This lifetime is longer than the previous record lifetime of a purely-organic, non-polymeric host–guest RTP system with β-estradiol as the host compound (*τ* = 17.0 s).^63^ Moreover, the lifetime of the coronene-*d*_12_@4-CN DBI (2r) system was only slightly shorter than that of coronene-*d*_12_ in PMMA (*τ* = 23 s)^59^ and of coronene-*d*_12_ in the metal–organic framework ZIF-8 (*τ* = 22.4 s),^55^ which are the host–guest systems with the currently longest known RTP lifetimes of coronene-*d*_12_ (see also ESI Table S3† for a comparison).

RTP quantum yields also increased through deuteration of 3. 4-B(OH)_2_ DBI (2u) showed the smallest absolute increase in *φ*_Phos_, from 1.0% to 2.0%. 4-CN DBI (2r), 4-Cl DBI (2b), 4-Br DBI (2e) and 4-SPh DBI (2n) all showed an increase in RTP quantum yield by a factor of 2.5. For 4-OMe DBI (2h), we observed an increase with a factor of 3.6 (Fig. 2b). 4-I DBI (2g) showed the smallest relative increase in RTP quantum yield, but showed an extremely high *φ*_Phos_ of 76.7%.

### Host–guest RTP from 4-Br DBI with different guests

**Fig. 3:**
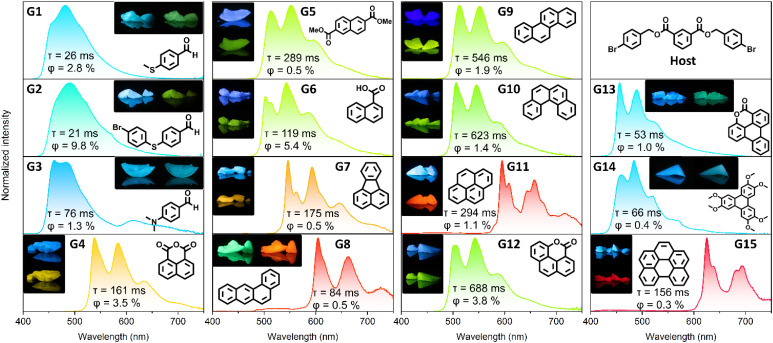
RTP emission spectra of guests (0.1 wt%) in 4-Br DBI (2e). The RTP quantum yield and average lifetimes are annotated. The photographs show the steady-state luminescence (left or top pictures) and the phosphorescence after removal of the light source (right or bottom pictures). Samples were excited with a 365 nm flashlight for photographs. For detailed measurement data (excitation wavelengths, quantum yields and lifetimes) see ESI Chapter 4.1.†

Motivated by our findings, we targeted color tunable host–guest systems. 2e was employed as a host, due to the balanced RTP quantum yield and lifetime values of the coronene (3)@4-Br DBI (2e) system. To achieve emission colors of the full visible spectrum, we focused on the readily available, structurally-diverse aromatic compounds G1–G15 (Fig. 3), and prepared host–guest systems in analogy to 3 to show the versatility of the host. The RTP excitation spectra of these systems all matched those of the absorption spectra of the respective guests in solution, indicating that no impurities in the guests were causing RTP in the host–guest systems.

Upon synthesizing 4-SMe DBI (2k), we could identify 4-(methylthio)-benzaldehyde (G1) as an RTP causing trace impurity (G1). Now using G1 as guest in 4-Br DBI (2e), it also caused blue-green RTP. Substituting the thiomethyl residue for a 4-bromophenylthioether caused a 3.5-fold increase in RTP QY from 2.8% for G1 to 9.8% for G2. Similar to G1, RTP was also obtained by doping 4-(dimethylamino)benzaldehyde (G3) into 2e. The RTP of G3 in a host–guest system has been described before,^33^ but no maximum at 613 nm was reported in the RTP emission spectrum. This maximum might be attributed to the long wave emission band (LE), arising from a twisted intramolecular charge-transfer state (TICT) of G3.^64,65^

Many polycyclic aromatic hydrocarbons showed RTP with 2e, highlighting the versatility of this simple, non-polymeric host compound. Compounds such as naphthalene derivatives G4–G6 and the structurally related fluoranthene (G7),^66–71^ benzophenanthrene isomers G8–G11,^72–75^ and benzo-[*ghi*]perylene (G15)^76–78^ allowed us to prepare host–guest RTP systems with colors ranging from yellow to deep red. Some of these guests have been described before in host–guest RTP systems (see ESI Table S6† for a comparison). For G10, to our best knowledge, there has not been a report of RTP yet except for a (currently) non-peer-reviewed study.^79^

Pyrene (G11) and its fully deuterated counterpart are well-studied compounds for host–guest RTP systems, but it is difficult to achieve notable quantum yields.^63,67,80–83^ For comparison, we also investigated host–guest systems with pyrene-*d*_10_ in 2e, 2b and 2g (see ESI Chapter 4.3 and ESI Table S7† for details), yielding RTP QY of up to 4.1%.

The only benzophenanthrene isomer that did not show RTP with 2e was triphenylene, which was expected to show blue RTP.^84^ Instead, we could obtain blue RTP by employing the literature-known guest G14.^85^ Moreover, neither unsubstituted naphthalene nor phenanthrene showed RTP as guests.

Since the introduction of acids, esters and anhydrides seemed to enable RTP for naphthalene derivatives G4–G6 with 2e, we applied this structural principle to phenanthrene and triphenylene, and synthesized G12 and G13 (see ESI† chapter 5.3 for details). A similar functionalization was recently used by Yu and coworkers to boost the RTP properties of coronene (3) as a guest by substituting it with esters.^86^ Incorporating G12 and G13 into 2e showed green and blue RTP respectively, with RTP spectrums close to their unsubstituted counterparts.^84^ Unfortunately, G13 proved to be light-sensitive in the host–guest system and degraded quickly upon irradiation, which might have contributed to the low RTP quantum yield.

In summary, the 4-Br DBI (2e) host showed great capability of enabling RTP for a variety of structurally different guests. Therefore, the compound may also serve as a capable host compound for testing new or unknown guests, as an alternative to polymeric hosts which might fail to yield significant RTP intensities in some cases (see ESI Table S6† for a comparison of 2e with hosts described in the literature for some of the guests shown here).

### Material properties of DBIs

**Fig. 4:**
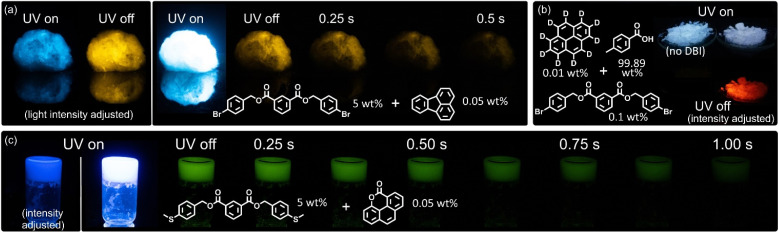
(a) Cotton wool after soaking it with a DCM solution of 4-Br DBI (2e) (5 wt%) and fluoranthene (G7) (0.05 wt%) and drying in air. (b) Two melt-cast samples from *p*-toluic acid (99.89 wt%), 4-Br DBI (2e) (0.1 wt%) and pyrene-*d*_10_ (0.01 wt%). The left sample was prepared without the DBI as a control. (c) Self-standing gel or gel-like precipitate from 4-SMe DBI (2k) (5 wt%) and G12 (0.05 wt%) from a DCM solution by addition of MeOH (wt% refers to the total wt% after MeOH addition; 1 : 1 mixture of MeOH and DCM). Photographs of samples under UV irradiation (365 nm flashlight) and after removing the light source.

Our host–guest systems are not limited to a crystalline environment. When cotton wool is soaked with a dichloromethane (DCM) solution of small amounts of 4-Br DBI (2e) and fluoranthene (G7), the resulting wool displayed RTP after drying under air (Fig. 4a). Despite the low RTP quantum yield of the G7@4-Br DBI (2e) host–guest system, the afterglow was easily visible by eye in a dark room.

Phosphorescence is also induced if the DBI host is present only in trace amounts and when both, DBI and guest, are embedded in a crystalline matrix together. When we prepared a melt-cast sample from *p*-toluic acid (99.99 wt%) and pyrene-*d*_10_ (0.01 wt%), no RTP was observed after UV-irradiation. However, when 4-Br DBI (2e) was added in trace amounts (0.1 wt%), red RTP could be observed (Fig. 4b, see also ESI Chapter 4.3† for details).

Finally, we found that selected DBIs showed gelation-type behavior when solutions in organic solvents were diluted with protic solvents. As a representative example, we diluted a DCM solution of G12@4-SMe DBI (2k) with methanol and found near instant gelation-like solidification of the solution (Fig. 4c). The solidified mixture was self-standing upon inversion of the vial, indicative of an organogel. It further showed the green RTP of G12, proving effective restriction of vibrational interference and oxygen diffusion of the host matrix. This result suggests that DBIs could be utilized as phosphorescent low-molecular weight gelators in the future. A similar result was recently reported by Xiang and co-workers, who prepared carbazole-derived amides of different (aromatic) acids as phosphorescent organogelators, including one of isophthalic acid.^87^

### Influence of the matrix on DBI host–guest RTP properties

We further investigated the role of the matrix for the RTP of DBI-based host–guest systems by embedding the guest pyrene-*d*_10_ (0.1 wt%) in four different matrices, and added a DBI (1 wt%) to gauge its influence on the resulting luminescence properties (Table 2). 4-H DBI (2a) host showed low fluorescence QY (*φ*_FL_ = 0.041) and miniscule RTP QY (*φ*_Phos_ = ∼0.0006), which could both be increased 2.5-fold by additional doping with 1 wt% 4-Br DBI (2e). RTP lifetime simultaneously decreased by 10% due to increased ISC by the heavy atoms of 2e. The increase of fluorescence QY by doping with 2e might be explained by the formation of non-emitting exciplexes of pyrene-*d*_10_ with 2a, but to a lesser extent with 2e. Similarly, host 2a previously showed a broad fluorescence maximum with coronene (3) as guest, indicative of exciplex fluorescence (see ESI Fig. S7†).

**Table 2:** Phosphorescence lifetimes and quantum yields of different DBI-matrix systems with 0.1 wt% pyrene-*d*_10_ as a guest; melt-cast samples under ambient conditions^a^

Host/matrix (≥98.9 wt%)	Co-host (1 wt%)	*τ*_P_ b ^,^ c ^,^ d	*φ*_Fl_ [%]	*φ*_Phos_ [%]
H-DBI (2a)	—	1.96 s	4.1	∼0.06
H-DBI (2a)	4-Br DBI (2e)	1.74 s	10.4	∼0.17
4-CF_3_ DBI (2t)	—	—	8.9	—
4-CF_3_ DBI (2t)	4-Br DBI (2e)	—	7.1	—
4-Br DBI (2e)	—	1.26 s	19.2	2.7
4-Br DBI (2e)	4-CF_3_ DBI (2t)	1.52 s	9.8	1.2
*p*-Toluic acid	—	—	n.d.	—
*p*-Toluic acid	4-Br DBI (2e)	1.01 s	5.4	1.0

a Excitation at 345 nm.

b Average lifetimes from multi-exponential decays.

c Emission measurement at 595 nm for RTP lifetime determination.

d See ESI Chapter 1.2 for details on the determination of the quantum yields and lifetimes. n.d.: not determined/measured.

4-CF_3_ DBI (2t) neither showed measurable RTP with pyrene-*d*_10_, nor with additional 2e. Moreover, fluorescence QY decreased by 20% by doping with 2e. In the steady-state emission spectra (see ESI Fig. S129†), a considerable decrease in excimer fluorescence of pyrene at ∼470 nm is seen as compared to similar DBI-based host–guest systems with pyrene-*d*_10_ (see ESI Fig. S116, S122–126†). This de-aggregation of pyrene-*d*_10_ is further indicative of the formation of non- or weakly emissive host–guest exciplexes between the guest and the DBI. By doping 2t with additional 4-Br DBI (2e), ISC of the proposed 2t-pyrene-*d*_10_ exciplex is increased through (external) heavy atoms. However, the resulting triplet species is not emissive as well which leads to an overall net-decrease in fluorescence emission.

When 4-CF_3_ DBI (2t) is doped into the pyrene-*d*_10_@4-Br DBI (2e) host–guest system instead, the effect is two-fold. Luminescence quantum yields decrease by 50–60% through the proposed non-emissive exciplex formation between guest and 2t, while RTP lifetime is increased by 20%. The increase in lifetime can be rationalized by reduced ISC rates due to the lack of heavy-atoms in 2t. In contrast, 4-H DBI (2a) and 4-Br DBI (2e) both stabilize triplet excited states of guests as seen by the long RTP lifetimes of the guests, and do not actively restrict the formation of these states.

DBIs are further proven as effective matrices for stabilizing triplet excited states of guests by comparison with *p*-toluic acid as a matrix. Without a DBI host, no RTP is observed from the guest (Fig. 4c) in this matrix. With 1% of 4-Br DBI (2e), RTP is enabled, but the lifetime is 20% shorter as compared to pure 2e as a host. Quantum yields are also reduced by a factor of 3.5 for fluorescence and 2.5 for RTP. The experiments show that DBIs are potent matrices for stabilizing triplet states of guests, and may also be used in small quantities in other matrices to modulate the RTP properties of guests.

### TD-DFT computations and mechanistic considerations

From Fig. 4b, it is apparent that the RTP of the pyrene-*d*_10_@4-Br DBI (2e) system arises more likely from the interaction of a single pair of molecules, rather than aggregation in form of clusters or a certain crystal phase. For this reason, we investigated a single pair of host and guest for time-dependent density functional theory (TD-DFT) computations.

TD-DFT computations were conducted with 4-Br DBI (2e) as the host and coronene (3), pyrene (G11) and the lactones G12 and G13 as guests. In the optimized gas-phase geometries, the benzyl residues of 2e are tilted towards the planar guest molecules.

**Fig. 5:**
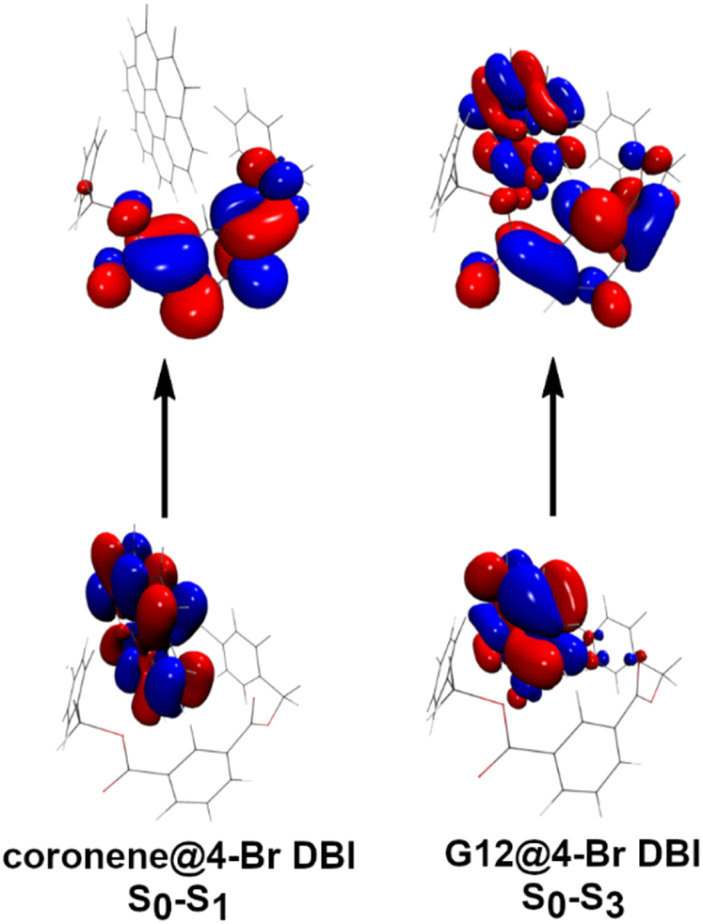
Natural transition orbitals (NTO) for TD-DFT computed S_0_→S_*n*_ transitions of coronene (3)@4-Br DBI (2e) (left) and G12@4-Br DBI (right).

A requirement for efficient intersystem crossing is the presence of excited singlet and triplet states being energetically within <0.1 eV, which was found in all four investigated systems (see ESI Table S8†). The excitation from the ground state guest@4-Br DBI (2e) to excited singlet states involves both the host and the guest (see ESI Fig. S183–186†). Inspection of the natural transition orbitals participating in the transitions revealed that the excitation is either of almost pure charge-transfer character as *e.g.* for coronene (3) (Fig. 5, left), or involves both the host and guest as for the lactones G12 and G13 (Fig. 5, right). This result is in agreement with findings by Yang *et al.*, who showed the importance charge-transfer between host and guest to realize RTP.^88^

**Fig. 6:**
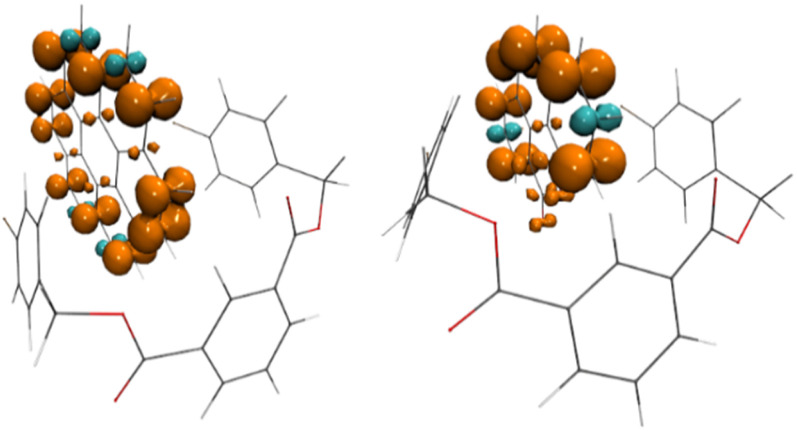
Spin density of T_1_-state of coronene (3)@4Br DBI (2e) (left) and G12@4-Br DBI (2e) (right) representation at +0.005/−0.005 e bohr^−3^ (orange/cyan).

The computed differences of the adiabatic energies (*E*_adia_ and *E*_0−0_) of the S_0_ and T_1_ are in reasonable agreement with the experimentally obtained phosphorescent energies (see ESI Table S9†), reproducing the observed color tunability of the systems. The spin density of the T_1_ state resides in all cases on the guest, suggesting that the observed phosphorescence stems from the guest (Fig. 6 and ESI Fig. S187†). This is further corroborated by the computed *E*_adia_ and *E*_0−0_ energies of the individual host and guests alone, leading to similar values for the guests, but significant larger energies for the host (see ESI Table S10†).

Pyrene (G11) is known to form exciplexes which can rapidly accelerate the ISC process.^89,90^ When we tested the influence of different doping amounts of pyrene-*d*_10_ on the fluorescence and RTP emission spectra of the pyrene-*d*_10_@4-Br DBI (2e) host–guest system, the excimer fluorescence of pyrene-*d*_10_ at 469 nm decreased with decreasing guest amounts (see ESI Chapter 4.2† for details). This indicates an origin of excimer emission solely from the pyrene dimer interactions in the solid state, rather than an exciplex between pyrene-*d*_10_ and 2e.^91^ This further suggests, along with our experiments and calculations, the formation of a non- or weakly-emissive exciplex between pyrene and DBI-hosts in the host–guest systems upon excitation. For isophthalic acid, its RTP is thought to be enabled by hyperfine coupling in a radical ion pair (RIP), as RIPs were shown to be involved in the mechanism of the RTP.^46^ Similarly, the RTP of benzoindole derivatives was recently shown to involve radical cations.^32^

In conclusion, we assume that the respective guest and DBI form an excited singlet state, which turns into a matrix-stabilized RIP. The singlet RIP then undergoes rapid ISC to a triplet RIP, which recombines to yield the guest in the triplet state and the DBI in the ground state.^92^ The radiative emission of the triplet guest is then observed as RTP. A similar mechanism was also suggested by Ma *et al.* very recently for the G10@benzophenone host–guest RTP system and for other PAH guests with the same host.^79^ We propose that the formation of RIPs for stabilizing excited states is finally responsible for the outstanding RTP properties that guests form with DBI hosts.

## Conclusions

We have shown that dibenzyl isophthalates (DBIs) are versatile, highly modifiable and easily accessible hosts for room temperature phosphorescence (RTP). Host–guest RTP systems with coronene (3) and coronene-*d*_12_ as guests showed RTP properties that rival or exceed comparable state-of-the-art host–guest RTP systems. Namely, the coronene-*d*_12_@4-I DBI (2g) system could achieve a RTP quantum yield of 76.7% while 4-CN DBI (2r) as a host let to a RTP lifetime of 21.0 s with the same guest. Furthermore, the versatility of 4-Br DBI (2e) as a host was shown by employing 15 different guests to construct host–guest RTP systems, emitting colors ranging from blue to red. The properties of DBIs, which could lead to the development of RTP material applications of DBIs, were outlined. A mechanism for the RTP of DBIs and guests was formulated which could help to further understand the phenomenon of host–guest RTP. In the future, we expect the development of more structurally diverse isophthalates as hosts for RTP applications. Furthermore, DBIs might serve as capable hosts for the research into new guest compounds.

## Data availability

For full experimental procedures, and spectroscopic and analytical data for all new compounds including copies of NMR spectra, see the ESI.† Notably, a full, concise summary of all photophysical data is presented in ESI Chapter 2.†

## Author contributions

M. M. and B. J. N. conceived the project. M. M. performed the syntheses, photophysical experiments and routine analytics. E. H. performed the DFT calculations. All authors designed the experiments and participated in writing and reviewing of the manuscript.

## Conflicts of interest

There are no conflicts of interest to declare.

## Supplementary Material

SC-OLF-D4SC07768G-s001
