# Supplementary material for: Dibenzyl isophthalates as versatile hosts in room temperature phosphorescence host–guest systems
Source: Chem Sci. 2025 Jan 7;16(6):2819–29. doi: 10.1039/d4sc07768g (PMC11726582; doi:10.1039/d4sc07768g)
Supplement: SC-016-D4SC07768G-s001 [file SC-016-D4SC07768G-s001.pdf]

Supplementary Information (SI) for Chemical Science.
This journal is © The Royal Society of Chemistry 2025

Dibenzyl Isophthalates as Versatile Hosts in Room Temperature Phosphorescence Host-Guest Systems

Supporting Information

Martin Molkenthin[a], Emanuel Hupf[b]*, and Boris J. Nachtsheim[a]*

[a] University of Bremen, Institute for Organic and Analytical Chemistry, 28359 Bremen, Germany.

[b] University of Bremen, Institute of Inorganic Chemistry and Crystallography, 28359 Bremen, Germany.

* Corresponding authors:

nachtsheim@uni-bremen.de

hupf@uni-bremen.de

Table of content

1 General Information ... 4

1.1 General analytical and experimental details ... 4

1.2 Luminescence measurements ... 5

1.3 Purity of chemicals and guests ... 9

1.4 Preparation of samples for phosphorescence measurements ... 10

2 Overview of photophysical data of host-guest systems ... 11

2.1 Various hosts with 0.1 wt% coronene (3) ... 11

2.2 Comparison of coronene- and coronene-d_{12} -based host-guest RTP systems with selected other hosts from the literature ... 12

2.3 Various hosts with 0.1 wt% coronene-d_{12} ... 13

2.4 4-Br DBI (2e) with various guests (0.1 wt%) ... 14

2.5 Comparison of 2e with other hosts from the literature ... 16

2.6 Various other host-guest systems ... 17

3 Photophysical properties of substituted dibenzyl isophthalate hosts doped with 0.1 wt% coronene (3) or coronene-d_{12} ... 18

3.1 UV-vis absorption and fluorescence emission spectra of coronene (3) and coronene-d_{12} in solution ... 18

3.2 Substituted dibenzyl isophthalates hosts with 0.1 wt% coronene ... 19

3.3 Substituted dibenzyl isophthalates hosts with 0.1 wt% coronene-d_{12} ... 43

3.4 Effect of different coronene concentrations in the 4-Br DBI (2e) on the luminescence emission ... 50

4 Photophysical properties of guests in solution and in host/guest-systems with DBI hosts ... 51

4.1 Photophysical properties of 0.1 wt% guests in the 4-Br DBI (2e) host ... 51

4.2 Effect of different pyrene-d_{10} concentrations in the 4-Br DBI (2e) on the luminescence emission ... 76

4.3 Photophysical properties of other isophthalate ester based host-guest systems 77

5 Preparation of compounds 92

5.1 General procedures 92

5.2 Preparation of isophthalic acid esters 93

5.3 Preparation of starting materials and guests 113

6 NMR spectra 125

6.1 NMR spectra of compounds 125

7 TD-DFT Computations 162

7.1 Computational Methodology 162

8 Stability tests 170

8.1 TGA/DSC measurements 170

8.2 Photostability tests 171

9 Spectra used for quantum yield determinations 174

9.1 Color scheme 174

9.2 Quantum yield spectra for DBIs + coronene (0.1 wt%) 174

9.3 Quantum yield spectra for DBIs + coronene-d_{12} (0.1 wt%) 178

9.4 Quantum yield spectra for 4-Br DBI (2e) + different guests (0.1 wt%) 179

9.5 Other quantum yield spectra 182

10 References 184

1 General Information

1.1 General analytical and experimental details

Unless otherwise stated, all reactions with moisture- or oxygen-sensitive reagents were performed using standard Schlenk techniques under a nitrogen atmosphere. Reagents were used as received from their commercial supplier. Dry tetrahydrofuran (THF) and DCM (DCM) were obtained from an *inert* PS-MD-6 solvent purification system. Dry DMF was received from a commercial supplier. Yields refer to isolated yields of compounds estimated to be >95% pure as determined by ^{1}H-NMR spectroscopy.

Kugelrohr distillations were performed on a *Büchi* B-585 Kugelrohr apparatus. The terminal bulb containing the crude mixture was placed about two thirds into the tube. In all cases, the product was collected from the first bulb attached to the terminal bulb at the described temperatures and pressures.

Column chromatography was performed on silica gel (0.040 – 0.063 mm) with the solvents given in the procedures.

^{1}H- and ^{13}C-NMR spectra were recorded on a *Bruker* Avance Neo 600 MHz spectrometer and a *Bruker* Avance Neo 600 MHz spectrometer at 24 °C. Chemical shifts for 1H-NMR spectra were reported as *δ* (parts per million) relative to the residual signal of $CHCl_3$ at 7.26 ppm (s), or DMSO-d_6 at 2.50 ppm (quin). Chemical shifts for ^{13}C-NMR spectra were reported as *δ* (parts per million) relative to the signal of $CDCl_3$ at 77.0 ppm (t), or DMSO-d_6 at 39.5 ppm (sept.). The following abbreviations were used to describe splitting patterns: br = broad, s = singlet, d = doublet, t = triplet, q = quartet, quin = quintet, sept = septet, m = multiplet. Coupling constants *J* are given in Hertz.

HR-ESI and HR-APCI mass spectra were recorded on a *Bruker* impact II mass spectrometer. High resolution (HR) EI mass spectra were recorded on the double focusing mass spectrometer ThemoQuest MAT 95 XL from *Finnigan* MAT. Low resolution EI mass spectra were recorded either on the same system or on an Agilent 5977A Series GC/MSD system (for GC-MS, the minimum *m/z* measured was 85). Low resolution APCI mass spectra were recorded on a *Advion* Expression CMS^{L} via ASAP probe or direct inlet. All signals were reported with the quotient from mass to charge *m/z*.

IR spectra were recorded on a *Nicolet* Thermo iS10 scientific spectrometer with a diamond ATR unit. The absorption bands are reported in cm^{-1}.

Melting points (Mp.) were determined on a *Büchi* M-5600 Melting Point and are uncorrected.

Simultaneous TGA-DSC testing was carried out on a SDT Q600 Thermal Gravimetric Analyser using 6–10 mg of compound under a constant flow of nitrogen gas (20 mL/min). The oven was heated from 25 °C to 300 °C at 10 °C per minute. A reference (empty crucible) was run each day directly prior to measurements.

UV-vis measurements were performed on a *Shimadzu* UV-2700. Samples were measured in 10x10 mm quartz cuvettes.

1.2 Luminescence measurements

Fluorescence and phosphorescence spectra were recorded on a *Jasco* FP-8300 fluorescence spectrometer at 20 °C. All spectra are corrected. All spectra were measured with 5 nm excitation bandwidth and 5 nm emission bandwidth unless otherwise stated. Steady state luminescence and phosphorescence spectra were recorded using the PMU-830 Liquid nitrogen cooling unit and the PPH-150 sample holder. Unless otherwise stated, all host compounds and host-guest systems were measured as solids in BRAND® micro UV-cuvettes. Both were measured as prepared according to section 1.4.

Phosphorescence spectra and excitation scans were recorded in the phosphorescence mode. The chopping period was 25 ms, the delay time 13 ms and the integration time 5 ms. The data interval was set to 1 nm and the response time to 0.5 - 1.5 s depending on the sample.

All samples were photoactivated[1] before measurement by irradiation with a Convoy S2+ UV flashlight (365 nm, 4W, light flux ca. 2 W) for 1-5 s (repeated five times from a distance of ca. 10 cm), until the phosphorescence intensity was steady by eye (shorter lifetimes required shorter photoactivation times). Repetitions were carried out because the final intensity of the RTP could best be gauged by eye from its afterglow. The phosphorescence intensity at a suitable maximum was afterwards monitored in the spectrometer for steadiness before measurements were carried out.

Solutions for fluorescence measurements were measured in a 10x10 mm quartz cuvette in the standard sample holder at a concentration of c = 3.0 µg/mL. All photostability tests were also performed in the standard sample holder. Photostability tests were measured with the time course program of the spectrometer in the fluorescence mode due to time restrictions of the phosphorescence mode. All spectra are corrected for baseline fluorescence emission at the same wavelength, which was determined before measurement and subtracted afterwards.

Phosphorescence lifetime decays curves for guests with long lifetimes (> 300 ms) were measured on the *Jasco* FP-8300 spectrometer using the standard sample holder. A self-built light-blocking plate connected to a spring was employed, which was used as fast a manual shutter (Fig. S1).

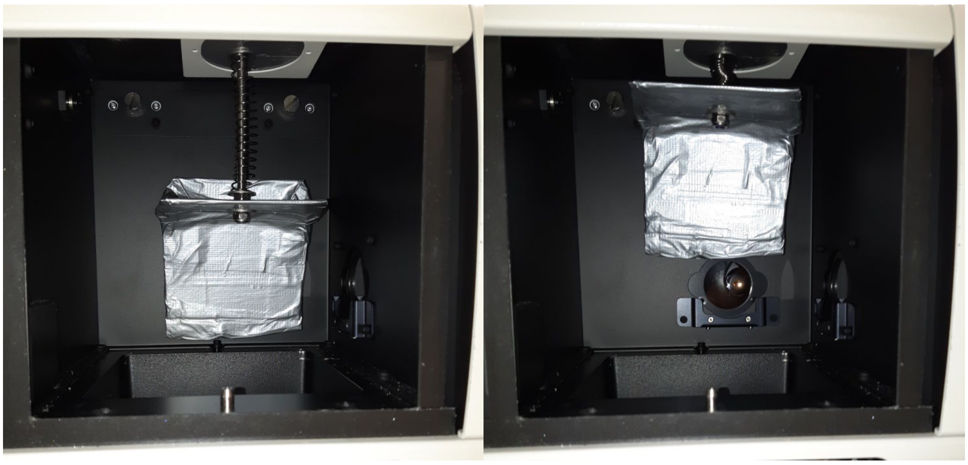

Figure S1. Self-built manual shutter connected to a spring in the spectrometer. Relaxed spring, closed shutter (left) and compressed spring, open shutter (right). The sample holder was removed for better visibility of the shutter.

When the manual shutter was closed, less than 0.01% of the excitation light reached the detector (compared to direct irradiation of the detector using a diffusor plate). The time it took to fully close the shutter was determined to be 40 ms on average, with a maximum closing time of 60 ms and a minimum closing time of 20 ms.

Phosphorescence lifetime decay curves were measured using the time course program in the fluorescence mode. The start time was set to 0 s and the end to 200 s. The data interval was set to 0.02 s, the response time 0.01 s and automatic AutoZero was enabled. The detector gain and if necessary the excitation and emission bandwidths were adjusted so that the intensity of the steady-state luminescence was sufficiently high to measure an initial phosphorescence intensity of at least 1000 a.u.

(10% of the detector limit). During the measurement, the manual shutter was opened until an equilibrium in intensity was reached, then the spring was released and a decay measured (Fig. S2). This action was performed one or multiple times during one measurement.

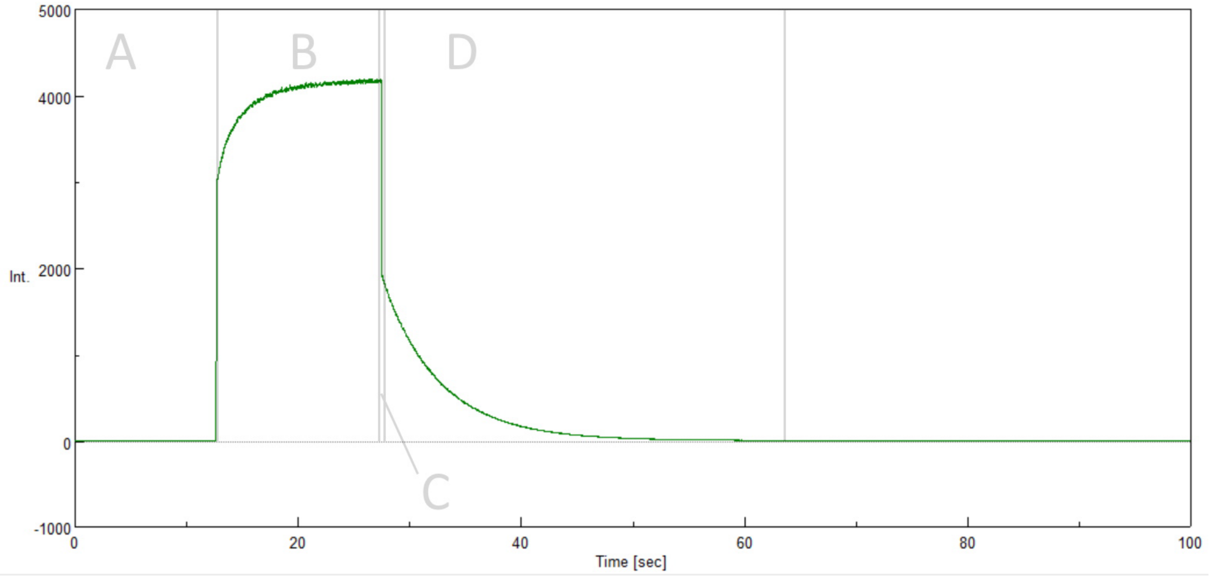

Figure S2. Example of a measured decay (4-OMe DBI (**2h**)). A: The manual shutter is closed. B: The manual shutter is opened until an equilibrium in intensity is reached. C: Closing of the shutter. D: Phosphorescence decay used for determination of the phosphorescence lifetime.

The phosphorescence lifetime was then determined using OriginPro software. Decays were fitted using exponential fitting functions ExpDecay# (# = 1,2,3; with # = 2 for bi-, and # = 3 for tri-exponential decays):

$$y = y_0 + \sum_{i=1}^{n} A_i e^{-\frac{-(x-x_0)}{\tau_i}}$$

y_0 was fixed at $y_0 = 0$, x_0 was fixed at the first data point of the decay. For the calculation of the function, the time interval between ≥100 ms after the first drop in intensity occurred and the time the intensity reached 10 - 1 a.u. was used. The fitting of the data was weighted by the “Variance ~ yfit” setting to account for the random noise of the instrument’s detector (fitting would otherwise lead to relatively huge residuals at absolutely low intensities very often, e.g. residuals of 5 a.u. at measured intensities of <5 a.u, which is multiple times higher than the instrumental error). For guests with <100 ms lifetimes, the phosphorescence lifetime measurement program was used to obtain decay curves (the chopping period was 400 ms, the measurement range 0 - 297 ms,

without delay time; fitting was performed from 106 - 297 ms)). For guests with 100 - 700 ms lifetimes, decay curves from the phosphorescence lifetime measurement program and the time course program were both measured, normalized and combined to give a decay curve that covered all relevant time ranges.

Average phosphorescence lifetimes were calculated using the formula:

$$\tau_{avg} = \frac{\sum_{i=1}^{n} B_i \tau_i}{\sum_{i=1}^{n} B_i}$$

The manual shutter experiment was also used to determine the ratio of phosphorescence intensity to steady-state luminescence intensity for guests with very long lifetimes. It was calculated by the dividing the first point of the decay curve after closing the shutter (start of section C in Fig. S2) by the intensity just before closing the shutter (end of section B in Fig. S2). For guests with short lifetimes, the phosphorescence lifetime program was used to determine the ratio.

Absolute quantum yields were determined using a *Jasco* ILF-835 integrating sphere. Quantum yields are corrected for the absorption of the cuvettes and for indirect excitations of the samples in the sphere. Quantum yields were calculated with the spectrometer software from incident light spectra (no sample), direct light spectra (of the sample) and indirect light spectra (of the sample).

Fluorescence quantum yields were obtained by subtracting phosphorescence spectra from the spectra that were used to calculate the total quantum yield. To do this, first the ratio of phosphorescence to steady-state luminescence intensity at the phosphorescence maximum was determined as described above. Then the phosphorescence spectrum was scaled to the intensity of the same wavelength as in the steady-state luminescence spectrum and multiplied by the ratio of phosphorescence to steady-state luminescence. This normalized phosphorescence spectrum was then subtracted from the steady-state luminescence spectrum to give a pure fluorescence spectrum, for which the quantum yield was determined in the same way as for the total quantum yield. The RTP quantum yield was then determined by subtracting the fluorescence quantum yield from the total quantum yield.

In cases in which the ($2 \cdot \lambda_{ex}$) nm maximum in the spectrum was overlapping with the phosphorescence spectrum (e.g. with pyrene as a guest), the spectrum was integrated only until before that maximum. Then the missing percentage of phosphorescence

intensity was determined by integrating the delayed phosphorescence spectrum and calculating the percentages of the areas integrated and missing. The measured phosphorescence quantum yields were then corrected by that ratio.

1.3 Purity of chemicals and guests

For full reproducibility and transparency of this work, we compiled a list of all aromatic starting materials, reagents and guests that we used. All chemicals except for benzaldehydes were used as received. Benzaldehydes were freshly distilled before use.

Table S1. Purity and supplier of all aromatic starting materials and guests used in this work.

Starting material/chemical; purity	Supplier	Supplier product code
Isophthaloyl dichloride, 98%	Alfa Aesar	A15904
Benzaldehyde ≥98%	VWR/Avantor	20863.291
(4-chlorophenyl)methanol, 97%	Maybridge	SB01197EA
o-Bromobenzaldehyde, 98%	BLDpharm	BD34552
3-Bromobenzyl alcohol, 99%	Fluorochem	001365
(4-Bromophenyl)methanol, 98%	BLDpharm	BD35197
4-Iodobenzaldehyde, 98%	BLDpharm	BD4431
Terephthalaldehyde, >98%	TCI	T0010
4-Formylbenzonitrile 98%	Apollo Scientific	OR4974
4-(Trifluoromethyl)benzaldehyde 95%	Apollo Scientific	PC53123
Methyl 4-formylbenzoate, >97%	Apollo Scientific	OR3659
4-(Hydroxymethyl)benzeneboronic acid, 96%	BLDpharm	BD5031
4-Methoxybenzaldehyde, 98%	abcr	AB108941
4-Fluorobenzaldehyde 99%	Apollo Scientific	PC3470
4-Methoxyphenol, 99%	Sigma Aldrich (Merck)	M18655
4-Bromophenol, 97%	Acros Organics	304411000
4-(Methylthio)benzaldehyde, 97%	abcr	AB174815
(4-(Phenylthio)phenyl)methanol, 98%	BLDpharm	BD146611
4-(Methylthio)benzenethiol	TCI	M1823
4-Bromo-2-methoxybenzyl alcohol, 97%	BLDpharm	BD210606
4-Bromobenzylamine, 98%	BLDpharm	BD01259492
1-Iodonaphthalene, 98%	BLDpharm	BD0392
Perylene, 95%	BLDpharm	BD147342
2-Biphenylboronic acid, 98%	BLDpharm	BD7980
2-Amino-3-bromobenzoic acid, 97%	BLDpharm	BD4524
Biphenyl-2-carboxylic acid, 98%	Alfa Aesar	A12049
Pyridine	Merck	107462
2,3,6,7,10,11-Hexamethoxytriphenylene, 97%[1]	BLDpharm	BD227281
Dimethyl 2,6-naphthalenedicarboxylate, 99%	BLDpharm	BD15323
1-Naphthoic acid, 98%	BLDpharm	BD22902
Fluoranthene 98%	Sigma Aldrich (Merck)	F807-5G

1,8-Naphthalic anhydride[1]	Fluka	70320
4-Dimethylaminobenzaldehyde, 99+ %	Acros Organics	168760250
Benzo[a]phenanthrene (purified by sublimation)	TCI	C0339
Benz[a]anthracene	HPC Standards	677532
Pyrene, 98%[2]	Acros Organics	180830250
Pyrene puriss. p.a., for fluorescence, ≥99.0% (GC) [2]	Sigma Aldrich (Merck)	82648
Pyrene-d_{10} 98 atom % D	Sigma Aldrich (Merck)	490695
Coronene, 95%	Acros Organics	110511000
Coronene-d_{12}	CDN Isotopes	D-0806

[1] These chemicals were purified by flash column chromatography on prior to use. [2] There was no difference in the phosphorescence spectra and properties between these two when they were doped into hosts. We used the pyrene with the higher purity for host/guest systems and the other pyrene for chemical synthesis.

1.4 Preparation of samples for phosphorescence measurements

Example for 0.1 wt% guest samples. Blank host compounds treated similarly without the addition of a DCM solution.

In a 4 mL glass vial with a septum, 100 mg of the host compound was combined with 100 µL of a 1 mg/mL DCM solution of the guest compound. The mixture was dried at 10 mbar (room temperature) by a cannula connected to a vacuum tube. After drying for 2 min, the vial was put into a pre-heated stainless-steel sample holder on a heating plate with the vacuum cannula still attached. The temperature of the sample holder was at least 160 °C or 10 °C above the melting point of the host compound. After melting, the vial was taken out of the holder and rotated a few times to achieve an even mixture. The cannula was then removed and the sample slowly cooled to rt in a Styrofoam container. For volatile aldehydes, the cannula was already removed before heating.

The only exception to this was the 4-$B(OH)_2$ DBI (**2u**), which was heated in an open vial.

Some host compounds did not solidify simply by cooling back to rt. After melting, these compounds were heated to and kept at 20 - 40 °C below their melting points until full crystallization was achieved.

2 Overview of photophysical data of host-guest systems

2.1 Various hosts with 0.1 wt% coronene (3)

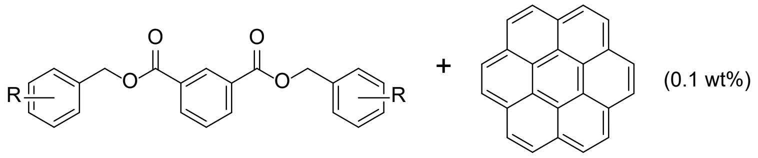

3

Table S2. Overview of the photophysical properties of various host-guest systems. DBI hosts with 0.1 wt% coronene (**3**). Bold numbers denote maxima.

DBI/Host	$\lambda_{ex, phos}$ [nm]	$\lambda_{em, FL}$ [nm]	φ_{FL} [%]	$\lambda_{em, phos}$ [nm]	φ_{Phos} [%]	$\tau_{Phos\ avg}$
4-H (**2a**)	**309**, 345	503	12.8	532, **569,** 616	0.4	5.11 ± 0.06 s
4-Cl (**2b**)	**323**, 347	**431**, 450, 458, 479, 488	11.6	534, **571**, 619	6.2	6.20 ± 0.01 s
2-Br (**2c**)	**313**, 348	450, **500**	5.8	**522**, 569, 619	13.6	1.58 ± 0.05 s
3-Br (**2d**)	**310**, 345	451, **501**	7.2	**523**, 570, 618	3.0	1.77 ± 0.01 s
4-Br (**2e**)	**322**, **347**, 385, 414, 431	430, **450**, 458, 479, 488	6.0	523, **570,** 618	10.5	3.61 ± 0.01 s
4-Br-2-OMe (**2f**)	325, **344**, 412	431, **450**, 458, 479, 486	4.5	**523,** 569, 617	26.5	1.29 ± 0.01 s
4-I (**2g**)	312, **347**, 413, 431	n.d.	0	**523,** 569, 618	42.5	65.4 ± 0.2 ms
4-OMe (**2h**)	**319**, **345**, 413	430, **448**, 456, 477, 510	22.2	522, 533, **569,** 617	2.4	5.70 ± 0.09 s
4-(OPh(4-OMe)) (**2i**)	322, **345**	429, **448**, 456, 477, 487	9.9	533, **570,** 617	1.1	3.08 ± 0.06 s
4-(OPh(4-Br)) (**2j**)	315, **348**	432, 465, 487, 498	9.2	**523,** 570, 618	9.7	0.94 ± 0.01 s
4-SMe (**2k**)	**348**, 413	431, **449**, 478	8.8	524, **570,** 617	4.2	5.13 ± 0.02 s
4-S(O)Me (**2l**)	**315**, 348	431, **450**, 457, 479, 488	31.9	523, 534, **570,** 618	1.9	5.36 ± 0.02 s
4-SO_2Me (**2m**)	**315**, 349, 415	431, 450, 457, 478	6.9	534, **570,** 618	1.5	4.91 ± 0.15 s
4-SPh (**2n**)	**348**, 415, 432	431, **449**, 478, 486	19.6	532, **569,** 617	3.7	6.46 ± 0.01 s
4-(SPh(4-SMe)) (**2o**)	**350**, 414, 431	431, **449**, 479, 488	20.7	**523,** 570, 617	1.0	2.12 ± 0.04 s
4-(SPh(4-Br)) (**2p**)	333, **348**, 415, 433	432, **450**, 479, 488	3.8	**524, 570,** 619	19.8	1.95 ± 0.03 s
4-CHO (**2q**)	**318**, **351**, 415, 433	432, **451**, **459**, 480, 488	2.6	535, **572,** 619	1.0	4.57 ± 0.10 s
4-CN (**2r**)	**316**, 349	432, **451**, 459, 480, 488	13.2	535, **571,** 619	2.8	6.39 ± 0.01 s

4-CO_2Me (**2s**)	**322**, **347**, 414	431, **450**, 458, 479, 488	19.0	534, **571,** 618	4.8	5.17 ± 0.06 s
4-CF_3 (**2t**)	**308**, 353, 413	432, 450, 466, **488**, 521	42.3	534, **571,** 618	0	3.27 ± 0.06 s
4-B$(OH)_2$ (**2u**)	**358,** 391, 414, 432	436, **450**, 459, 479, 488	11.5	534, **571,** 618	1.0	6.32 ± 0.07 s
**S1**	312, **345**, 412	438, 449, 457, 447, 502	3.7	**522,** 569, 617	1.8	1.46 ± 0.02 s
**S2**	313, 331, **347**	**501**, 522	12.5	**523,** 570, 618	7.9	0.55 ± 0.01 s

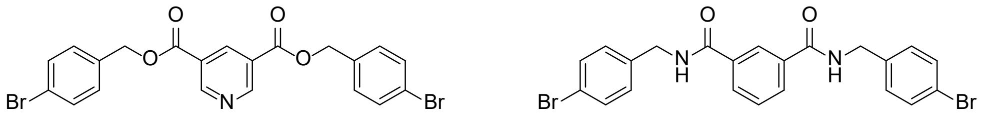

S1 **S2**

Figure S3. Molecular structures of hosts **S1** and **S2**.

2.2 Comparison of coronene- and coronene-d_{12}-based host-guest RTP systems with selected other hosts from the literature

Table S3. Overview of the photophysical properties of coronene- and coronene-d_{12} –based host-guest RTP systems under air. To our best knowledge, these systems include the currently known host-guest systems with the longest RTP lifetimes and quantum yields for these guests.

Guest	Host	φ_{FL} [%]	φ_{Phos} [%]	τ_{Phos} [s]	Comment	Reference
**Coronene**	**ZIF-8**	8.7	1.8	7.42		Ref.[2]
**Coronene**	**PMMA**	10.6	1.7	5.58		Ref.[2]
**Coronene**	**PMMA**	19	4	6.0		Ref.[3]
**Coronene**	**β-estradiol**	25	3.4	6.0		Ref.[4]
**Coronene**	**melamine formaldehyde (MF) polymer**	28.7	22.7	4.83		Ref.[5]
**Coronene**	**4-Methoxybenzophenone**	n.d.	n.d.	4.12	Given as two lifetimes for bi-exponential decay in ref.	Ref.[6]
**Coronene-d_{12}**	**4-Methoxybenzophenone**	n.d.	n.d.	6.54	Given as two lifetimes for bi-exponential decay in ref.	Ref.[6]
**Coronene-d_{12}**	**ZIF-8**	10.1	3.7	22.4		Ref.[2]
**Coronene-d_{12}**	**PMMA**	20	12	23		Ref.[3]
**Coronene-d_{12}**	**β-estradiol**	24	11	17		Ref.[7]

2.3 Various hosts with 0.1 wt% coronene-d12

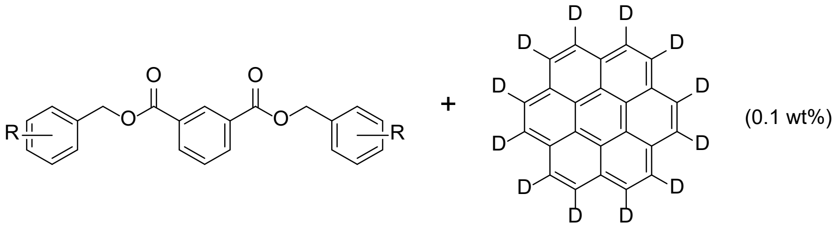

Table S4. Overview of the photophysical properties of various host-guest systems. Selected DBI hosts with 0.1 wt% coronene-d_{12}. Bold numbers denote maxima.

DBI/Host	$\lambda_{ex,\ phos}$ [nm]	$\lambda_{em,\ FL}$ [nm]	φ_{FL} [%]	$\lambda_{em,\ phos}$ [nm]	φ_{Phos} [%]	$\tau_{Phos\ avg}$
4-Cl (**2b**)	**327**, 344, 393, 409, 431	430, 448, **456**, 476, 486	19.1	532, **569,** 616	15.7	18.8 ± 0.1 s
4-Br (**2e**)	**328**, 344, 392, 408, 431	430, 448, **455**, 476, 485	7.3	522, **568,** 616	25.9	5.90 ± 0.02 s
4-I (**2g**)	322, **346**, 386, 392, 407, 430	n.d.	0.5	523, **567,** 616	76.7	96.3 ± 0.3 ms
4-CN (**2r**)	**326**, **347**, 392, 414, 431	431, 449, **456**, 478, 485	13.5	533, **569,** 616	7.3	21.0 ± 0.1 s
4-OMe (**2h**)	**318**, **345**, 392, 408, 429	430, 454, **474**, 504	25.9	513, 531, 544, **567,** 614	8.6	19.8 ± 0.1 s
4-SPh (**2n**)	**352**, 392, 414, 431	430, 448, **455**, 476, 484	21.8	531, **567,** 614	9.4	20.0 ± 0.1 s
4-B(OH)$_2$ (**2u**)	310, **353**, 417, 437	433, **456,** 478, 486, 509	17.5	532, **569,** 616	2.0	16.6 ± 0.2 s

2.4 4-Br DBI (2e) with various guests (0.1 wt%)

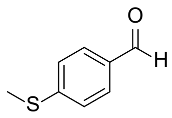

G1

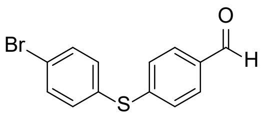

G2

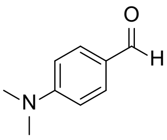

G3

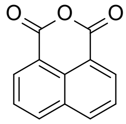

G4

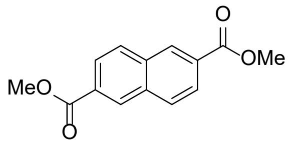

G5

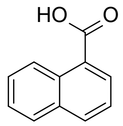

G6

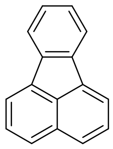

G7

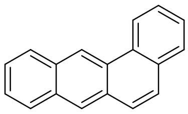

G8

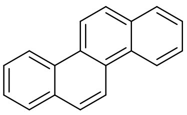

G9

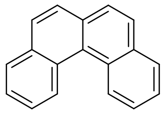

G10

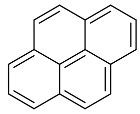

G11

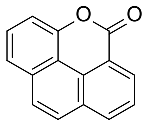

G12

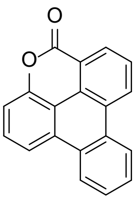

G13

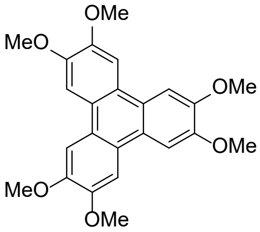

G14

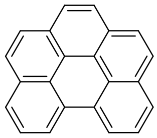

G15

Figure S4. Overview of guest structures.

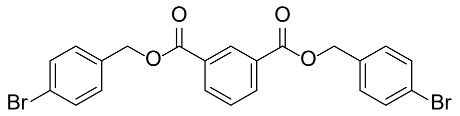

Table S5. Overview of the photophysical properties of the 4-Br DBI (**2e**) host with various guests (0.1 wt%). Bold numbers denote maxima.

Guest	$\lambda_{ex, phos}$ [nm]	$\lambda_{em, FL}$ [nm]	φ_{FL} [%]	$\lambda_{em, phos}$ [nm]	φ_{Phos} [%]	$\tau_{Phos\ avg}$
**G1** [a]	321	398	n.d.	481	2.8	26.3 ± 0.6 ms
**G2** [a]	327	410	n.d.	489	9.8	21.1 ± 0.6 ms
**G3**	340	412	1.8	**461**, **482**, 613	1.3	75.6 ± 0.5 ms
**G4**	**333**, 342	446	6.9	**538**, 584, 633	3.5	161 ± 2 ms
**G5**	303, 338, **353**	381	17.7	513, **552**, 595	0.5	289 ± 2 ms
**G6**	314, **326**	431	15.7	502, 514, **543**, 585	5.4	119 ± 1 ms
**G7**	326, 348, **363**	461	31.2	**546**, 562, 593, 645	0.5	175 ± 2 ms
**G8**	**304**, 333, 346, 362	**493**, 529, 570	36.0	**604**, 662, 725	0.5	83.9 ± 0.5 ms
**G9**	312, **325**	**400**, 423	43.8	**512**, **551**, 597	1.9	546 ± 8 ms
**G10**	**307**, 320, 356, 375	417	12.2	**505**, **546**, 587	1.4	623 ± 4 ms
**G11**	328, **344**	387, 398, **468**	26.9	**595**, 608, 644, 658, 715	1.1	294 ± 1 ms
**G12** [a]	**312**, **322**, 356, **374**	401, **417**	5.2	502, 506, **542**, 586	3.8	688 ± 11 ms
**G13** [a]	**322**, 346, 365	389, **407**, 427	14.0	456, **490**, 523	1.0	52.9 ± 0.6 ms
**G14**	313	403, 420, **456**, 481	7.2	461, **484**, 520, 569	0.4	66.3 ± 2.2 ms
**G15**	**307**, 330, **347**, 369, 391	412, 425, 436, 449, **482**, 504	38.2	**625**, 692	0.3	156 ± 1 ms
**Pyrene-*d*$_{10}$**	327, **344**	385, 396, **469**	19.2	**593**, 606, 655, 715	2.7	1.26 ± 0.02 s

[a] No previous report of phosphorescence yet.

2.5 Comparison of 2e with other hosts from the literature

Table S6. Overview of the photophysical properties of the 4-Br DBI (**2e**) host with various guests (0.1 wt%) in comparison with various other, comparable host-guest RTP systems (dopant amoung may differ).

Guest	Host	φ_{FL} [%]	φ_{Phos} [%]	τ_{Phos}	Comment	Reference
**G1** [a]	**2e**	n.d.	2.8	26.3 ± 0.6 ms		This work
**G2** [a]	**2e**	n.d.	9.8	21.1 ± 0.6 ms		This work
**G3**	**2e**	1.8	1.3	75.6 ± 0.5 ms		This work
**G3**	**1-(4-bromophenyl)-1*H*-imidazole (1BBI)**	n.d.	14.8	122 ms	No LE phosphorescence at 612 nm	Ref.[8]
**G4**	**2e**	6.9	3.5	161 ± 2 ms		This work
**G4**	**pentachloropyridine (PCP)**	17.3 (φ_{total})		363 ms	No separate φ_{Phos} given	Ref.[9]
**G4**	**phthalic anhydride (PA)**	5.9 (φ_{total})		492 ms	No separate φ_{Phos} given	Ref.[9]
**G4**	**1,2-dicyanobenzene (DCB)**	23.0 (φ_{total})		603 ms	No separate φ_{Phos} given	Ref.[9]
**G4**	**melamine formaldehyde (MF) polymer**	n.d.	2.9	574 ms		Ref.[10]
**G5**	**2e**	17.7	0.5	289 ± 2 ms		This work
**G5**	**poly(vinyl alcohol) film (PVA)**	24.0 (φ_{total})	"<1 %"	710 ms		Ref.[11]
**G6**	**2e**	15.7	5.4	119 ± 1 ms		This work
**G6**	**PVA film**	n.d.		55 ms		Ref.[12]
**G6**	**poly (methyl methacrylate) film (PMMA)**	n.d.		n.d.	RTP only after N_2 purging	Ref.[12]
**G6**	**melamine formaldehyde (MF) polymer**	n.d.	2.9	1.14 s		Ref.[10]
**G7**	**2e**	31.2	0.5	175 ± 2 ms		This work
**G7**	**melamine formaldehyde (MF) polymer**	40.6	N/A	357 ms		Ref.[5]
**G8**	**2e**	36.0	0.5	83.9 ± 0.5 ms		This work
**G8**	**poly(styrene) film**	n.d.	n.d.	225 ms		Ref.[13]
**G9**	**2e**	43.8	1.9	546 ± 8 ms		This work
**G9**	**β-estradiol**	n.d.	0.91	1.4 s		Ref.[14]
**G10**	**2e**	12.2	1.4	623 ± 4 ms		This work
**G10**	**benzophenone**	6.2 (φ_{total})		835 ms		Ref.[15]
**G10**	**4-OMe-benzophenone**	2.6 (φ_{total})		860 ms		Ref.[15]
**G11**	**2e**	26.9	1.1	294 ± 1 ms		This work
**G11**	**benzophenone**	n.d.	0.81	311 ms		Ref.[16]
**G11**	**triphenylene**	n.d.	n.d.	410 ms		Ref.[17]
**G11**	**β-estradiol**	43	0.05	0.20 s		Ref.[14]
**G11**	**melamine formaldehyde (MF) polymer**	62.8	NA	392 ms		Ref.[5]
**G12** [a]	**2e**	5.2	3.8	688 ± 11 ms		This work
**G13** [a]	**2e**	14.0	1.0	52.9 ± 0.6 ms		This work
**G14**	**2e**	7.2	0.4	66.3 ± 2.2 ms		This work
**G14**	**poly(acrylonitrile) film**	16.5	6.1	302 ms		Ref.[18]
**G15**	**2e**	38.2	0.3	156 ± 1 ms		This work
**G15**	**β-estradiol**	46	0.1	0.34 s		Ref.[14]

[a] No previous report of phosphorescence yet.

2.6 Various other host-guest systems

Table S7. Overview of the photophysical properties of various other host-guest systems and blank hosts. Bold numbers denote maxima.

Host	Guest	$\lambda_{ex, phos}$ [nm]	$\lambda_{em, FL}$ [nm]	φ_{FL} [%]	$\lambda_{em, phos}$ [nm]	φ_{Phos} [%]	$\tau_{Phos\ avg}$
4-Cl (**2b**)	**pyrene-*d*$_{10}$** (0.1 wt%)	347	385, 397, **473**	35.9	**593**, 606, 655, 721	1.4	2.48 ± 0.04 s
4-Br (**2e**)	**pyrene-*d*$_{10}$** (0.1 wt%)	327, **344**	385, 396, **469**	19.2	**593**, 606, 655, 715	2.7	1.26 ± 0.02 s
4-I (**2g**)	**pyrene-*d*$_{10}$** (0.1 wt%)	329, **346**, 377	385, 398, **476**	3.4	**595**, 606, 656, 717	4.1	124 ± 2 ms
p-toluuic acid (95 wt%) 4-Br (**2e**) (5 wt%)	**pyrene-*d*$_{10}$** (0.01 wt%)	331, **349**	399	n.d.	**595**, 606, 656, 726	n.d.	1.12 ± 0.03 s
p-toluuic acid (99.89 wt%) 4-Br (**2e**) (0.1 wt%)	**pyrene-*d*$_{10}$** (0.01 wt%)	331, **349**	395	n.d.	**597**, 657, 722	n.d.	0.99 ± 0.03 s
4-SMe (**2k**)	-	351	438	n.d.	507	n.d.	20.8 ± 0.8 ms
4-OMe (**2h**)	**G1** (0.1 wt%)	347	409	n.d.	**493**, 501	n.d.	34.3 ± 0.3 ms
4-Br (**2e**)	**G1** (0.1 wt%)	321	398	n.d.	481	2.8	26.3 ± 0.6 ms
4-Br (**2e**)	**G2** (0.1 wt%)	327	410	n.d.	489	9.8	21.1 ± 0.6 ms
4-(SPh(4-Br)) (**2p**) (10 wt%) 4-Br (**2e**) (90 wt%)	-	349	415	n.d.	499	n.d.	n.d.
4-(SPh(4-Br)) (**2p**) (50 wt%) 4-Br (**2e**) (50 wt%)	-	348	415	n.d.	**523**, 544[1], 559	n.d.	n.d.
4-(SPh(4-Br)) (**2p**) (75 wt%) 4-Br (**2e**) (25 wt%)	-	362	**376**, **392**	n.d.	**505**, **544**	n.d.	n.d.
4-(SPh(4-Br)) (**2p**) (90 wt%) 4-Br (**2e**) (10 wt%)	-	363	**378**, **392**	n.d.	**506**, **544**	n.d.	n.d.
4-(SPh(4-Br)) (**2p**)	-	364	**377**, **393**	n.d.	**505**, 544	n.d.	32.9 ± 0.2 ms

[1] Broad emission.

3 Photophysical properties of substituted dibenzyl isophthalate hosts doped with 0.1 wt% coronene (3) or coronene-d_{12}

3.1 UV-vis absorption and fluorescence emission spectra of coronene (3) and coronene-d_{12} in solution

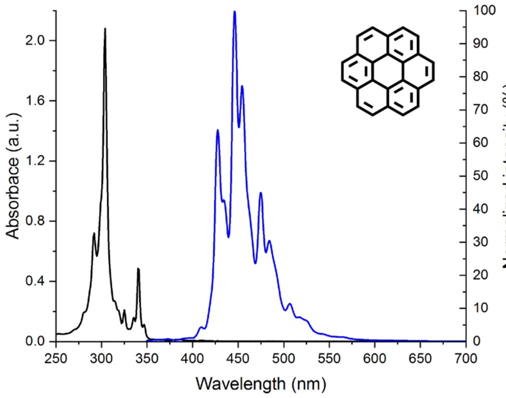

Figure S5. UV-absorption spectrum and normalized fluorescence emission spectrum of coronene (**3**) in DCM.

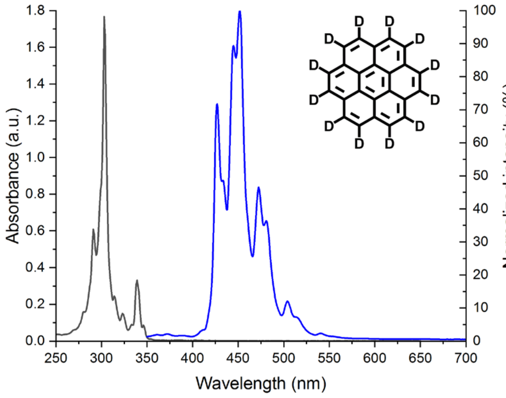

Figure S6. UV-absorption spectrum and normalized fluorescence emission spectrum of coronene-d_{12} in DCM.

3.2 Substituted dibenzyl isophthalates hosts with 0.1 wt% coronene

3.2.1 4-H DBI (2a)

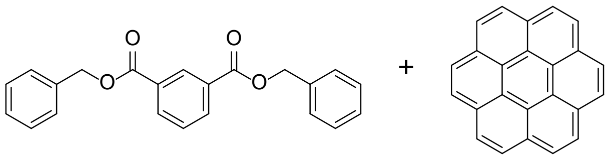

(0.1 wt%)

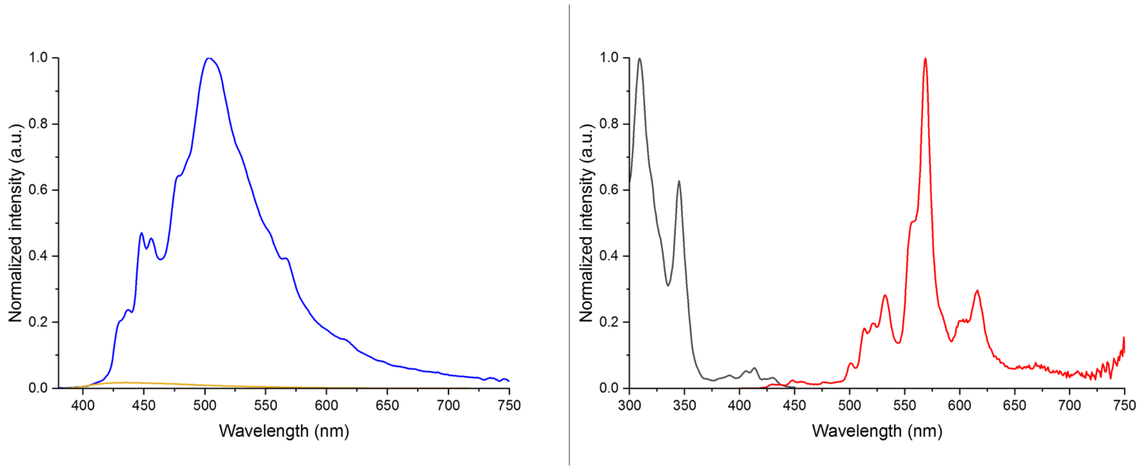

Figure S7. (Left) normalized steady-state luminescence spectrum (blue line) and normalized background spectrum (host without guest, orange line) of 0.1 wt% coronene (**3**) in 4-H DBI (**2a**). Excitation at 350 nm. (Right) phosphorescence excitation spectrum (emission at 571 nm, black line) and phosphorescence emission spectrum (red line, excitation at 350 nm) of the same host/guest system. The total luminescence quantum yield was determined to be φ = 0.132. The fluorescence quantum yield was determined to be φ_{FL} = 0.128 and the phosphorescence quantum yield φ_{Phos} = 0.004.

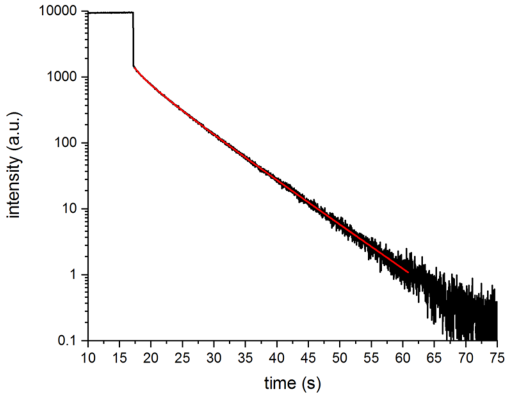

Figure S8. Phosphorescence lifetime decay curve (black line) and fitted lifetime decay (red line) of coronene (**3**) in 4-H DBI (**2a**) (0.1 wt%). Excitation at 350 nm, emission at 571 nm. The lifetime was determined to be: τ_1 = 0.98 ± 0.08 s (11 %); τ_2 = 3.41 ± 0.15 s (26 %); τ3 = 6.50 ± 0.03 s (64 %); τ_{avg} = 5.11 ± 0.06 s.

3.2.2 4-Cl DBI (2b)

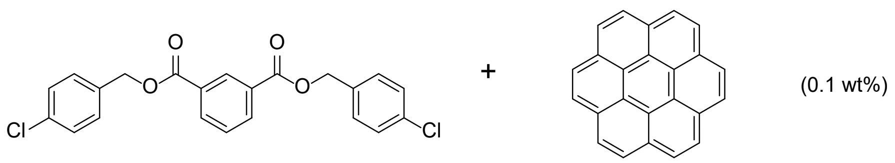

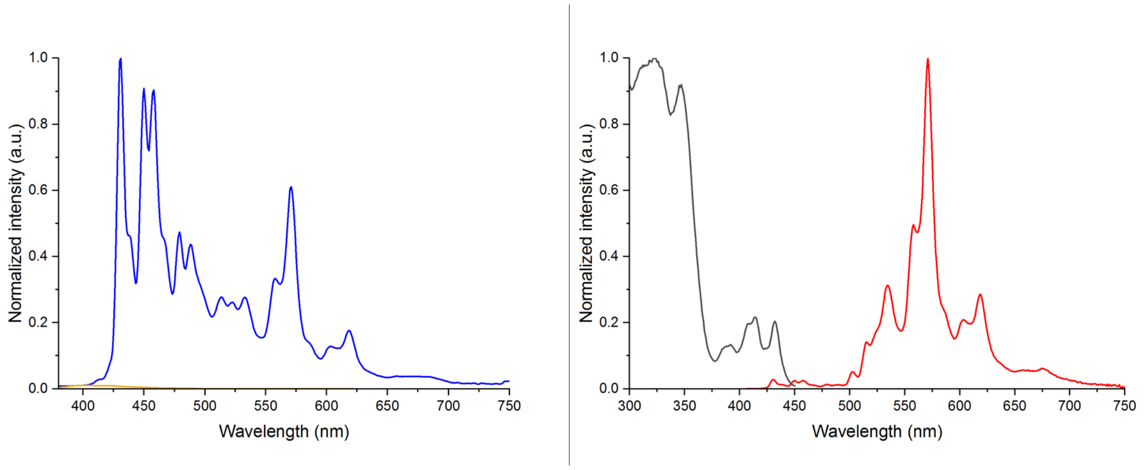

Figure S9. (Left) normalized steady-state luminescence spectrum (blue line) and normalized background spectrum (host without guest, orange line) of 0.1 wt% coronene (**3**) in 4-Cl DBI (**2b**). Excitation at 350 nm. (Right) phosphorescence excitation spectrum (emission at 571 nm, black line) and phosphorescence emission spectrum (red line, excitation at 350 nm) of the same host/guest system. The total luminescence quantum yield was determined to be φ = 0.178. The fluorescence quantum yield was determined to be φ_{FL} = 0.116 and the phosphorescence quantum yield φ_{Phos} = 0.062.

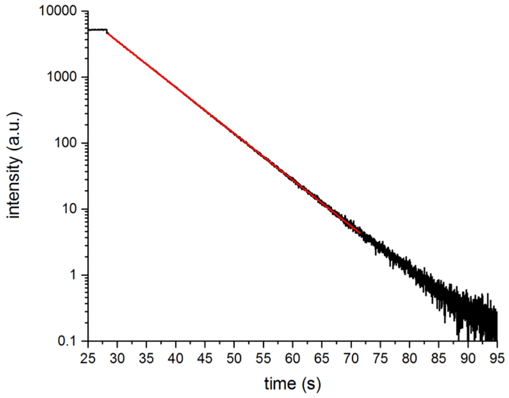

Figure S10. Phosphorescence lifetime decay curve (black line) and fitted lifetime decay (red line) of coronene (**3**) in 4-Cl DBI (**2b**) (0.1 wt%). Excitation at 350 nm, emission at 571 nm. The lifetime was determined to be: τ = 6.20 ± 0.01 s.

3.2.3 2-Br DBI (2c)

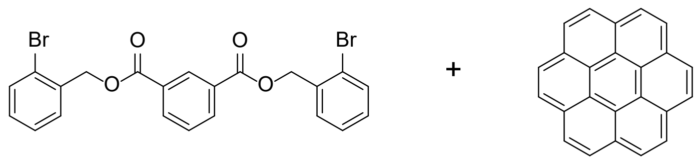

(0.1 wt%)

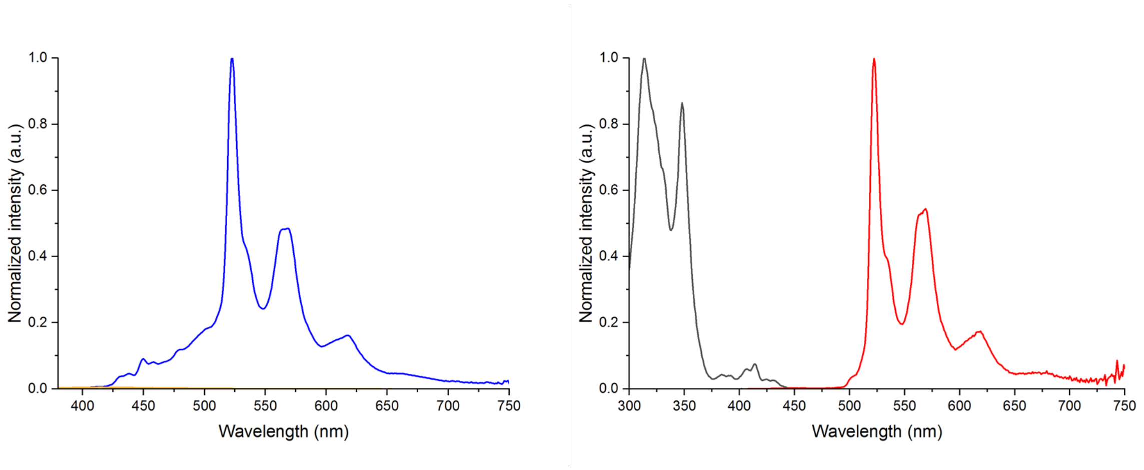

Figure S11. (Left) normalized steady-state luminescence spectrum (blue line) and normalized background spectrum (host without guest, orange line) of 0.1 wt% coronene (**3**) in 2-Br DBI (**2c**). Excitation at 350 nm. (Right) phosphorescence excitation spectrum (emission at 571 nm, black line) and phosphorescence emission spectrum (red line, excitation at 350 nm) of the same host/guest system. The total luminescence quantum yield was determined to be φ = 0.194. The fluorescence quantum yield was determined to be φ_{FL} = 0.058 and the phosphorescence quantum yield φ_{Phos} = 0.136.

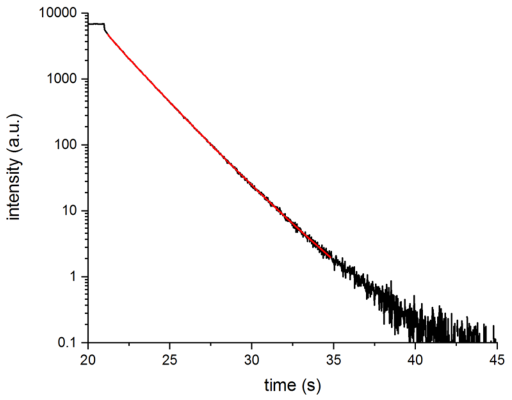

Figure S12. Phosphorescence lifetime decay curve (black line) and fitted lifetime decay (red line) of coronene (**3**) in 2-Br DBI (**2c**) (0.1 wt%). Excitation at 350 nm, emission at 571 nm. The lifetime was determined to be: τ_1 = 0.46 ± 0.04 s (5 %); τ_2 = 1.58 ± 0.02 s (88 %); τ_3 = 2.43 ± 0.12 s (7 %); τ_{avg} = 1.58 ± 0.05 s.

3.2.4 3-Br DBI (2d)

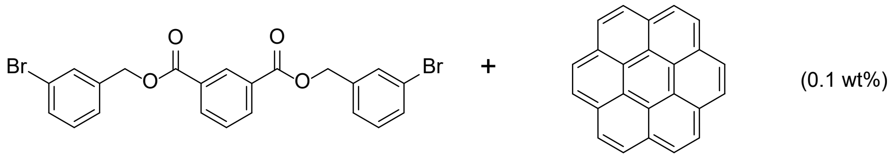

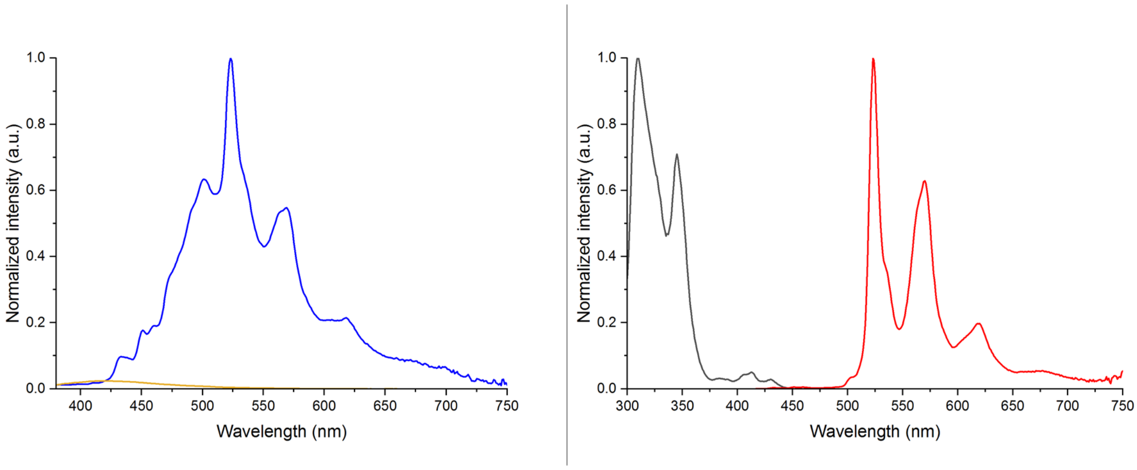

Figure S13. (Left) normalized steady-state luminescence spectrum (blue line) and normalized background spectrum (host without guest, orange line) of 0.1 wt% coronene (**3**) in 3-Br DBI (**2d**). Excitation at 350 nm. (Right) phosphorescence excitation spectrum (emission at 571 nm, black line) and phosphorescence emission spectrum (red line, excitation at 350 nm) of the same host/guest system. The total luminescence quantum yield was determined to be φ = 0.102. The fluorescence quantum yield was determined to be φ_{FL} = 0.072 and the phosphorescence quantum yield φ_{Phos} = 0.030.

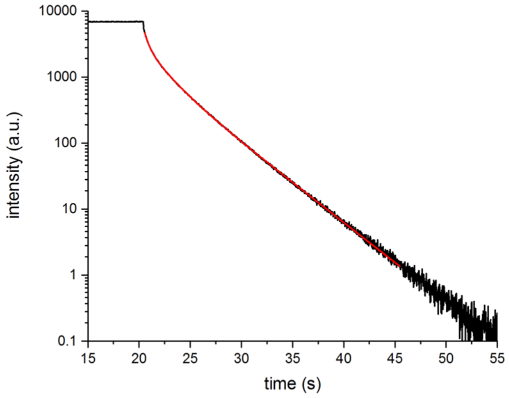

Figure S14. Phosphorescence lifetime decay curve (black line) and fitted lifetime decay (red line) of coronene (**3**) in 3-Br DBI (**2d**) (0.1 wt%). Excitation at 350 nm, emission at 571 nm. The lifetime was determined to be: τ_1 = 0.42 ± 0.01 s (37 %); τ_2 = 1.60 ± 0.01 s (32 %); τ_3 = 3.59 ± 0.01 s (30 %); τ_{avg} = 1.77 ± 0.01 s.

3.2.5 4-Br DBI (2e)

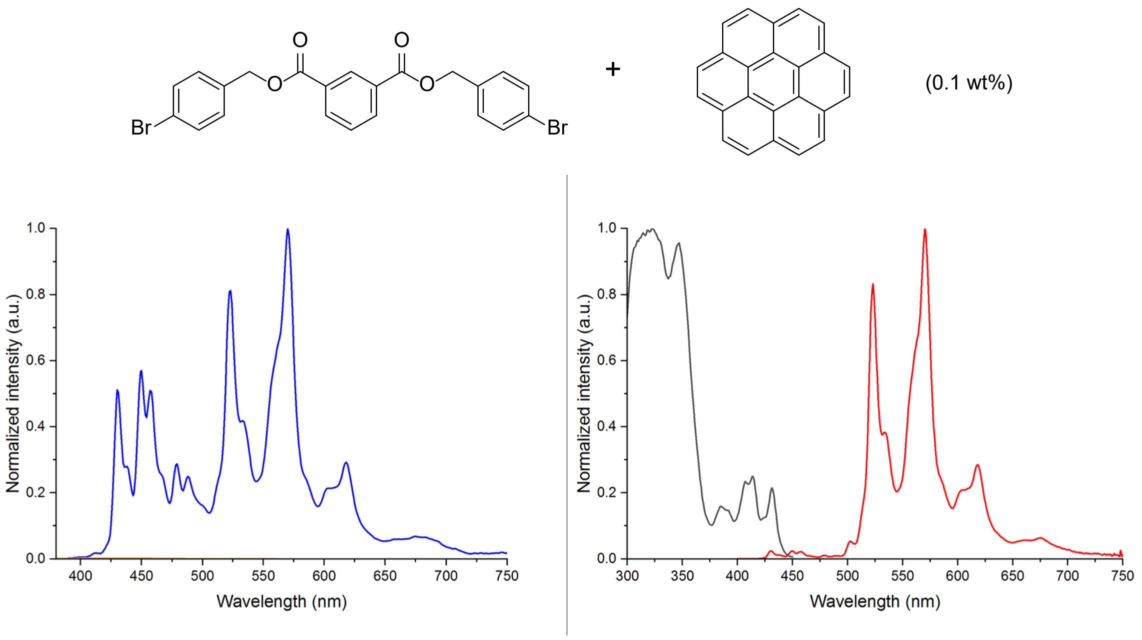

Figure S15. (Left) normalized steady-state luminescence spectrum (blue line) and normalized background spectrum (host without guest, orange line) of 0.1 wt% coronene (**3**) in 4-Br DBI (**2e**). Excitation at 350 nm. (Right) phosphorescence excitation spectrum (emission at 571 nm, black line) and phosphorescence emission spectrum (red line, excitation at 350 nm) of the same host/guest system. The total luminescence quantum yield was determined to be φ = 0.165. The fluorescence quantum yield was determined to be φ_{FL} = 0.060 and the phosphorescence quantum yield φ_{Phos} = 0.105.

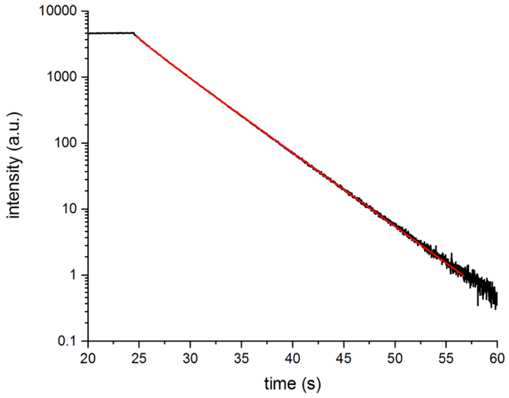

Figure S16. Phosphorescence lifetime decay curve (black line) and fitted lifetime decay (red line) of coronene (**3**) in 4-Br DBI (**2e**) (0.1 wt%). Excitation at 350 nm, emission at 571 nm. The lifetime was determined to be: τ_1 = 1.84 ± 0.03 s (14 %); τ_2 = 3.90 ± 0.01 s (86 %); τ_{avg} = 3.61 ± 0.01 s.

3.2.6 4-Br-2-OMe DBI (2f)

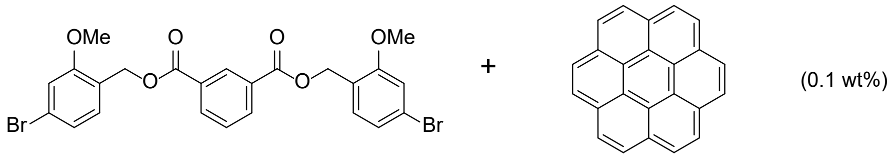

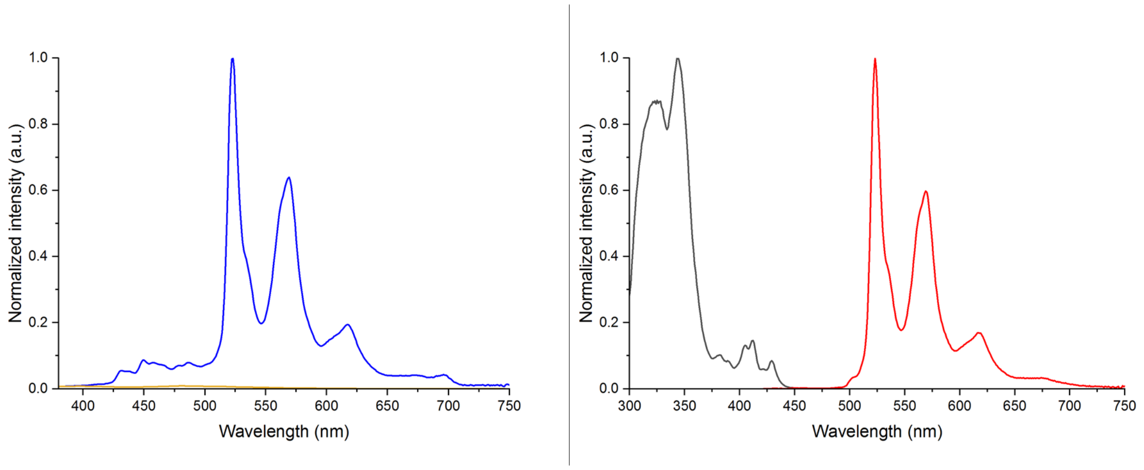

Figure S17. (Left) normalized steady-state luminescence spectrum (blue line) and normalized background spectrum (host without guest, orange line) of 0.1 wt% coronene (**3**) in 4-Br-2-OMe DBI (**2f**). Excitation at 350 nm. (Right) phosphorescence excitation spectrum (emission at 571 nm, black line) and phosphorescence emission spectrum (red line, excitation at 350 nm) of the same host/guest system. The total luminescence quantum yield was determined to be φ = 0.310. The fluorescence quantum yield was determined to be φ_{FL} = 0.045 and the phosphorescence quantum yield φ_{Phos} = 0.265.

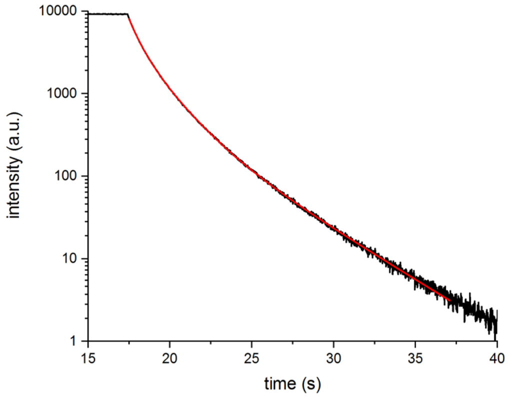

Figure S18. Phosphorescence lifetime decay curve (black line) and fitted lifetime decay (red line) of coronene (**3**) in 4-Br-2-OMe DBI (**2f**) (0.1 wt%). Excitation at 350 nm, emission at 571 nm. The lifetime was determined to be: τ_1 = 0.58 ± 0.01 s (44 %); τ_2 = 1.52 ± 0.01 s (47 %); τ_3 = 3.58 ± 0.01 s (9 %); τ_{avg} = 1.29 ± 0.01 s.

3.2.7 4-I DBI (2g)

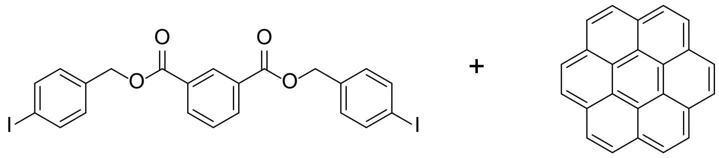

(0.1 wt%)

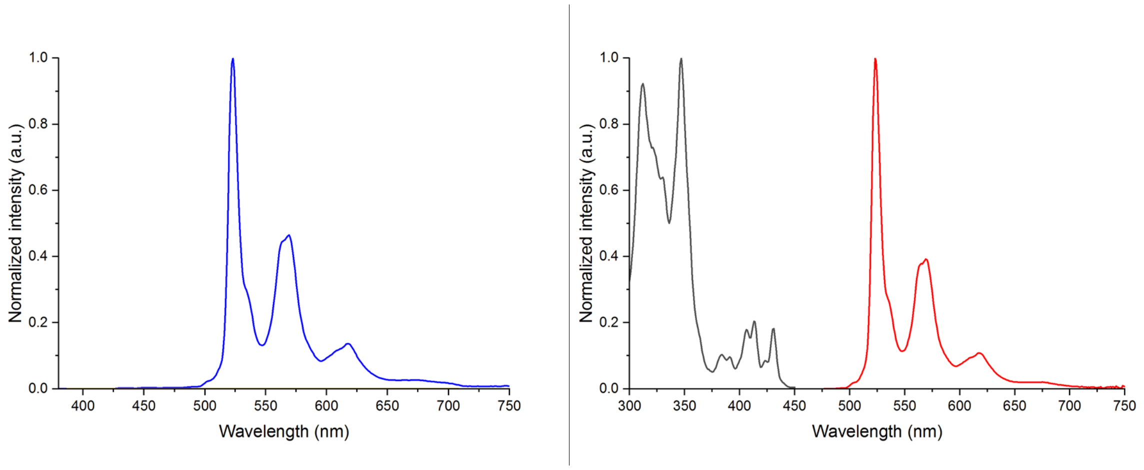

Figure S19. (Left) normalized steady-state luminescence spectrum (blue line) and normalized background spectrum (host without guest, orange line) of 0.1 wt% coronene (**3**) in 4-I DBI (**2g**). Excitation at 350 nm. (Right) phosphorescence excitation spectrum (emission at 571 nm, black line) and phosphorescence emission spectrum (red line, excitation at 350 nm) of the same host/guest system. The total luminescence quantum yield was determined to be φ = 0.425. The fluorescence quantum yield was determined to be φ_{FL} ~ 0.0 and the phosphorescence quantum yield φ_{Phos} = 0.425.

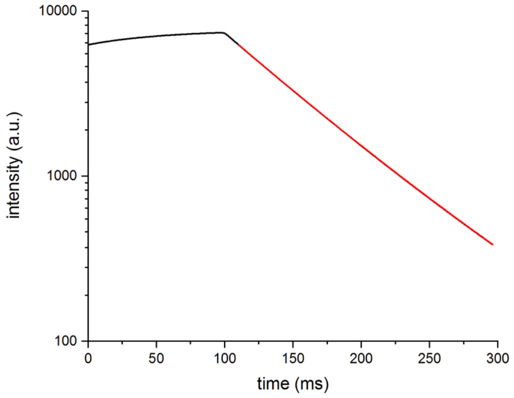

Figure S20. Phosphorescence lifetime decay curve (black line) and fitted lifetime decay (red line) of coronene (**3**) in 4-I DBI (**2g**) (0.1 wt%). Excitation at 350 nm, emission at 571 nm. The lifetime was determined to be: τ_1 = 55.6 ± 0.1 ms (78 %); τ_2 = 99.6 ± 0.7 ms (22 %); τ_{avg} = 65.4 ± 0.2 ms.

3.2.8 4-OMe DBI (2h)

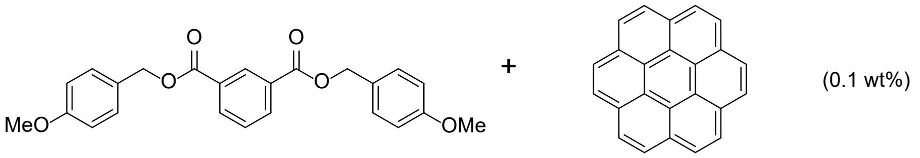

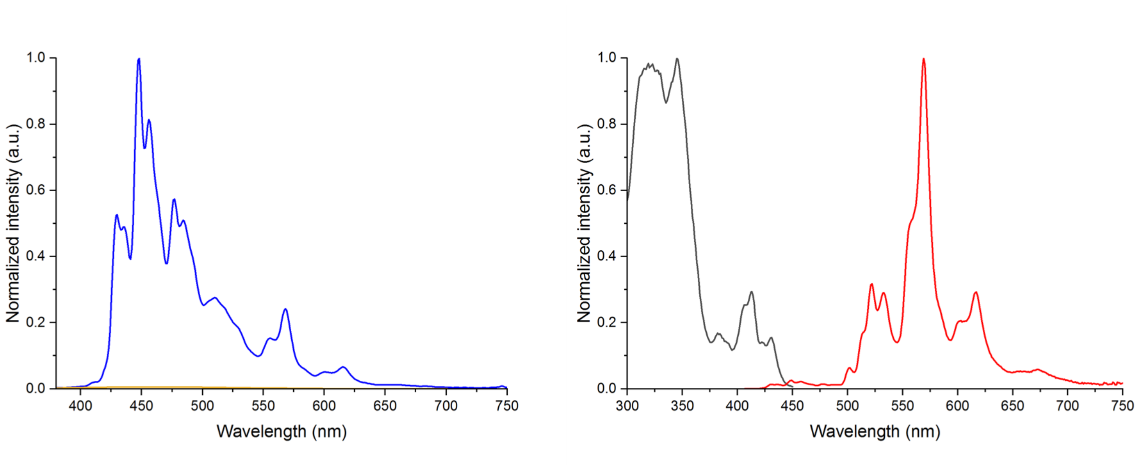

Figure S21. (Left) normalized steady-state luminescence spectrum (blue line) and normalized background spectrum (host without guest, orange line) of 0.1 wt% coronene (**3**) in 4-OMe DBI (**2h**). Excitation at 350 nm. (Right) phosphorescence excitation spectrum (emission at 571 nm, black line) and phosphorescence emission spectrum (red line, excitation at 350 nm) of the same host/guest system. The total luminescence quantum yield was determined to be φ = 0.246. The fluorescence quantum yield was determined to be φ_{FL} = 0.222 and the phosphorescence quantum yield φ_{Phos} = 0.024.

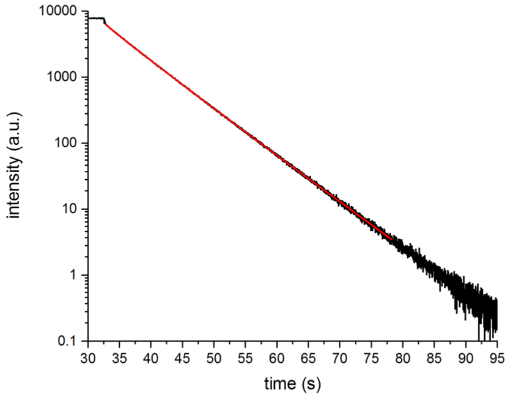

Figure S22. Phosphorescence lifetime decay curve (black line) and fitted lifetime decay (red line) of coronene (**3**) in 4-OMe DBI (**2h**) (0.1 wt%). Excitation at 350 nm, emission at 571 nm. The lifetime was determined to be: τ_1 = 0.78 ± 0.06 s (3 %); τ_2 = 4.46 ± 0.12 s (22 %); τ_3 = 6.28 ± 0.03 s (75 %); τ_{avg} = 5.70 ± 0.09 s.

3.2.9 4-(OPh(4-OMe)) DBI (2i)

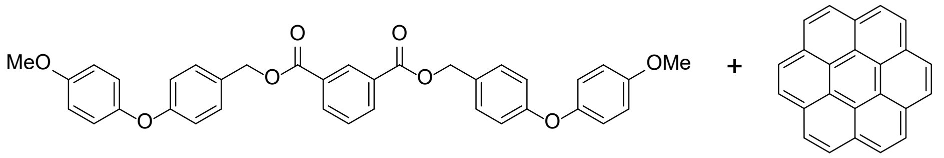

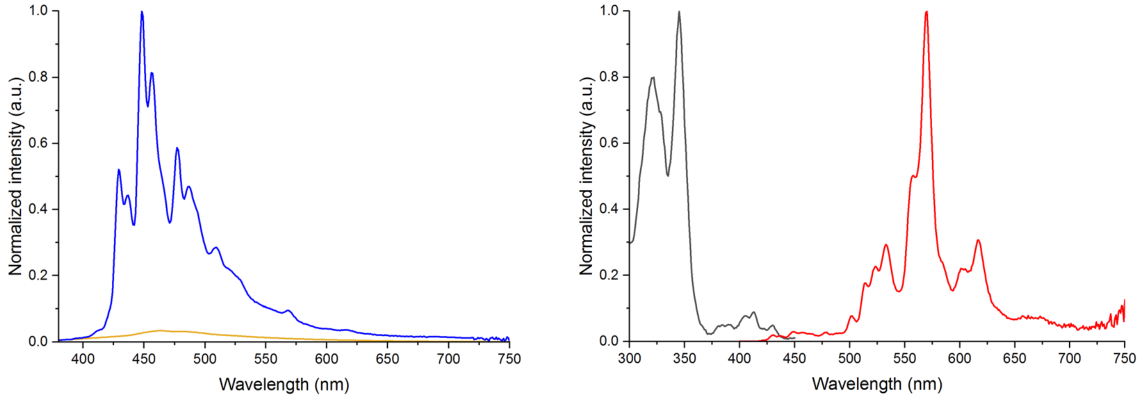

Figure S23. (Left) normalized steady-state luminescence spectrum (blue line) and normalized background spectrum (host without guest, orange line) of 0.1 wt% coronene (**3**) in 4-(OPh(4-OMe)) DBI (**2i**). Excitation at 350 nm. (Right) phosphorescence excitation spectrum (emission at 571 nm, black line) and phosphorescence emission spectrum (red line, excitation at 350 nm) of the same host/guest system. The total luminescence quantum yield was determined to be φ = 0.110. The fluorescence quantum yield was determined to be φ_{FL} = 0.099 and the phosphorescence quantum yield φ_{Phos} = 0.011.

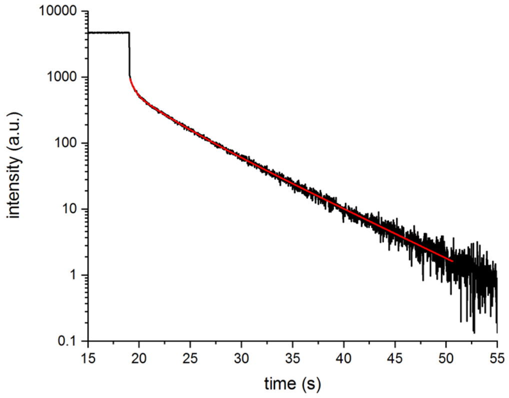

Figure S24. Phosphorescence lifetime decay curve (black line) and fitted lifetime decay (red line) of coronene (**3**) in 4-OPh(4-OMe) DBI (**2i**) (0.1 wt%). Excitation at 350 nm, emission at 571 nm. The lifetime was determined to be: τ_1 = 0.40 ± 0.02 s (36 %); τ_2 = 2.71 ± 0.13 s (25 %); τ_3 = 5.82 ± 0.05 s (39 %); τ_{avg} = 3.08 ± 0.06 s.

3.2.10 4-(OPh(4-Br)) DBI (2j)

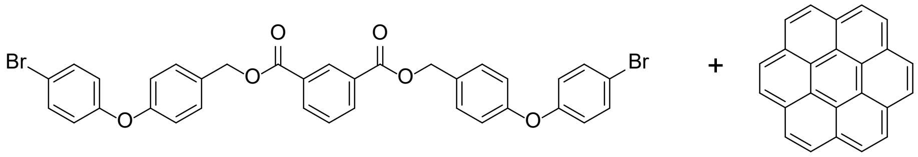

(0.1 wt%)

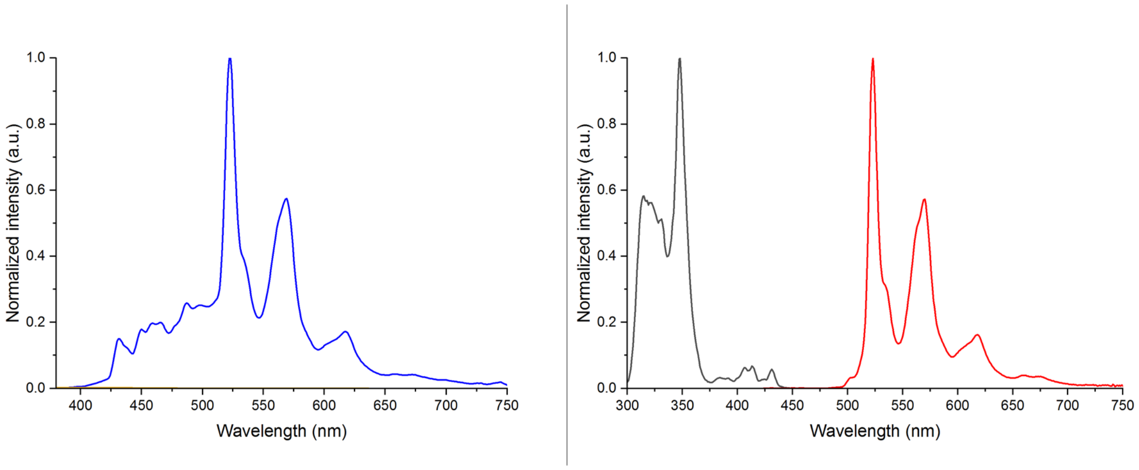

Figure S25. (Left) normalized steady-state luminescence spectrum (blue line) and normalized background spectrum (host without guest, orange line) of 0.1 wt% coronene (**3**) in 4-(OPh(4-Br)) DBI (**2j**). Excitation at 350 nm. (Right) phosphorescence excitation spectrum (emission at 571 nm, black line) and phosphorescence emission spectrum (red line, excitation at 350 nm) of the same host/guest system. The total luminescence quantum yield was determined to be φ = 0.189. The fluorescence quantum yield was determined to be φ_{FL} = 0.092 and the phosphorescence quantum yield φ_{Phos} = 0.097.

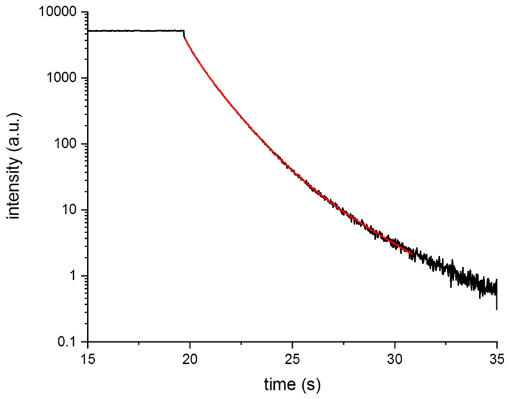

Figure S26. Phosphorescence lifetime decay curve (black line) and fitted lifetime decay (red line) of coronene (**3**) in 4-(OPh(4-Br)) DBI (**2j**) (0.1 wt%). Excitation at 350 nm, emission at 571 nm. The lifetime was determined to be: τ_1 = 0.40 ± 0.01 s (27 %); τ_2 = 1.06 ± 0.01 s (69 %); τ_3 = 2.55 ± 0.05 s (4 %); τ_{avg} = 0.94 ± 0.01 s.

3.2.11 4-SMe DBI (2k)

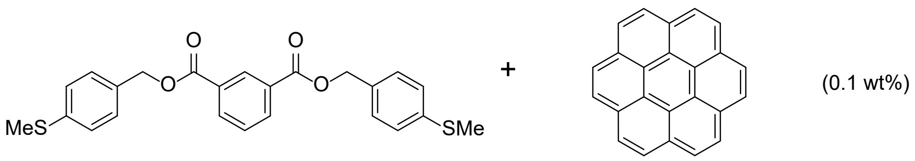

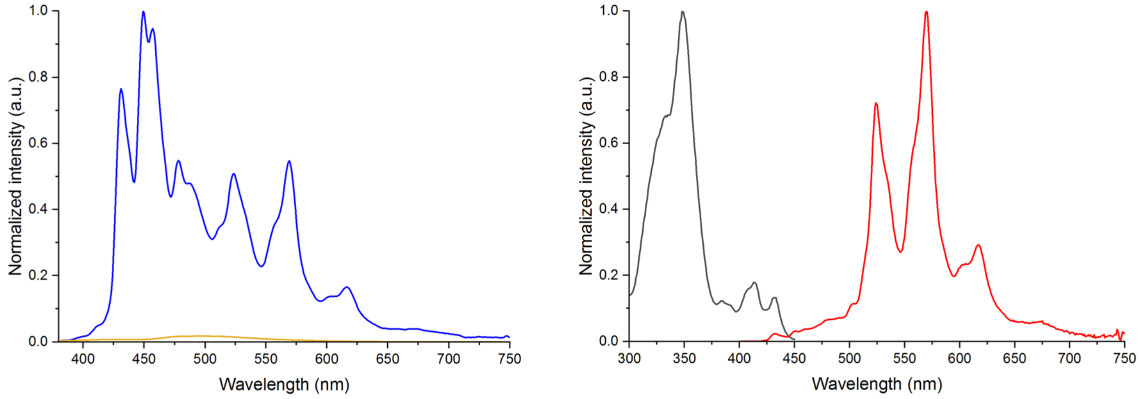

Figure S27. (Left) normalized steady-state luminescence spectrum (blue line) and normalized background spectrum (host without guest, orange line) of 0.1 wt% coronene (**3**) in 4-SMe DBI (**2k**). Excitation at 350 nm. (Right) phosphorescence excitation spectrum (emission at 571 nm, black line) and phosphorescence emission spectrum (red line, excitation at 350 nm) of the same host/guest system. The total luminescence quantum yield was determined to be φ = 0.130. The fluorescence quantum yield was determined to be φ_{FL} = 0.088 and the phosphorescence quantum yield φ_{Phos} = 0.042.

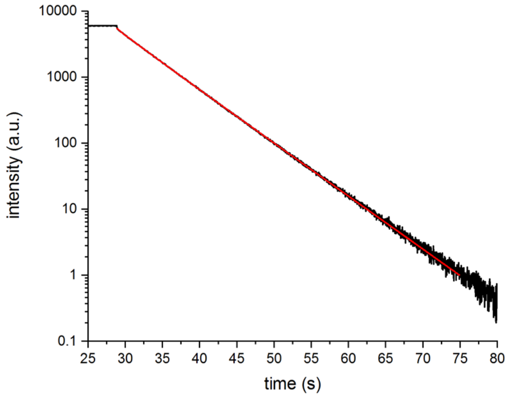

Figure S28. Phosphorescence lifetime decay curve (black line) and fitted lifetime decay (red line) of coronene (**3**) in 4-SMe DBI (**2k**) (0.1 wt%). Excitation at 350 nm, emission at 571 nm. The lifetime was determined to be: τ_1 = 0.32 ± 0.04 s (2 %); τ_2 = 3.28 ± 0.13 s (8 %); τ_3 = 5.42 ± 0.01 s (90 %); τ_{avg} = 5.13 ± 0.02 s.

3.2.12 4-S(O)Me DBI (2l)

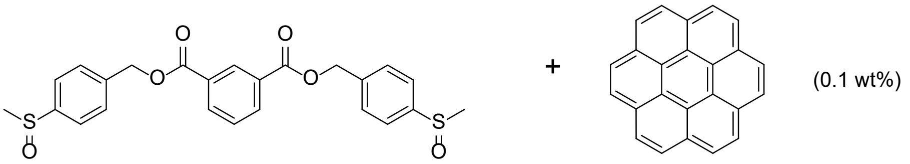

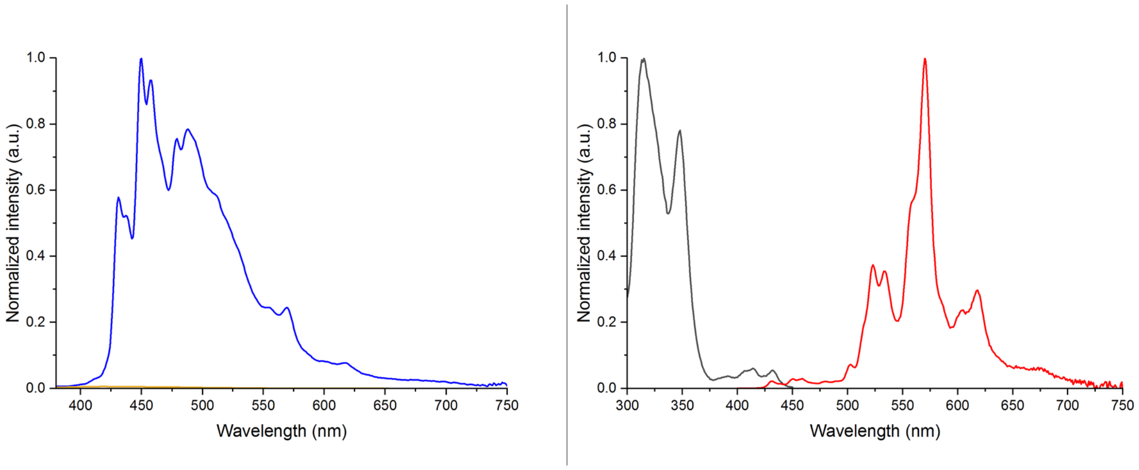

Figure S29. (Left) normalized steady-state luminescence spectrum (blue line) and normalized background spectrum (host without guest, orange line) of 0.1 wt% coronene (**3**) in 4-S(O)Me DBI (**2l**). Excitation at 350 nm. (Right) phosphorescence excitation spectrum (emission at 571 nm, black line) and phosphorescence emission spectrum (red line, excitation at 350 nm) of the same host/guest system. The total luminescence quantum yield was determined to be φ = 0.338. The fluorescence quantum yield was determined to be φ_{FL} = 0.319 and the phosphorescence quantum yield φ_{Phos} = 0.019.

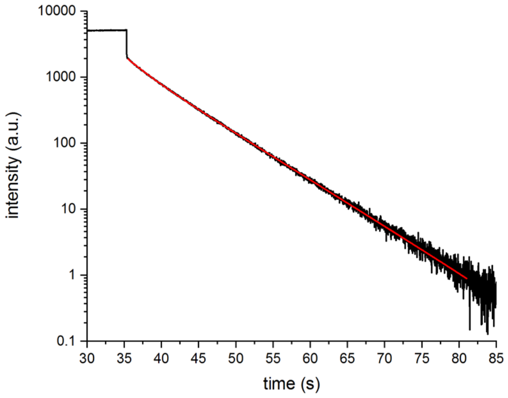

Figure S30. Phosphorescence lifetime decay curve (black line) and fitted lifetime decay (red line) of coronene (**3**) in 4-S(O)Me DBI (**2l**) (0.1 wt%). Excitation at 350 nm, emission at 571 nm. The lifetime was determined to be: τ_1 = 6.19 ± 0.01 s (78 %); τ_2 = 2.48 ± 0.04 s (22 %); τ_{avg} = 5.36 ± 0.02 s.

3.2.13 4-SO_2Me DBI (2m)

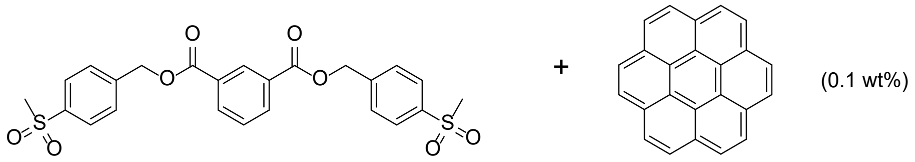

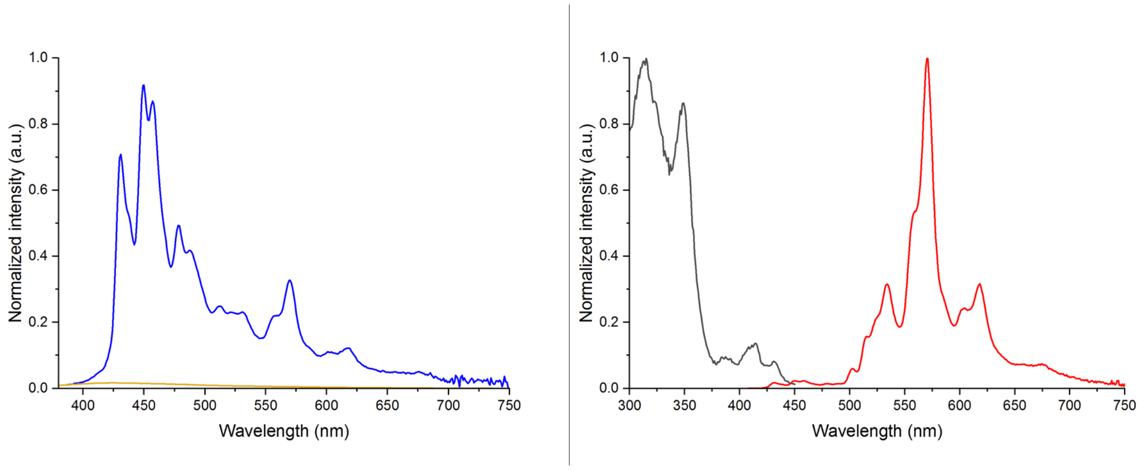

Figure S31. (Left) normalized steady-state luminescence spectrum (blue line) and normalized background spectrum (host without guest, orange line) of 0.1 wt% coronene (**3**) in 4-SO_2Me DBI (**2m**). Excitation at 350 nm. (Right) phosphorescence excitation spectrum (emission at 571 nm, black line) and phosphorescence emission spectrum (red line, excitation at 350 nm) of the same host/guest system. The total luminescence quantum yield was determined to be φ = 0.084. The fluorescence quantum yield was determined to be φ_{FL} = 0.069 and the phosphorescence quantum yield φ_{Phos} = 0.015.

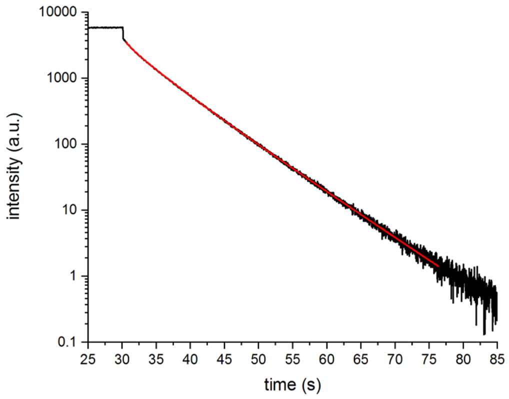

Figure S32. Phosphorescence lifetime decay curve (black line) and fitted lifetime decay (red line) of coronene (**3**) in 4-SO_2Me DBI (**2m**) (0.1 wt%). Excitation at 350 nm, emission at 571 nm. The lifetime was determined to be: τ_1 = 1.01 ± 0.04 s (11 %); τ_2 = 3.86 ± 0.08 s (33 %); τ_3 = 6.33 ± 0.25 s (56 %); τ_{avg} = 4.91 ± 0.15 s.

3.2.14 4-SPh DBI (2n)

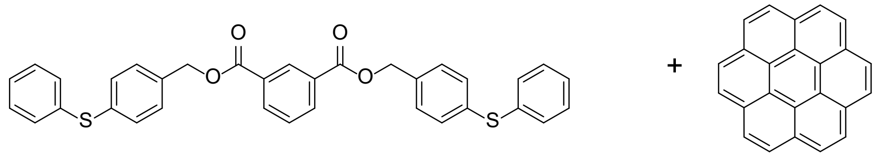

(0.1 wt%)

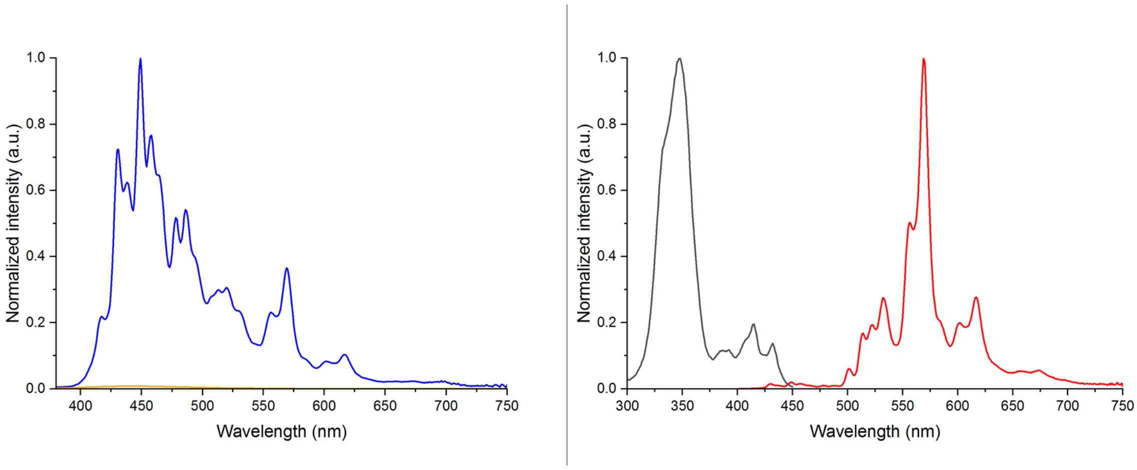

Figure S33. (Left) normalized steady-state luminescence spectrum (blue line) and normalized background spectrum (host without guest, orange line) of 0.1 wt% coronene (**3**) in 4-SPh DBI (**2n**). Excitation at 350 nm. (Right) phosphorescence excitation spectrum (emission at 571 nm, black line) and phosphorescence emission spectrum (red line, excitation at 350 nm) of the same host/guest system. The total luminescence quantum yield was determined to be φ = 0.233. The fluorescence quantum yield was determined to be φ_{FL} = 0.196 and the phosphorescence quantum yield φ_{Phos} = 0.037.

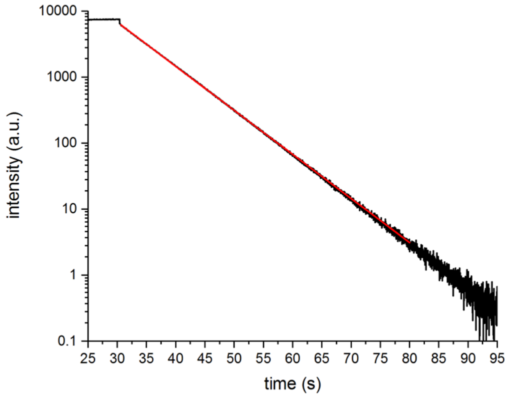

Figure S34. Phosphorescence lifetime decay curve (black line) and fitted lifetime decay (red line) of coronene (**3**) in 4-SPh DBI (**2n**) (0.1 wt%). Excitation at 350 nm, emission at 571 nm. The lifetime was determined to be: τ = 6.46 ± 0.01 s.

3.2.15 4-(SPh(4-SMe)) DBI (2o)

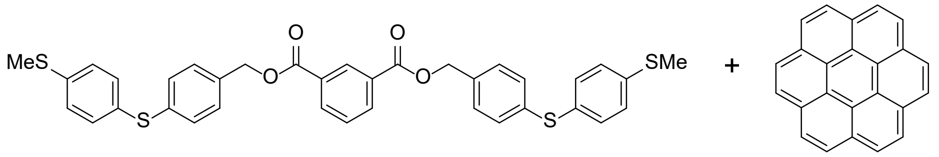

(0.1 wt%)

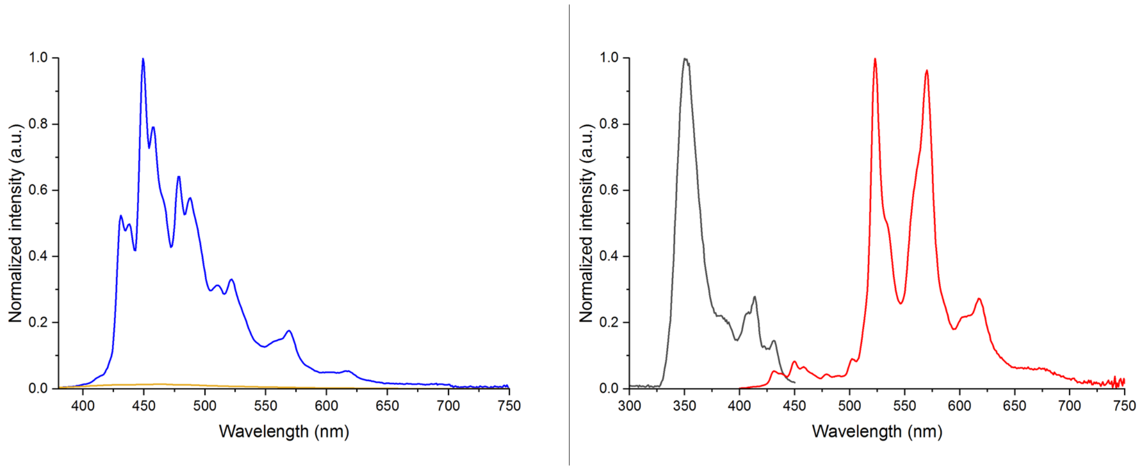

Figure S35. (Left) normalized steady-state luminescence spectrum (blue line) and normalized background spectrum (host without guest, orange line) of 0.1 wt% coronene (**3**) in 4-(SPh(4-SMe)) DBI (**2o**). Excitation at 350 nm. (Right) phosphorescence excitation spectrum (emission at 571 nm, black line) and phosphorescence emission spectrum (red line, excitation at 350 nm) of the same host/guest system. The total luminescence quantum yield was determined to be φ = 0.217. The fluorescence quantum yield was determined to be φ_{FL} = 0.207 and the phosphorescence quantum yield φ_{Phos} = 0.010.

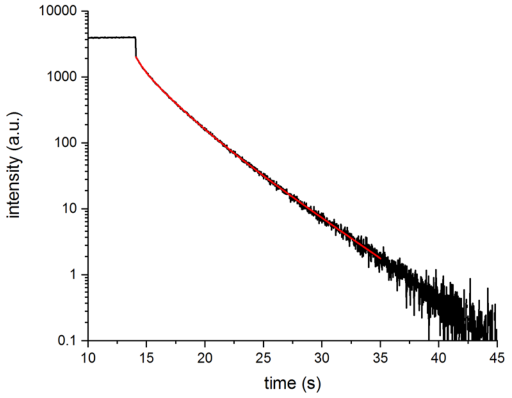

Figure S36. Phosphorescence lifetime decay curve (black line) and fitted lifetime decay (red line) of coronene (**3**) in 4-(SPh(4-SMe)) DBI (**2o**) (0.1 wt%). Excitation at 350 nm, emission at 571 nm. The lifetime was determined to be: τ_1 = 0.53 ± 0.02 s (21 %); τ_2 = 1.94 ± 0.04 s (50 %); τ_3 = 3.6 ± 0.04 s (29 %); τ_{avg} = 2.12 ± 0.04 s.

3.2.16 4-(SPh(4-Br)) DBI (2p)

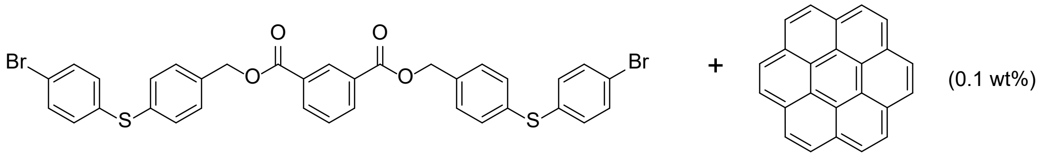

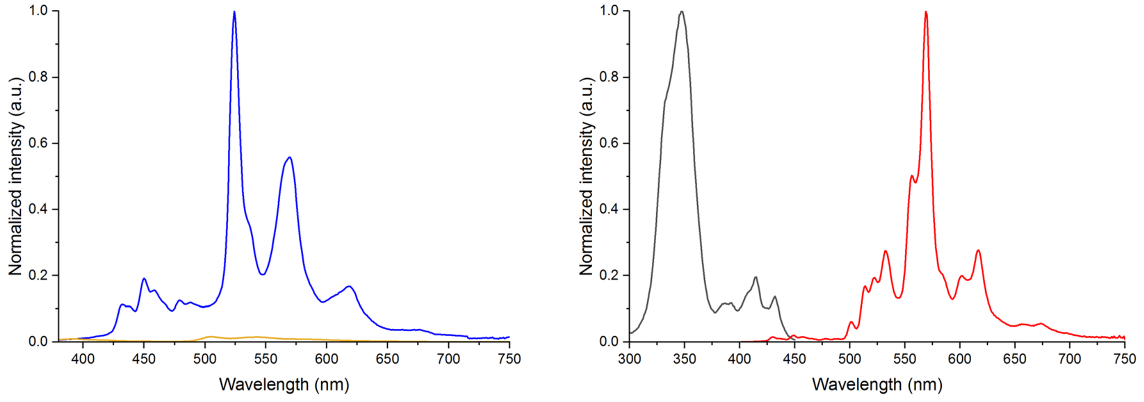

Figure S37. (Left) normalized steady-state luminescence spectrum (blue line) and normalized background spectrum (host without guest, orange line) of 0.1 wt% coronene (**3**) in 4-(SPh(4-Br)) DBI (**2p**). Excitation at 350 nm. (Right) phosphorescence excitation spectrum (emission at 571 nm, black line) and phosphorescence emission spectrum (red line, excitation at 350 nm) of the same host/guest system. The total luminescence quantum yield was determined to be φ = 0.236. The fluorescence quantum yield was determined to be φ_{FL} = 0.038 and the phosphorescence quantum yield φ_{Phos} = 0.198.

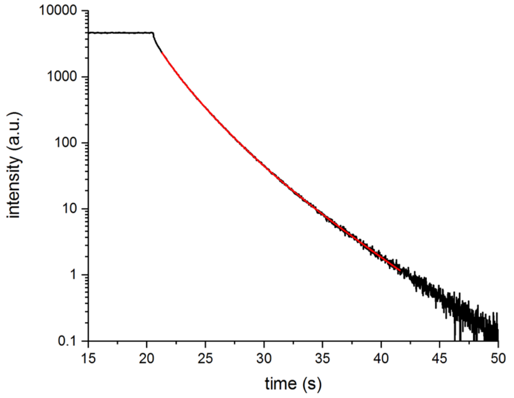

Figure S38. Phosphorescence lifetime decay curve (black line) and fitted lifetime decay (red line) of coronene (**3**) in 4-(SPh(4-Br)) DBI (**2p**) (0.1 wt%). Excitation at 350 nm, emission at 571 nm. The lifetime was determined to be: τ_1 = 0.83 ± 0.03 s (18 %); τ_2 = 1.89 ± 0.02 s (68 %); τ_3 = 3.57 ± 0.03 s (15 %); τ_{avg} = 1.95 ± 0.03 s.

3.2.17 4-CHO DBI (2q)

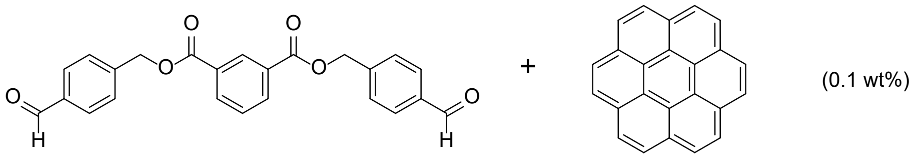

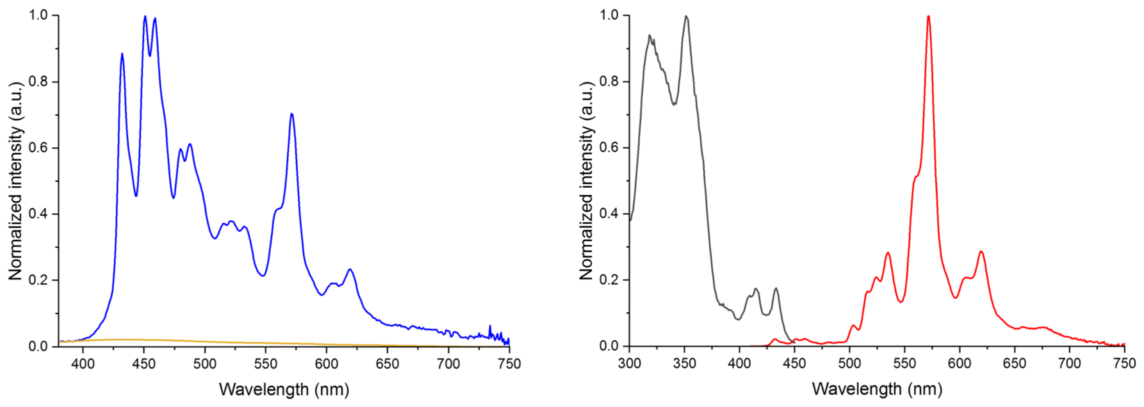

Figure S39. (Left) normalized steady-state luminescence spectrum (blue line) and normalized background spectrum (host without guest, orange line) of 0.1 wt% coronene (**3**) in 4-CHO DBI (**2q**). Excitation at 350 nm. (Right) phosphorescence excitation spectrum (emission at 571 nm, black line) and phosphorescence emission spectrum (red line, excitation at 350 nm) of the same host/guest system. The total luminescence quantum yield was determined to be φ = 0.036. The fluorescence quantum yield was determined to be φ_{FL} = 0.026 and the phosphorescence quantum yield φ_{Phos} = 0.010.

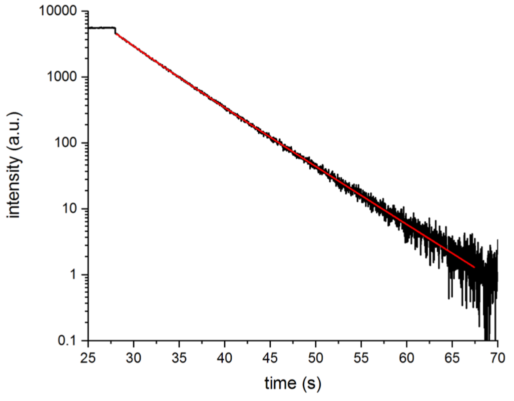

Figure S40. Phosphorescence lifetime decay curve (black line) and fitted lifetime decay (red line) of coronene (**3**) in 4-CHO DBI (**2q**) (0.1 wt%). Excitation at 350 nm, emission at 571 nm. The lifetime was determined to be: τ_1 = 3.08 ± 0.11 s (21 %); τ_2 = 4.97 ± 0.02 s (79 %); τ_{avg} = 4.57 ± 0.10 s.

3.2.18 4-CN DBI (2r)

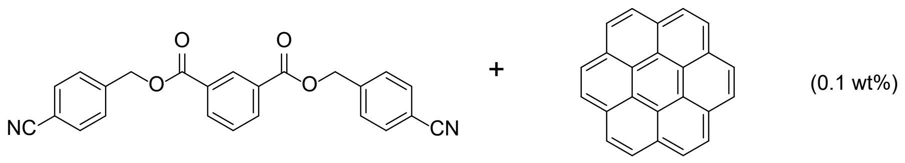

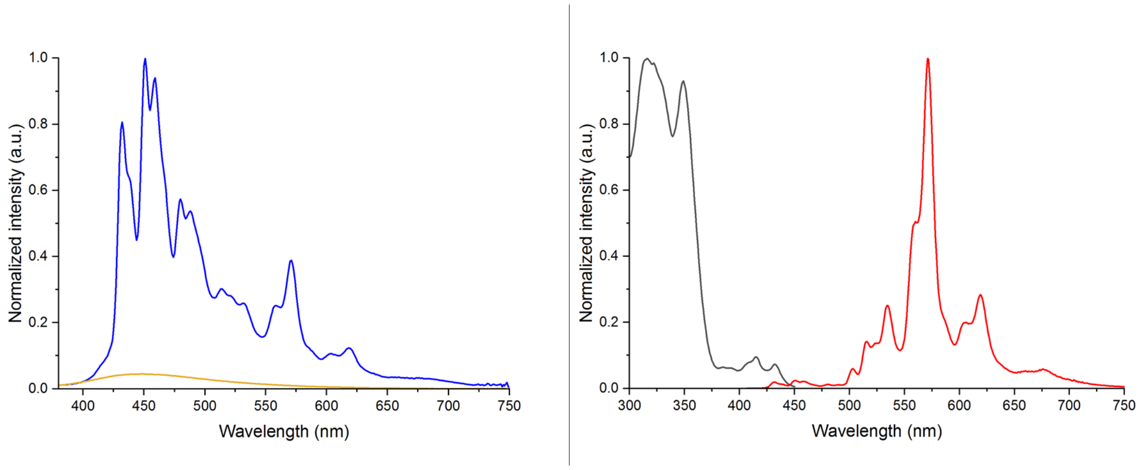

Figure S41. (Left) normalized steady-state luminescence spectrum (blue line) and normalized background spectrum (host without guest, orange line) of 0.1 wt% coronene (**3**) in 4-CN DBI (**2r**). Excitation at 350 nm. (Right) phosphorescence excitation spectrum (emission at 571 nm, black line) and phosphorescence emission spectrum (red line, excitation at 350 nm) of the same host/guest system. The total luminescence quantum yield was determined to be φ = 0.160. The fluorescence quantum yield was determined to be φ_{FL} = 0.132 and the phosphorescence quantum yield φ_{Phos} = 0.028.

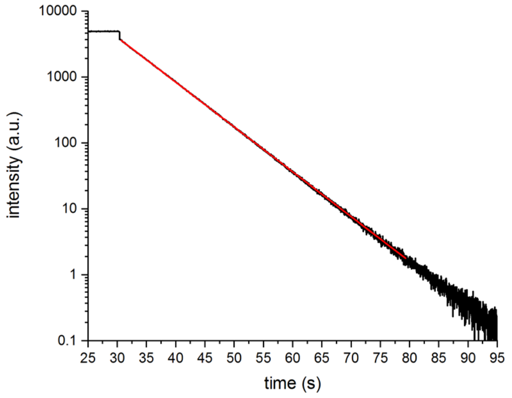

Figure S42. Phosphorescence lifetime decay curve (black line) and fitted lifetime decay (red line) of coronene (**3**) in 4-CN DBI (**2r**) (0.1 wt%). Excitation at 350 nm, emission at 571 nm. The lifetime was determined to be: τ = 6.39 ± 0.01 s.

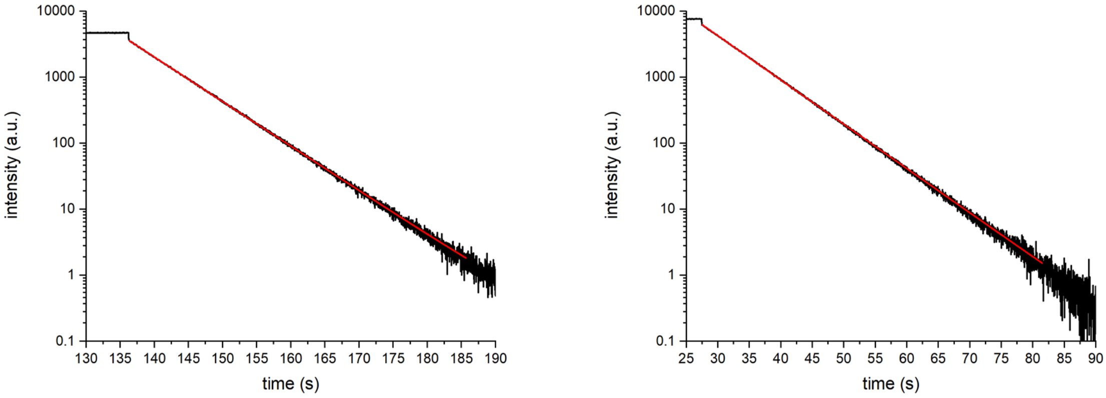

Figure S43. Phosphorescence lifetime decay curves (black lines) and fitted lifetime decays (red lines) of coronene (**3**) in 4-CN DBI (**2r**) (0.1 wt%) after two years storage at room temperature in the dark. Measured under air (left) and under argon atmosphere (right). Excitation at 350 nm, emission at 571 nm. The lifetimes were determined to be: τ = 6.44 ± 0.01 s (air atmosphere), and τ = 6.48 ± 0.01 s (argon atmosphere).

3.2.19 4-CO_2Me DBI (2s)

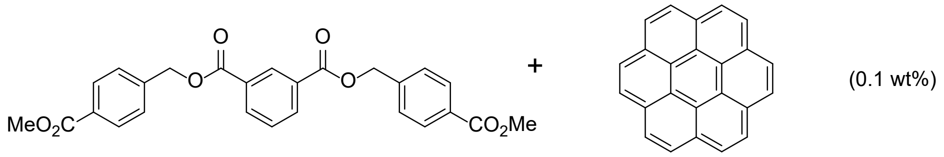

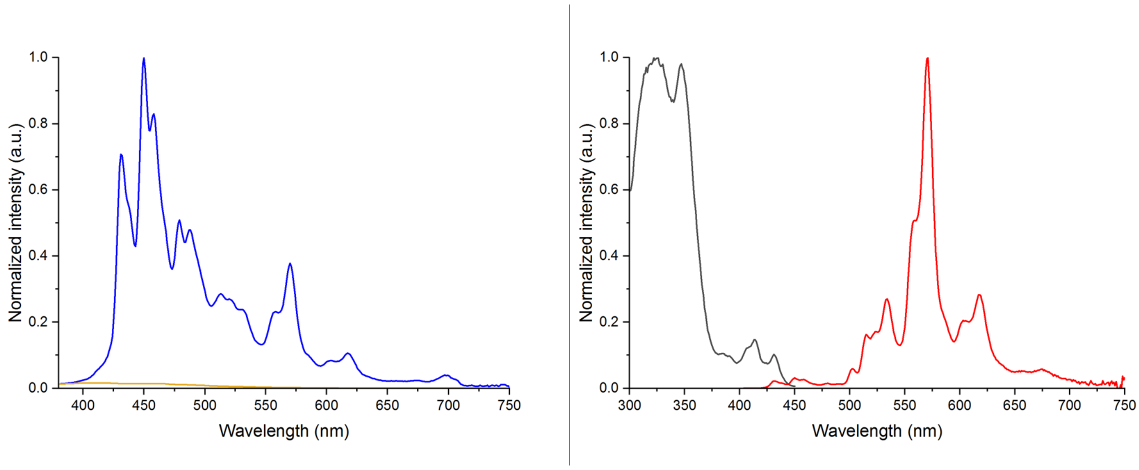

Figure S44. (Left) normalized steady-state luminescence spectrum (blue line) and normalized background spectrum (host without guest, orange line) of 0.1 wt% coronene (**3**) in 4-CO_2Me DBI (**2s**). Excitation at 350 nm. (Right) phosphorescence excitation spectrum (emission at 571 nm, black line) and phosphorescence emission spectrum (red line, excitation at 350 nm) of the same host/guest system. The total luminescence quantum yield was determined to be φ =

0.238. The fluorescence quantum yield was determined to be φ_{FL} = 0.190 and the phosphorescence quantum yield φ_{Phos} = 0.048.

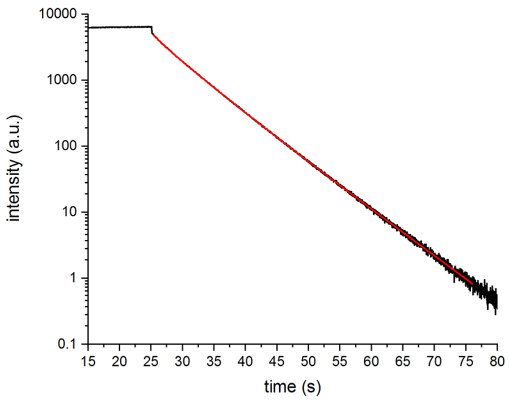

Figure S45. Phosphorescence lifetime decay curve (black line) and fitted lifetime decay (red line) of coronene (**3**) in 4-CO_2Me DBI (**2s**) (0.1 wt%). Excitation at 350 nm, emission at 571 nm. The lifetime was determined to be: τ_1 = 1.23 ± 0.04 s (7 %); τ_2 = 4.30 ± 0.07 s (36 %); τ_3 = 6.24 ± 0.02 s (57 %); τ_{avg} = 5.17 ± 0.06 s

3.2.20 4-CF_3 DBI (2t)

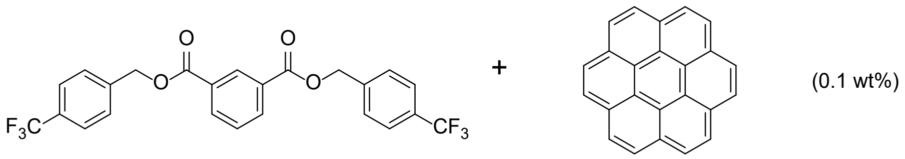

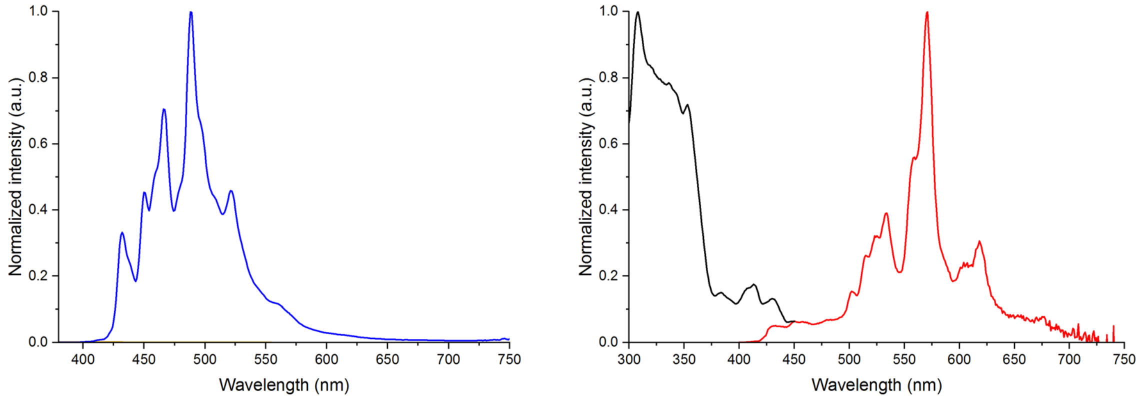

Figure S46. (Left) normalized steady-state luminescence spectrum (blue line) and normalized background spectrum (host without guest, orange, below visibility) of 0.1 wt% coronene (**3**) in 4-CF_3 DBI (**2t**). Excitation at 350 nm. (Right) phosphorescence excitation spectrum (emission at 571 nm, black line) and phosphorescence emission spectrum (red line, excitation at 350

nm) of the same host/guest system. The total luminescence quantum yield was determined to be φ = 0.423. The fluorescence quantum yield was determined to be φ_{FL} = 0.426 and the phosphorescence quantum yield φ_{Phos} ≈ 0.

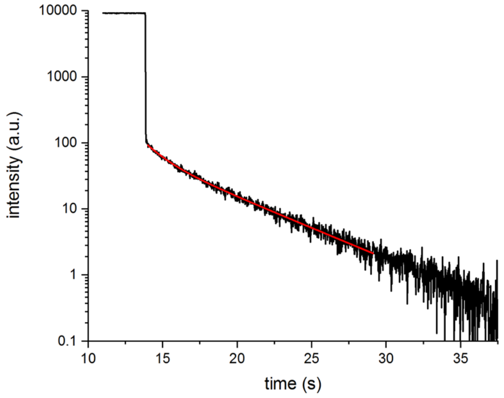

Figure S47. Phosphorescence lifetime decay curve (black line) and fitted lifetime decay (red line) of coronene (**3**) in 4-CF_3 DBI (**2t**) (0.1 wt%). Excitation at 350 nm, emission at 571 nm. The lifetime was determined to be: τ_1 = 1.22 ± 0.07 s (40 %); τ_2 = 4.63 ± 0.05 s (60 %); τ_{avg} = 3.27 ± 0.06 s.

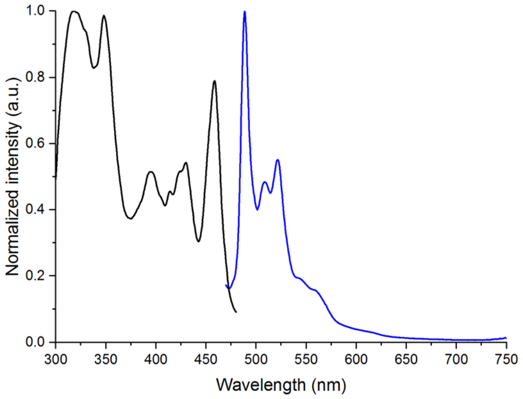

Figure S48. Normalized steady-state luminescence spectrum (blue line) and steady-state luminescence excitation spectrum (black line, emission at 490 nm) of 0.1 wt% coronene (**3**) in 4-CF_3 DBI (**2t**). Excitation at 460 nm.

3.2.21 4-B(OH)₂ DBI (2u)

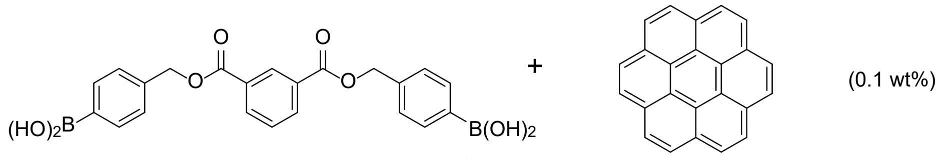

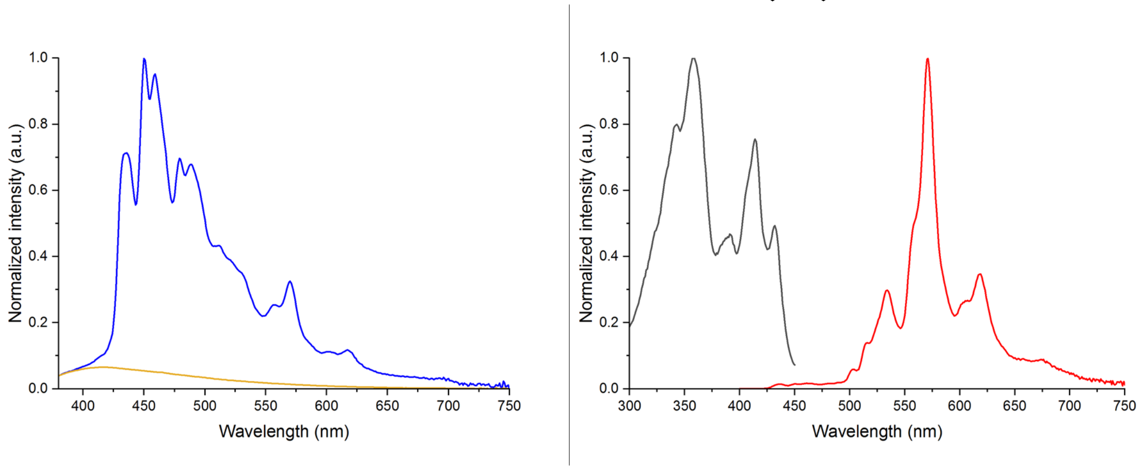

Figure S49. (Left) normalized steady-state luminescence spectrum (blue line) and normalized background spectrum (host without guest, orange line) of 0.1 wt% coronene (**3**) in 4-B(OH)$_2$ DBI (**2u**). Excitation at 350 nm. (Right) phosphorescence excitation spectrum (emission at 571 nm, black line) and phosphorescence emission spectrum (red line, excitation at 350 nm) of the same host/guest system. The total luminescence quantum yield was determined to be φ = 0.125. The fluorescence quantum yield was determined to be $φ_{FL}$ = 0.115 and the phosphorescence quantum yield $φ_{Phos}$ = 0.010.

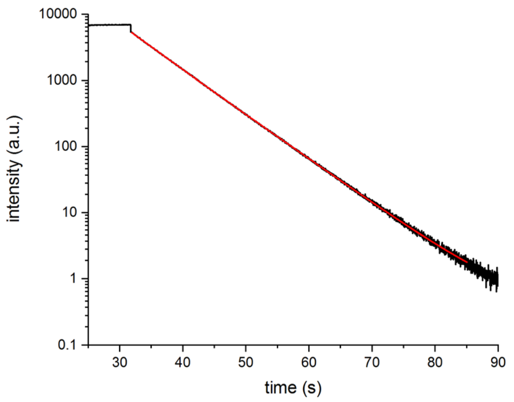

Figure S50. Phosphorescence lifetime decay curve (black line) and fitted lifetime decay (red line) of coronene (**3**) in 4-B(OH)$_2$ DBI (**2u**) (0.1 wt%). Excitation at 350 nm, emission at 571 nm. The lifetime was determined to be: $τ_1$ = 4.10 ± 0.14 s (9 %); $τ_2$ = 6.53 ± 0.01 s (91 %); $τ_{avg}$ = 6.32 ± 0.07 s.

3.2.22 Bis(4-bromobenzyl) pyridine-3,5-dicarboxylate (S1)

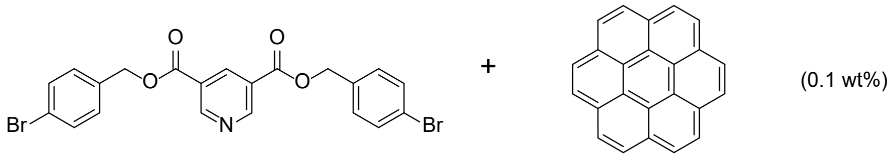

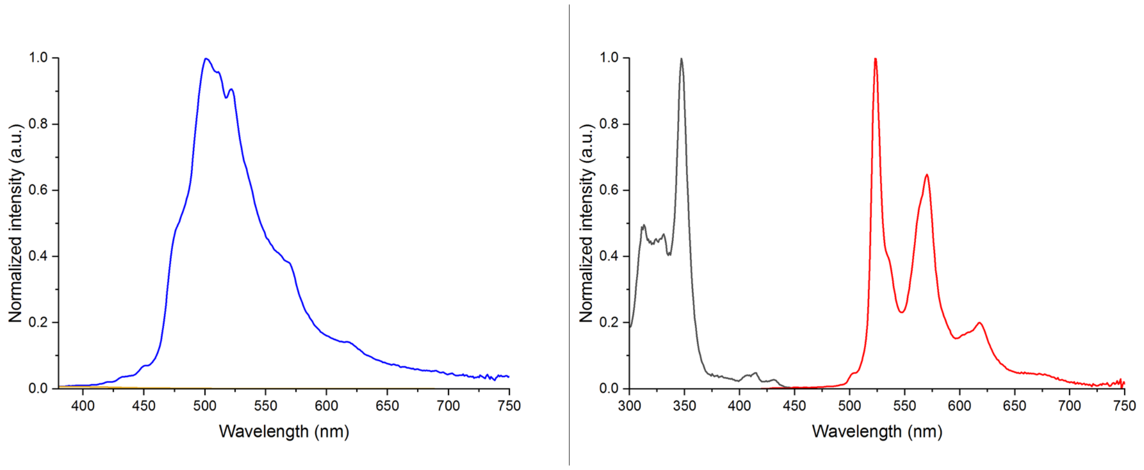

Figure S51. (Left) normalized steady-state luminescence spectrum (blue line) and normalized background spectrum (host without guest, orange line) of 0.1 wt% coronene (**3**) in bis(4-bromobenzyl) pyridine-3,5-dicarboxylate (**S1**). Excitation at 350 nm. (Right) phosphorescence excitation spectrum (emission at 571 nm, black line) and phosphorescence emission spectrum (red line, excitation at 350 nm) of the same host/guest system. The total luminescence quantum yield was determined to be φ = 0.143. The fluorescence quantum yield was determined to be φ_{FL} = 0.125 and the phosphorescence quantum yield φ_{Phos} = 0.018.

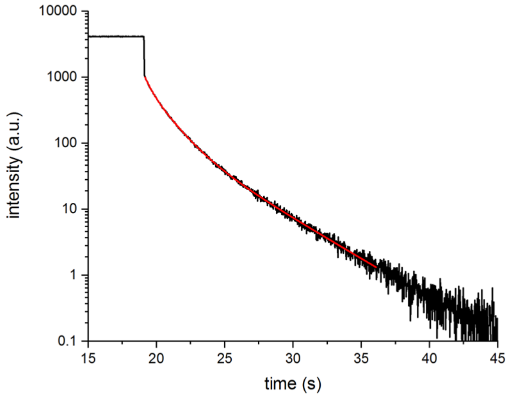

Figure S52. Phosphorescence lifetime decay curve (black line) and fitted lifetime decay (red line) of coronene (**3**) in bis(4-bromobenzyl) pyridine-3,5-dicarboxylate (**S1**) (0.1 wt%). Excitation at 350 nm, emission at 571 nm. The lifetime was determined to be: τ_1 = 0.43 ± 0.02 s (26 %); τ_2 = 1.36 ± 0.02 s (59 %); τ_3 = 3.57 ± 0.03 s (15 %); τ_{avg} = 1.46 ± 0.02 s.

3.2.23 N^1,N^3-bis(4-bromobenzyl)isophthalamideate (S2)

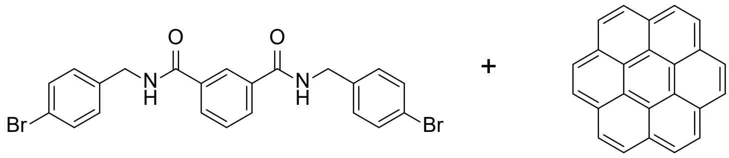

(0.1 wt%)

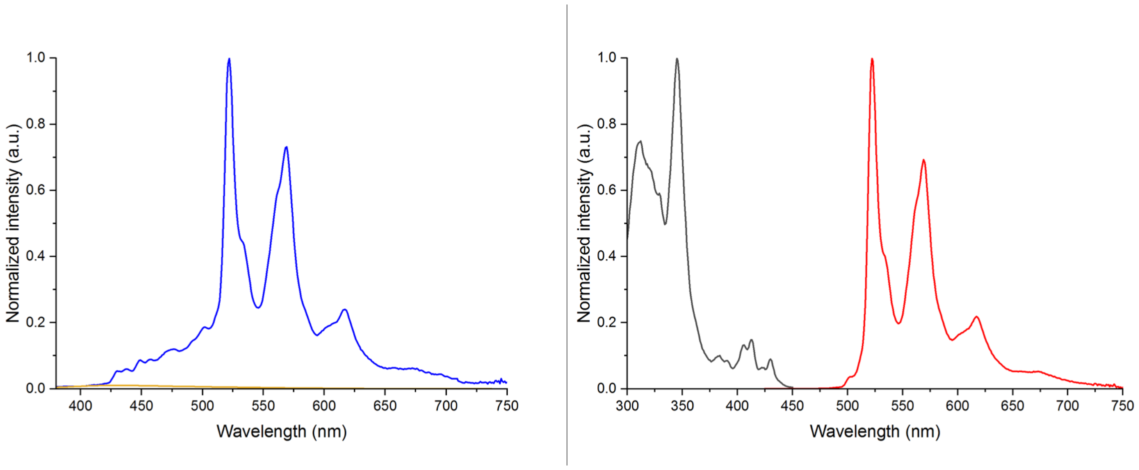

Figure S53. (Left) normalized steady-state luminescence spectrum (blue line) and normalized background spectrum (host without guest, orange line) of 0.1 wt% coronene (**3**) in N^1,N^3-bis(4-bromobenzyl)isophthalamideate (**S2**). Excitation at 350 nm. (Right) phosphorescence excitation spectrum (emission at 571 nm, black line) and phosphorescence emission spectrum (red line, excitation at 350 nm) of the same host/guest system. The total luminescence quantum yield was determined to be φ = 0.116. The fluorescence quantum yield was determined to be φ_{FL} = 0.037 and the phosphorescence quantum yield φ_{Phos} = 0.079.

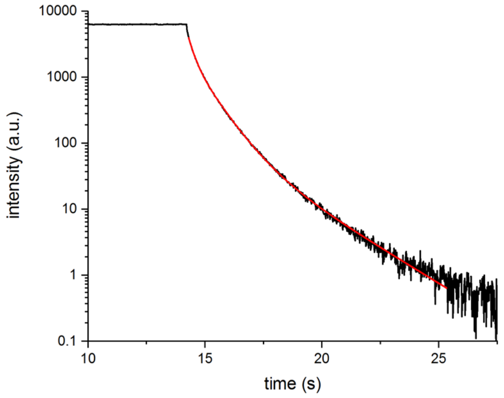

Figure S54. Phosphorescence lifetime decay curve (black line) and fitted lifetime decay (red line) of coronene (**3**) in N^1,N^3-bis(4-bromobenzyl)isophthalamideate (**S2**) (0.1 wt%). Excitation at 350 nm, emission at 571 nm. The lifetime was determined to be: τ_1 = 0.23 ± 0.01 s (51 %); τ_2 = 0.77 ± 0.01 s (46 %); τ_3 = 2.01 ± 0.03 s (4 %); τ_{avg} = 0.55 ± 0.01 s.

3.3 Substituted dibenzyl isophthalates hosts with 0.1 wt% coronene-d_{12}

3.3.1 4-Cl DBI (2b)

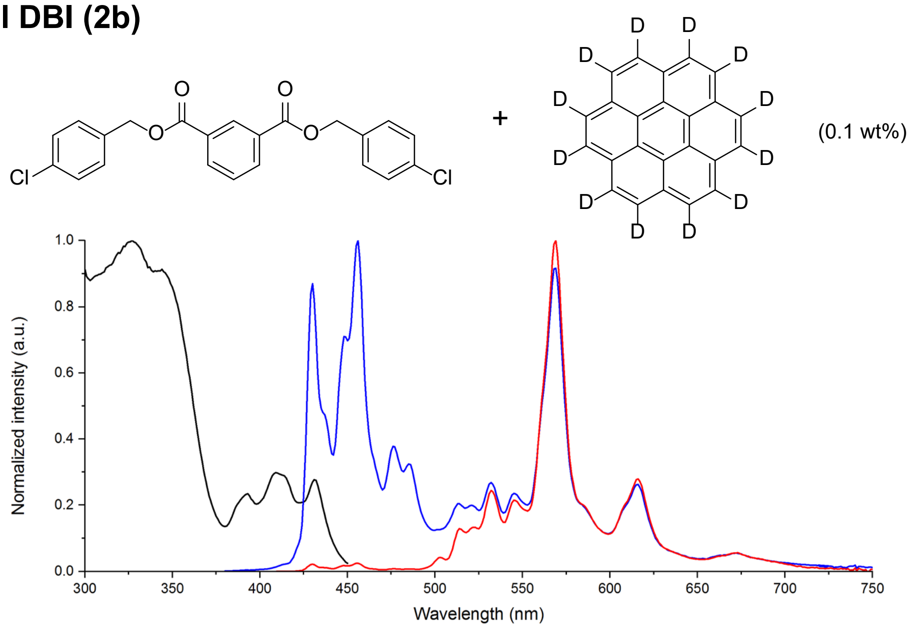

Figure S55. Luminescence of 0.1 wt% of coronene-d_{12} in 4-Cl DBI (**2b**). Normalized phosphorescence excitation spectrum (black line; emission at 571 nm), steady-state luminescence emission spectrum (blue line) and phosphorescence spectrum (red line; excitation for both at 350 nm). The total luminescence quantum yield was determined to be φ = 0.348. The fluorescence quantum yield was determined to be φ_{FL} = 0.191 and the phosphorescence quantum yield φ_{Phos} = 0.157.

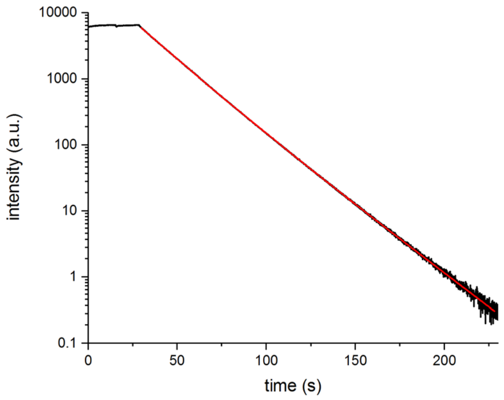

Figure S56. Phosphorescence lifetime decay curve (black line) and fitted lifetime decay (red line) of coronene-d_{12} in 4-Cl DBI (**2b**) (0.1 wt%). Excitation at 350 nm, emission at 571 nm. The lifetime was determined to be: τ_1 = 16.3 ± 0.1 s (52 %); τ_2 = 21.6 ± 0.1 s (48 %); τ_{avg} = 18.8 ± 0.1 s.

3.3.2 4-Br DBI (2e)

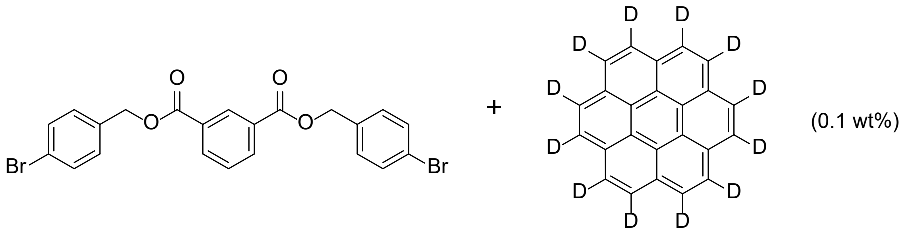

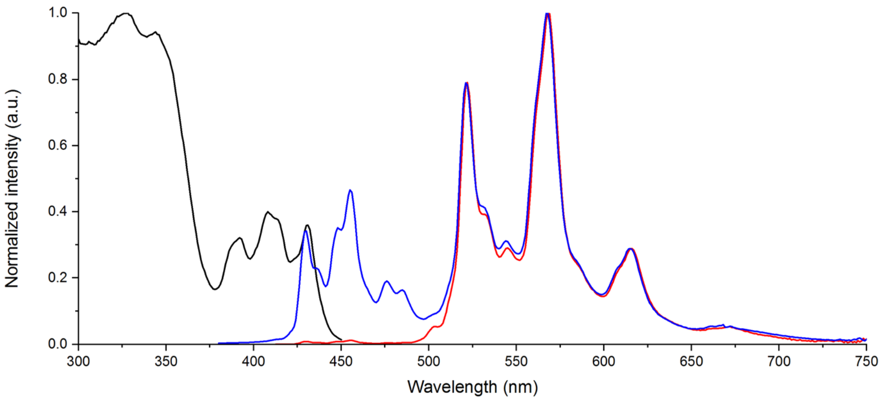

Figure S57. Luminescence of 0.1 wt% of coronene-d_{12} in 4-Br DBI (**2e**). Normalized phosphorescence excitation spectrum (black line; emission at 571 nm), steady-state luminescence emission spectrum (blue line) and phosphorescence spectrum (red line; excitation for both at 350 nm). The total luminescence quantum yield was determined to be φ = 0.332. The fluorescence quantum yield was determined to be φ_{FL} = 0.073 and the phosphorescence quantum yield φ_{Phos} = 0.259.

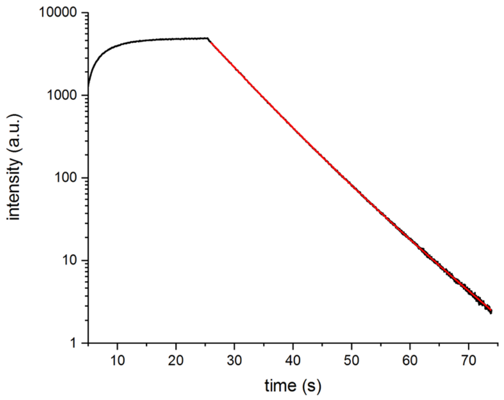

Figure S58. Phosphorescence lifetime decay curve (black line) and fitted lifetime decay (red line) of coronene-d_{12} in 4-Br DBI (**2e**) (0.1 wt%). Excitation at 350 nm, emission at 571 nm. The lifetime was determined to be: τ_1 = 5.43 ± 0.01 s (82 %); τ_2 = 8.01 ± 0.05 s (18 %); τ_{avg} = 5.90 ± 0.02 s.

3.3.3 4-I DBI (2g)

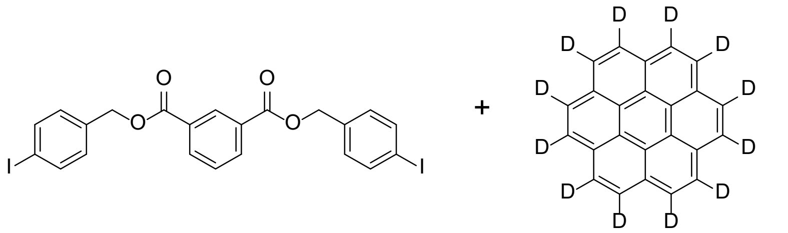

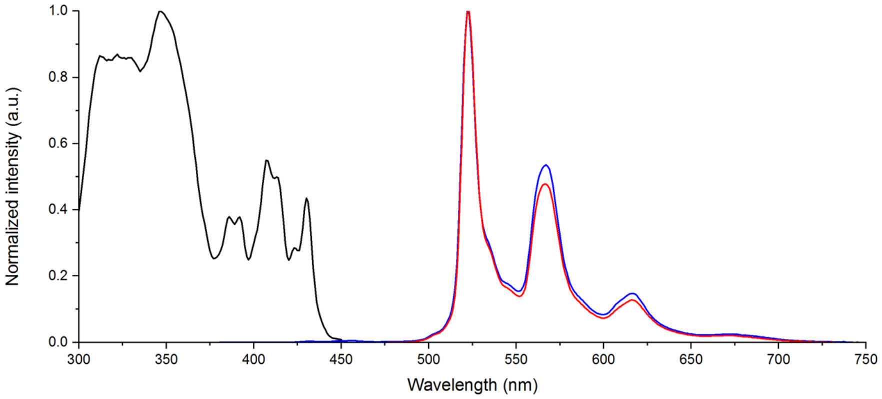

Figure S59. Luminescence of 0.1 wt% of coronene-d_{12} in 4-I DBI (**2g**). Normalized phosphorescence excitation spectrum (black line; emission at 571 nm), steady-state luminescence emission spectrum (blue line) and phosphorescence spectrum (red line; excitation for both at 350 nm). The total luminescence quantum yield was determined to be $\varphi = 0.772$. The fluorescence quantum yield was determined to be $\varphi_{FL} = 0.005$ and the phosphorescence quantum yield $\varphi_{Phos} = 0.767$.

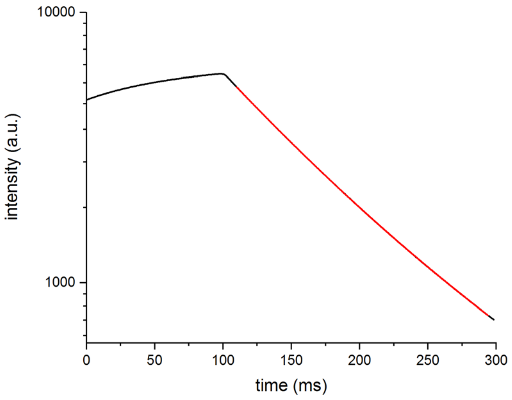

Figure S60. Phosphorescence lifetime decay curve (black line) and fitted lifetime decay (red line) of coronene-d_{12} in 4-I DBI (**2g**) (0.1 wt%). Excitation at 350 nm, emission at 571 nm. The lifetime was determined to be: τ_1 = 68.2 ± 0.1 ms (71 %); τ_2 = 164 ± 1 ms (29 %); τ_{avg} = 96.3 ± 0.3 ms.

3.3.4 4-OMe DBI (2h)

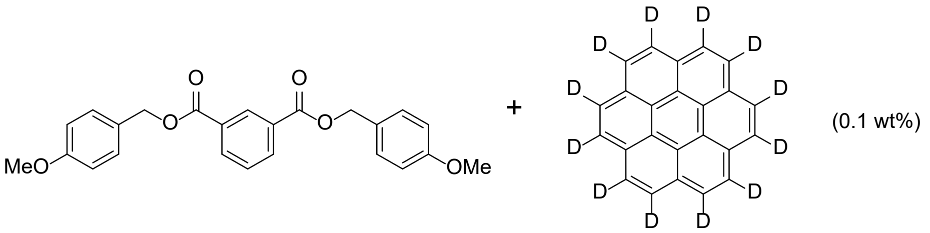

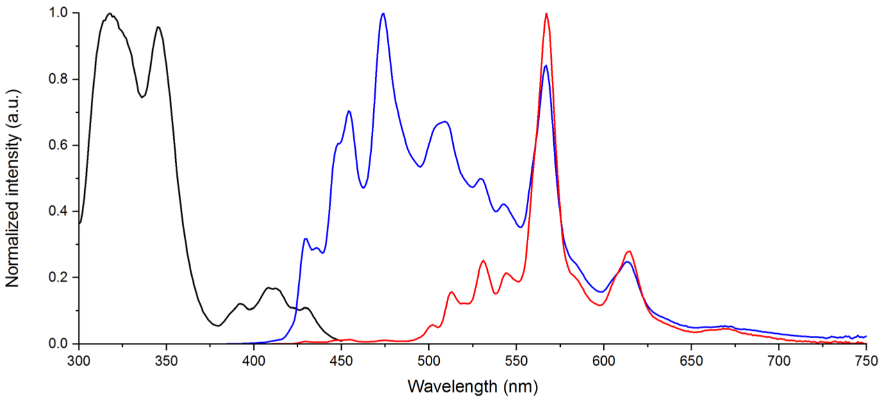

Figure S61. Luminescence of 0.1 wt% of coronene-d_{12} in 4-OMe DBI (**2h**). Normalized phosphorescence excitation spectrum (black line; emission at 571 nm), steady-state luminescence emission spectrum (blue line) and phosphorescence spectrum (red line; excitation for both at 350 nm). The total luminescence quantum yield was determined to be φ = 0.345. The fluorescence quantum yield was determined to be φ_{FL} = 0.259 and the phosphorescence quantum yield φ_{Phos} = 0.086.

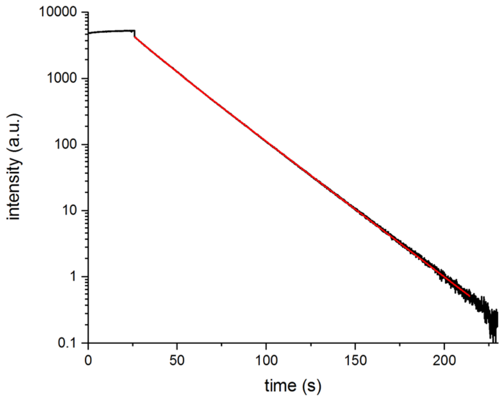

Figure S62. Phosphorescence lifetime decay curve (black line) and fitted lifetime decay (red line) of coronene-d_{12} in 4-OMe DBI (**2h**) (0.1 wt%). Excitation at 350 nm, emission at 571 nm. The lifetime was determined to be: τ_1 = 11.9 ± 0.1 s (15 %); τ_2 = 21.2 ± 0.1 s (85 %); τ_{avg} = 19.8 ± 0.1 s.

3.3.5 4-SPh DBI (2n)

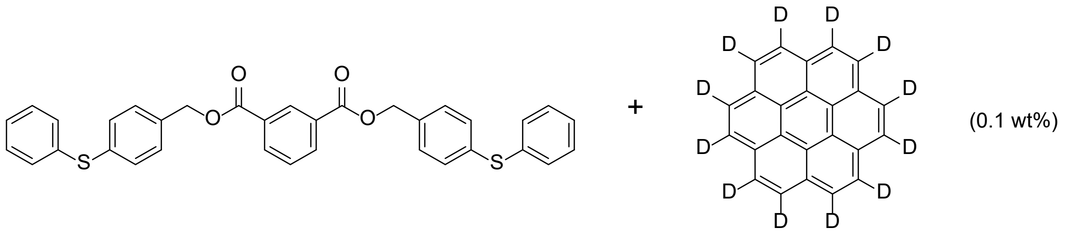

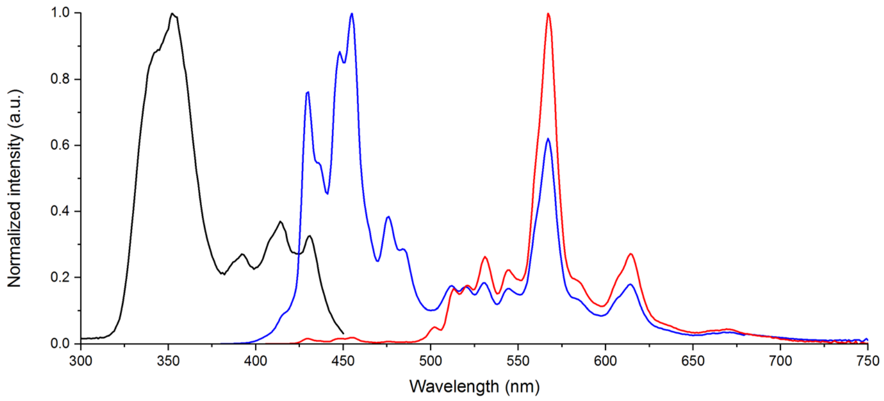

Figure S63. Luminescence of 0.1 wt% of coronene-d_{12} in 4-SPh DBI (**2n**). Normalized phosphorescence excitation spectrum (black line; emission at 571 nm), steady-state luminescence emission spectrum (blue line) and phosphorescence spectrum (red line; excitation for both at 350 nm). The total luminescence quantum yield was determined to be φ = 0.312. The fluorescence quantum yield was determined to be φ_{FL} = 0.218 and the phosphorescence quantum yield φ_{Phos} = 0.094.

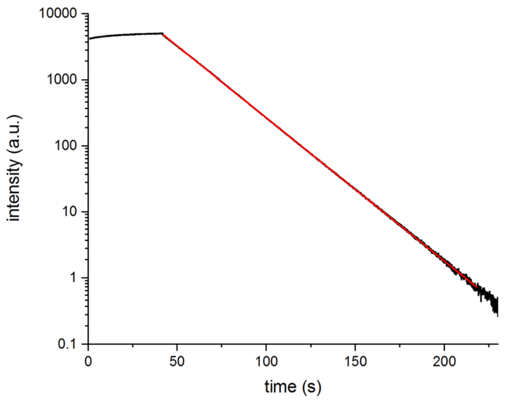

Figure S64. Phosphorescence lifetime decay curve (black line) and fitted lifetime decay (red line) of coronene-d_{12} in 4-SPh DBI (**2n**) (0.1 wt%). Excitation at 350 nm, emission at 571 nm. The lifetime was determined to be: τ = 20.0 ± 0.1 s.

3.3.6 4-CN DBI (2r)

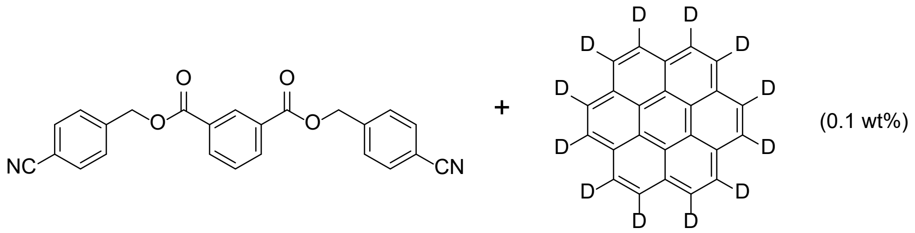

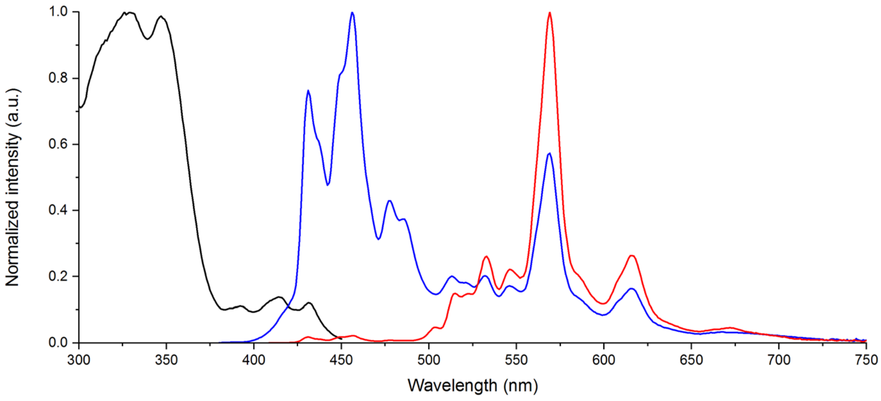

Figure S65. Luminescence of 0.1 wt% of coronene-d_{12} in 4-CN DBI (**2r**). Normalized phosphorescence excitation spectrum (black line; emission at 571 nm), steady-state luminescence emission spectrum (blue line) and phosphorescence spectrum (red line; excitation for both at 350 nm). The total luminescence quantum yield was determined to be φ = 0.208. The fluorescence quantum yield was determined to be φ_{FL} = 0.135 and the phosphorescence quantum yield φ_{Phos} = 0.073.

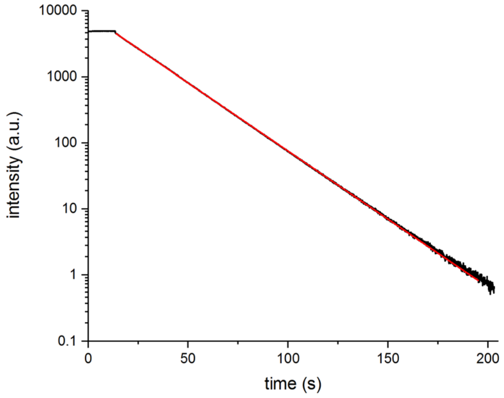

Figure S66. Phosphorescence lifetime decay curve (black line) and fitted lifetime decay (red line) of coronene-d_{12} in 4-CN DBI (**2r**) (0.1 wt%). Excitation at 350 nm, emission at 571 nm. The lifetime was determined to be: τ = 21.0 ± 0.1 s.

3.3.7 4-B(OH)2 DBI (2u)

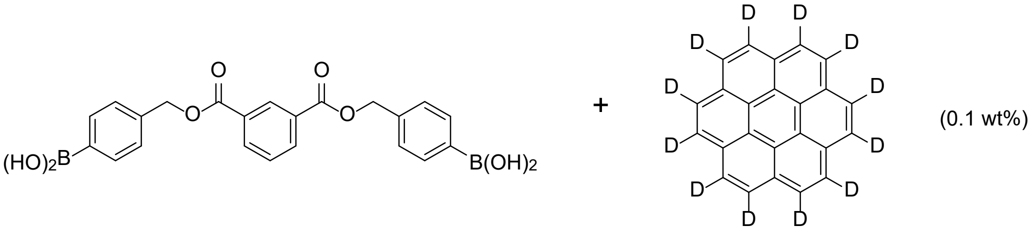

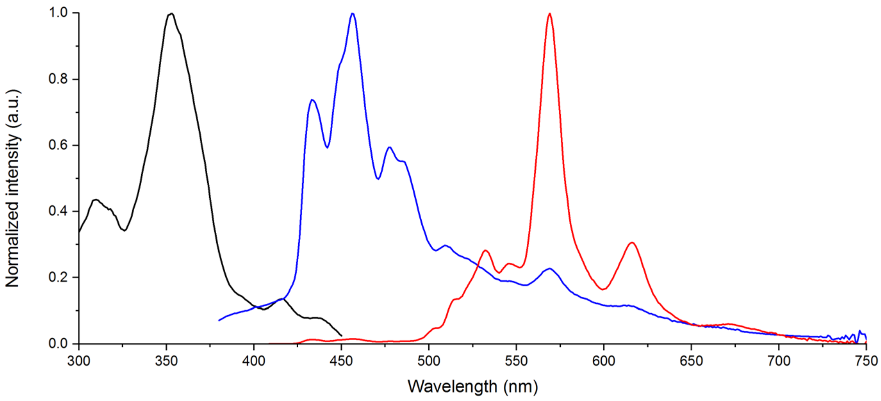

Figure S67. Luminescence of 0.1 wt% of coronene-d_{12} in 4-B(OH)$_2$ DBI (**2u**). Normalized phosphorescence excitation spectrum (black line; emission at 571 nm), steady-state luminescence emission spectrum (blue line) and phosphorescence spectrum (red line; excitation for both at 350 nm). The total luminescence quantum yield was determined to be φ = 0.195. The fluorescence quantum yield was determined to be φ_{FL} = 0.175 and the phosphorescence quantum yield φ_{Phos} = 0.020.

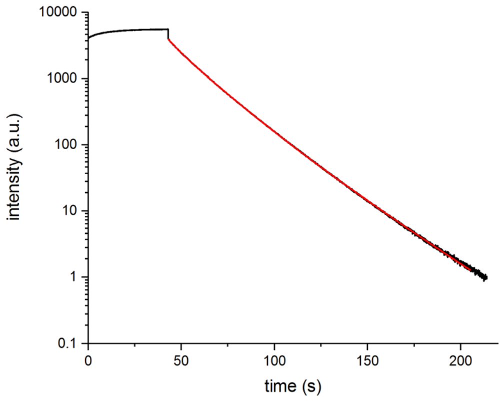

Figure S68. Phosphorescence lifetime decay curve (black line) and fitted lifetime decay (red line) of coronene-d_{12} in 4-B(OH)$_2$ DBI (**2u**) (0.1 wt%). Excitation at 350 nm, emission at 571 nm. The lifetime was determined to be: τ_1 = 5.16 ± 0.06 s (12 %); τ_2 = 15.9 ± 0.1 s (65 %); τ_3 = 24.4 ± 0.1 s (23 %); τ_{avg} = 16.6 ± 0.2 s.

3.4 Effect of different coronene concentrations in the 4-Br DBI (2e) on the luminescence emission

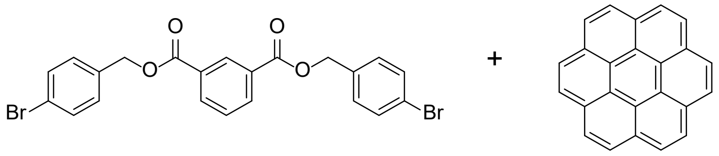

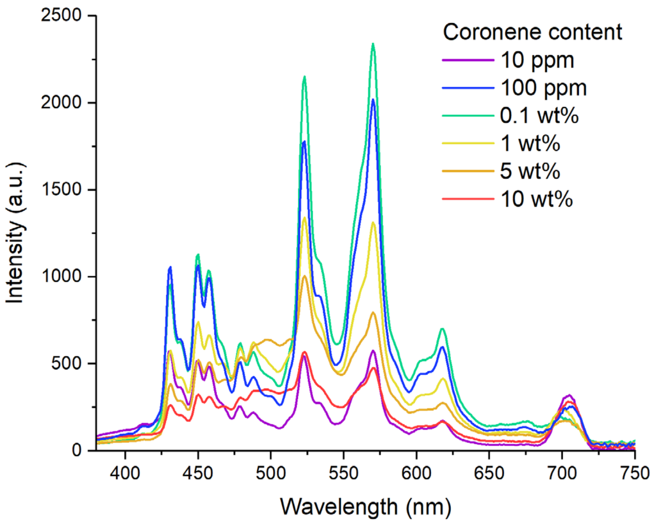

Figure S69. Comparison of different concentrations of coronene (**3**) in the 4-Br DBI (**2e**). Steady state luminescence spectra (excitation at 345 nm). The 2nd order diffraction maxima at 690 nm are present. At concentrations of 5 wt% or higher of coronene (**3**), inhomogeneous mixtures formed.

4 Photophysical properties of guests in solution and in host/guest-systems with DBI hosts

4.1 Photophysical properties of 0.1 wt% guests in the 4-Br DBI (2e) host

4.1.1 4-(Methylthio)benzaldehyde (G1)

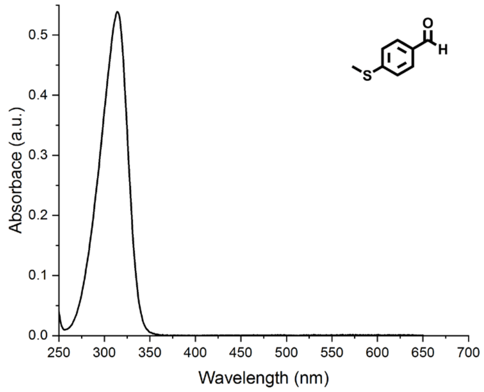

Figure S70. UV-absorption spectrum of 4-(methylthio)benzaldehyde (**G1**) in DCM. We did not observe fluorescence by this compound in solution.

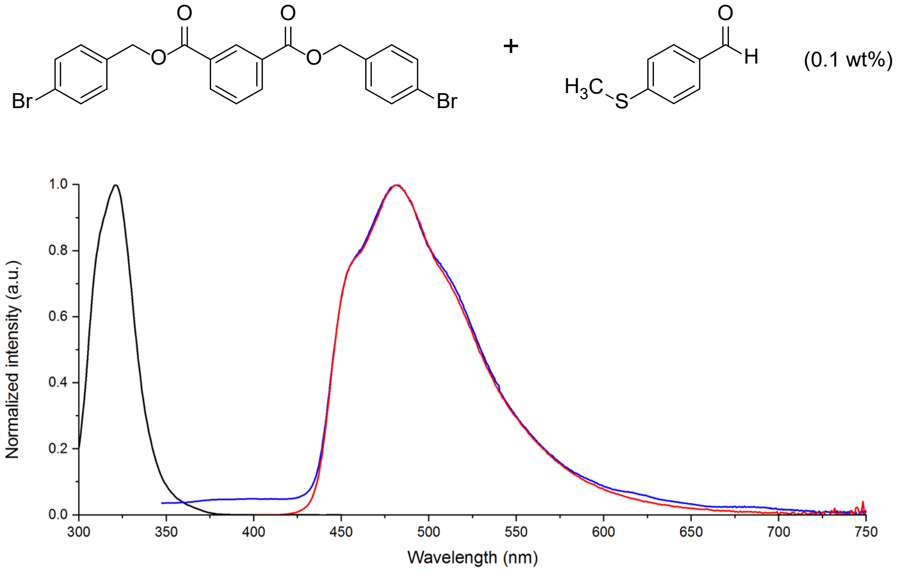

Figure S71. Luminescence of 0.1 wt% of 4-(methylthio)benzaldehyde (**G1**) in 4-Br DBI (**2e**). Normalized phosphorescence excitation spectrum (black line; emission at 481 nm), steady-state luminescence emission spectrum (blue line) and phosphorescence spectrum (red line; excitation for both at 322 nm). The total luminescence quantum yield was determined to be

φ = 0.028. The fluorescence quantum yield was too low to determine and the phosphorescence quantum yield φ_{Phos} ~ 0.028.

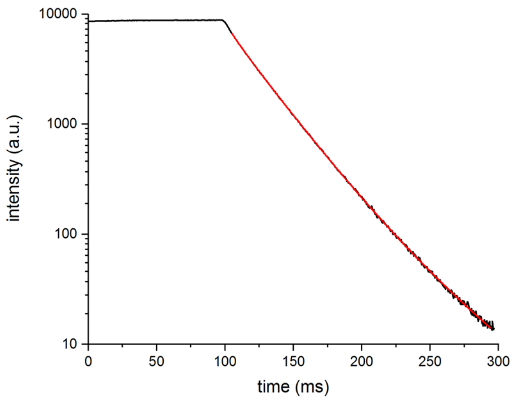

Figure S72. Phosphorescence lifetime decay curve (black line) and fitted lifetime decay (red line) of 4-(methylthio)benzaldehyde (**G1**) in 4-Br DBI (**2e**) (0.1 wt%). Excitation at 325 nm, emission at 481 nm. The lifetime was determined to be: τ_1 = 7.54 ± 0.61 ms (6 %); τ_2 = 25.9 ± 0.2 ms (87 %); τ_3 = 50.2 ± 1.9 ms (7 %); τ_{avg} = 26.3 ± 0.6 ms.

4.1.2 4-((4-Bromophenyl)thio)benzaldehyde (G2)

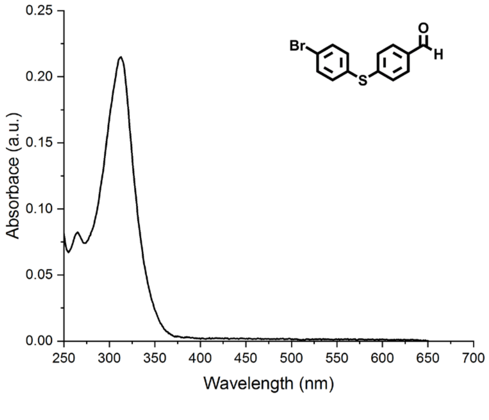

Figure S73. UV-absorption spectrum of 4-((4-bromophenyl)thio)benzaldehyde (**G2**) in DCM. We did not observe fluorescence by this compound in solution.

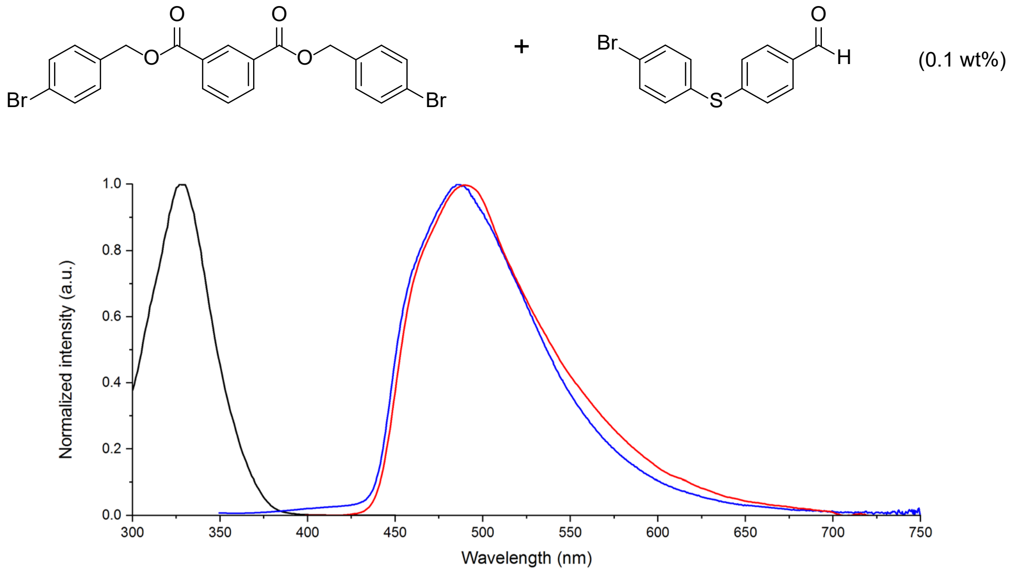

Figure S74. Luminescence of 0.1 wt% of 4-((4-bromophenyl)thio)benzaldehyde (**G2**) in 4-Br DBI (**2e**). Normalized phosphorescence excitation spectrum (black line; emission at 489 nm), steady-state luminescence emission spectrum (blue line) and phosphorescence spectrum (red line; excitation for both at 330 nm). The total luminescence quantum yield was determined to be φ = 0.098. The fluorescence quantum yield was too low to determine and the phosphorescence quantum yield φ_{Phos} = 0.098.

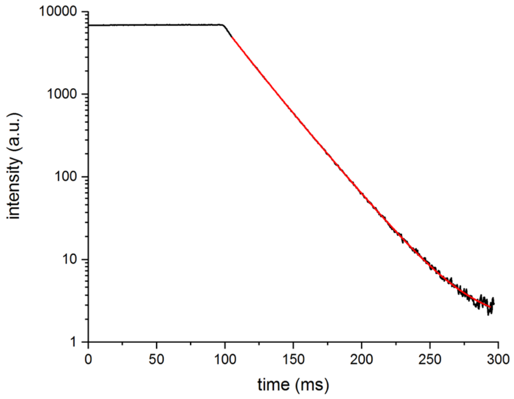

Figure S75. Phosphorescence lifetime decay curve (black line) and fitted lifetime decay (red line) of 4-((4-bromophenyl)thio)benzaldehyde (**G2**) in 4-Br DBI (**2e**) (0.1 wt%). Excitation at 325 nm, emission at 460 nm. The lifetime was determined to be: τ_1 = 14.9 ± 0.5 ms (22 %); τ_2 = 22.8 ± 0.1 ms (78 %); τ_{avg} = 21.1 ± 0.6 ms.

4.1.3 4-(Dimethylamino)benzaldehyde (G3)

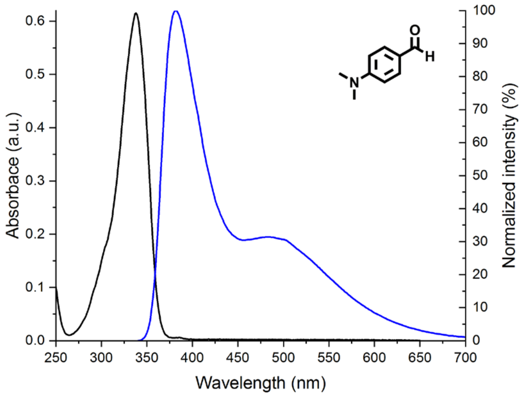

Figure S76. UV-absorption spectrum and normalized fluorescence emission spectrum of 4-(dimethylamino)benzaldehyde (**G3**) in DCM.

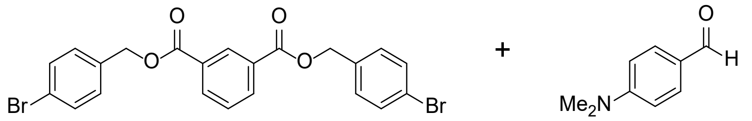

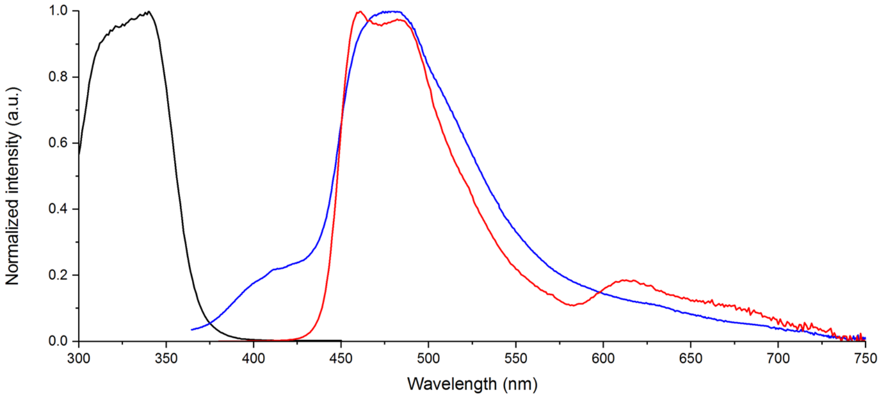

Figure S77. Luminescence of 0.1 wt% of 4-(dimethylamino)benzaldehyde (**G3**) in 4-Br DBI (**2e**). Normalized phosphorescence excitation spectrum (black line; emission at 480 nm), steady-state luminescence emission spectrum (blue line) and phosphorescence spectrum (red line; excitation for both at 340 nm). The total luminescence quantum yield was determined to be φ = 0.031. The fluorescence quantum yield was determined to be φ_{FL} = 0.018 and the phosphorescence quantum yield φ_{Phos} = 0.013.

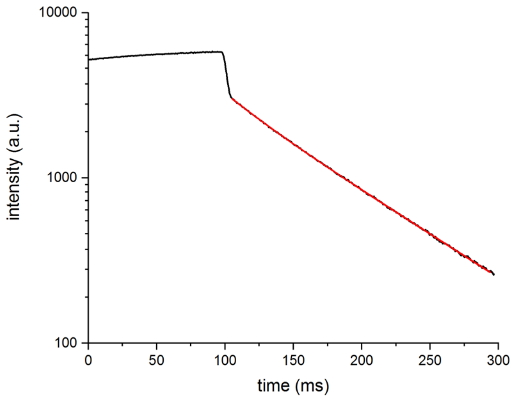

Figure S78. Phosphorescence lifetime decay curve (black line) and fitted lifetime decay (red line) of 4-(dimethylamino)benzaldehyde (**G3**) in 4-Br DBI (**2e**) (0.1 wt%). Excitation at 345 nm, emission at 458 nm. The lifetime was determined to be: τ_1 = 28.0 ± 1.0 ms (13 %); τ_2 = 82.7 ± 0.3 ms (87 %); τ_{avg} = 75.6 ± 0.5 ms.

4.1.4 1,8-Naphthalic anhydride (G4)

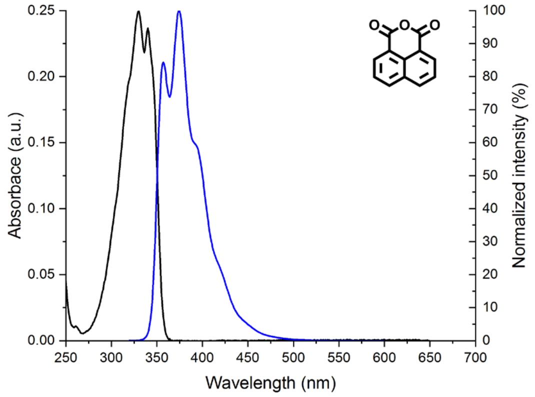

Figure S79. UV-absorption spectrum and normalized fluorescence emission spectrum of 1,8-naphthalic anhydride (**G4**) in DCM.

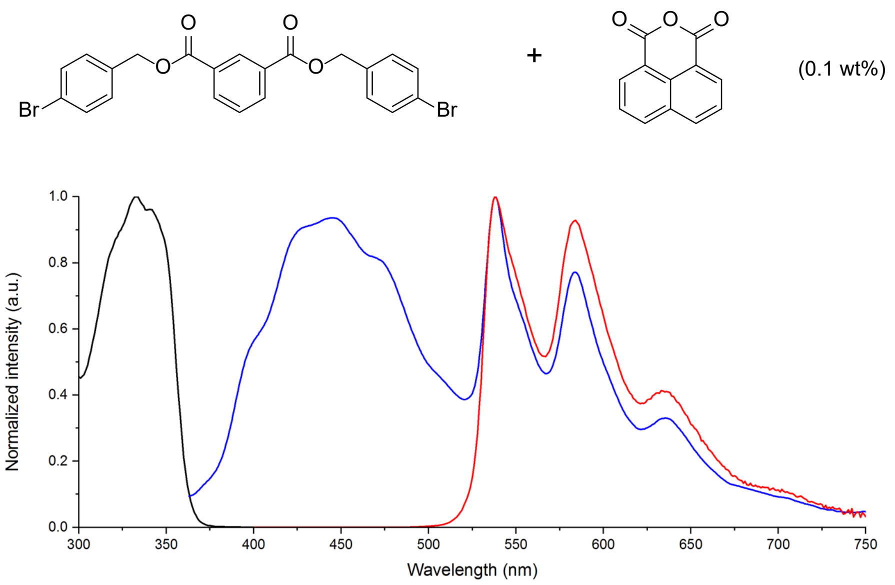

Figure S80. Luminescence of 0.1 wt% of 1,8-naphthalic anhydride (**G4**) in 4-Br DBI (**2e**). Normalized phosphorescence excitation spectrum (black line; emission at 584 nm), steady-state luminescence emission spectrum (blue line) and phosphorescence spectrum (red line; excitation for both at 340 nm). The total luminescence quantum yield was determined to be φ = 0.104. The fluorescence quantum yield was determined to be φ_{FL} = 0.069 and the phosphorescence quantum yield φ_{Phos} = 0.035.

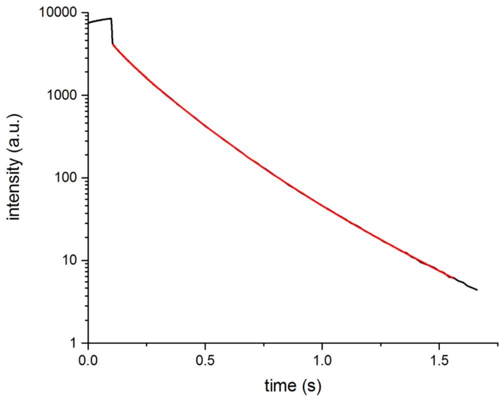

Figure S81. Phosphorescence lifetime decay curve (black line) and fitted lifetime decay (red line) of 1,8-naphthalic anhydride (**G4**) in 4-Br DBI (**2e**) (0.1 wt%). Excitation at 340 nm, emission at 584 nm. The lifetime was determined to be: τ_1 = 9.44 ± 0.51 ms (2 %); τ_2 = 58.6 ± 2.2 ms (16 %); τ_3 = 185 ± 1 ms (82 %); τ_{avg} = 161 ± 2 ms.

4.1.5 Dimethyl naphthalene-2,6-dicarboxylate (G5)

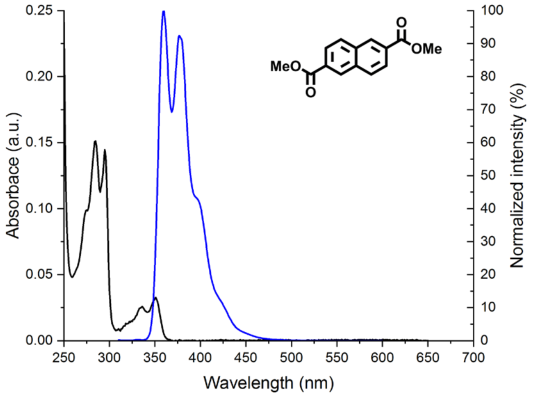

Figure S82. UV-absorption spectrum and normalized fluorescence emission spectrum of dimethyl naphthalene-2,6-dicarboxylate (**G5**) in DCM.

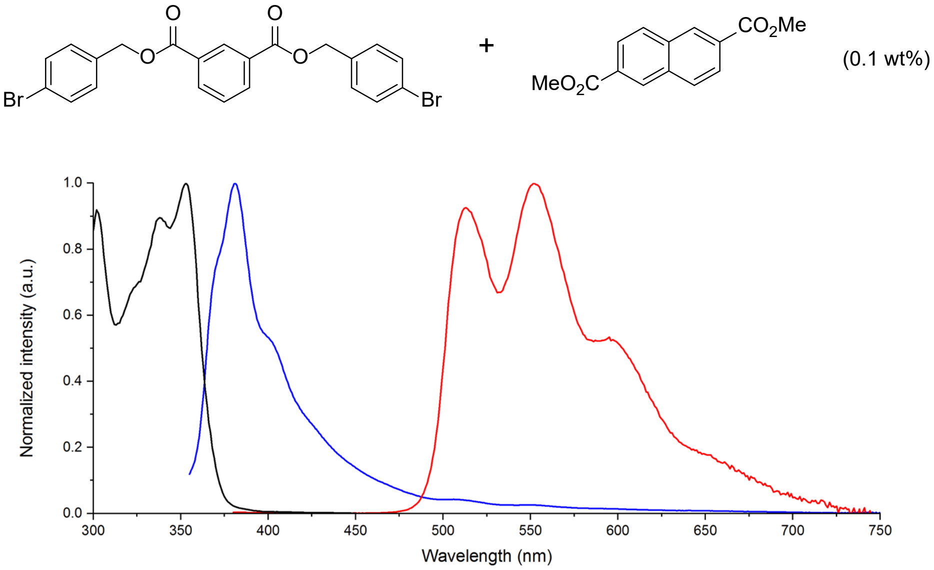

Figure S83. Luminescence of 0.1 wt% of dimethyl naphthalene-2,6-dicarboxylate (**G5**) in 4-Br DBI (**2e**). Normalized phosphorescence excitation spectrum (black line; emission at 555 nm), steady-state luminescence emission spectrum (blue line) and phosphorescence spectrum (red line; excitation for both at 352 nm). The total luminescence quantum yield was determined to be φ = 0.182. The fluorescence quantum yield was determined to be φ_{FL} = 0.177 and the phosphorescence quantum yield φ_{Phos} = 0.005.

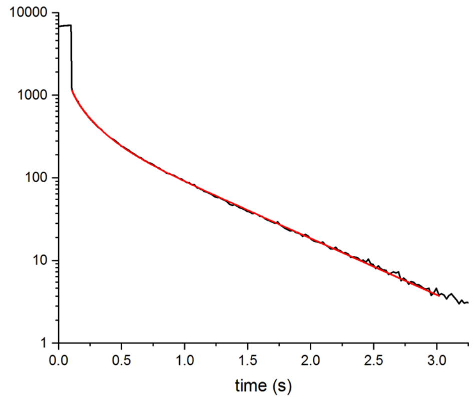

Figure S84. Phosphorescence lifetime decay curve (black line) and fitted lifetime decay (red line) of dimethyl naphthalene-2,6-dicarboxylate (**G5**) in 4-Br DBI (**2e**) (0.1 wt%). Excitation at 365 nm, emission at 513 nm. The lifetime was determined to be: τ_1 = 42.8 ± 1.2 ms (19 %); τ_2 = 158 ± 3 ms (49 %); τ_3 = 636 ± 2 ms (32 %); τ_{avg} = 289 ± 2 ms.

4.1.6 1-Naphthoic acid (G6)

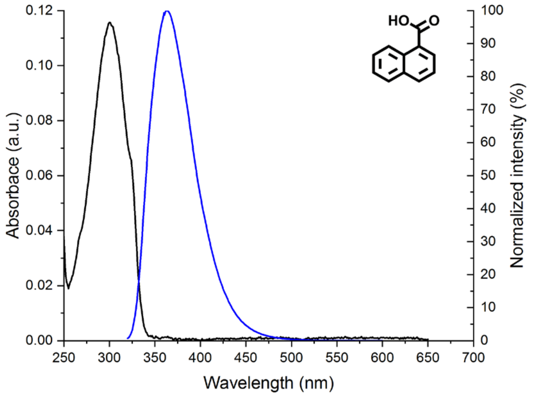

Figure S85. UV-absorption spectrum and normalized fluorescence emission spectrum of 1-naphthoic acid (**G6**) in DCM.

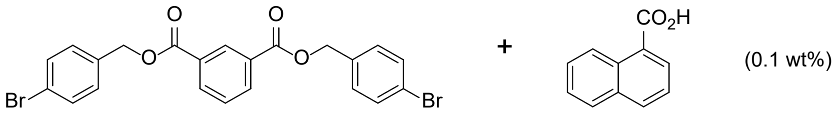

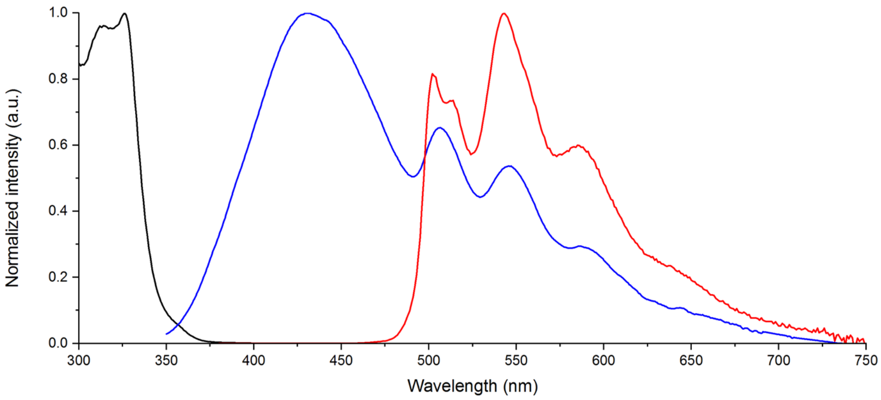

Figure S86. Luminescence of 0.1 wt% of 1-naphthoic acid (**G6**) in 4-Br DBI (**2e**). Normalized phosphorescence excitation spectrum (black line; emission at 543 nm), steady-state luminescence emission spectrum (blue line) and phosphorescence spectrum (red line; excitation for both at 326 nm). The total luminescence quantum yield was determined to be φ = 0.211. The fluorescence quantum yield was determined to be φ_{FL} = 0.157 and the phosphorescence quantum yield φ_{Phos} = 0.054.

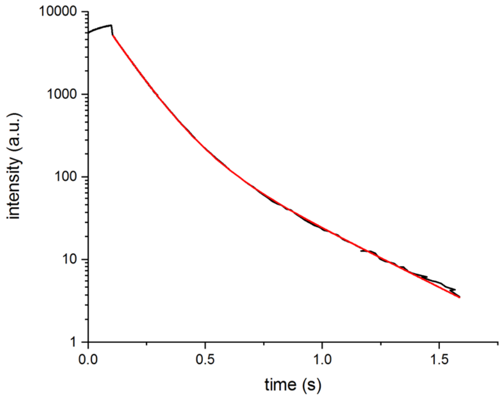

Figure S87. Phosphorescence lifetime decay curve (black line) and fitted lifetime decay (red line) of 1-naphthoic acid (**G6**) in 4-Br DBI (**2e**) (0.1 wt%). Excitation at 326 nm, emission at 543 nm. The lifetime was determined to be: τ_1 = 10.9 ± 2.5 ms (1 %); τ_2 = 102.8 ± 0.2 ms (91 %); τ_3 = 307 ± 3 ms (9 %); τ_{avg} = 119 ± 1 ms.

4.1.7 Fluoranthene (G7)

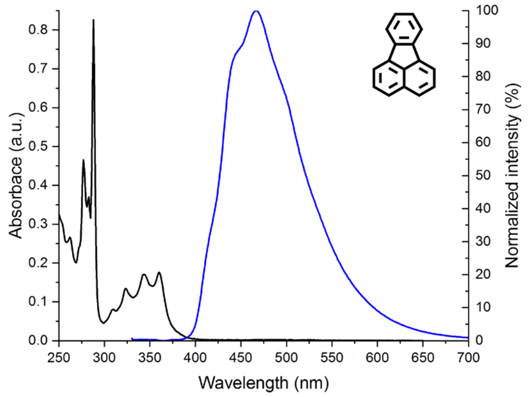

Figure S88. UV-absorption spectrum and normalized fluorescence emission spectrum of fluoranthene (**G7**) in DCM.

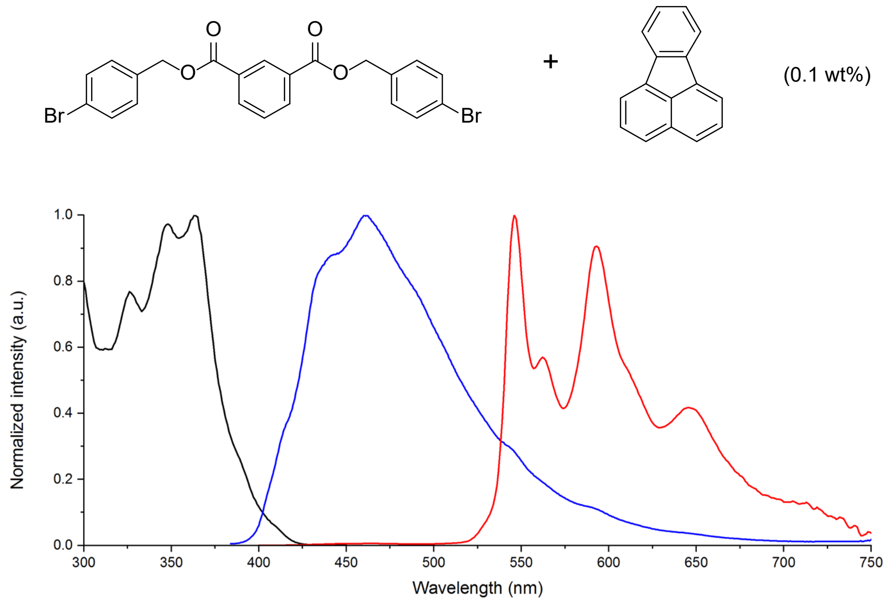

Figure S89. Luminescence of 0.1 wt% of fluoranthene (**G7**) in 4-Br DBI (**2e**). Normalized phosphorescence excitation spectrum (black line; emission at 593 nm), steady-state luminescence emission spectrum (blue line) and phosphorescence spectrum (red line; excitation for both at 364 nm). The total luminescence quantum yield was determined to be $\varphi = 0.317$. The fluorescence quantum yield was determined to be $\varphi_{FL} = 0.312$ and the phosphorescence quantum yield $\varphi_{Phos} = 0.005$.

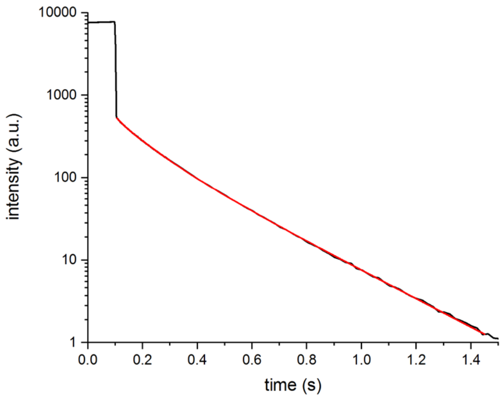

Figure S90. Phosphorescence lifetime decay curve (black line) and fitted lifetime decay (red line) of fluoranthene (**G7**) in 4-Br DBI (**2e**) (0.1 wt%). Excitation at 365 nm, emission at 546 nm. The lifetime was determined to be: τ_1 = 24.5 ± 0.9 ms (7 %); τ_2 = 114.2 ± 1.5 ms (45 %); τ_3 = 253 ± 1 ms (48 %); τ_{avg} = 175 ± 2 ms.

4.1.8 Tetraphene (G8)

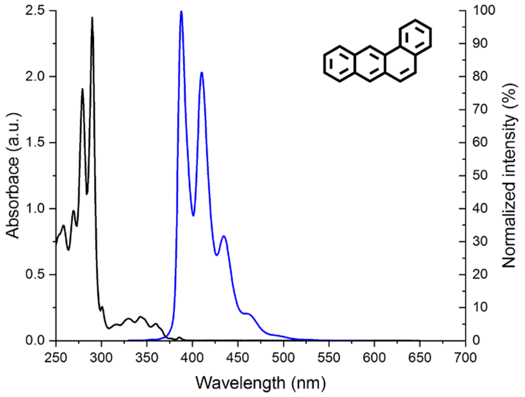

Figure S91. UV-absorption spectrum and normalized fluorescence emission spectrum of tetraphene (**G8**) in DCM.

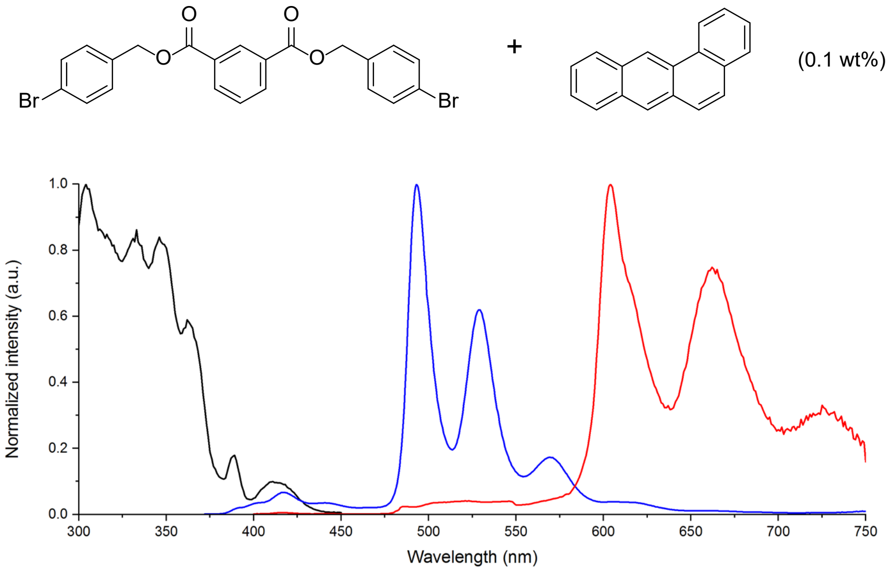

Figure S92. Luminescence of 0.1 wt% of tetraphene (**G8**) in 4-Br DBI (**2e**). Normalized phosphorescence excitation spectrum (black line; emission at 604 nm), steady-state luminescence emission spectrum (blue line) and phosphorescence spectrum (red line; excitation for both at 346 nm). The total luminescence quantum yield was determined to be φ = 0.365. The fluorescence quantum yield was determined to be φ_{FL} = 0.360 and the phosphorescence quantum yield φ_{Phos} = 0.005.

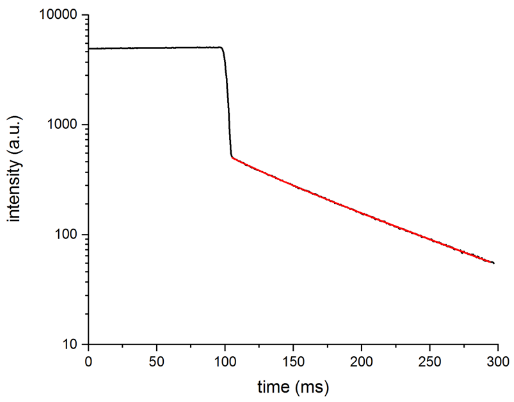

Figure S93. Phosphorescence lifetime decay curve (black line) and fitted lifetime decay (red line) of tetraphene (**G8**) in 4-Br DBI (**2e**) (0.1 wt%). Excitation at 345 nm, emission at 602 nm. The lifetime was determined to be: τ_1 = 32.7 ± 1.2 ms (17 %); τ_2 = 94.2 ± 0.5 ms (83 %); τ_{avg} = 83.9 ± 0.5 ms.

4.1.9 Chrysene (G9)

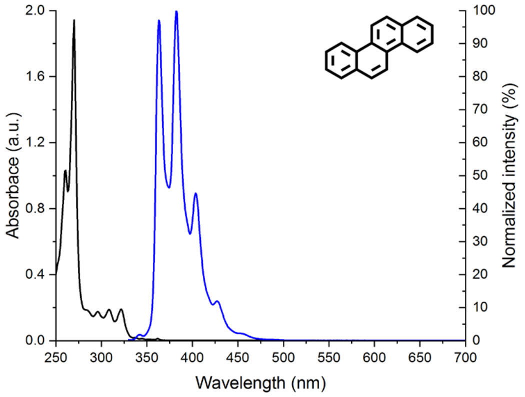

Figure S94. UV-absorption spectrum and normalized fluorescence emission spectrum of chrysene (**G9**) in DCM.

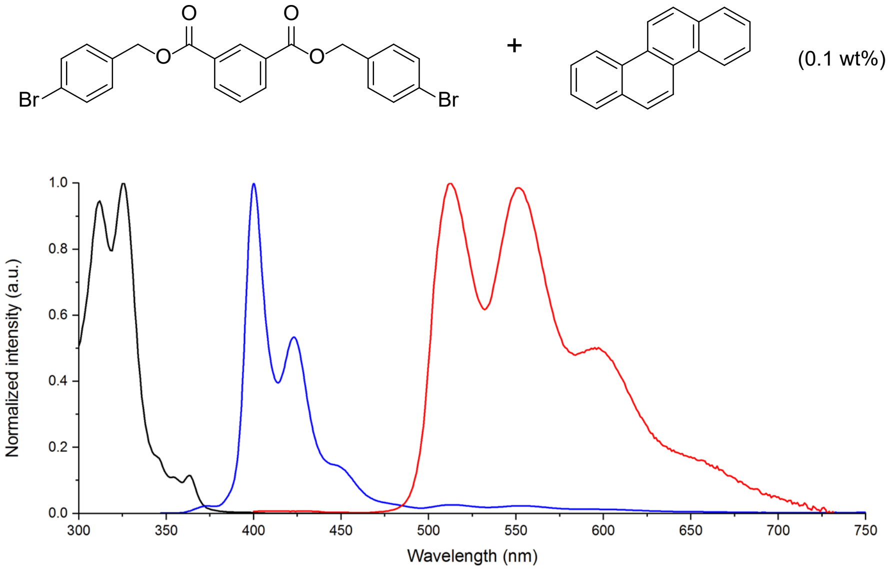

Figure S95. Luminescence of 0.1 wt% of chrysene (**G9**) in 4-Br DBI (**2e**). Normalized phosphorescence excitation spectrum (black line; emission at 552 nm), steady-state luminescence emission spectrum (blue line) and phosphorescence spectrum (red line; excitation for both at 325 nm). The total luminescence quantum yield was determined to be $\varphi = 0.457$. The fluorescence quantum yield was determined to be $\varphi_{FL} = 0.438$ and the phosphorescence quantum yield $\varphi_{Phos} = 0.019$.

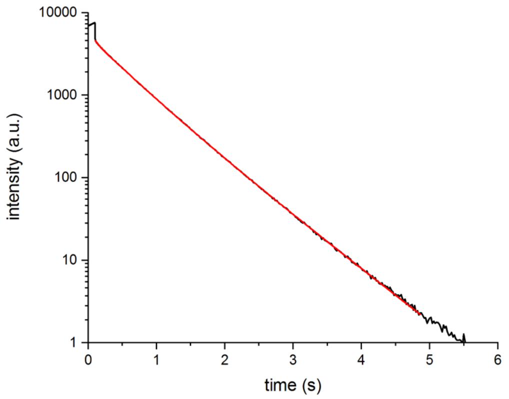

Figure S96. Phosphorescence lifetime decay curve (black line) and fitted lifetime decay (red line) of chrysene (**G9**) in 4-Br DBI (**2e**) (0.1 wt%). Excitation at 325 nm, emission at 512 nm. The lifetime was determined to be: τ_1 = 54.1 ± 0.8 ms (6 %); τ_2 = 467 ± 5 ms (49 %); τ_3 = 691 ± 4 ms (46 %); τ_{avg} = 546 ± 8 ms.

4.1.10 Benzo[*c*]phenanthrene (G10)

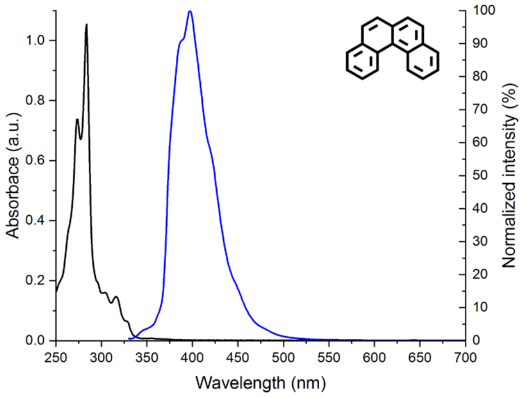

Figure S97. UV-absorption spectrum and normalized fluorescence emission spectrum of benzo[*c*]phenanthrene in DCM.

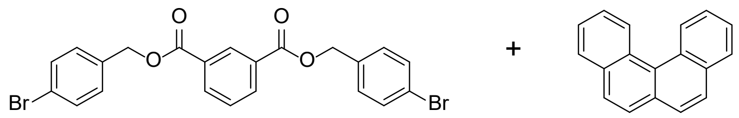

(0.1 wt%)

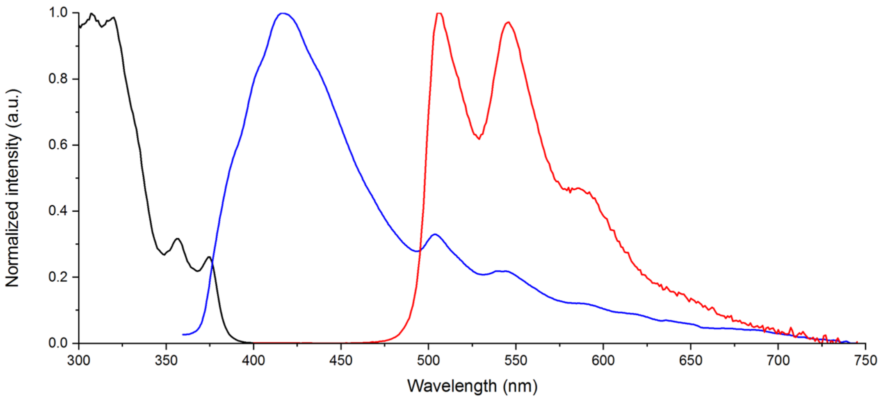

Figure S98. Luminescence of 0.1 wt% of benzo[*c*]phenanthrene (**G10**) in 4-Br DBI (**2e**). Normalized phosphorescence excitation spectrum (black line; emission at 546 nm), steady-state luminescence emission spectrum (blue line) and phosphorescence spectrum (red line; excitation for both at 320 nm). The total luminescence quantum yield was determined to be φ = 0.136. The fluorescence quantum yield was determined to be φ_{FL} = 0.122 and the phosphorescence quantum yield φ_{Phos} = 0.014.

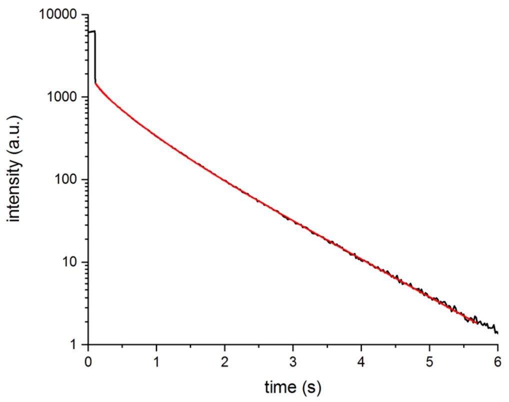

Figure S99. Phosphorescence lifetime decay curve (black line) and fitted lifetime decay (red line) of benzo[*c*]phenanthrene (**G10**) in 4-Br DBI (**2e**) (0.1 wt%). Excitation at 325 nm, emission at 505 nm. The lifetime was determined to be: τ_1 = 104 ± 3 ms (12 %); τ_2 = 433 ± 5 ms (44 %); τ_3 = 950 ± 3 ms (44 %); τ_{avg} = 623 ± 4 ms.

4.1.11 Pyrene (G11)

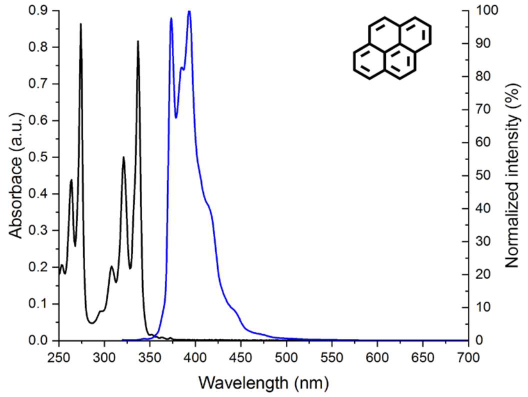

Figure S100. UV-absorption spectrum and normalized fluorescence emission spectrum of pyrene (**G11**) in DCM.

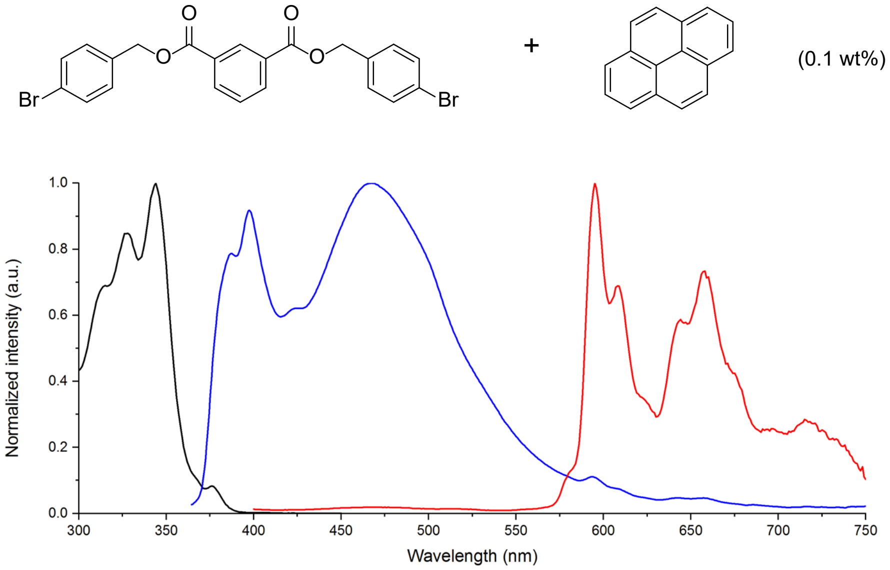

Figure S101. Luminescence of 0.1 wt% of pyrene (**G11**) in 4-Br DBI (**2e**). Normalized phosphorescence excitation spectrum (black line; emission at 593 nm), steady-state luminescence emission spectrum (blue line) and phosphorescence spectrum (red line; excitation for both at 344 nm). The total luminescence quantum yield was determined to be φ = 0.280. The fluorescence quantum yield was determined to be φ_{FL} = 0.269 and the phosphorescence quantum yield φ_{Phos} = 0.011.

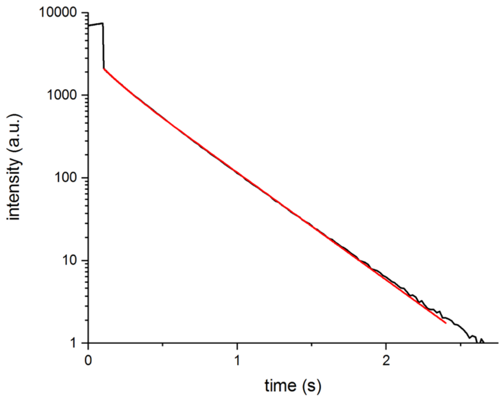

Figure S102. Phosphorescence lifetime decay curve (black line) and fitted lifetime decay (red line) of pyrene (**G11**) in 4-Br DBI (**2e**) (0.1 wt%). Excitation at 345 nm, emission at 595 nm. The lifetime was determined to be: τ_1 = 119 ± 2 ms (19 %); τ_2 = 334 ± 1 ms (81 %); τ_{avg} = 294 ± 1 ms.

4.1.12 5*H*-naphtho[8,1,2-*cde*]chromen-5-one (G12)

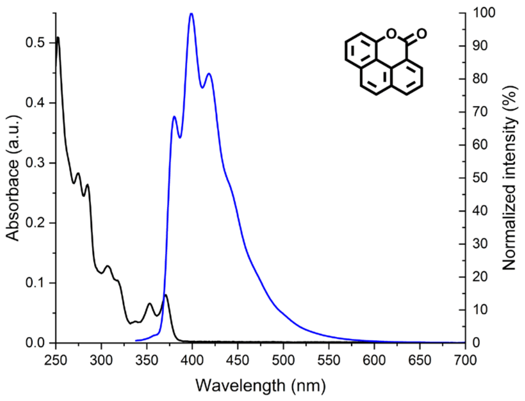

Figure S103. UV-absorption spectrum and normalized fluorescence emission spectrum of 5*H*-naphtho[8,1,2-*cde*]chromen-5-one (**G12**) in DCM. The fluorescence quantum yield of this compound was determined to be Φ = 0.070 in DCM.

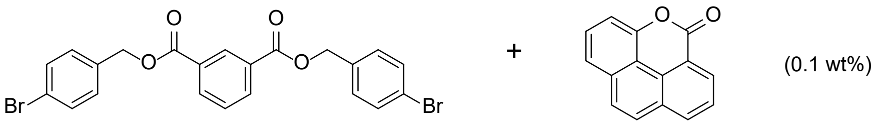

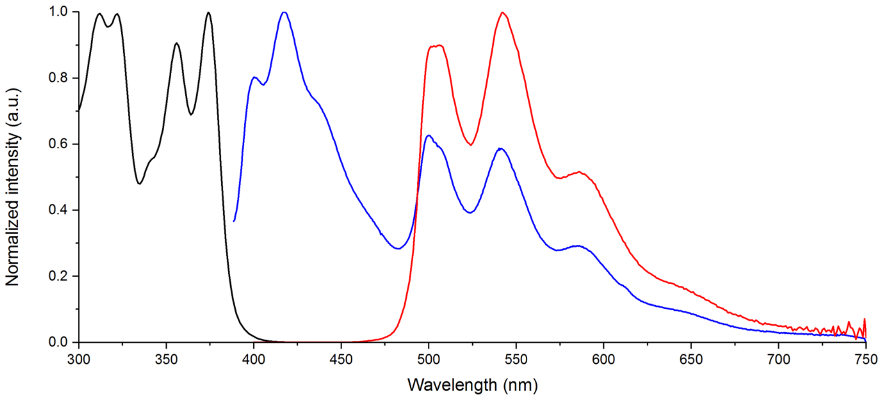

Figure S104. Luminescence of 0.1 wt% of 5*H*-naphtho[8,1,2-*cde*]chromen-5-one (**G12**) in 4-Br DBI (**2e**). Normalized phosphorescence excitation spectrum (black line; emission at 542 nm), steady-state luminescence emission spectrum (blue line) and phosphorescence spectrum (red line; excitation for both at 374 nm). The total luminescence quantum yield was determined to be $\varphi = 0.090$. The fluorescence quantum yield was determined to be $\varphi_{FL} = 0.052$ and the phosphorescence quantum yield $\varphi_{Phos} = 0.038$.

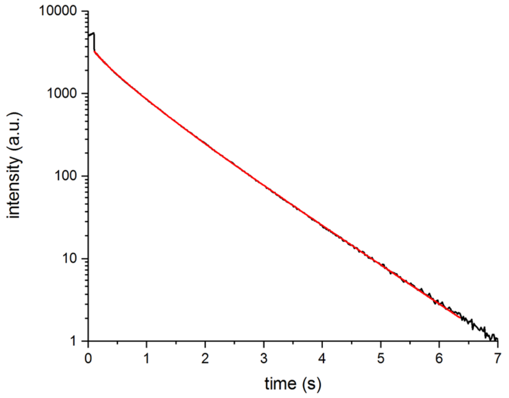

Figure S105. Phosphorescence lifetime decay curve (black line) and fitted lifetime decay (red line) of 5*H*-naphtho[8,1,2-*cde*]chromen-5-one (**G12**) in 4-Br DBI (**2e**) (0.1 wt%). Excitation at 375 nm, emission at 505 nm. The lifetime was determined to be: τ_1 = 170 ± 8 ms (12 %); τ_2 = 519 ± 18 ms (36 %); τ_3 = 921 ± 6 ms (52 %); τ_{avg} = 688 ± 11 ms.

4.1.13 5*H*-phenanthro[1,10,9-*cde*]chromen-5-one (G13)

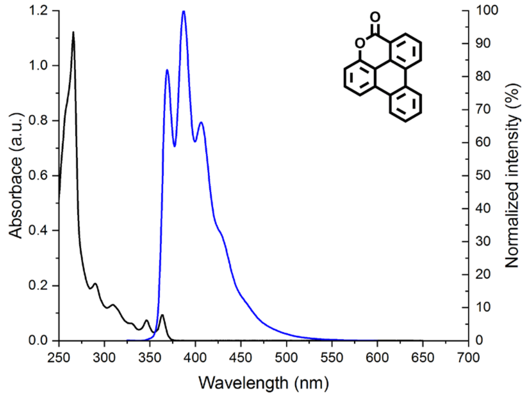

Figure S106. UV-absorption spectrum and normalized fluorescence emission spectrum of 5*H*-phenanthro[1,10,9-*cde*]chromen-5-one (**G13**) in DCM. The fluorescence quantum yield of this compound was determined to be φ = 0.097 in DCM.

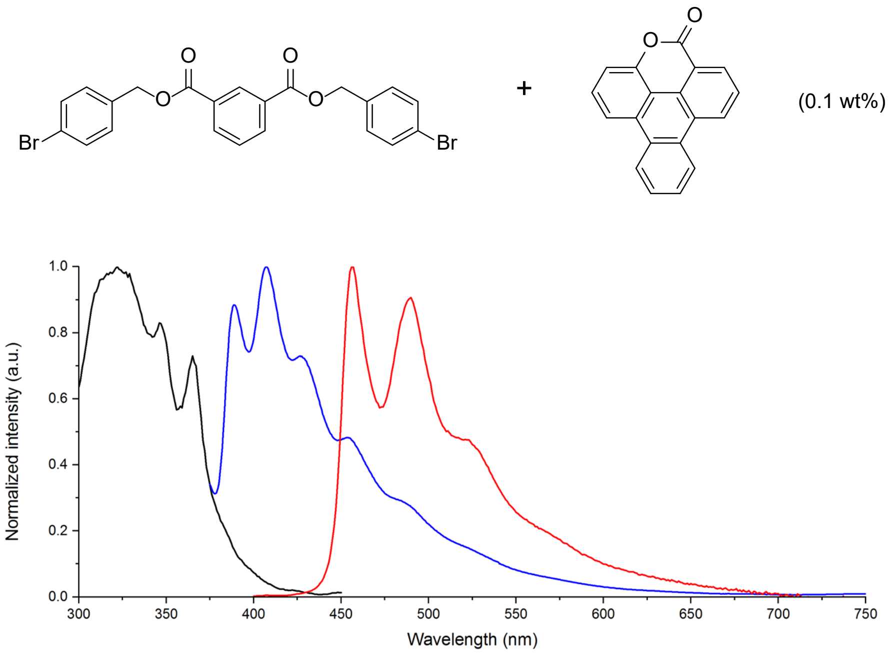

Figure S107. Luminescence of 0.1 wt% of 5*H*-phenanthro[1,10,9-*cde*]chromen-5-one (**G13**) in 4-Br DBI (**2e**). Normalized phosphorescence excitation spectrum (black line; emission at 457 nm), steady-state luminescence emission spectrum (blue line) and phosphorescence spectrum (red line; excitation for both at 370 nm). The total luminescence quantum yield was determined to be φ = 0.150. The fluorescence quantum yield was determined to be φ_{FL} = 0.140 and the phosphorescence quantum yield φ_{Phos} = 0.010.

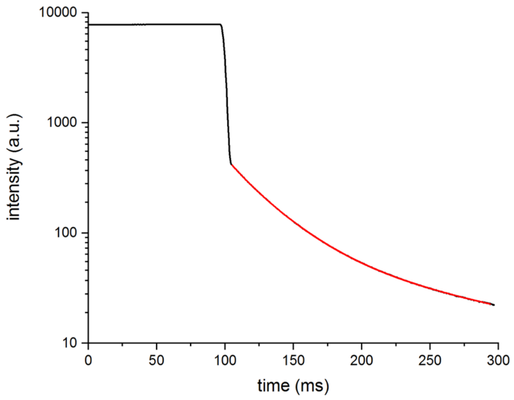

Figure S108. Phosphorescence lifetime decay curve (black line) and fitted lifetime decay (red line) of 5*H*-phenanthro[1,10,9-*cde*]chromen-5-one (**G13**) in 4-Br DBI (**2e**) (0.1 wt%). Excitation at 365 nm, emission at 490 nm. The lifetime was determined to be: τ_1 = 14.5 ± 0.5 ms (14 %); τ_2 = 33.4 ± 0.3 ms (72 %); τ_3 = 184 ± 2 ms (15 %); τ_{avg} = 52.9 ± 0.6 ms.

4.1.14 2,3,6,7,10,11-Hexamethoxytriphenylene (G14)

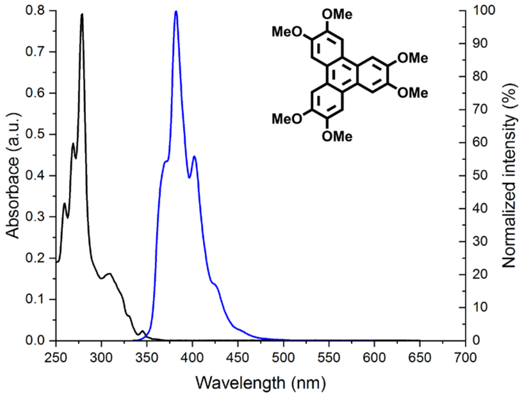

Figure S109. UV-absorption spectrum and normalized fluorescence emission spectrum of 2,3,6,7,10,11-hexamethoxytriphenylene (**G14**) in DCM.

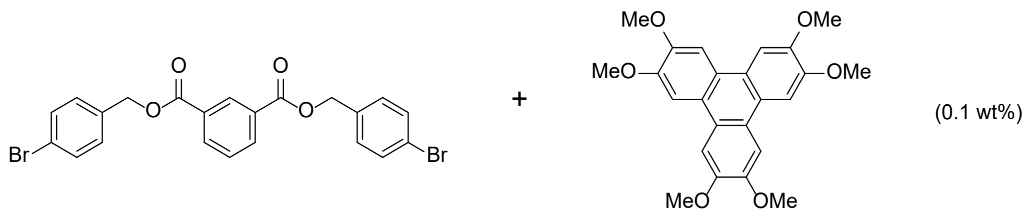

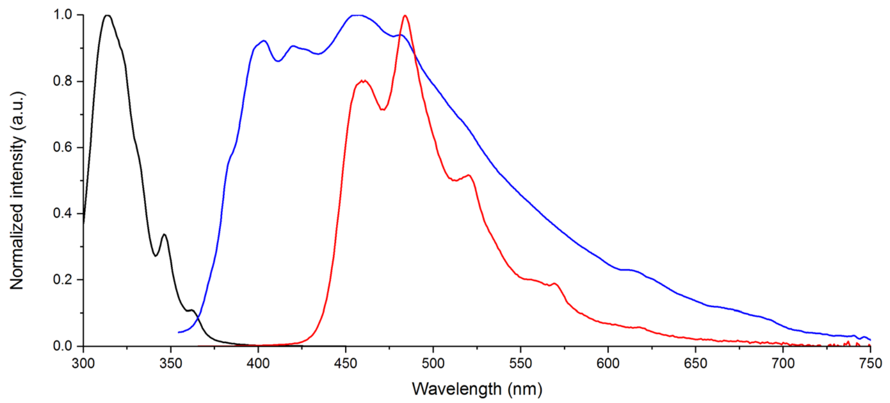

Figure S110. Luminescence of 0.1 wt% of 2,3,6,7,10,11-hexamethoxytriphenylene (**G14**) in 4-Br DBI (**2e**). Normalized phosphorescence excitation spectrum (black line; emission at 460 nm), steady-state luminescence emission spectrum (blue line) and phosphorescence spectrum (red line; excitation for both at 313 nm). The total luminescence quantum yield was determined to be φ = 0.076. The fluorescence quantum yield was determined to be φ_{FL} = 0.072 and the phosphorescence quantum yield φ_{Phos} = 0.004.

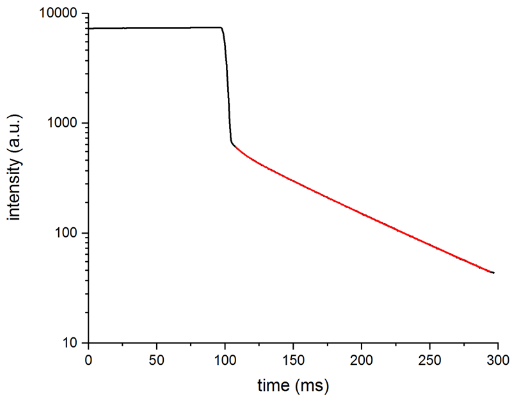

Figure S111. Phosphorescence lifetime decay curve (black line) and fitted lifetime decay (red line) of 2,3,6,7,10,11-hexamethoxytriphenylene (**G14**) in 4-Br DBI (**2e**) (0.1 wt%). Excitation at 315 nm, emission at 457 nm. The lifetime was determined to be: τ_1 = 7.33 ± 0.20 ms (10 %); τ_2 = 46.1 ± 3.1 ms (23 %); τ_3 = 82.4 ± 1.3 ms (67 %); τ_{avg} = 66.3 ± 2.2 ms.

4.1.15 Benzo[*ghi*]perylene (G15)

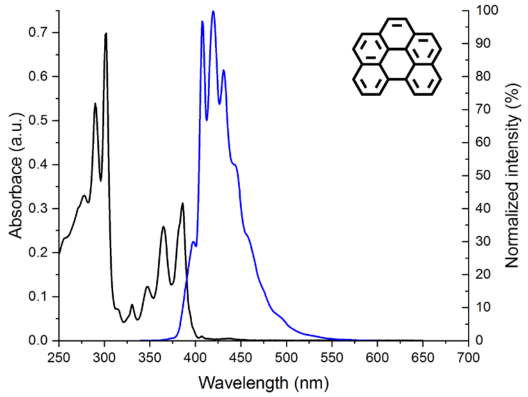

Figure S112. UV-absorption spectrum and normalized fluorescence emission spectrum of benzo[*ghi*]perylene (**G15**) in DCM.

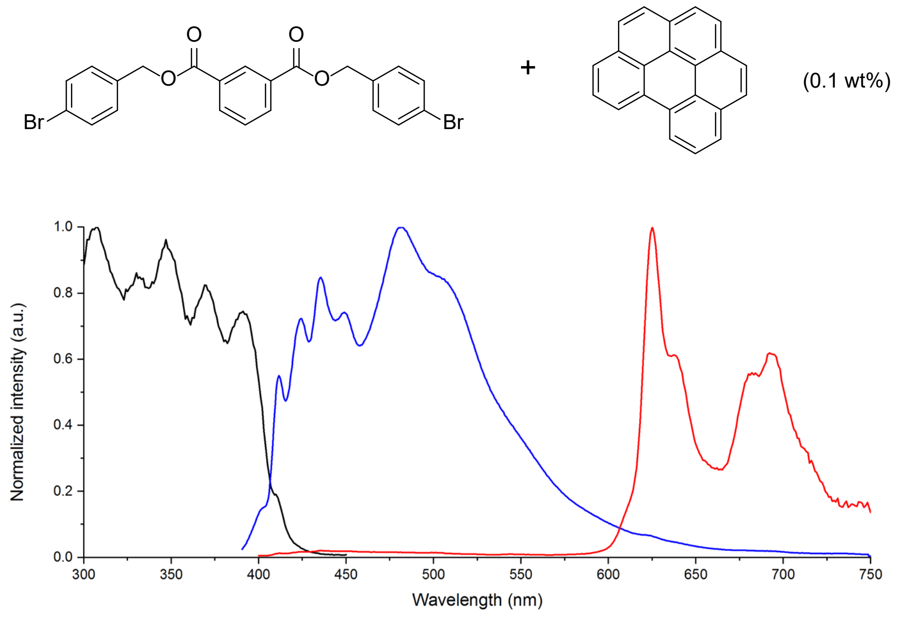

Figure S113. Luminescence of 0.1 wt% of benzo[*ghi*]perylene (**G15**) in 4-Br DBI (**2e**). Normalized phosphorescence excitation spectrum (black line; emission at 612 nm), steady-state luminescence emission spectrum (blue line) and phosphorescence spectrum (red line; excitation for both at 372 nm). The total luminescence quantum yield was determined to be φ = 0.385. The fluorescence quantum yield was determined to be φ_{FL} = 0.382 and the phosphorescence quantum yield φ_{Phos} = 0.003.

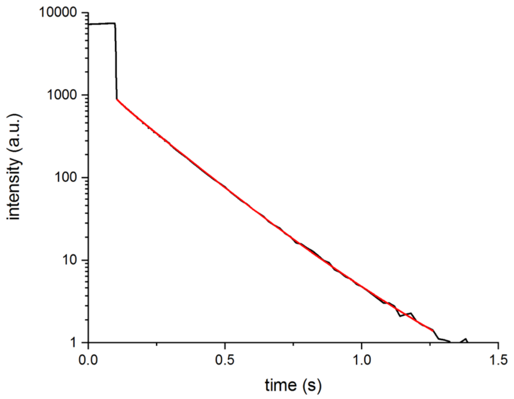

Figure S114. Phosphorescence lifetime decay curve (black line) and fitted lifetime decay (red line) of benzo[*ghi*]perylene (**G15**) in 4-Br DBI (**2e**) (0.1 wt%). Excitation at 372 nm, emission at 612 nm. The lifetime was determined to be: τ_1 = 33.0 ± 1.2 ms (7 %); τ_2 = 164 ± 1 ms (94 %); τ_{avg} = 156 ± 1 ms.

4.1.16 Pyrene-d_{10}

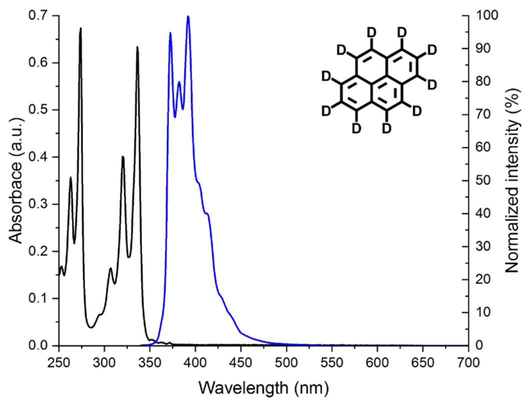

Figure S115. UV-absorption spectrum and normalized fluorescence emission spectrum of pyrene-d_{10} in DCM.

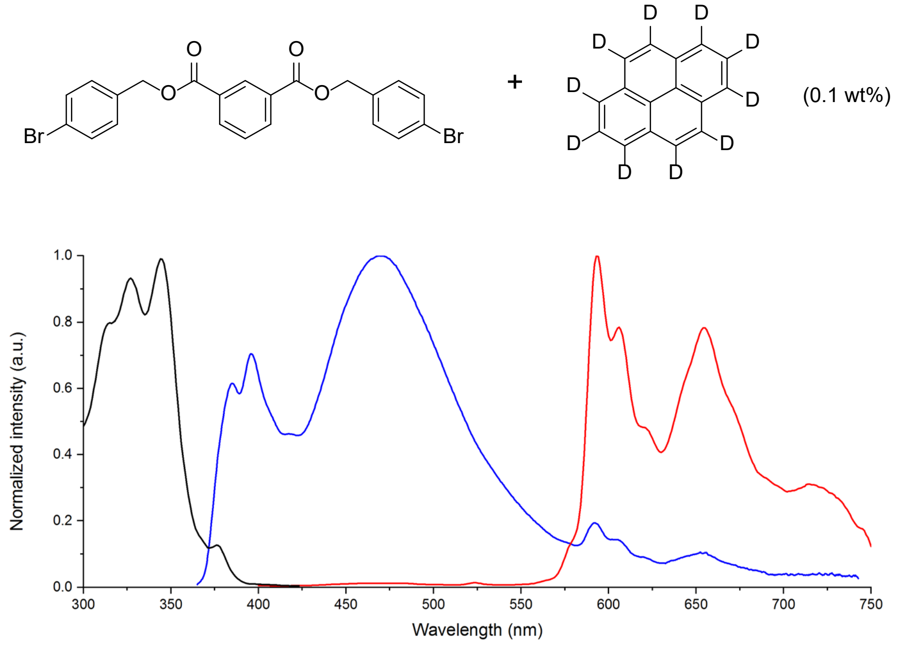

Figure S116. Luminescence of 0.1 wt% of pyrene-d_{10} in 4-Br DBI (**2e**). Normalized phosphorescence excitation spectrum (black line; emission at 595 nm), steady-state luminescence emission spectrum (blue line) and phosphorescence spectrum (red line; excitation for both at 345 nm). The total luminescence quantum yield was determined to be φ = 0.219. The fluorescence quantum yield was determined to be φ_{FL} = 0.192 and the phosphorescence quantum yield φ_{Phos} = 0.027.

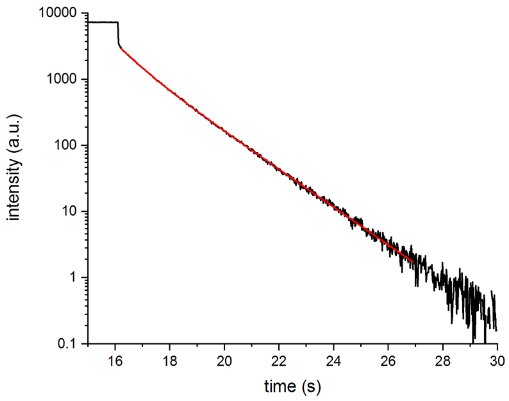

Figure S117. Phosphorescence lifetime decay curve (black line) and fitted lifetime decay (red line) of pyrene-d_{10} in 4-Br DBI (**2e**) (0.1 wt%). Excitation at 345 nm, emission at 595 nm. The lifetime was determined to be: τ_1 = 0.75 ± 0.02 s (34 %); τ_2 = 1.53 ± 0.01 s (66 %); τ_{avg} = 1.26 ± 0.02 s.

4.2 Effect of different pyrene-d_{10} concentrations in the 4-Br DBI (2e) on the luminescence emission

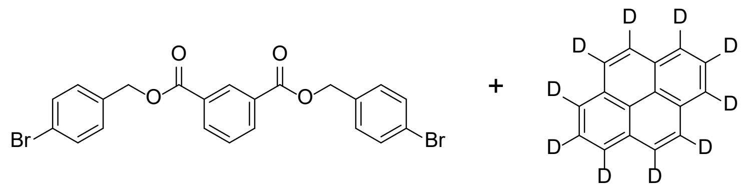

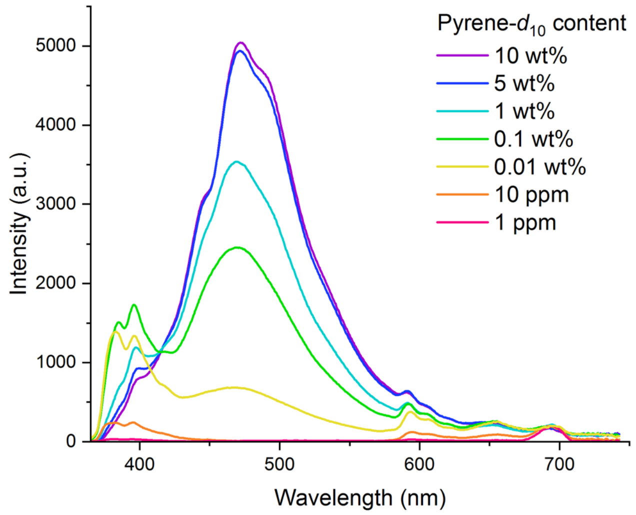

Figure S118. Comparison of different concentrations of pyrene-d_{10} in the 4-Br DBI (**2e**). Steady state luminescence spectra (excitation at 345 nm). The 2nd order diffraction maxima at 690 nm were left present as internal standards since the spectra are not normalized. The spectra were measured in the integrating sphere.

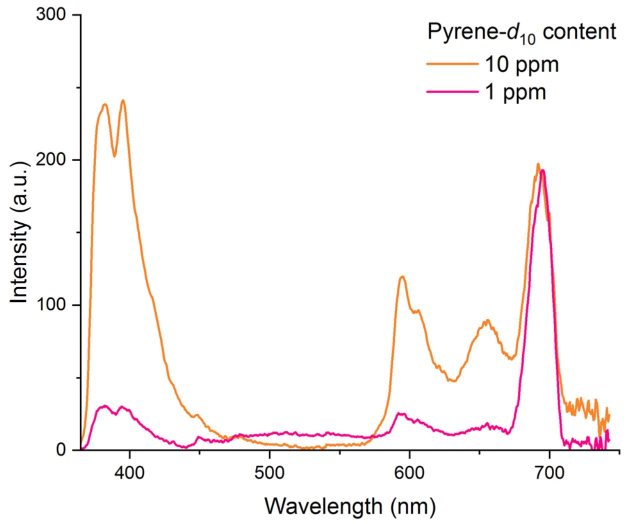

Figure S119. Enlarged spectra of the 10 ppm and 1 ppm wt% doping amounts from the previous figure for better visibility.

Note: The normalized phosphorescence spectra were unaffected by different wt% of pyrene-d_{10}.

4.3 Photophysical properties of other isophthalate ester based host-guest systems

4.3.1 4-Cl DBI (2b) with 0.1 wt% pyrene-d_{10}

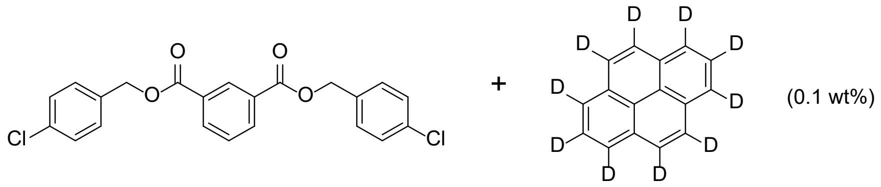

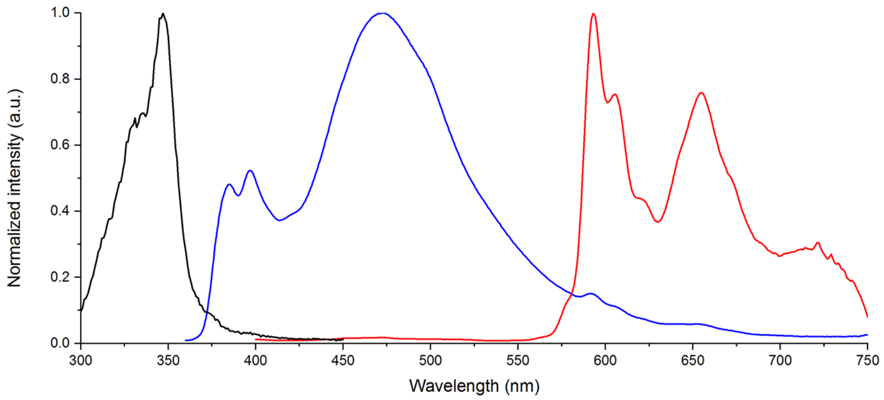

Figure S120. Luminescence of 0.1 wt% of pyrene-d_{10} in 4-Cl DBI (**2b**). Normalized phosphorescence excitation spectrum (black line; emission at 593 nm), steady-state luminescence emission spectrum (blue line) and phosphorescence spectrum (red line; excitation for both at 347 nm). The total luminescence quantum yield was determined to be φ = 0.373. The fluorescence quantum yield was determined to be φ_{FL} = 0.359 and the phosphorescence quantum yield φ_{Phos} = 0.014.

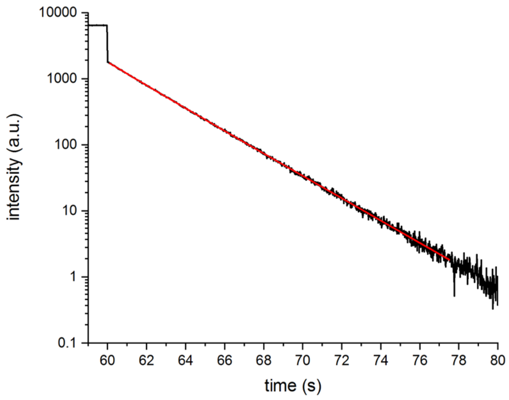

Figure S121. Phosphorescence lifetime decay curve (black line) and fitted lifetime decay (red line) of pyrene-d_{10} in 4-Cl DBI (**2b**) (0.1 wt%). Exitation at 347 nm, emission at 593 nm. The lifetime was determined to be: τ_1 = 1.37 ± 0.05 s (8 %); τ_2 = 2.57 ± 0.01 s (93 %); τ_{avg} = 2.48 ± 0.04 s.

4.3.2 4-I DBI (2g) with 0.1 wt% pyrene-d_{10}

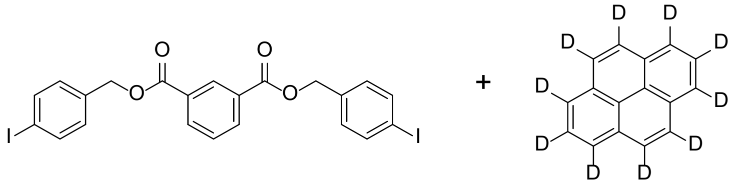

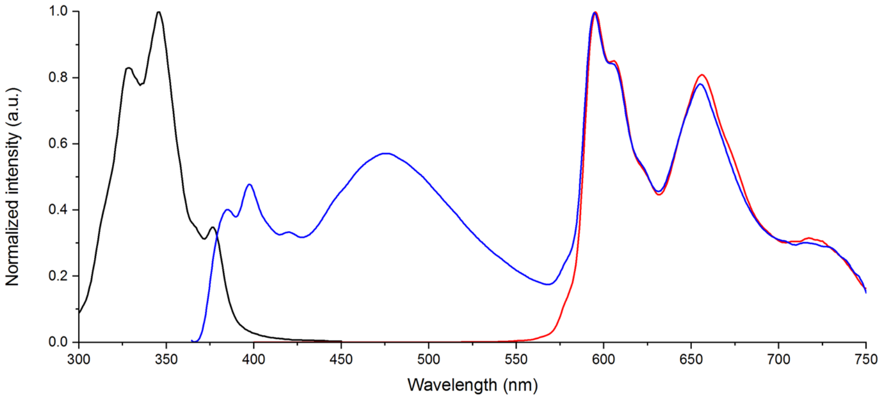

Figure S122. Luminescence of 0.1 wt% of pyrene-d_{10} in 4-I DBI (**2g**). Normalized phosphorescence excitation spectrum (black line; emission at 595 nm), steady-state luminescence emission spectrum (blue line) and phosphorescence spectrum (red line; excitation for both at 345 nm). The total luminescence quantum yield was determined to be φ = 0.075. The fluorescence quantum yield was determined to be φ_{FL} = 0.034 and the phosphorescence quantum yield φ_{Phos} = 0.041.

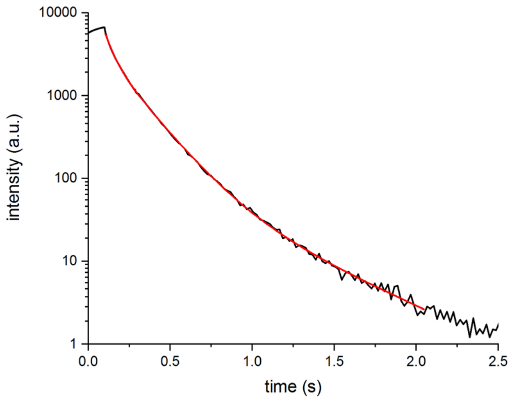

Figure S123. Phosphorescence lifetime decay curve (black line) and fitted lifetime decay (red line) of pyrene-d_{10} in 4-I DBI (**2g**) (0.1 wt%). Excitation at 345 nm, emission at 595 nm. The lifetime was determined to be: τ_1 = 46.1 ± 1.4 ms (45 %); τ_2 = 173 ± 2 ms (52 %); τ_3 = 489 ± 9 ms (3 %); τ_{avg} = 124 ± 2 ms.

4.3.3 All-H DBI (2a) with 0.1 wt% pyrene-d_{10}

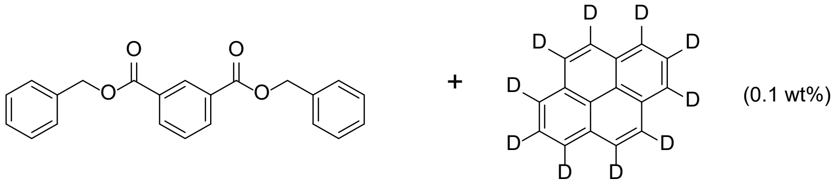

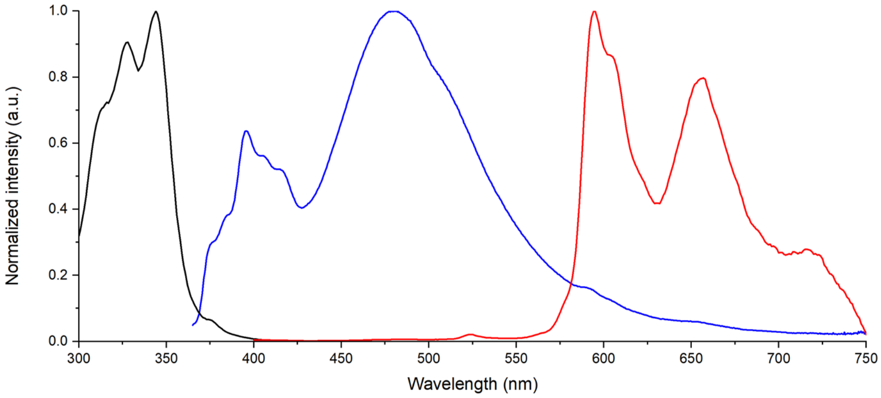

Figure S124. Luminescence of 0.1 wt% of pyrene-d_{10} in all-H DBI (**2a**). Normalized phosphorescence excitation spectrum (black line; emission at 595 nm), steady-state luminescence emission spectrum (blue line) and phosphorescence spectrum (red line; excitation for both at 345 nm). The total luminescence quantum yield was determined to be φ = 0.041. The fluorescence quantum yield was determined to be φ_{FL} = 0.041 and the phosphorescence quantum yield φ_{Phos} ≈ 0.0006.

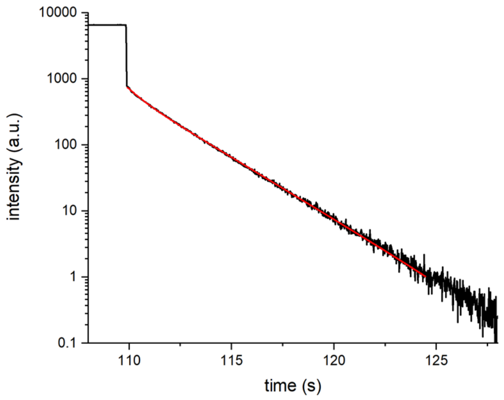

Figure S125. Phosphorescence lifetime decay curve (black line) and fitted lifetime decay (red line) of pyrene-d_{10} in all-H DBI (**2a**) (0.1 wt%). Excitation at 345 nm, emission at 595 nm. The lifetime was determined to be: τ_1 = 0.69 ± 0.03 s (22 %); τ_2 = 2.32 ± 0.01 s (78 %); τ_{avg} = 1.96 ± 0.02 s.

4.3.4 All-H DBI (2a) with 1 wt% 4-Br DBI (2e) and 0.1 wt% pyrene-d_{10}

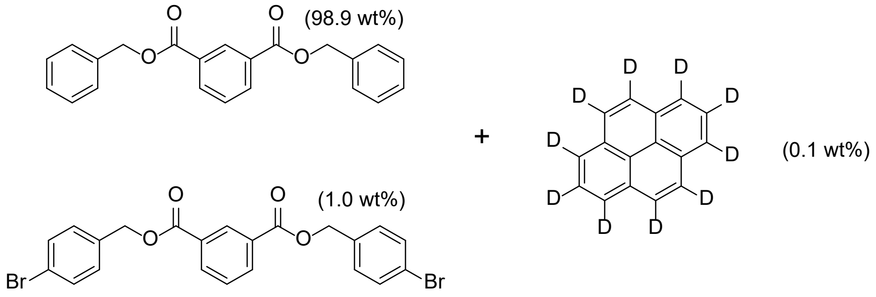

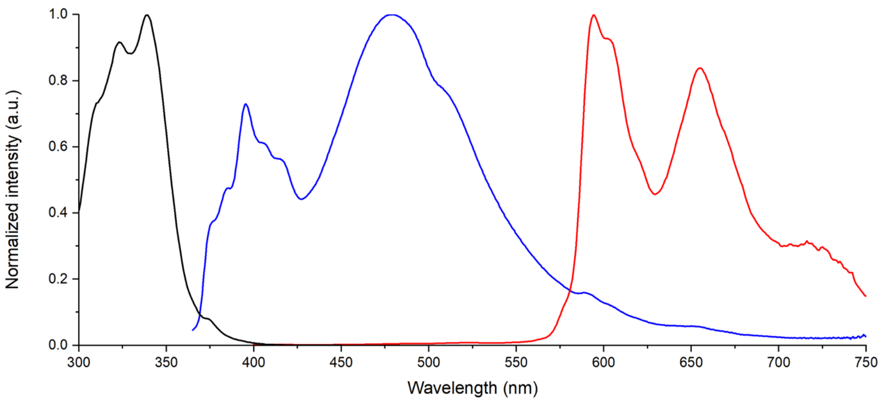

Figure S126. Luminescence of 0.1 wt% of pyrene-d_{10} in a composite of all-H DBI (**2a**, 98.9 wt%) and 4-Br DBI (**2e**, 1.0 wt%). Normalized phosphorescence excitation spectrum (black line; emission at 595 nm), steady-state luminescence emission spectrum (blue line) and phosphorescence spectrum (red line; excitation for both at 345 nm). The total luminescence

quantum yield was determined to be φ ≈ 0.1058 (0.106). The fluorescence quantum yield was determined to be φ_{FL} = 0.104 and the phosphorescence quantum yield φ_{Phos} ≈ 0.0017.

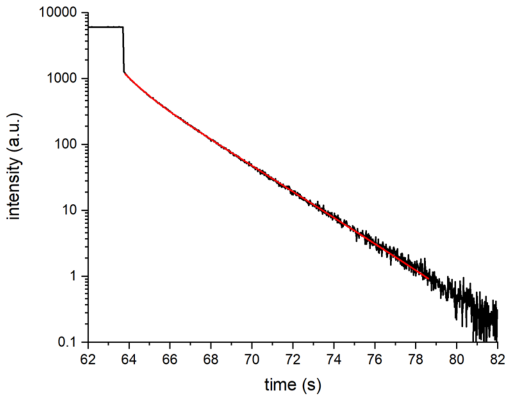

Figure S127. Phosphorescence lifetime decay curve (black line) and fitted lifetime decay (red line) of pyrene-d_{10} (0.1 wt%) in a composite of all-H DBI (**2a**, 98.9 wt%) and 4-Br DBI (**2e**, 1.0 wt%). Excitation at 345 nm, emission at 595 nm. The lifetime was determined to be: τ_1 = 0.79 ± 0.02 s (34 %); τ_2 = 2.21 ± 0.01 s (66 %); τ_{avg} = 1.74 ± 0.01 s.

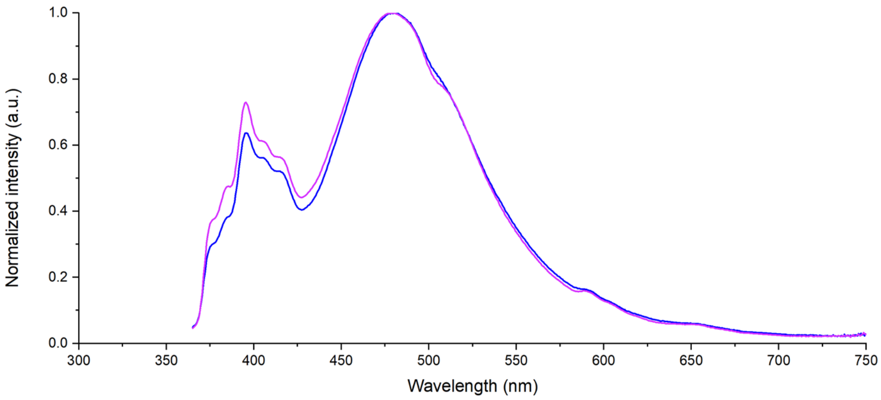

Figure S128. Comparison of normalized steady-state spectra of of 0.1 wt% of pyrene-d_{10} in all-H DBI (**2a**) (blue line), and of 0.1 wt% of pyrene-d_{10} in a composite of all-H DBI (**2a**, 98.9 wt%) and 4-Br DBI (**2e**, 1.0 wt%) (purple line). Excitation at 345 nm.

4.3.5 4-CF_3 DBI (2a) with 0.1 wt% pyrene-d_{10}; with and without 1 wt% 4-Br DBI (2e)

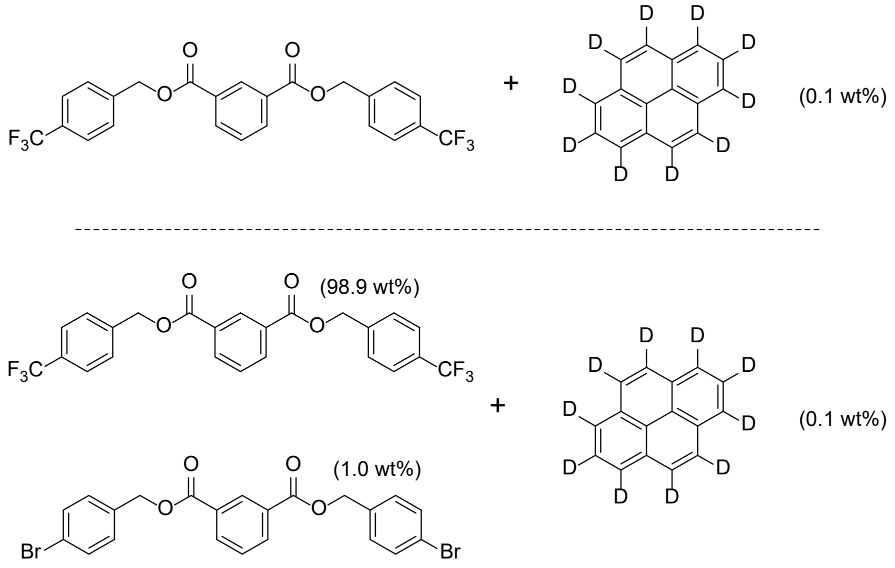

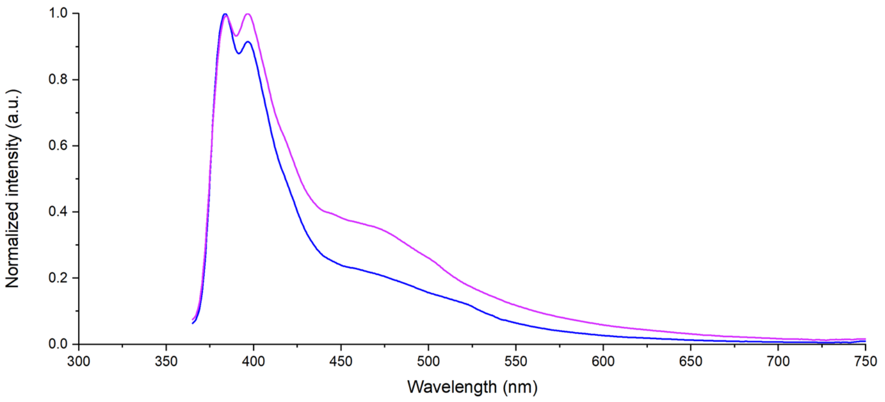

Figure S129. Normalized steady-state luminescence of 0.1 wt% pyrene-d_{10} in 4-CF_3 DBI (**2t**) (blue line) and normalized steady-state luminescence of 0.1 wt% pyrene-d_{10} in 4-CF_3 DBI (**2t**, 98.9 wt%) with 4-Br DBI (**2e**, 1 wt%) (purple line; excitation for both at 345 nm). The total luminescence quantum yields were determined to be φ = 0.089 (only **2a** + pyrene-d_{10}) and φ = 0.071 (with 1 wt% **2e**).

4.3.6 4-Br DBI (2e) with 1 wt% 4- CF_3 DBI (2t) and 0.1 wt% pyrene-d_{10}

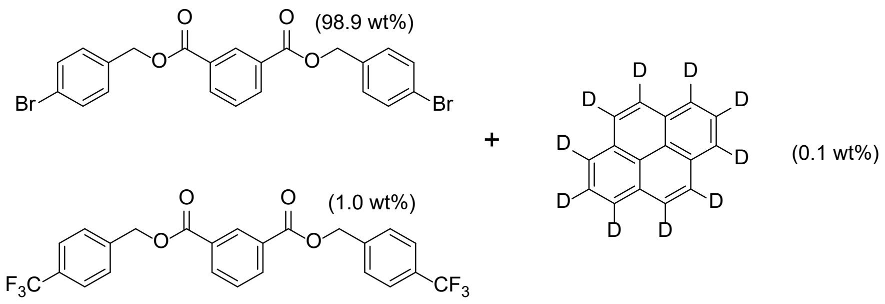

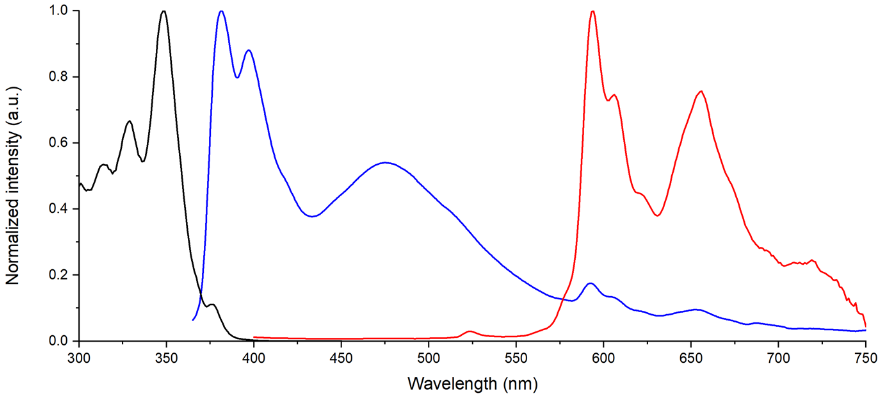

Figure S130. Luminescence of 0.1 wt% of pyrene-d_{10} in a composite of 4-Br DBI (**2e**, 98.9 wt%) and 4-CF_3 DBI (**2t**, 1.0 wt%). Normalized phosphorescence excitation spectrum (black line; emission at 595 nm), steady-state luminescence emission spectrum (blue line) and phosphorescence spectrum (red line; excitation for both at 345 nm). The total luminescence quantum yield was determined to be φ = 0.110. The fluorescence quantum yield was determined to be φ_{FL} = 0.098 and the phosphorescence quantum yield φ_{Phos} = 0.012.

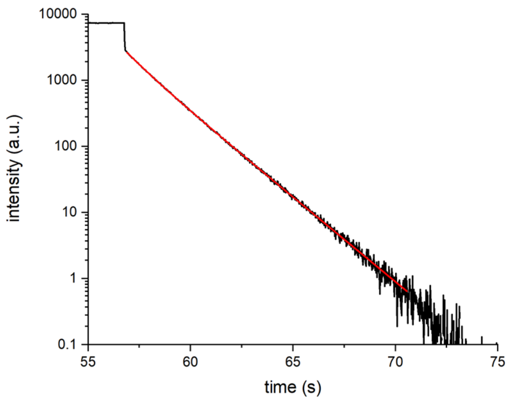

Figure S131. Phosphorescence lifetime decay curve (black line) and fitted lifetime decay (red line) of pyrene-d_{10} (0.1 wt%) in a composite of 4-Br DBI (**2e**, 98.9 wt%) and 4-CF_3 DBI (**2t**, 1.0 wt%). Excitation at 345 nm, emission at 595 nm. The lifetime was determined to be: τ_1 = 0.91 ± 0.02 s (23 %); τ_2 = 1.70 ± 0.01 s (77 %); τ_{avg} = 1.52 ± 0.02 s.

4.3.7 *p*-Toluic acid with 1 wt% 4-Br DBI (2e) and 0.1 wt% pyrene-*d*₁₀

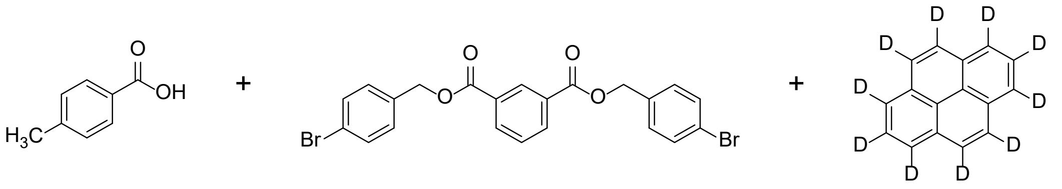

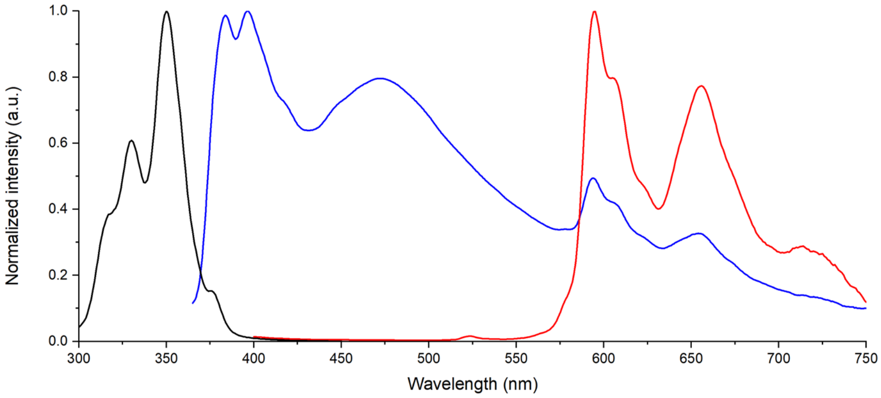

Figure S132. Normalized steady-state luminescence spectrum (blue line) of 0.1 wt% pyrene-d_{10} in a mixture of *p*-toluic acid (98.9 wt%) and 4-Br DBI (**2e**) (1 wt%). Phosphorescence excitation spectrum (emission at 593 nm, black line) and phosphorescence emission spectrum (red line) of the same host/guest system. Excitation at 345 nm. The total luminescence quantum yield was determined to be φ = 0.064. The fluorescence quantum yield was determined to be φ_{FL} = 0.054 and the phosphorescence quantum yield φ_{Phos} = 0.010.

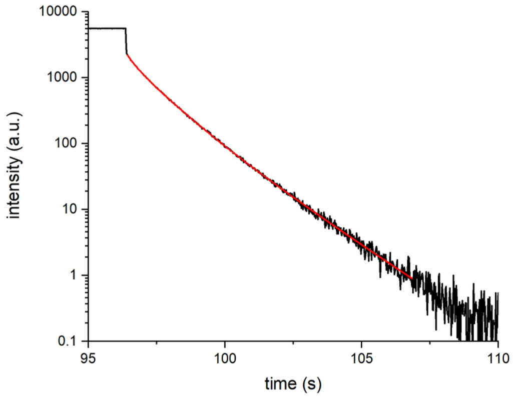

Figure S133. Phosphorescence lifetime decay curve (black line) and fitted lifetime decay (red line) of 0.1 wt% pyrene-d_{10} in a mixture of *p*-toluic acid (98.9 wt%) and 4-Br DBI (**2e**) (1 wt%). Excitation at 345 nm, emission at 595 nm. The lifetime was determined to be: τ_1 = 0.27 ± 0.02 s (17 %); τ_2 = 0.93 ± 0.03 s (54 %); τ_3 = 1.58 ± 0.03 s (29 %); τ_{avg} = 1.01 ± 0.04 s.

4.3.8 *p*-Toluic acid with 5 wt% 4-Br DBI (2e) and 100 ppm pyrene-d_{10}

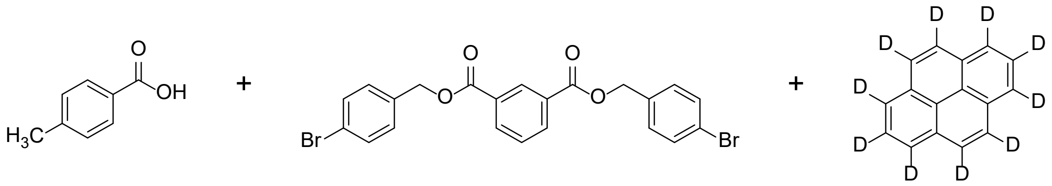

(94.99 wt%) (5 wt%) (0.01 wt%)

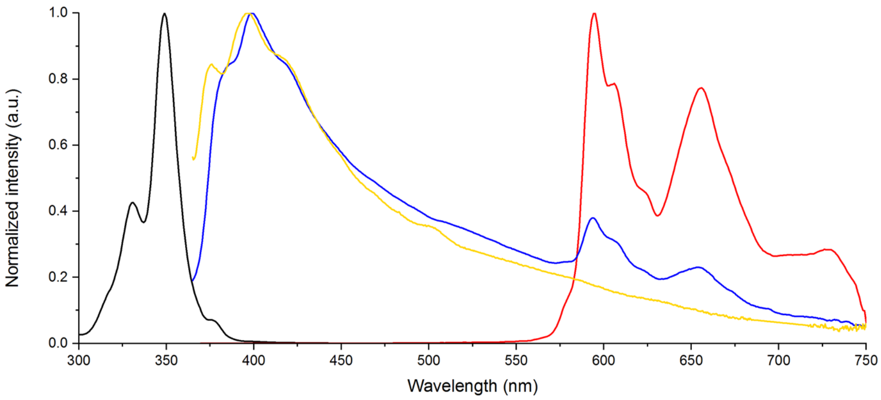

Figure S134. Normalized steady-state luminescence spectrum (blue line) of 100 ppm pyrene-d_{10} in a mixture of *p*-toluic acid (95 wt%) and 4-Br DBI (**2e**) (5 wt%) and normalized control spectrum (orange line, no 4-Br DBI (**2e**), same concentration of pyrene-d_{10}). Phosphorescence excitation spectrum (emission at 593 nm, black line) and phosphorescence emission spectrum (red line) of the same host/guest system. Excitation at 349 nm.

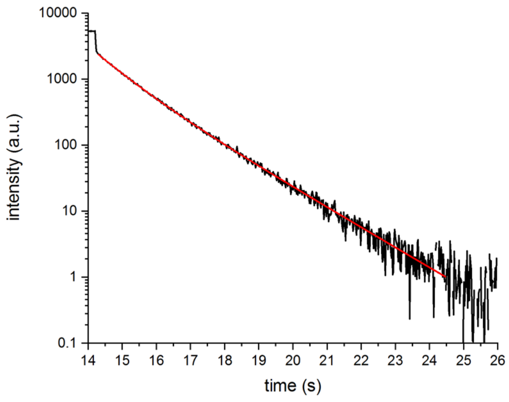

Figure S135. Phosphorescence lifetime decay curve (black line) and fitted lifetime decay (red line) of 100 ppm pyrene-d_{10} in a mixture of *p*-toluic acid (95 wt%) and 4-Br DBI (**2e**) (5 wt%). Excitation at 349 nm, emission at 595 nm. The lifetime was determined to be: τ_1 = 0.79 ± 0.02 s (49 %); τ_2 = 1.44 ± 0.02 s (51 %); τ_{avg} = 1.12 ± 0.03 s.

4.3.9 *p*-Toluic acid with 0.1 wt% 4-Br DBI (2e) and 100 ppm pyrene-d_{10}

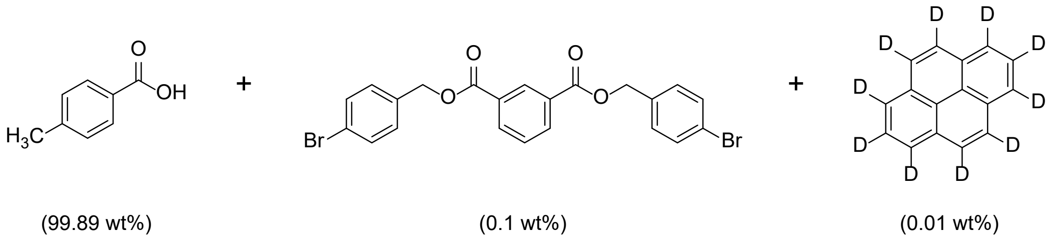

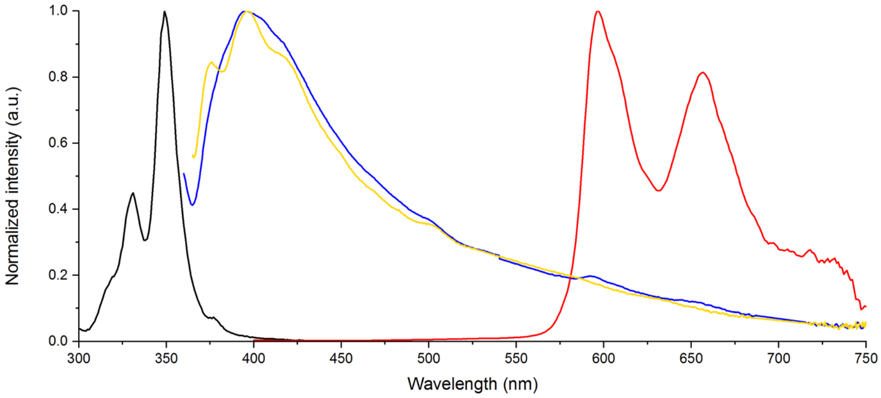

Figure S136. Normalized steady-state luminescence spectrum (blue line) of 100 ppm pyrene-d_{10} in a mixture of *p*-toluic acid (99.89 wt%) and 4-Br DBI (**2e**) (0.1 wt%) and normalized control spectrum (orange line, no 4-Br DBI (**2e**), same concentration of pyrene-d_{10}). Phosphorescence excitation spectrum (emission at 593 nm, black line) and phosphorescence emission spectrum (red line) of the same host/guest system. Excitation at 349 nm.

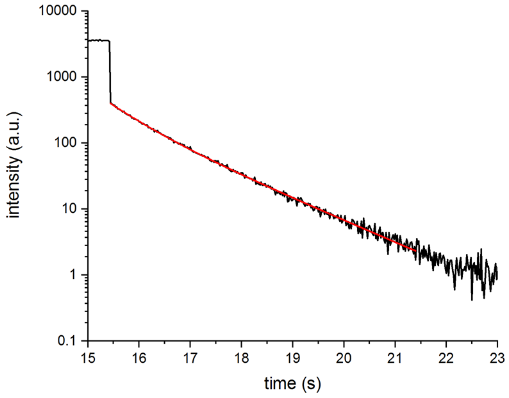

Figure S137. Phosphorescence lifetime decay curve (black line) and fitted lifetime decay (red line) of 100 ppm pyrene-d_{10} in a mixture of *p*-toluic acid (99.89 wt%) and 4-Br DBI (**2e**) (0.1 wt%). Exitation at 349 nm, emission at 593 nm. The lifetime was determined to be: τ1 = 0.61 ± 0.03 s (46 %); τ2 = 1.32 ± 0.03 s (54 %); τavg = 0.99 ± 0.03 s.

4.3.10 4-SMe DBI (2k) with phosphorescent impurities

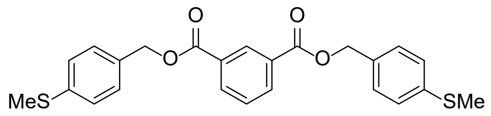

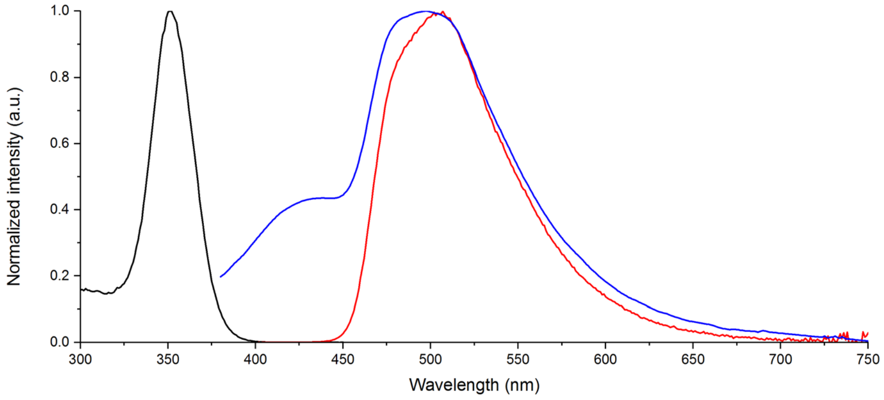

Figure S138. Luminescence of 4-SMe DBI (**2k**). Normalized phosphorescence excitation spectrum (black line; emission at 505 nm), steady-state luminescence emission spectrum (blue line) and phosphorescence spectrum (red line; excitation for both at 351 nm).

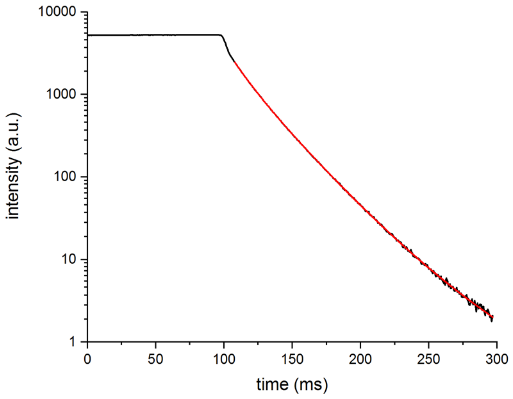

Figure S139. Phosphorescence lifetime decay curve (black line) and fitted lifetime decay (red line) of the 4-SMe DBI (**2k**). Excitation at 351 nm, emission at 505 nm. The lifetime was determined to be: τ_1 = 11.1 ± 0.3 ms (29 %); τ_2 = 24.1 ± 0.3 ms (69 %); τ_3 = 52.6 ± 3.7 ms (2 %); τ_{avg} = 20.8 ± 0.8 ms.

4.3.11 4-OMe DBI (2h) with 0.1 wt% 4-(methylthio)benzaldehyde (G1)

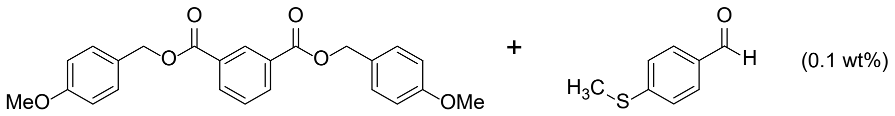

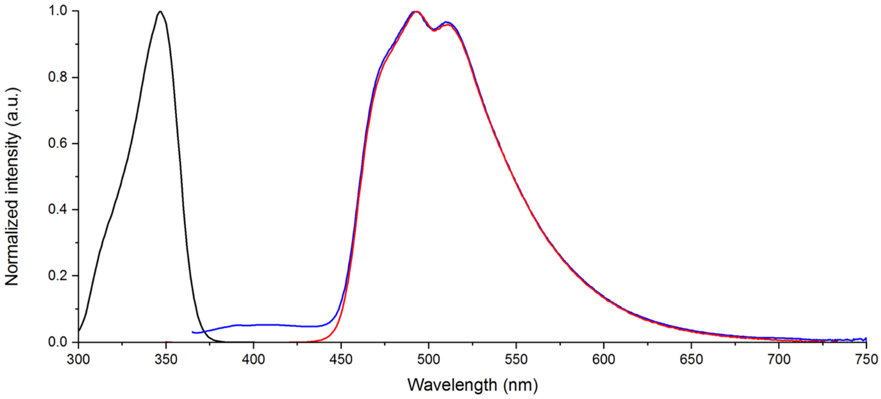

Figure S140. Luminescence of 0.1 wt% 4-(methylthio)benzaldehyde (**G1**) in 4-OMe DBI (**2h**). Normalized phosphorescence excitation spectrum (black line; emission at 491 nm), steady-state luminescence emission spectrum (blue line) and phosphorescence spectrum (red line; excitation for both at 347 nm).

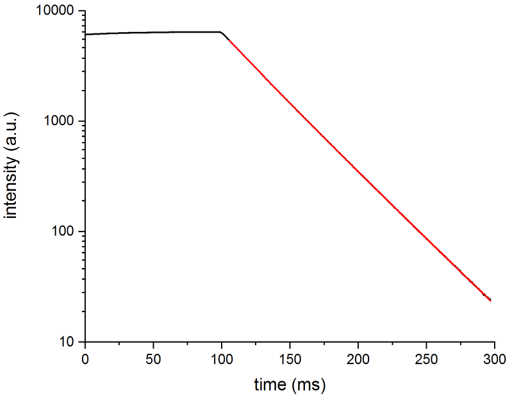

Figure S141. Phosphorescence lifetime decay curve (black line) and fitted lifetime decay (red line) of 4-(methylthio)benzaldehyde (**G1**) in 4-OMe DBI (**2h**) (0.1 wt%). Excitation at 347 nm, emission at 491 nm. The lifetime was determined to be: τ_1 = 25.9 ± 0.2 ms (23 %); τ_2 = 36.8 ± 0.1 ms (77 %); τ_{avg} = 34.3 ± 0.3 ms.

4.3.12 4-(SPh(4-Br)) DBI (2p) with phosphorescent impurities

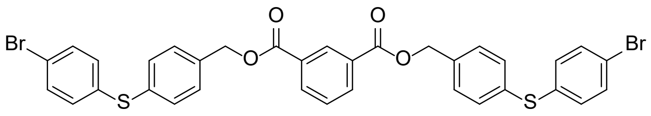

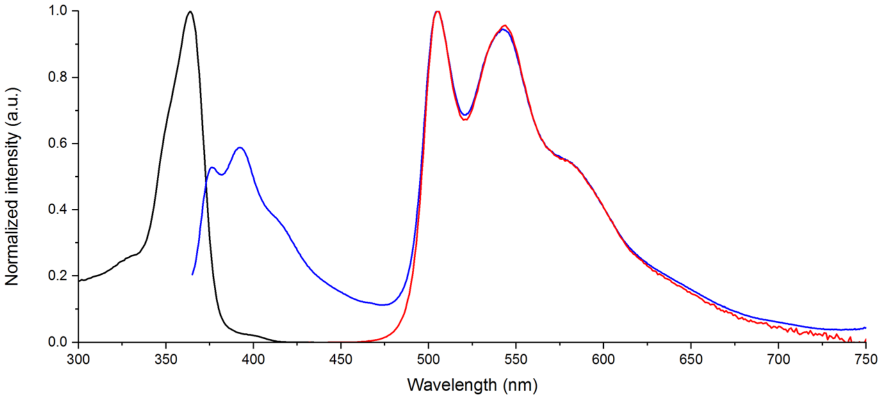

Figure S142. Luminescence of 4-(SPh(4-Br)) DBI (**2p**) Normalized phosphorescence excitation spectrum (black line; emission at 505 nm), steady-state luminescence emission spectrum (blue line) and phosphorescence spectrum (red line; excitation for both at 364 nm).

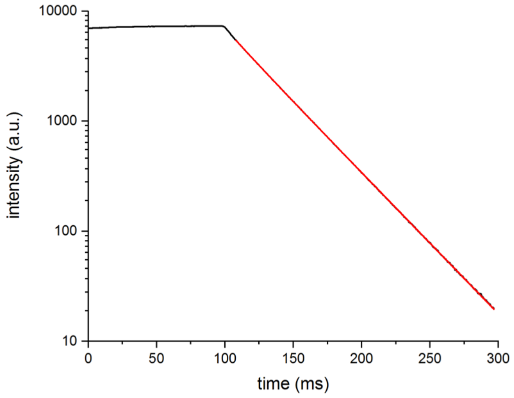

Figure S143. . Phosphorescence lifetime decay curve (black line) and fitted lifetime decay (red line) of the 4-(SPh(4-Br)) DBI (**2p**). Excitation at 364 nm, emission at 505 nm. The lifetime was determined to be: τ_1 = 15.7 ± 0.3 ms (5 %); τ_2 = 33.9 ± 0.01 ms (95 %); τ_{avg} = 32.9 ± 0.2 ms.

4.3.13 4-(SPh(4-Br)) DBI (2p) with varying amounts of 4-Br DBI (2e)

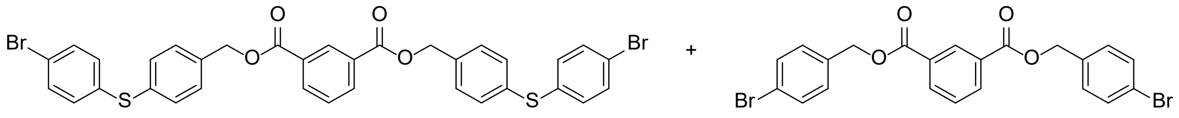

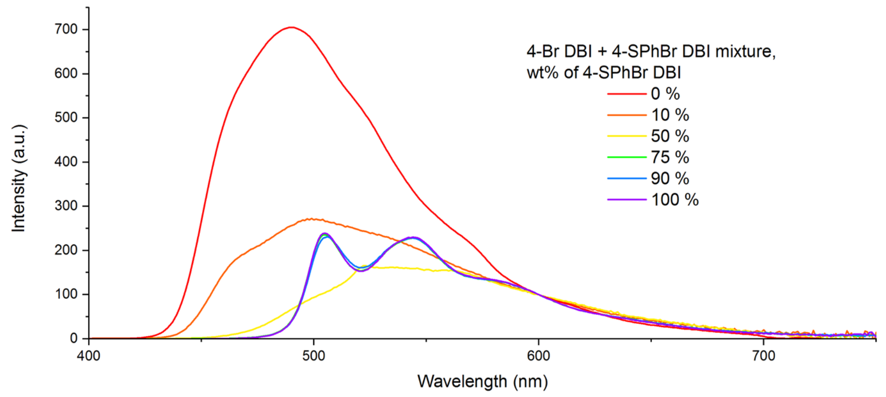

Figure S144. Phosphorescence spectra of mixtures of the 4-(SPh(4-Br)) DBI (**2p**) and the 4-Br DBI (**2e**). The sample for 0% consists of 99.9% 4-Br DBI (**2e**) and 0.1 wt% 4-((4-bromophenyl)thio)benzaldehyde (**G2**). Normalized arbitrarily at 600 nm for better visibility of the shift of the maximum. Excitation at 330 nm (0%), 348 nm (10%), 351 nm (50%), 362 nm (75%), 364 nm (90%), 364 nm (100%).

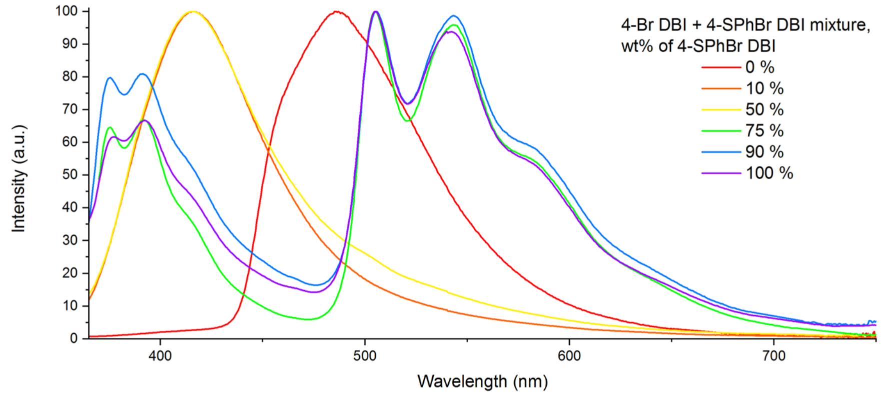

Figure S145. Steady-state emission spectra of mixtures of the 4-(SPh(4-Br)) DBI (**2p**) and the 4-Br DBI (**2e**). The sample for 0% consists of 99.9% 4-Br DBI (**2e**) and 0.1 wt% 4-((4-bromophenyl)thio)benzaldehyde (**G2**). Normalized to their respective maxima. Excitation at 330 nm (0%), 348 nm (10%), 351 nm (50%), 362 nm (75%), 364 nm (90%), 364 nm (100%).

5 Preparation of compounds

5.1 General procedures

5.1.1 General procedure 1 (GP1): Synthesis of 4'-substituted 4-formyl diphenyl ethers and sulfides

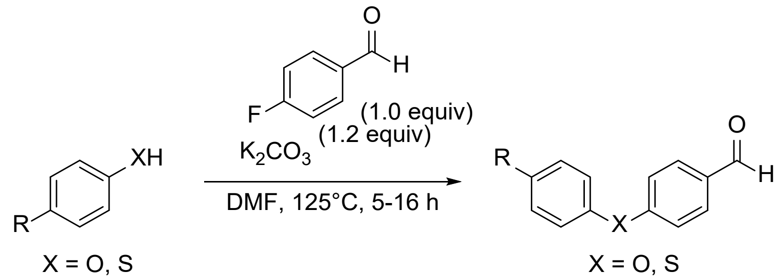

According to a modified literature procedure.[19] 4-Fluorobenzaldehyde (1.0 eq.), the corresponding thiol or phenol (1.0 eq.) and potassium carbonate (1.2 eq.) were suspended in dry DMF (0.7 M) in a pressure vial. The vial was purged with nitrogen, closed tightly and the mixture was stirred at 125°C for 5 h (thiols) or 16 h (phenols). After cooling to rt, ethyl acetate (half of the amount of DMF) was added and the mixture was filtered to remove inorganic salts. The solvents were removed from the filtrate under reduced pressure on a rotary evaporator at 75°C and then the residue was purified by Kugelrohr distillation.

5.1.2 General procedure 2 (GP2): Synthesis of benzyl alcohols

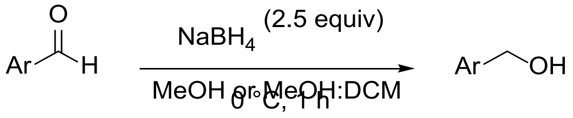

The corresponding benzaldehyde (1.0 eq.) was dissolved in methanol (0.4 M) and cooled to 0 °C. For aldehydes containing more than one aromatic ring, DCM (1 mL per mmol of aldehyde) was added to increase the solubility. $NaBH_4$ (2.5 eq.) was then added in three portions over 15 min and the mixture was stirred for 1 h at 0 °C. Afterwards, sat. aq. $NaHCO_3$ sol. was added (the same volume as methanol) and methanol was removed on a rotary evaporator. Water and ethyl acetate (3 mL per mmol of aldehyde) were added to the resulting residue. The phases were separated and the aqueous phase was extracted twice with ethyl acetate. The combined organic phases were dried over Na_2SO_4, filtered and the solvent removed under reduced

pressure. The resulting benzyl alcohol (>95% yield and purity in every case) was directly used in the esterification.

5.1.3 General procedure 3 (GP3): Esterification *via* isophthaloyl chloride

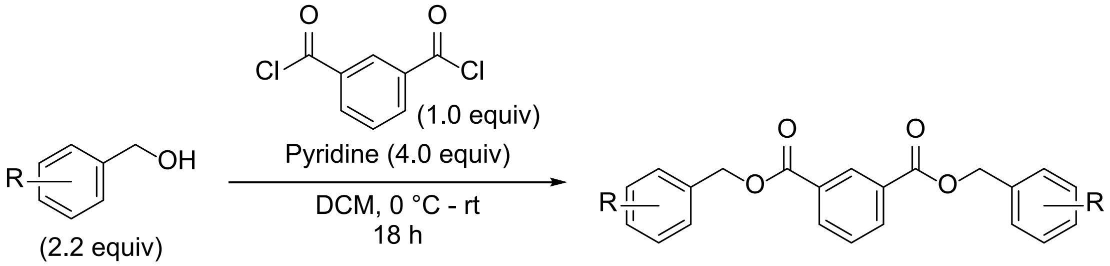

The corresponding benzyl alcohol (2.2 eq.) was dissolved in DCM (0.3 M) and pyridine (4.0 eq.) was added. The mixture was cooled using an ice bath and isophthaloyl dichloride (1.0 eq.) was added in small portions over 2 min. After 5 min, the ice bath was removed and the mixture stirred overnight at ambient temperature. Purification was carried out according to each compound.

5.2 Preparation of isophthalic acid esters

5.2.1 Preparation of dibenzyl isophthalate (2a)

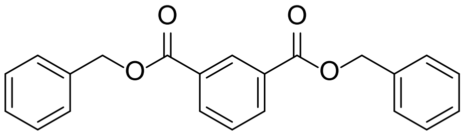

2a

The reaction was carried out according to GP3 (scale: 12.0 mmol isophthaloyl dichloride). Purification: The reaction mixture was washed with sat. $NaHCO_3$ sol. (50 mL) and brine (50 mL) and dried over Na_2SO_4. After removal of the solvent, the residue was dissolved in a boiling mixture of acetone (80 mL), MeOH (25 mL) and water (30 mL). The hot solution was cooled to rt overnight and afterwards for further 2 h at -17 °C. It was filtered, and the precipitate was washed with MeOH (50 mL). After drying, the product was obtained as a colorless solid (3.80 g, 11.0 mmol, 91 %)

^{1}H NMR (600 MHz, $CDCl_3$) δ = 8.79 (t, *J* = 1.7 Hz, 1H), 8.28 (dd, *J* = 7.8 Hz, 1.8 Hz, 2H), 7.53 (t, *J* = 7.7 Hz, 1H), 7.51 – 7.45 (m, 4H), 7.41 (tt, *J* = 8.1 Hz, 1.8 Hz, 4H), 7.39 – 7.32 (m, 2H), 5.41 (s, 4H) ppm.

^{13}C NMR (151 MHz, $CDCl_3$) δ = 165.6, 135.8, 134.1, 131.0, 130.7, 128.7, 128.5, 128.4, 67.1 ppm.

IR (ATR): $\tilde{\nu}$ (cm^{-1}): 3035, 2959, 1712, 1606, 1091, 964, 948, 755, 731, 712, 669.

MS (APCI) *m/z* = 347.2 $[M+H]^+$

Mp. 84 °C.

5.2.2 Preparation of bis(4-chlorobenzyl) isophthalate (2b)

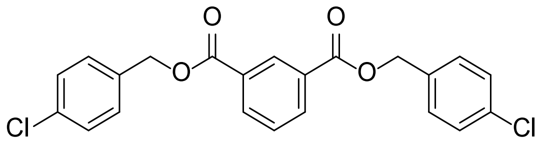

2b

The reaction was carried out according to GP3 (scale: 5.00 mmol isophthaloyl dichloride). Purification: DCM was removed under reduced pressure. The residue was suspended in a mixture of acetone (65 mL), MeOH (12 mL) and pyridine (0.5 mL) and gently heated until completely dissolved. Water (12 mL) was added, which caused gelation of the mixture. It was heated to reflux again until completely dissolved and cooled to rt over 1 h, resulting in gelation. The fragile gel was destroyed by vigorous shaking of the flask, and the resulting suspension was stored at 4 °C overnight. Afterwards, it was kept for further 2 h at –17 °C. The suspension was filtered, and the precipitate washed with a mixture of H_2O and MeOH (1:1, 100 mL) and with MeOH (100 mL). After drying, the product was obtained as a colorless solid (1.85 g, 4.45 mmol, 89%).

^1H NMR (600 MHz, $CDCl_3$) δ = 8.72 (t, *J* = 1.7 Hz, 1H), 8.25 (dd, *J* = 7.8 Hz, 1.8 Hz, 2H), 7.54 (t, *J* = 7.8 Hz, 1H), 7.40 – 7.34 (m, 8H), 5.35 (s, 4H) ppm.

^{13}C NMR (151 MHz, $CDCl_3$) δ = 165.6, 134.5, 134.3, 134.2, 131.1, 130.6, 129.9, 128.0, 129.0, 66.4 ppm.

IR (ATR): $\tilde{\nu}$ (cm^{-1}): 1723, 1496, 1374, 1319, 1240, 1160, 1096, 1074, 1019, 965, 883, 799, 725.

HR-MS (ESI): Calculated for $C_{22}H_{16}Cl_2NaO_4^+$ $[M+Na]^+$: *m/z* = 437.03179, found: *m/z* = 437.03175.

Mp. 144 °C.

5.2.3 Preparation of bis(2-bromobenzyl) isophthalate (2c)

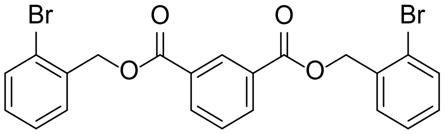

2c

The reaction was carried out according to GP3 (scale: 5.00 mmol isophthaloyl dichloride). Purification: DCM was removed under reduced pressure. MeOH (20 mL) was added to the residue, and the resulting suspension was sonicated for 5 min, then subsequently stored at – 17 °C for 1 h. The suspension was filtered, washed with MeOH (50 mL) and dried under vacuum. The product showed blue-green phosphorescence upon irradiation with a 365 nm flashlight at this point, despite being pure by NMR. Therefore, it was re-dissolved in a boiling mixture of acetone (50 mL), MeOH (12 mL), pyridine (1 mL) and H_2O (12 mL). After slowly cooling to rt overnight, the resulting suspension was stored for further 24 h at 4 °C. Afterwards, it was filtered, and the precipitate and washed with MeOH (100 mL). After drying, the product was obtained as very fine, crystalline needles (1.75 g, 3.47 mmol, 69%) that showed no phosphorescence.

^{1}H NMR (600 MHz, $CDCl_3$) δ = 8.80 (td, *J* = 1.7 Hz, 0.5 Hz, 1H), 8.29 (dd, *J* = 7.8 Hz, 1.7 Hz, 2H), 7.61 (dd, *J* = 8.0 Hz, 1.2 Hz, 2H, 7.56 (td, *J* = 7.8 Hz, 0.3 Hz, 1H), 7.51 (dd, *J* = 7.6 Hz, 1.7 Hz, 2H), 7.34 (td, *J* = 7.5 Hz, 1.2 Hz, 2H), 7.23 (td, *J* = 7.7 Hz, 1.7 Hz, 2H), 5.47 (s, 4H) ppm.

^{13}C NMR (151 MHz, $CDCl_3$) δ = 165.5, 135.2, 134.3, 133.1, 131.2, 130.6, 130.2, 130.1, 128.9, 127.7, 123.8, 66.8 ppm.

IR (ATR): $\tilde{\nu}$ (cm^{-1}): 1720, 1470, 1442, 1375, 1294, 1229, 1137, 1073, 1031, 998, 944, 907, 831, 756, 746, 728, 661, 653.

HR-MS (ESI): Calculated for $C_{22}H_{16}Br_2NaO_4^+$ [M+Na]$^+$: *m/z* = 524.93075, found: *m/z* = 524.92997.

Mp. 103-104 °C.

5.2.4 Preparation of bis(3-bromobenzyl) isophthalate (2d)

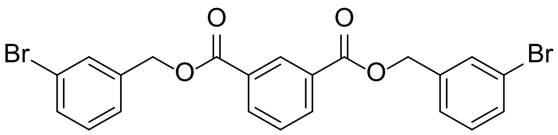

2d

The reaction was carried out according to GP3 (scale: 5.00 mmol isophthaloyl dichloride). Purification: DCM was removed under reduced pressure. Acetone (50 mL), MeOH (12 mL) and pyridine (0.5 mL) were added and the mixture was sonicated until everything was dissolved. H_2O (12 mL) was added, which caused gelation of the mixture. The gel was heated to reflux until completely dissolved. The hot solution was cooled to 4 °C overnight and afterwards for further 2 h at -17 °C. It was filtered, and the precipitate was washed with a mixture of H_2O:MeOH (1:1, 50 mL) and pure MeOH (75 mL). After drying, the product was obtained as a colorless solid (2.08 g, 4.13 mmol, 83%).

^{1}H NMR (600 MHz, $CDCl_3$) δ = 8.74 (t, *J* = 1.7 Hz, 1H), 8.27 (dd, *J* = 7.8 Hz, 1.7 Hz, 2H), 7.60 (t, *J* = 1.9 Hz, 2H), 7.56 (t, *J* = 7.8 Hz, 1H), 7.48 (ddd, *J* = 8.0 Hz, 2.0 Hz, 1.1 Hz, 2H), 7.40 – 7.37 (m, 2H), 7.26 (t, *J* = 7.8 Hz, 2H), 5.35 (s, 4H) ppm.

^{13}C NMR (151 MHz, $CDCl_3$) δ = 165.5, 138.1, 134.3, 131.6, 131.4, 131.1, 130.5, 130.4, 129.0, 127.0, 122.8, 66.2 ppm.

IR (ATR): $\tilde{\nu}$ (cm^{-1}): 1721, 1605, 1570, 1479, 1438, 1376, 1318 1288, 1241, 1138, 1095, 1071, 995, 961, 766, 724, 680, 666.

HR-MS (ESI): Calculated for $C_{22}H_{16}Br_2NaO_4^+$ $[M+Na]^+$: *m/z* = 524.93075, found: *m/z* = 524.93051.

Mp. 95 °C.

5.2.5 Preparation of bis(4-bromobenzyl) isophthalate (2e)

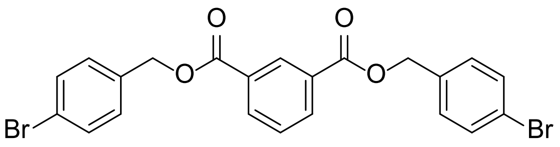

2e

The reaction was carried out according to GP3 (scale: 20.0 mmol isophthaloyl dichloride). Purification: DCM was removed under reduced pressure. The residue was dissolved in acetone (200 mL) by gentle heating. A mixture of MeOH (50 mL), H_2O (40 mL), more acetone (150 mL) and pyridine (1 mL) were added. The suspension was heated to reflux for 5 min and then slowly cooled to rt overnight. It was placed in a freezer at -17 °C for 1 h and was quickly filtered afterwards. The precipitate was washed with a mixture of H_2O/MeOH (1:1, 100 mL) and pure MeOH (100 mL). After drying, the product was obtained as a colorless solid (8.40 g, 16.7 mmol, 83 %).

^1H NMR (600 MHz, $CDCl_3$) δ = 8.72 (t, *J* = 1.6 Hz, 1H), 8.25 (dd, *J* = 7.8 Hz, 1.7 Hz, 2H), 7.54 (t, *J* = 8.3 Hz, 1H), 7.53 – 7.50 (m, 4H), 7.34 – 7.31 (m, 4H), 5.33 (s, 4H) ppm.

^{13}C NMR (151 MHz, $CDCl_3$) δ = 165.5, 134.8, 134.2, 131.9, 131.0, 130.5, 130.1, 128.9, 122.6, 66.4 ppm.

IR (ATR): $\tilde{\nu}$ (cm^{-1}): 1719, 1489, 1372, 1313, 1295, 1239, 1141, 1098, 1070, 1014, 963, 882, 795, 723, 657.

HR-MS (ESI): Calculated for $C_{22}H_{16}Br_2NaO_4^+$ $[M+Na]^+$: *m/z* = 524.93075, found: *m/z* = 524.93061.

Mp. 145-146 °C.

5.2.6 Preparation of bis(4-bromo-2-methoxybenzyl) isophthalate (2f)

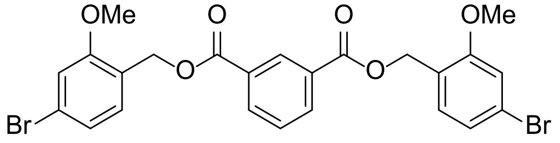

2f

The reaction was carried out according to GP3 (scale: 2.14 mmol isophthaloyl dichloride). Purification: DCM was removed under reduced pressure. MeOH (50 mL)

was added to the residue and the resulting suspension was sonicated for 5 min. It was filtered and washed with MeOH (50 mL). The residue was dried, finely powdered and suspended again in a mixture of MeOH (25 mL) and DCM (5 mL). After sonication for 5 min, the suspension was filtered and washed with MeOH (20 mL). The crude solid was further purified by column chromatography (DCM with 1% ethyl acetate) to remove a fluorescent impurity. The product was obtained as a colorless solid (830 mg, 1.47 mmol, 69%).

^{1}H NMR (600 MHz, $CDCl_3$) δ = 8.71 (s, 1H), 8.24 (d, *J* = 7.7 Hz, 2H), 7.52 (t, *J* = 7.6 Hz, 1H), 7.27 (d, *J* = 7.1 Hz, 2H)*, 7.11 (d, *J* = 8.1 Hz, 2H), 7.05 (s, 2H), 5.36 (s, 4H), 3.84 (s, 6H) ppm. *Overlaps with the solvent peak.

^{13}C NMR (151 MHz, $CDCl_3$) δ = 165.8, 158.3, 134.1, 131.1, 130.9, 130.9, 128.8, 123.7, 123.4, 123.2, 114.4, 62.1, 55.9 ppm.

IR (ATR): $\tilde{\nu}$ (cm^{-1}): 2985, 1719, 1595, 1489, 1441, 1378, 1315, 1295, 1235, 1145, 1072, 1027, 999, 979, 875, 849, 838, 796, 727.

HR-MS (EI, 70 eV): Calculated for $C_{24}H_{20}Br_2O_6^{+\bullet}$ $[M]^{+\bullet}$: *m/z* = 561.96211, found: *m/z* = 561.96308.

Mp. 135-137 °C.

5.2.7 Preparation of bis(4-iodobenzyl) isophthalate (2g)

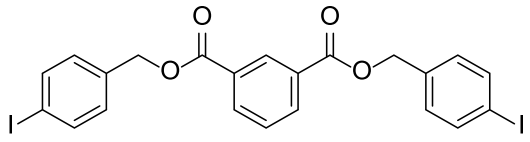

2g

The reaction was carried out according to GP3 (scale: 5.00 mmol isophthaloyl dichloride). Purification: DCM (50 mL) was added and the solution was washed with sat. $NaHCO_3$ sol. (2x 50 mL), sat. NH_4Cl sol. (50 mL) and brine (50 mL). The organic phase was dried over Na_2SO_4 and the solvent removed under reduced pressure. The residue was dissolved in a boiling mixture of acetone (60 mL), MeOH (12 mL) and H_2O (15 mL). The hot solution was cooled to rt overnight and for further 2 h at –17 °C. The resulting suspension was filtered and the precipitate washed with MeOH (100 mL). After drying, the product was obtained as a colorless solid (2.52 g, 4.66 mmol, 93%).

^{1}H NMR (601 MHz, $CDCl_3$) δ = 8.71 (t, *J* = 1.7 Hz, 1H), 8.24 (dd, *J* = 7.8 Hz, 1.8 Hz, 2H), 7.74 – 7.70 (m, 4H), 7.53 (t, *J* = 7.8 Hz, 1H), 7.21 – 7.18 (m, 4H), 5.32 (s, 4H) ppm.

^{13}C NMR (151 MHz, $CDCl_3$) δ = 165.5, 137.9, 135.5, 134.2, 131.1, 130.6, 130.3, 128.9, 94.3, 66.5 ppm.

IR (ATR): $\tilde{\nu}$ (cm^{-1}): 1717, 1484, 1370, 1313, 1296, 1237, 1143, 1097, 1072, 979, 797, 726, 656.

HR-MS (EI, 70 eV): Calculated for $C_{22}H_{16}I_2O_4^{+\bullet}$ $[M]^{+\bullet}$: *m/z* = 597.91326, found: *m/z* = 597.91356.

Mp. 133 °C.

5.2.8 Preparation of bis(4-methoxybenzyl) isophthalate (2h)

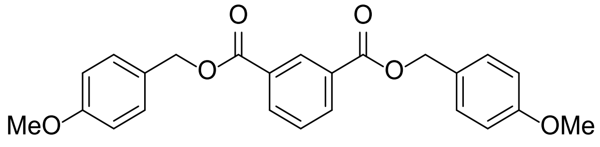

2h

The reaction was carried out according to GP3 (scale: 12.0 mmol isophthaloyl dichloride). Purification: DCM was removed under reduced pressure. The residue was dissolved in a boiling mixture of acetone (100 mL), MeOH (30 mL) and H_2O (40 mL). The hot solution was cooled to rt overnight and afterwards for further 2 h at –17 °C. It was filtered, and the precipitate was washed with MeOH (100 mL). After drying, the product was obtained as a colorless solid (4.18 g, 10.3 mmol, 86 %).

^{1}H NMR (600 MHz, $CDCl_3$) δ = 8.89 – 8.53 (m, 1H), 8.22 (dd, *J* = 7.8 Hz, 1.8 Hz, 2H), 7.50 (t, *J* = 7.5 Hz, 1H), 7.42 – 7.37 (m, 4H), 6.94 – 6.89 (m, 4H), 5.32 (s, 4H), 3.82 (s, 6H) ppm.

^{13}C NMR (151 MHz, $CDCl_3$) δ = 165.8, 159.9, 134.1, 131.0, 130.9, 130.4, 128.7, 128.0, 114.1, 67.0, 55.4 ppm.

IR (ATR): $\tilde{\nu}$ (cm^{-1}): 2960, 1726, 1609, 1584, 1514, 1449, 1366, 1305, 1240, 1226, 1178, 1085, 1029, 952, 938, 919, 851, 818, 806, 758, 725.

HR-MS (ESI): Calculated for $C_{24}H_{22}NaO_6^+$ $[M+Na]^+$: *m/z* = 429.13086, found: *m/z* = 429.13024.

Mp. 104-105 °C.

5.2.9 Preparation of bis(4-(4-methoxyphenoxy)benzyl) isophthalate (2i)

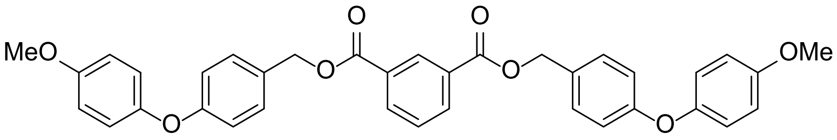

2i

The reaction was carried out according to GP3 (scale: 2.20 mmol isophthaloyl dichloride). Purification: DCM was removed under reduced pressure and the residue was dissolved in a mixture of MeOH (10 mL) and acetone (3 mL). H_2O (ca. 5 mL) was added until a precipitate formed. The suspension was cooled for 1 h in an ice bath, then quickly filtered. The precipitate was washed with MeOH (50 mL). After drying, the product was obtained as a colorless solid (890 mg, 1.43 mmol, 65%).

^{1}H NMR (600 MHz, $CDCl_3$) δ = 8.72 (s, 1H), 8.24 (d, *J* = 7.8 Hz, 2H), 7.51 (t, *J* = 7.8 Hz, 1H), 7.39 (d, *J* = 8.2 Hz, 4H), 7.00 (d, *J* = 8.7 Hz, 4H), 6.95 (d, *J* = 8.2 Hz, 4H), 6.89 (d, *J* = 8.8 Hz, 4H), 5.33 (s, 4H), 3.81 (s, 6H) ppm.

^{13}C NMR (151 MHz, $CDCl_3$) δ = 165.8, 159.0, 156.3, 149.9, 134.1, 131.1, 130.8, 130.3, 129.7, 128.7, 121.2, 117.6, 115.1, 66.9, 55.8 ppm.

IR (ATR): $\tilde{\nu}$ (cm^{-1}): 1723, 1610, 1505, 1440, 1376, 1271, 1225, 1166, 1100, 1025, 1013, 876, 839, 825, 814, 780, 722, 690.

HR-MS (EI, 70 eV): Calculated for $C_{36}H_{30}O_8^{+\bullet}$ [$M^{+\bullet}$]: *m/z* = 590.19352, found: *m/z* = 590.19418.

Mp. 81 °C.

5.2.10 Preparation of bis(4-(4-bromophenoxy)benzyl) isophthalate (2j)

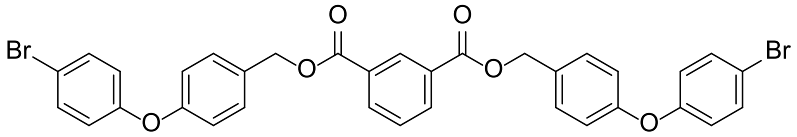

2j

The reaction was carried out according to GP3 (scale: 2.50 mmol isophthaloyl dichloride). Purification: DCM was removed under reduced pressure. The residue was

purified by column chromatography (DCM with 1 % ethyl acetate), which yielded a colorless oil (1.16 g). MeOH (1 mL) and DCM (1 mL) were added to the oil and subsequently removed again under reduced pressure (40 °C, rotary evaporator), which initiated crystallization of the compound. Full crystallization was then achieved by adding MeOH (1 mL). The compound was dried under reduced pressure, then finely powdered and suspended in MeOH (10 mL). After sonication for 10 min, the suspension was filtered and the precipitate washed with MeOH (50 mL). After drying, the product was obtained as a colorless solid (1.06 g, 1.54 mmol, 62%).

^{1}H NMR (600 MHz, $CDCl_3$) δ = 8.74 (t, *J* = 1.7 Hz, 1H), 8.26 (dd, *J* = 7.8 Hz, 1.7 Hz, 2H), 7.53 (t, *J* = 7.8 Hz, 1H), 7.46 – 7.41 (m, 8H), 7.03 – 6.98 (m, 4H), 6.93 – 6.88 (m, 4H), 5.36 (s, 4H) ppm.

^{13}C NMR (151 MHz, $CDCl_3$) δ = 165.7, 157.2, 156.3, 134.2, 132.9, 131.1, 131.1, 130.8, 130.4, 128.8, 120.8, 119.0, 116.2, 66.70 ppm.

IR (ATR): $\tilde{\nu}$ (cm^{-1}): 1716, 1612, 1578, 1508, 1482, 1376, 1315, 1303, 1238, 1142, 1093, 1070, 994, 968, 846, 817, 727.

HR-MS (ESI): Calculated for $C_{34}H_{24}Br_2NaO_6^+$ [M+Na]$^+$: *m/z* = 708.98318, found: *m/z* = 708.98196.

Mp. 93 °C.

5.2.11 Preparation of bis(4-(methylthio)benzyl) isophthalate (2k)

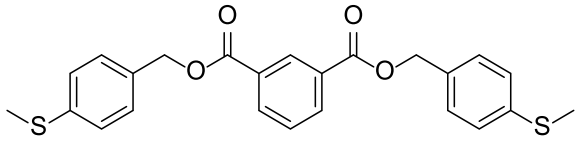

2k

The reaction was carried out according to GP3 (scale: 6.00 mmol isophthaloyl dichloride). Purification: DCM was removed under reduced pressure. MeOH (20 mL) was added to the residue, resulting in a gel-like suspension, which was sonicated for 2 min. The precipitate was filtered off and washed with a mixture of MeOH and H_2O (10:1, 50 mL), followed by pure MeOH (75 mL). After drying, the product was obtained as a colorless solid (2.40 g, 5.48 mmol, 91 %).

^{1}H NMR (600 MHz, $CDCl_3$) δ = 8.71 (t, *J* = 1.7 Hz, 1H), 8.23 (dd, *J* = 7.8 Hz, 1.8 Hz, 2H), 7.50 (t, *J* = 7.8 Hz, 1H), 7.38 – 7.35 (m, 4H), 7.28 – 7.24 (m, 4H)*, 5.32 (s, 4H), 2.48 (s, 6H) ppm. *Overlaps with the solvent peak.

^{13}C NMR (151 MHz, $CDCl_3$) δ = 165.7, 139.2, 134.1, 132.6, 131.1, 130.8, 129.2, 128.8, 126.7, 66.9, 15.9 ppm.

IR (ATR): $\tilde{\nu}$ (cm^{-1}): 2961, 1728, 1599, 1495, 1450, 1434, 1407, 1369, 1250, 1226, 1188, 1084, 956, 945, 848, 798, 734, 726, 704.

HR-MS (EI, 70 eV): Calculated for $C_{24}H_{22}O_4S_2^{+\bullet}$ $[M]^{+\bullet}$: *m/z* = 438.09540, found: *m/z* = 438.09580.

Mp. 104 °C.

2k always showed visible, short-lived, blue-green phosphorescence due to the presence of trace amounts of 4-(methylthio)benzaldehyde (**G1**). Despite our best efforts, we were unable to remove this phosphorescent impurity either by recrystallization, by washing, by distillation or by column chromatography. The given procedure therefore only shows the method that gave otherwise pure **2k** in the simplest and highest yielding way.

We identified the compound as the RTP-causing impurity by the following procedure: Freshly-distilled 4-(methylthio)benzaldehyde (**G1**, 1.0 g) was dissolved in DCM (20 mL) and stirred with a saturated, aqueous solution of $Na_2S_2O_5$ (25 mL) for 30 min. The mixture was filtered and the phases were separated. The organic phase was dried over Na_2SO_4, filtered and the solvent removed. A GC-MS analysis of the obtained mixture showed over 95 % removal of 4-(methylthio)benzaldehyde in relation to the other impurities. TLC separation of the mixture on a 20 x 2 cm TLC plate (eluent: DCM) gave well-separated spots, which were scratched off and each extracted with a 1:10 mixture of MeOH and DCM. Using these extracts, host-guest systems were prepared with the 4-Br DBI (**2e**) according to the general procedure described in this ESI. After we identified the spot that caused the RTP, we finally identified it as 4-(methylthio)benzaldehyde (**G1**) using GC-MS. The compound could be clearly separated on the TLC from its closest impurity, 4-(methylthio)acetophenone, which did not cause RTP with **2e**.

5.2.12 Preparation of bis(4-(methylsulfinyl)benzyl) isophthalate (2l)

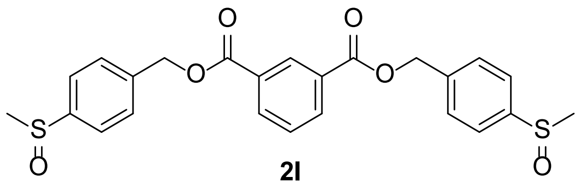

2l

According to a literature procedure.[20] Bis(4-(methylthio)benzyl) isophthalate (877 mg, 2.00 mmol, 1.0 eq.) was dissolved in HFIP (8 mL) and cooled to 0 °C. 30 % H_2O_2 (450 µL, 4.40 mmol, 2.2 eq.) was added and the mixture was stirred at 0 °C for 20 min. Ethyl acetate (5 mL), DCM (70 mL) and H_2O (25 mL) were added, and the phases were separated. The organic phase was dried over Na_2SO_4 and the solvents were removed. The residue was suspended in acetonitrile (10 mL) and kept at –17 °C for 1 h. The suspension was sonicated for 20 s and then filtered. The precipitate was washed with acetonitrile (10 mL). After drying, the product was obtained as a colorless solid (550 mg, 1.17 mmol, 58 %).

^{1}H NMR (600 MHz, $CDCl_3$) δ = 8.75 (s, 1H), 8.28 (dd, *J* = 7.9 Hz, 1.7 Hz, 2H), 7.68 (d, *J* = 8.0 Hz, 4H), 7.61 (d, *J* = 8.0 Hz, 4H), 7.56 (t, *J* = 7.8 Hz, 1H), 5.44 (s, 4H), 2.74 (s, 6H) ppm.

^{13}C NMR (151 MHz, $CDCl_3$) δ = 165.5, 146.1, 139.1, 134.4, 131.1, 130.5, 129.2, 129.0, 124.1, 66.4, 44.1 ppm.

IR (ATR): $\tilde{\nu}$ (cm^{-1}): 1719, 1610, 1372, 1258, 1168, 1086, 1045, 1007, 960, 806, 725, 679.

HR-MS (ESI): Calculated for $C_{24}H_{22}NaO_6S_2^+$ $[M+Na]^+$: *m/z* = 493.07500, found: *m/z* = 493.07469.

Mp. 139 °C.

5.2.13 Preparation of bis(4-(methylsulfonyl)benzyl) isophthalate (2m)

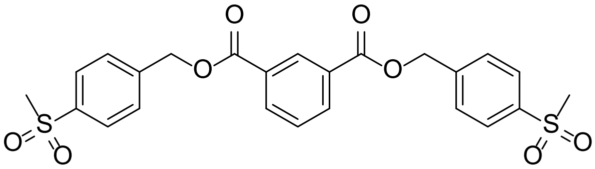

2m

Bis(4-(methylthio)benzyl) isophthalate (1.01 g, 2.30 mmol, 1.0 eq.) was dissolved in $CHCl_3$ (20 mL) and cooled with an ice bath. *m*CPBA (70%, 2.38 g, 9.66 mmol, 4.2 eq.) was added and the mixture was stirred for 16 h at ambient temperature, slowly warming up to rt overnight. Afterwards, more $CHCl_3$ (20 mL) was added and the solution was washed with sat. $NaHCO_3$ sol. (3x 50 mL) and brine (50 mL). The organic phase was dried over Na_2SO_4 and the solvent removed under reduced pressure. The residue was suspended in DCM (15 mL), sonicated for 2 min, and *n*-pentane was added (10 mL). The suspension was cooled to 4 °C for 1 h and then filtered. The precipitate was washed with *n*-pentane (25 mL). After drying, the product was obtained as a colorless solid (540 mg, 1.07 mmol, 47 %).

^{1}H NMR (600 MHz, $CDCl_3$) δ = 8.75 (s, 1H), 8.30 (dd, *J* = 7.8 Hz, 1.7 Hz, 2H), 7.98 (d, *J* = 8.2 Hz, 4H), 7.65 (d, *J* = 8.1 Hz, 4H), 7.59 (t, *J* = 7.8 Hz, 1H), 5.48 (s, 4H), 3.06 (s, 6H) ppm.

^{13}C NMR (151 MHz, $CDCl_3$) δ = 165.4, 142.0, 140.7, 134.5, 131.2, 130.4, 129.2, 128.9, 128.0, 66.0, 44.7 ppm.

IR (ATR): $\tilde{\nu}$ (cm^{-1}): 1730, 1710, 1611, 1444, 1371, 1308, 1283, 1257, 1141, 1089, 1007, 962, 823, 814, 758, 725.

HR-MS (ESI): Calculated for $C_{24}H_{22}NaO_8S_2^+$ [M+Na]$^+$: *m/z* = 525.06483, found: *m/z* = 525.06458.

Mp. 188-190 °C.

5.2.14 Preparation of bis(4-(phenylthio)benzyl) isophthalate (2n)

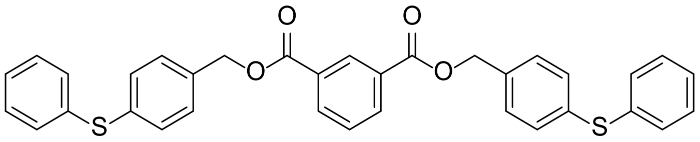

2n

The reaction was carried out according to GP3 (scale: 12.0 mmol isophthaloyl dichloride). Purification: DCM (50 mL) and 1 M HCl (50 mL) were added to the reaction mixture. The phases were separated and the organic phase was washed with sat. $NaHCO_3$ sol. (100 mL) which caused a precipitate to form. The organic phase was filtered over a very short plug of silica, which yielded a brownish oil after solvent removal. MeOH (50 mL) and H_2O (5 mL) were added to the oil, and the mixture was

left standing at rt overnight. The formed precipitate was filtered off and washed with cyclohexane (50 mL). The solid was further purified by column chromatography (DCM) to yield a colorless oil. *n*-Pentane (50 mL) was added to the oil, and the mixture was left standing at 4 °C overnight and subsequently for 1 h at rt, causing the product to precipitate. It was filtered off and washed with *n*-pentane (20 mL). After drying, the product was obtained as a colorless solid (3.40 g, 6.04 mmol, 50 %).

^{1}H NMR (601 MHz, $CDCl_3$) δ = 8.75 (t, *J* = 1.8 Hz, 1H), 8.26 (dd, *J* = 7.8 Hz, 1.8 Hz, 2H), 7.53 (t, *J* = 7.8 Hz, 1H), 7.41 – 7.37 (m, 8H), 7.35 – 7.31 (m, 8H), 7.30 – 7.26 (m, 2H), 5.36 (s, 4H) ppm.

^{13}C NMR (151 MHz, $CDCl_3$) δ = 165.6, 136.8, 135.0, 134.4, 134.2, 131.8, 131.0, 130.64, 130.61, 129.4, 129.2, 128.8, 127.6, 66.6 ppm.

IR (ATR): $\tilde{\nu}$ (cm^{-1}): 3058, 1712, 1582, 1493, 1474, 1440, 1375, 1317, 1299, 1228, 1143, 1073, 1014, 975, 807, 744, 725, 688.

HR-MS (EI, 70 eV): Calculated for $C_{34}H_{26}O_4S_2^{+\bullet}$ $[M]^{+\bullet}$: m/z = 562.12670, found: m/z = 562.12657.

Mp. 65-66 °C.

5.2.15 Preparation of bis(4-((4-(methylthio)phenyl)thio)benzyl) isophthalate (2o)

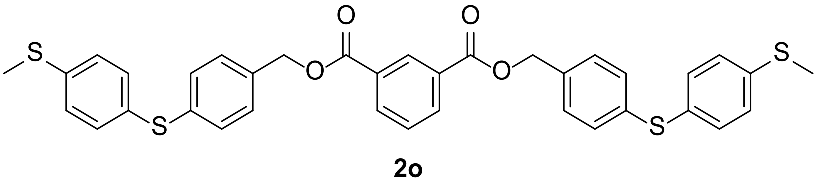

2o

The reaction was carried out according to GP3 (scale: 2.50 mmol isophthaloyl dichloride). Purification: DCM was removed under reduced pressure. Pyridine (0.5 mL), THF (5 mL) and MeOH (5 mL) were added to the residue, resulting in a clear solution. Further addition of MeOH (15 mL) lead to precipitation of the product, which was filtered off, washed with MeOH (30 mL) and dried. The product was obtained as a colorless solid (1.16 g, 1.77 mmol, 71%).

^{1}H NMR (600 MHz, $CDCl_3$) δ = 8.71 (td, *J* = 1.7 Hz, 0.4 Hz, 1H), 8.24 (dd, *J* = 7.8 Hz, 1.8 Hz, 2H), 7.52 (td, *J* = 7.8 Hz, 0.6 Hz, 1H), 7.38 – 7.30 (m, 8H), 7.30 – 7.23 (m,

4H)*, 7.23 – 7.18 (m, 4H), 5.33 (s, 4H), 2.48 (s, 6H) ppm. *Overlaps with the solvent peak.

^{13}C NMR (151 MHz, $CDCl_3$) δ = 165.7, 139.0, 137.6, 134.19, 134.17, 133.1, 131.1, 130.7, 130.6, 129.8, 129.3, 128.8, 127.3, 66.7, 15.8 ppm.

IR (ATR): $\tilde{\nu}$ (cm^{-1}): 1722, 1610, 1493, 1475, 1440, 1373, 1283, 1259, 1165, 1100, 1014, 804, 720.

HR-MS (APCI): Calculated for $C_{36}H_{31}O_4S_4^+$ $[M+H]^+$: *m/z* = 655.10997, found: *m/z* = 655.10818.

Mp. 86-89 °C.

5.2.16 Preparation of bis(4-((4-bromophenyl)thio)benzyl) isophthalate (2p)

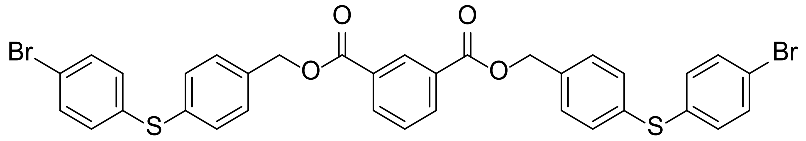

2p

The reaction was carried out according to GP3 (scale: 2.50 mmol isophthaloyl dichloride). Purification: MeOH (15 mL) was added to the reaction mixture, which had become a suspension during the reaction, and the precipitate was filtered off after stirring for 10 min at rt. The solid was washed with MeOH (50 mL) and then dried. The product was obtained as a colorless solid (1.29 g, 1.79 mmol, 72%).

^{1}H NMR (600 MHz, $CDCl_3$) δ = 8.73 (t, *J* = 1.5 Hz, 1H), 8.26 (dd, *J* = 7.8 Hz, 1.7 Hz, 2H), 7.54 (t, *J* = 7.8 Hz, 1H), 7.45 – 7.41 (m, 4H), 7.41 – 7.37 (m, 4H), 7.35 – 7.31 (m, 4H), 7.23 – 7.19 (m, 4H), 5.36 (s, 4H) ppm.

^{13}C NMR (151 MHz, $CDCl_3$) δ = 165.6, 135.9, 135.1, 134.8, 134.2, 132.9, 132.5, 131.2, 131.1, 130.7, 129.4, 128.9, 121.5, 66.6 ppm.

IR (ATR): $\tilde{\nu}$ (cm^{-1}): 1719, 1610, 1494, 1472, 1374, 1264, 1242, 1159, 1098, 1079, 1066, 995, 910, 809, 723.

HR-MS (APCI): Calculated for $C_{34}H_{25}Br_2O_4S_2^+$ $[M+H]^+$: *m/z* = 718.95555, found: *m/z* = 718.95363.

Mp. 127-128 °C.

5.2.17 Preparation of bis(4-formylbenzyl) isophthalate (2q)

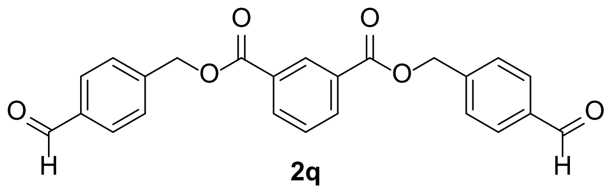

2q

The reaction was carried out according to GP3 (scale: 17.0 mmol isophthaloyl dichloride). Purification: DCM was removed under reduced pressure. The residue was dissolved in a boiling mixture of acetone (150 mL), MeOH (45 mL), H_2O (60 mL) and pyridine (2 mL). The hot solution was cooled to rt overnight, and the resulting suspension was stored at –17 °C for 2 h. The crystalline precipitate was quickly filtered off and washed with MeOH (150 mL). After drying, the product was obtained as a colorless solid (3.67 g, 9.12 mmol, 54 %).

^1H NMR (600 MHz, $CDCl_3$) δ = 10.04 (s, 2H), 8.78 (t, *J* = 1.8 Hz, 1H), 8.30 (dd, *J* = 7.8 Hz, 1.7 Hz, 2H), 7.91 (d, *J* = 8.2 Hz, 4H), 7.61 (d, *J* = 8.3 Hz, 4H), 7.58 (t, *J* = 7.8 Hz, 1H), 5.47 (s, 4H) ppm.

^{13}C NMR (151 MHz, $CDCl_3$) δ = 191.9, 165.4, 142.5, 136.4, 134.4, 131.2, 130.5, 130.2, 129.1, 128.5, 66.3 ppm.

IR (ATR): $\tilde{\nu}$ (cm^{-1}): 2767, 1716, 1684, 1607, 1452, 1444, 1378, 1277, 1253, 1212, 1165, 1097, 1009, 847, 806, 727, 708.

HR-MS (ESI): Calculated for $C_{24}H_{18}NaO_6^+$ $[M+Na]^+$: *m/z* = 425.09956, found: *m/z* = 425.09901.

Mp. 141-145 °C.

5.2.18 Preparation of bis(4-cyanobenzyl) isophthalate (2r)

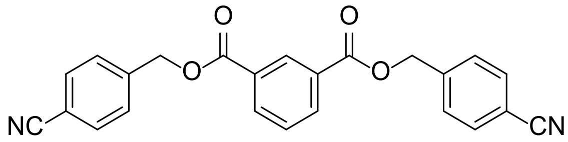

2r

The reaction was carried out according to GP3 (scale: 10.0 mmol isophthaloyl dichloride). Purification: DCM was removed under reduced pressure. The residue was dissolved in a boiling mixture of acetone (45 mL), acetonitrile (20 mL), H_2O (10 mL)

and pyridine (0.75 mL). After cooling to rt overnight, the resulting suspension was stored at –5 °C for 1 h. It was filtered, and the precipitate was washed with MeOH (60 mL). After drying, the product was obtained as a fluffy, colorless solid (2.92 g, 7.37 mmol, 74%).

^{1}H NMR (600 MHz, $CDCl_3$) δ = 8.75 (t, *J* = 1.8 Hz, 1H), 8.28 (dd, *J* = 7.8 Hz, 1.7 Hz, 2H), 7.69 (d, *J* = 8.2 Hz, 2H), 7.58 (t, *J* = 7.8 Hz, 1H), 7.55 (d, *J* = 8.0 Hz, 2H), 5.44 (s, 2H) ppm.

^{13}C NMR (151 MHz, $CDCl_3$) δ = 165.3, 141.1, 134.4, 132.7, 131.2, 130.4, 129.1, 128.6, 118.6, 112.5, 66.0 ppm.

IR (ATR): $\tilde{\nu}$ (cm^{-1}): 2227, 1720, 1613, 1509, 1441, 1376, 1315, 1304, 1290, 1232, 1143, 1097, 1073, 996, 810, 726, 623.

HR-MS (ESI): Calculated for $C_{24}H_{16}N_2NaO_4^+$ [M+Na]$^+$: *m/z* = 419.10023, found: *m/z* = 419.09981.

Mp. 177 °C.

5.2.19 Preparation of bis(4-(methoxycarbonyl)benzyl) isophthalate (2s)

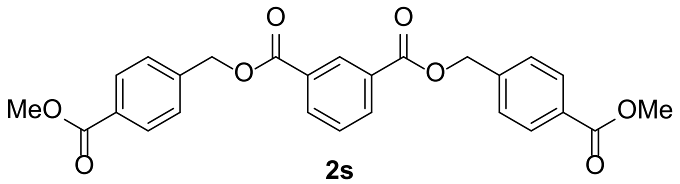

2s

GP2 for the preparation of the corresponding benzyl alcohol from the corresponding benzaldehyde was modified for this reaction. Absolute EtOH was used as the solvent, and only 0.5 eq. of $NaBH_4$ were used.

The reaction was carried out according to GP3 (scale: 10.8 mmol isophthaloyl dichloride). Purification: DCM was removed under reduced pressure. The residue was dissolved in a hot mixture of acetone (40 mL), acetonitrile (40 mL), H_2O (20 mL) and pyridine (1 mL). After cooling to rt overnight, the resulting suspension was stored at -17 °C for 1 h. It was filtered, and the precipitate was washed with MeOH (100 mL). After drying, 2.34 g of a colorless solid were obtained, which was purified by column chromatography (DCM:EtOAc:pyridine 100:5:1) to yield the product as a colorless solid (2.18 g, 4.71 mmol, 44%).

^{1}H NMR (600 MHz, $CDCl_3$) δ = 8.76 (s, 1H), 8.28 (dd, *J* = 7.8 Hz, 1.7 Hz, 2H), 8.06 (d, *J* = 8.3 Hz, 4H), 7.56 (t, *J* = 7.8 Hz, 1H), 7.51 (d, *J* = 8.1 Hz, 4H), 5.44 (s, 4H), 3.93 (s, 6H) ppm.

^{13}C NMR (151 MHz, $CDCl_3$) δ = 166.82, 165.50, 140.85, 134.33, 131.13, 130.57, 130.27, 130.10, 128.99, 127.93, 66.44, 52.34 ppm.

IR (ATR): $\tilde{\nu}$ (cm^{-1}): 1717, 1611, 1437, 1375, 1273, 1236, 1194, 1159, 1097, 1013, 966, 842, 830, 747, 728, 712, 686.

HR-MS (ESI): Calculated for $C_{26}H_{22}NaO_8^+$ $[M+Na]^+$: *m/z* = 485.12069, found: *m/z* = 485.12049.

Mp. 121-123 °C.

5.2.20 Preparation of bis(4-(trifluoromethyl)benzyl) isophthalate (2t)

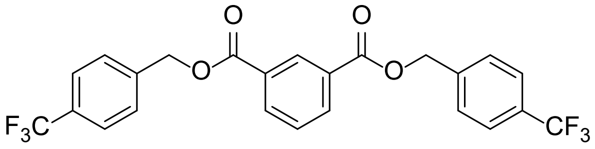

2t

The reaction was carried out according to GP3 (scale: 10.8 mmol isophthaloyl dichloride). Purification: DCM was removed under reduced pressure. The residue was dissolved in a boiling mixture of acetone (40 mL), acetonitrile (40 mL), H_2O (15 mL) and pyridine (1 mL). After cooling to rt overnight, the resulting suspension was stored at –17 °C for 30 min. The precipitate was filtered off, washed with MeOH (100 mL) and dried. The product was obtained as a colorless solid (3.92 g, 8.13 mmol, 75%).

^{1}H NMR (600 MHz, $CDCl_3$) δ = 8.76 (t, *J* = 1.8 Hz, 1H), 8.28 (dd, *J* = 7.8 Hz, 1.7 Hz, 2H), 7.65 (d, *J* = 8.1 Hz, 4H), 7.57 (dt, *J* = 7.9 Hz, 4.3 Hz, 3.4 Hz, 5H), 5.44 (s, 4H) ppm.

^{13}C NMR (151 MHz, $CDCl_3$) δ = 165.5, 139.8, 134.4, 131.1, 130.9, 130.7, 130.5, 130.4, 129.0, 128.4, 126.8, 125.83, 125.81, 125.78, 125.76, 125.1, 123.2, 121.4, 66.3 ppm.

^{19}F NMR (565 MHz, $CDCl_3$) δ = -62.7 ppm.

IR (ATR): $\tilde{\nu}$ (cm^{-1}): 1735, 1709, 1622, 1611, 1584, 1377, 1326, 1256, 1154, 1100, 1066, 1003, 820, 724, 707.

HR-MS (ESI): Calculated for $C_{24}H_{16}F_6NaO_4^+$ $[M+Na]^+$: *m/z* = 505.08450, found: *m/z* = 505.08403.

Mp. 115 °C.

5.2.21 Preparation of (((isophthaloylbis(oxy))bis(methylene))bis(4,1-phenylene))diboronic acid (2u)

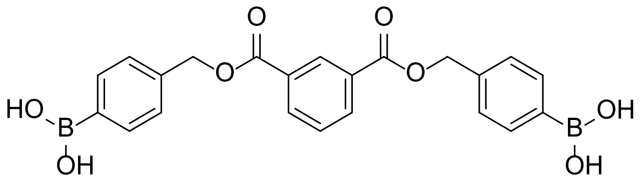

2u

Before the reaction, (4-(hydroxymethyl)phenyl)boronic acid was co-evaporated with dry pyridine (3x 20 mL) to form a tar-like residue which fully dissolved in DCM. Otherwise, the starting material would be too insoluble for the reaction.

The reaction was carried out according to a slightly modified GP3 procedure (scale: 6.60 mmol isophthaloyl dichloride): As the reaction solvent, a mixture of pyridine (20 ml) and DCM (10 mL) was used. No further pyridine was added. Purification: DCM was removed under reduced pressure. The residue was suspended in a mixture of acetone (150 mL) and a H_2O solution (150 mL) of 0.2 M $KHCO_3$ and 0.2 M KH_2PO_4. The mixture was heated to reflux until all solids dissolved and then cooled to rt. MeOH (5 mL) and DCM (5 mL) were added, and the mixture was stirred for 10 h at rt. During this time, a precipitate formed. The precipitate was filtered off and washed with H_2O (50 mL). It was re-suspended in acetone (15 mL), stirred for 5 min and filtered again. After drying, 706 mg (1.63 mmol, 26 %) of the product were obtained as a colorless solid.

^{1}H NMR (600 MHz, DMSO-d_6) δ = 8.53 (t, *J* = 1.8 Hz, 1H), 8.26 (dd, *J* = 7.8 Hz, 1.8 Hz, 2H), 8.06 (s, 4H), 7.81 (d, *J* = 8.0 Hz, 4H), 7.72 (t, *J* = 7.8 Hz, 1H), 7.43 (d, *J* = 8.0 Hz, 4H), 5.39 (s, 4H) ppm.

^{13}C NMR (151 MHz, DMSO-d_6) δ = 164.8, 137.6, 134.3, 134.1 (weak, broad signal), 133.9, 130.2, 129.8, 129.7, 126.9, 66.6 ppm.

IR (ATR): $\tilde{\nu}$ (cm^{-1}): 3370 (br), 3304 (br), 1727, 1694, 1614, 1563, 1340, 1305, 1245, 1149, 1083, 1004, 815, 796, 720.

HR-MS (ESI, MeOH): Calculated for $C_{24}H_{24}B_2NaO_8^+$ [M+2MeOH-2H$_2$O+Na]$^+$: *m/z* = 485.15495, found: *m/z* = 485.15546.

Mp. 235-240 °C.

5.2.22 Preparation of bis(4-bromobenzyl) pyridine-3,5-dicarboxylate (S1)

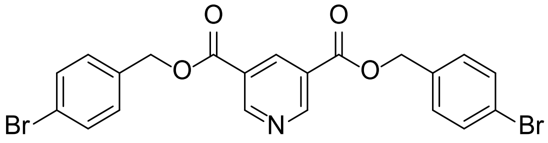

S1

Under a nitrogen atmosphere, 4-bromobenzyl alcohol (2.22 g, 12.0 mmol, 2.0 eq.), pyridine-3,5-dicarboxylic acid (1.00 g, 6.00 mmol, 1.0 eq.) and DMAP (1.61 g, 13.2 mmol, 2.2 eq.) were dissolved in DCM (35 mL) and cooled with an ice bath. EDC • HCl (2.53 g, 13.2 mmol, 2.2 eq.) was added and the ice bath was removed. The mixture was stirred for 2 d at rt. Afterwards, the solvent was removed under reduced pressure and the residue was suspended in MeOH (25 mL), H_2O (1 mL) and pyridine (0.5 mL). After sonication for 2 min, the suspension was filtered and washed with MeOH (25 mL). After drying, the product was obtained as a colorless solid (2.40 g, 4.75 mmol, 79 %).

^{1}H NMR (601 MHz, $CDCl_3$) δ = 9.37 (d, *J* = 2.1 Hz, 2H), 8.86 (t, *J* = 2.1 Hz, 1H), 7.70 – 7.43 (m, 4H), 7.43 – 7.28 (m, 4H), 5.36 (s, 4H) ppm.

^{13}C NMR (151 MHz, $CDCl_3$) δ = 164.2, 154.6, 138.3, 134.6, 132.0, 130.3, 126.0, 122.9, 66.8 ppm.

IR (ATR): $\tilde{\nu}$ (cm^{-1}): 3001, 1717, 1598, 1487, 1447, 1438, 1369, 1265, 1226, 1165, 1111, 1070, 1001, 869, 799, 746, 728, 692, 659.

HR-MS (EI, 70 eV): Calculated for $C_{21}H_{15}Br_2NO_4^{+\bullet}$ [M]$^{+\bullet}$: *m/z* = 502.93623, found: *m/z* = 502.93615.

Mp. 146-147 °C.

5.2.23 Preparation of *N*[1],*N*[3]-bis(4-bromobenzyl)isophthalamideate (S2)

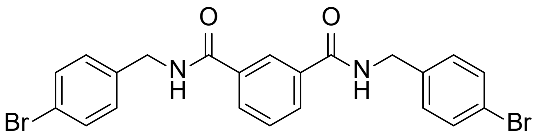

S2

4-Bromobenzylamine (1.2 mL, 11.0 mmol, 2.2 eq.) and triethylamine (2.8 mL, 20.0 mmol, 4.0 eq.) were dissolved in DCM (15 mL) and the mixture was cooled to -15 °C. A solution of Isophthaloyl dichloride (1.02 g, 5.00 mmol, 1.0 eq.) in DCM (10 mL) was added dropwise over 5 min. After complete addition, the cooling was removed and the mixture stirred at ambient temperature overnight. The solvent was removed under reduced pressure and MeOH (50 mL) and pyridine (0.5 mL) were added to the residue. The suspension was sonicated for 5 min, filtered, and the precipitate was washed with MeOH (100 mL). After drying, the product was obtained as a colorless solid (1.77 g, 3.52 mmol, 70%).

^{1}H NMR (600 MHz, DMSO-d_6) δ = 9.18 (t, *J* = 6.0 Hz, 2H), 8.39 (s, 1H), 8.03 (dd, *J* = 7.7 Hz, 1.8 Hz, 2H), 7.58 (t, *J* = 7.7 Hz, 1H), 7.52 (d, *J* = 8.1 Hz, 4H), 7.29 (d, *J* = 8.0 Hz, 4H), 4.46 (d, *J* = 5.9 Hz, 4H) ppm.

^{13}C NMR (151 MHz, DMSO-d_6) δ = 165.8, 139.0, 134.4, 131.2, 129.9, 129.6, 128.5, 126.4, 119.8, 42.2 ppm.

IR (ATR): $\tilde{\nu}$ (cm^{-1}): 3266, 1632, 1538, 1487, 1423, 1402, 1294, 1240, 1073, 1012, 980, 911, 840, 794, 716, 681.

HR-MS (EI, 70 eV): Calculated for $C_{22}H_{18}Br_2N_2O_2^{+\bullet}$ $[M]^{+\bullet}$: *m/z* = 499.97295, found: *m/z* = 499.97159.

Mp. 190 °C.

5.3 Preparation of starting materials and guests

5.3.1 Preparation of 4-(hydroxymethyl)benzaldehyde (S3)

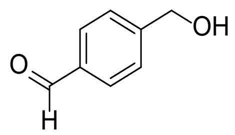

S3

According to a modified literature procedure.[21] Terephthalaldehyde (7.00 g, 52.5 mmol, 1.0 eq) was suspended in a mixture of abs. EtOH (87 mL) and THF (123 mL) and cooled with an ice bath. $NaBH_4$ (522 mg, 13.8 mmol, 0.26 eq.) was added in small portions over 30 min and the mixture was stirred for a further 6 h in the ice bath. Afterwards, brine (50 mL) and water (100 mL) were added and the organic solvents were removed under reduced pressure. The reaction mixture was extracted with DCM (3x 75 mL) and the combined organic phases were dried over Na_2SO_4. The residue was subjected to column chromatography (cyclohexane:EtOAc, 100:0 to 70:30) to give the product as a colorless solid (5.12 g, 37.6 mmol, 72 %).

^{1}H NMR (600 MHz, $CDCl_3$) δ = 9.99 (s, 1H), 7.86 (d, J = 8.1 Hz, 2H), 7.52 (d, J = 7.9 Hz, 2H), 4.80 (s, 2H), 2.00 (s, 1H)* ppm.

^{13}C NMR (151 MHz, $CDCl_3$) δ = 192.2, 147.9, 135.8, 130.2, 127.1, 64.7 ppm.

*Integral is higher in the spectrum because it contains the H_2O signal present in $CDCl_3$

IR (ATR): $\tilde{\nu}$ (cm^{-1}): 3335 (br), 2840, 2746, 1687, 1577, 1447, 1425, 1386, 1347, 1305, 1205, 1165, 1023, 1010, 848, 824, 768, 638, 615.

GC-MS (EI, 70 eV) m/z = 137 (6), 136 (65), 135 (33), 134 (3), 133 (4), 118 (4), 108 (15), 107 (100), 105 (7), 91 (5), 90 (9), 89 (23).

Mp. 40 - 42 °C.

5.3.2 Preparation of 4-(4-methoxyphenoxy)benzaldehyde (S4)

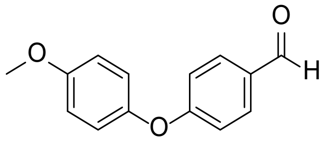

S4

The reaction was performed according to GP1 (scale: 20.0 mmol 4-fluorobenzaldehyde). Kugelrohr Distillation: 170 °C/0.20 mbar. The product was obtained as a colorless oil (3.79 g, 16.6 mmol, 83%).

^{1}H NMR (600 MHz, $CDCl_3$) δ = 9.90 (s, 1H), 7.82 (d, *J* = 7.5 Hz, 2H), 7.02 (dd, *J* = 13.2 Hz, 8.1 Hz, 4H), 6.94 (d, *J* = 8.2 Hz, 2H), 3.83 (s, 3H) ppm.

^{13}C NMR (151 MHz, $CDCl_3$) δ = 190.9, 164.3, 157.0, 148.3, 132.1, 131.0, 122.0, 116.9, 115.3, 55.8 ppm.

IR (ATR): $\tilde{\nu}$ (cm^{-1}): 3005, 2962, 2837, 2751, 1680, 1594, 1577, 1491, 1294, 1231, 1150, 1100, 1033, 829, 787, 744, 705.

GC-MS (EI, 70 eV) *m/z* = 229 (15), 228 (100), 227 (14), 214 (4), 213 (25), 157 (7), 129 (6), 128 (5), 123 (3), 114 (4), 105 (3).

Mp. 59-60 °C.

5.3.3 Preparation of 4-(4-bromophenoxy)benzaldehyde (S5)

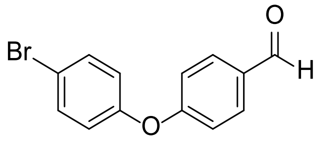

S5

The reaction was performed according to GP1 (scale: 9.35 mmol 4-fluorobenzaldehyde). Kugelrohr Distillation: 185 °C/0.30 mbar. The product was obtained as a slightly yellow oil (2.20 g, 7.94 mmol, 85%).

^{1}H NMR (600 MHz, $CDCl_3$) δ = 9.93 (s, 1H), 7.88 – 7.84 (m, 2H), 7.54 – 7.49 (m, 2H), 7.09 – 7.03 (m, 2H), 7.00 – 6.95 (m, 2H) ppm.

^{13}C NMR (151 MHz, $CDCl_3$) δ = 190.8, 162.7, 154.5, 133.3, 132.1, 131.8, 122.2, 117.9, 117.8 ppm.

IR (ATR): $\tilde{\nu}$ (cm^{-1}): 3057, 1685, 1599, 1575, 1479, 1228, 1153, 1106, 1099, 1065, 1009, 854, 870, 823, 815, 762.

GC-MS (EI, 70 eV) *m/z* = 279 (14), 278 (97), 277 (90), 276 (100), 275 (81), 221 (4), 219 (4), 169 (6), 168 (33), 141 (5), 140 (8), 139 (15), 137 (7), 115 (4).

Mp. 69-71 °C.

5.3.4 Preparation of 4-((4-(methylthio)phenyl)thio)benzaldehyde (S6)

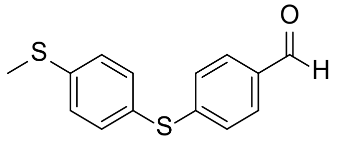

S6

The reaction was performed according to GP1 (scale: 10.0 mmol 4-fluorobenzaldehyde). Kugelrohr Distillation: 210 °C/0.30 mbar. The product was obtained as yellowish crystals (2.26 g, 8.68 mmol, 87%).

^{1}H NMR (600 MHz, $CDCl_3$) δ = 9.90 (s, 1H), 7.71 (d, *J* = 8.1 Hz, 2H), 7.44 (d, *J* = 8.3 Hz, 2H), 7.28 (d, *J* = 8.2 Hz, 2H), 7.20 (d, *J* = 8.1 Hz, 2H), 2.52 (s, 3H) ppm.

^{13}C NMR (151 MHz, $CDCl_3$) δ = 191.3, 147.8, 141.2, 135.2, 133.8, 130.3, 127.2, 126.9, 126.8, 15.4 ppm.

IR (ATR): $\tilde{\nu}$ (cm^{-1}): 2833, 2736, 1695, 1586, 1558, 1474, 1391, 1210, 1170, 1104, 1075, 1010, 813, 693.

HR-MS (APCI): Calculated for $C_{14}H_{13}OS_2^+$ $[M+H]^+$: *m/z* = 261.04023, found: *m/z* = 261.03996.

Mp. 90-92 °C.

5.3.5 Preparation of 4-((4-bromophenyl)thio)benzaldehyde (G2)

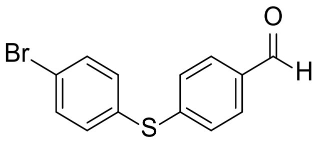

G2

The reaction was performed according to GP1 (scale: 9.35 mmol 4-fluorobenzaldehyde). Kugelrohr Distillation: 195 °C/0.25 mbar. The product was obtained as a crystalline solid (2.46 g, 8.39 mmol, 90%).

^{1}H NMR (600 MHz, $CDCl_3$) δ = 9.93 (s, 1H), 7.77 – 7.72 (m, 2H), 7.57 – 7.52 (m, 2H), 7.44 – 7.32 (m, 2H), 7.30 – 7.21 (m, 2H) ppm.

^{13}C NMR (151 MHz, $CDCl_3$) δ = 191.2, 146.2, 135.7, 134.2, 133.1, 131.0, 130.4, 127.8, 123.7 ppm.

IR (ATR): $\tilde{\nu}$ (cm^{-1}): 2834, 1694, 1587, 1562, 1385, 1300, 1209, 1166, 1070, 1006, 806, 690.

GC-MS (EI, 70 eV) *m/z* = 296 (6), 295 (17), 294 (100), 293 (51), 292 (98), 291 (37), 213 (16), 186 (5), 185 (17), 184 (62), 183 (6), 152 (11), 139 (8), 109 (4), 108 (12), 92 (6).

Mp. 68-69 °C.

5.3.6 Preparation of 2-(2-bromobenzyl)-1,3-dioxolane (S7)

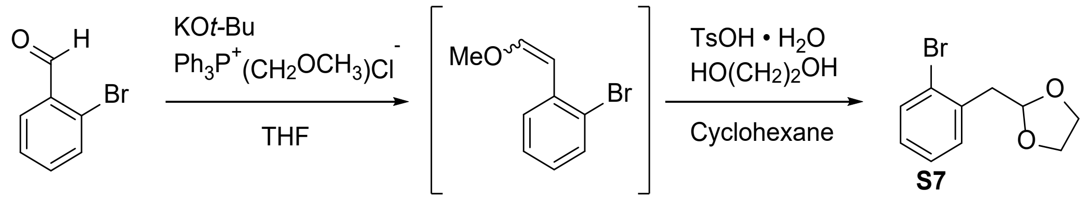

S7

According to a slightly modified literature procedure.[22] In an oven-dried Schlenk flask, (methoxymethyl)triphenyl-phosphonium chloride (11.8 g, 34.5 mmol, 1.50 eq.) was stirred in dry THF (150 mL) under a nitrogen atmosphere in an ice bath. Potassium *tert*-butoxide (7.75 g, 69.1 mmol, 3.00 eq.) was added in small portions over 5 min and the mixture was stirred at 0 °C for 30 min. 2-Bromobenzaldehyde (2.68 mL, 23.0 mmol, 1.00 eq.) was added over 5 min and stirred for 10 min at 0°C. The ice bath was removed and the mixture stirred for 3 h at ambient temperature. The reaction was quenched by the addition of sat. NH_4Cl sol. (80 mL). Water was added until all solids dissolved and the phases were separated. The aqueous phase was extracted with EtOAc (2x 50 mL). The combined organic phases were dried over Na_2SO_4 and the solvent removed under reduced pressure. Et_2O (150 mL) was added and the resulting suspension filtered and washed with further Et_2O (3x 25 mL) to remove triphenylphosphine oxide. The filtrate was concentrated to ca. 50 mL under reduced pressure, then kept at 4 °C for 1 h. The formed precipitate was filtered off and washed with cold Et_2O (3x 10 mL) to remove more triphenylphosphine oxide. The solvent of the filtrate was removed under reduced pressure, and the residue was dissolved in

cyclohexane (60 mL) in a 250 mL flask. Ethylene glycol (6.7 mL, 120 mmol, 5.2 eq.) and TsOH • H_2O (438 mg, 2.30 mmol, 10 mol%) were added and a Soxhlet extractor (50 mL volume) was placed on top of the flask. The extractor was filled with 3Å molecular sieves and cyclohexane (45 mL), and it was sealed with a balloon on top. The mixture was heated to 105 °C (oil bath temperature) for 16 h. After cooling to rt, Na_2CO_3 (1 g) was added, followed by water (100 mL). The phases were separated and the aqueous phase was extracted with cyclohexane (1x 50 mL). The combined organic phases were dried over Na_2SO_4 and the solvent was removed under reduced pressure. The resulting yellow oil was subjected to Kugelrohr distillation at 110 °C/0.28 mbar, which yielded the product (4.10 g, 17.3 mmol, 75%) as a clear oil.

^{1}H NMR (601 MHz, $CDCl_3$) δ = 7.55 (dd, *J* = 8.0 Hz, 1.3 Hz, 1H), 7.35 (dd, *J* = 7.6 Hz, 1.7 Hz, 1H), 7.26 (td, *J* = 7.6 Hz, 1.3 Hz, 1H), 7.10 (td, *J* = 7.7 Hz, 1.8 Hz, 1H), 5.17 (t, *J* = 5.0 Hz, 1H), 4.03 – 3.96 (m, 2H), 3.90 – 3.84 (m, 2H), 3.14 (d, *J* = 5.0 Hz, 2H) ppm.

^{13}C NMR (151 MHz, $CDCl_3$) δ = 136.0, 132.9, 132.0, 128.5, 127.5, 125.1, 103.4, 65.1, 40.8 ppm.

IR (ATR): $\tilde{\nu}$ (cm^{-1}): 2881, 1473, 1438, 1398, 1130, 1117, 1026, 986, 942, 821, 749, 659.

GC-MS (EI, 70 eV) *m/z* = 244 (6), 243 (10), 242 (7), 241 (9), 184 (6), 183 (6), 182 (6), 172 (8), 171 (98), 170 (9), 169 (100), 133 (6), 119 (5), 118 (5), 117 (5), 105 (17), 104 (9), 103 (22), 102 (19), 101 (7), 91 (37), 90 (76).

5.3.7 Preparation of benzo[*c*]phenanthrene (G10)

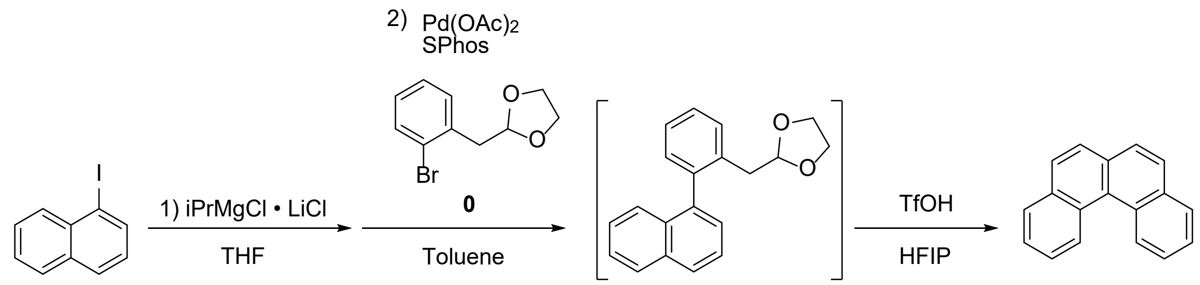

G10

According to two slightly modified literature procedures.[23,24] In an oven-dried Schlenk flask under a nitrogen atmosphere, *i*PrMgCl • LiCl (1.3 M in THF, 1.92 mL, 2.50 mmol, 1.25 eq.) was cooled to –10 °C using an acetone/ice bath. 1-Iodonaphthalene (0.35 mL, 2.40 mmol, 1.20 eq) was added over 5 min and the mixture was stirred at

– 10 °C for 30 min. In a separate, oven-dried Schlenk flask, a mixture of 2-(2-bromobenzyl)-1,3-dioxolane (486 mg, 2.00 mmol, 1.00 eq.), $Pd(OAc)_2$ (18.0 mg, 80.0 µmol, 4 mol%) and SPhos (49.0 mg, 120 µmol, 6 mol%) in dry toluene (3 mL) was stirred at rt in a water bath under a nitrogen atmosphere for 10 min. The solution containing the Grignard reagent was taken by syringe and added dropwise to the toluene mixture over 5 min. After 20 min, the reaction was quenched by the addition of MeOH (0.5 mL) and sat. $NaHCO_3$ sol. (4 mL). Et_2O (15 mL) and water (15 mL) were added, and the phases were separated. The organic phase was dried over Na_2SO_4 and the solvent removed under reduced pressure. The residue was subjected to column chromatography.[1] First, the column was eluted with cyclohexane (ca. five column volumes), then with a 10+1 mixture of cyclohexane and toluene until all nonpolar impurities were eluted. The mixture containing the coupling product was eluted with a 10+1 mixture of cyclohexane and ethyl acetate. After column chromatography, a red oil was obtained. It was dissolved in HFIP (20 mL) and cooled in an ice bath. TfOH (40 µL, 462 µmol, 23 mol%) was added and the mixture was stirred at 0 °C for 20 min. Na_2CO_3 was added (100 mg), followed by water (20 mL) and ethyl acetate (30 mL). The phases were separated and the organic phase was dried over Na_2SO_4. The residue was purified by flash column chromatography (cyclohexane + toluene 4+1) to yield the product as a colorless solid (210 mg, 813 µmol, 41%).

[1]The desired coupling product was inseparable by TLC from some impurities, but these impurities did not react in the next step and could then be easily separated from the final product. The column chromatography was done to remove all impurities which could later not be removed from the final product by column chromatography (mainly naphthalene and dimerized naphthalene).

^{1}H NMR (600 MHz, $CDCl_3$) δ = 9.17 (d, J = 8.5 Hz, 1H), 8.05 (d, J = 7.9 Hz, 1H), 7.92 (d, J = 8.4 Hz, 1H), 7.85 (d, J = 8.5 Hz, 1H), 7.71 (ddd, J = 8.4 Hz, 6.8 Hz, 1.5 Hz, 1H), 7.65 (t, J = 7.6 Hz, 7.1 Hz, 1H) ppm.

^{13}C NMR (151 MHz, $CDCl_3$) δ = 133.6, 131.1, 130.5, 128.7, 128.0, 127.6, 127.5, 127.0, 126.3, 126.0 ppm.

IR (ATR): $\tilde{\nu}$ (cm^{-1}): 3042, 1902, 1599, 1495, 1418, 1362, 1227, 1129, 1030, 945, 866, 830, 806, 740, 667.

GC-MS (EI, 70 eV) m/z = 229 (19), 228 (100), 227 (52), 226 (51), 225 (10), 224 (11), 200 (3), 114 (8), 113 (25), 112 (9), 101 (5), 100 (4).

Mp. 66-67 °C.

5.3.8 Preparation of pyrene-4,5-dione (S8)

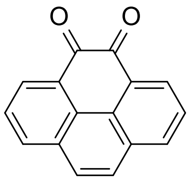

S8

According to a slightly modified literature procedure.[25] Pyrene (7.50 g, 37.2 mmol, 1.0 eq.) was dissolved in a mixture of DCM (150 mL) and THF (150 mL). $RuCl_3 \cdot 3\,H_2O$ (969 mg, 3.72 mmol, 10 mol%) was added, followed by the dropwise addition of a solution of $NaIO_4$ (35.7 g, 167 mmol, 4.5 eq.) in H_2O (190 mL) over 20 min. The mixture was vigorously stirred for 2.5 h at rt. Volatiles were removed under reduced pressure and the residue was suspended in water (250 mL) and DCM (100 mL). The aqueous phase was extracted with DCM (4x 50 mL) and the combined organic phases were washed with brine (100 mL). After drying over Na_2SO_4 and filtering, the solvent was removed and the residue was purified by column chromatography (DCM). The resulting solid (3.5 g) was suspended in ethyl acetate (75 mL) and heated to reflux. While still hot, cyclohexane (50 mL) was added and the suspension was cooled to 4 °C for 3 h. Afterwards the suspension was filtered and the solid washed with ethyl acetate (15 mL). After drying, the product was obtained as an orange solid (3.37 g, 14.5 mmol, 39%).

^1H NMR (601 MHz, $CDCl_3$) δ = 8.42 (d, *J* = 7.4 Hz, 2H), 8.12 (d, *J* = 7.9 Hz, 2H), 7.79 (d, *J* = 0.9 Hz, 2H), 7.71 (t, *J* = 7.6 Hz, 2H) ppm.

^{13}C NMR (151 MHz, $CDCl_3$) δ = 180.5, 135.9, 132.1, 130.3, 130.2, 128.5, 128.1, 127.4 ppm.

IR (ATR): $\tilde{\nu}$ (cm^{-1}): 1665, 1613, 1519, 1351, 1265, 1172, 1064, 1030, 1002, 887, 837, 772, 704.

GC-MS (EI, 70 eV) *m/z* = 235 (5), 234 (28), 233 (14), 232 (53), 205 (20), 204 (100), 177 (8), 176 (39), 175 (13), 174 (11), 150 (11), 149 (4), 116 (4).

Mp. 304 °C.

5.3.9 Preparation of 5*H*-naphtho[8,1,2-*cde*]chromen-5-one (G12)

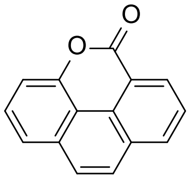

G12

According to two modified literature procedures.[26,27] Pyrene-4,5-dione (50.0 mg, 215 µmol, 1.0 eq.) was dissolved in DMF (650 µL). $Pb(OAc)_4$ (115 mg, 258 µmol, 1.2 eq.) and Oxone® (200 mg, 325 µmol, 1.5 eq. [= 3.0 eq. $KHSO_5$]) were added, and the resulting suspension was stirred for 16 h at rt. Additional Oxone® (80 mg, 130 µmol, 0.6 eq.) and $Pb(OAc)_4$ (250 mg, 564 µmol, 2.6 eq.) were added and the mixture was heated to 50 °C for 2 h. After cooling to rt, the reaction mixture was diluted with DCM (50 mL) and washed with sat. NH_4Cl sol. (2x 30 mL), sat. $NaHCO_3$ sol. (30 mL) and brine (30 mL). The aqueous phase was extracted with DCM (1x 20 mL) and the combined organic phases were dried over Na_2SO_4. After removal of the solvent, the residue was purified by column chromatography (DCM). The product was obtained as a colorless solid (18.0 mg, 81.7 µmol, 38%).

^{1}H NMR (600 MHz, $CDCl_3$) δ = 8.62 (dd, *J* = 7.6 Hz, 1.1 Hz, 1H), 8.28 (dd, *J* = 7.8 Hz, 1.1 Hz, 1H), 7.94 – 7.87 (m, 3H), 7.83 – 7.75 (m, 2H), 7.57 (dd, *J* = 7.3 Hz, 1.5 Hz, 1H) ppm.

^{13}C NMR (151 MHz, $CDCl_3$) δ = 161.6, 150.6, 133.0, 131.5, 129.9, 128.9, 128.8, 127.9, 127.7, 127.3, 126.6, 122.8, 120.0, 114.2, 113.3 ppm.

IR (ATR): $\tilde{\nu}$ (cm^{-1}): 1729, 1629, 1590, 1432, 1277, 1235, 1116, 1073, 1084, 838, 823, 730, 698.

GC-MS (EI, 70 eV) *m/z* = 221 (17), 220 (100), 192 (15), 164 (8), 163 (23), 162 (3), 110 (3), 96 (4).

Mp. 200 °C.

5.3.10 Preparation of triphenylene-1-carboxylic acid (S9)

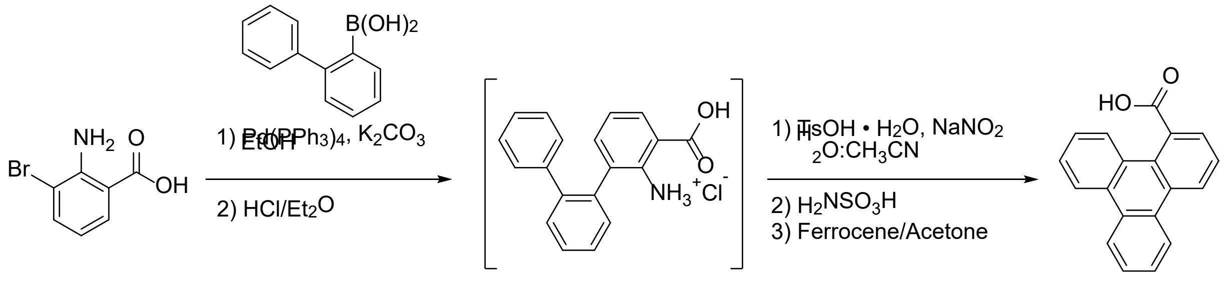

S9

According to two slightly modified literature procedures.[28,29] In a 75 mL pressure vessel, 2-amino-3-bromobenzoic acid (900 mg, 4.17 mmol, 1.0 eq.), 2-biphenylboronic acid (990 mg, 5.00 mmol, 1.2 eq.), K_2CO_3 (1.38 g, 10.0 mmol, 2.4 eq.) and $Pd(PPh_3)_4$ (72 mg, 63 µmol, 1.5 mol%) were suspended in abs. EtOH (15 mL). Argon was bubbled through the mixture for 10 min before the vessel was closed tightly and put into a pre-heated oil bath (100 °C). It was stirred for 18 h at this temperature. After cooling to rt, EtOAc (50 mL) was added and the mixture was filtered over Celite. The Celite was rinsed with more EtOAc (20 mL) and the filtrate was reduced to approximately 3 mL. DCM (5 mL) was added and the mixture was sonicated until completely dissolved. 1 M HCl in Et_2O (20 mL, 20 mmol, 4.8 eq.) and Et_2O (30 mL) were added and the resulting suspension was filtered. The precipitate was washed with Et_2O (20 mL), EtOAc (25 mL) and *n*-pentane (20 mL) to give a pale brownish solid. This solid was dissolved in a mixture of acetonitrile (13 mL) and H_2O (3 mL), TsOH • H_2O (1.59 g, 8.34 mmol, 2.0 eq.) was added and the mixture was cooled to 0 °C. A solution of $NaNO_2$ (432 mg, 6.26 mmol) in H_2O (2 mL) was added over 5 min and stirring continued for further 15 min at 0 °C. Excess nitrite was quenched by careful addition of H_2NSO_3H (284 mg, 2.92 mmol, 0.7 eq.) in H_2O (2 mL) over 5 min. This was followed by N_2 bubbling (degassing for the next step) for 5 min. The solution was taken up by syringe and added dropwise over 15 min to a stirred, degassed solution of ferrocene (233 mg, 1.25 mmol, 20 mol%) in acetone (13 mL) at rt. After complete addition, the mixture was stirred under air for 15 min, then poured into H_2O (130 mL) and stirred for further 5 min at rt. The suspension was filtered and the solid washed with H_2O (100 mL) and MeOH (100 mL). After drying, the product was obtained as an off-white solid (470 mg, 1.73 mmol, 41%).

^{1}H NMR (601 MHz, DMSO-*d*$_6$) δ = 13.44 (s, 1H), 8.93 (d, *J* = 8.1 Hz, 1H), 8.81 (d, *J* = 6.7 Hz, 3H), 8.35 (d, *J* = 8.2 Hz, 1H), 7.84 – 7.69 (m, 5H), 7.63 (t, *J* = 7.6 Hz, 1H) ppm.

^{13}C NMR (151 MHz, DMSO-d_6) δ = 173.3, 133.3, 130.9, 130.6, 129.9, 129.2, 128.7, 128.47, 128.46, 128.41, 128.3, 127.8, 127.4, 127.1, 127.0, 125.6, 124.4, 124.2, 124.0 ppm.

IR (ATR): $\tilde{\nu}$ (cm^{-1}): 2632 (br), 1672, 1489, 1420, 1396, 1303, 1281, 1262, 1211, 1160, 1132, 910, 818, 785, 737, 727, 704.

HR-MS (EI, 70 eV): calculated for $C_{19}H_{12}O_2^{+\bullet}$ $[M]^{+\bullet}$: *m/z* = 272.08318, found: *m/z* = 272.08285.

Mp. 242-245 °C.

5.3.11 Preparation of 5*H*-phenanthro[1,10,9-*cde*]chromen-5-one (G13)

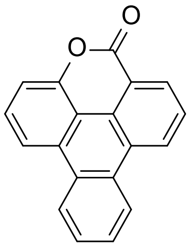

G13

According to a slightly modified literature procedure.[30] Triphenylene-1-carboxylic acid (250 mg, 918 µmol, 1.0 eq.). $Cu(OAc)_2 \cdot H_2O$ (9.2 mg, 46 µmol, 5 mol%) and dibenzoyl peroxide (75%, 371 mg, 1.15 mmol, 1.25 eq.) were suspended in HFIP (10 mL) in a 100 mL flask with an attached reflux condenser. The mixture was degassed by argon bubbling for 10 min and then put into a pre-heated oil bath (75 °C). The mixture was stirred for 24 h at 75 °C under an argon atmosphere. After cooling to rt, DCM (50 mL) was added to the mixture and the organic phase was washed with sat. NH_4Cl sol. (50 mL), sat. $NaHCO_3$ sol. (50 mL) and brine (50 mL). The organic phase was dried over Na_2SO_4, filtered and the solvent removed under reduced pressure. The residue was purified by column chromatography (DCM) to yield the product as a slightly orange solid (75 mg, 277 µmol, 30%).

Despite being pure by NMR, the product was further purified to remove colorful impurities. It was slowly crystallized by evaporation from a DCM/acetone solvent mixture in a crystallizing dish. By this method, the colorful impurities crystallized at the edges of the dish, while the product could be collected as very fine, colorless needles from the middle.

^1H NMR (600 MHz, $CDCl_3$) δ = 8.78 (d, *J* = 8.1 Hz, 1H), 8.56 – 8.48 (m, 3H), 8.30 (d, *J* = 8.1 Hz, 1H), 7.85 (t, *J* = 7.8 Hz, 1H), 7.74 (t, *J* = 8.0 Hz, 1H), 7.68 (dt, *J* = 7.6 Hz, 3.8 Hz, 2H), 7.48 (d, *J* = 7.9 Hz, 1H) ppm.

^{13}C NMR (151 MHz, $CDCl_3$) δ = 161.2, 150.6, 129.8, 129.1, 129.0, 128.6, 128.30, 128.24, 128.19, 128.11, 127.9, 127.3, 123.8, 123.4, 120.1, 118.0, 114.2, 112.9 ppm.

IR (ATR): $\tilde{\nu}$ (cm^{-1}): 1725, 1591, 1433, 1412, 1324, 1271, 1226, 1145, 1104, 1080, 1054, 1027, 1008, 946, 866, 803, 762, 745, 722.

HR-MS (EI, 70 eV): calculated for $C_{19}H_{10}O_2^{+\bullet}$ $[M]^{+\bullet}$: *m/z* = 270.06753, found: *m/z* = 270.06756.

Mp. 244-246 °C.

5.3.12 Preparation of benzo[*ghi*]perylene (G15)

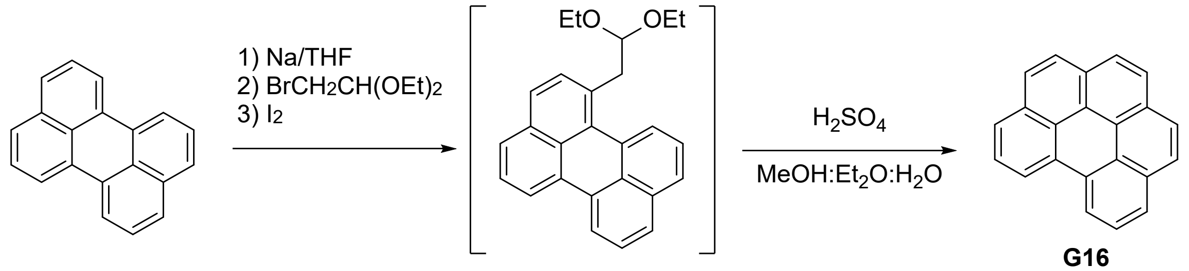

G16

According to a slightly modified literature procedure.[31] In an oven-dried Schlenk flask, perylene (2.52 g, 10.0 mmol, 1.0 eq.) was dissolved in dry THF (100 mL) under a nitrogen atmosphere. The flask was placed in a supersonic bath and cooled to 0 °C. Sodium (506 mg, 22.0 mmol, 2.2 eq.) was added in small bits over 10 min while the mixture was continuously sonicated. The mixture was sonicated for 3.5 h at a temperature between 0 °C – 20 °C.. Afterwards, the mixture was cooled to –78 °C and 2-bromo-1,1-diethoxyethane (1.50 mL, 10.0 mmol, 1.0 eq.) was added over 2 min. The mixture was stirred for 45 min at –78 °C and for 15 min at rt in a water bath before being cooled to –10 °C in an acetone/ice bath. Iodine (3.81 g, 15.0 mmol, 1.5 eq.) was added and the mixture was stirred for 5 min at –10 °C. Sodium carbonate (2.5 g) was added and the mixture was stirred at rt for 5 min. Sat. $NaHCO_3$ sol. (20 mL) was added and the mixture was filtered and washed with Et_2O (75 mL) and water (15 mL). The filtrate was moved into a separating funnel and a freshly prepared sat. $Na_2S_2O_5$ solution was added until as noticeable discoloration set in, to remove excess iodine.

The phases were separated and the aqueous phase was extracted with Et_2O (1x 30 mL). The combined organic phases were dried over Na_2SO_4 and the solvent was removed under reduced pressure. The residue was suspended in Et_2O (30 mL) and filtered to remove some of the unreacted perylene starting material. The intermediate acetal was purified by column chromatography (cyclohexane:toluene:pyridine, 1+1+0.02) and gave a mixture of acetal isomers as a red oil (1.70 g). This mixture was dissolved in a mixture of MeOH (20 mL), Et_2O (25 mL) and water (0.5 mL). Concentrated sulfuric acid (0.8 mL) was added and the mixture was stirred for 21 h at rt. The precipitated solid was filtered off, washed with water (100 mL), MeOH (60 mL) and Et_2O (10 mL) and yielded 1.25 g of the crude product. The crude product was subjected to column chromatography (cyclohexane + toluene 1+1) to yield 340 mg (1.23 mmol, 12%) of the product as a yellow solid.

^{1}H NMR (600 MHz, $CDCl_3$) δ = 9.02 (d, *J* = 7.7 Hz, 1H), 8.36 (s, 1H), 8.20 (d, *J* = 7.6 Hz, 1H), 8.14 (d, *J* = 8.7 Hz, 1H), 8.10 (d, *J* = 8.7 Hz, 1H), 8.03 (t, *J* = 7.7 Hz, 1H) ppm.

^{13}C NMR (151 MHz, $CDCl_3$) δ = 132.4, 130.6, 129.4, 127.6, 126.7, 126.4, 125.9, 125.8, 124.1, 120.9 ppm.

IR (ATR): $\tilde{\nu}$ (cm^{-1}): 3044, 2921, 1612, 1510, 1390, 1337, 1304, 1259, 1202, 1144, 1128, 963, 894, 842, 809, 765, 750.

MS (EI, 70 eV) *m/z* = 278 (3), 277 (23), 276 (100), 275 (12), 274 (23), 273 (5), 272 (7), 139 (5), 138 (20), 138 (6), 137 (17), 137 (4), 136 (8), 125 (4), 124 (4), 44 (7).

Mp. 274-276 °C.

6 NMR spectra

6.1 NMR spectra of compounds

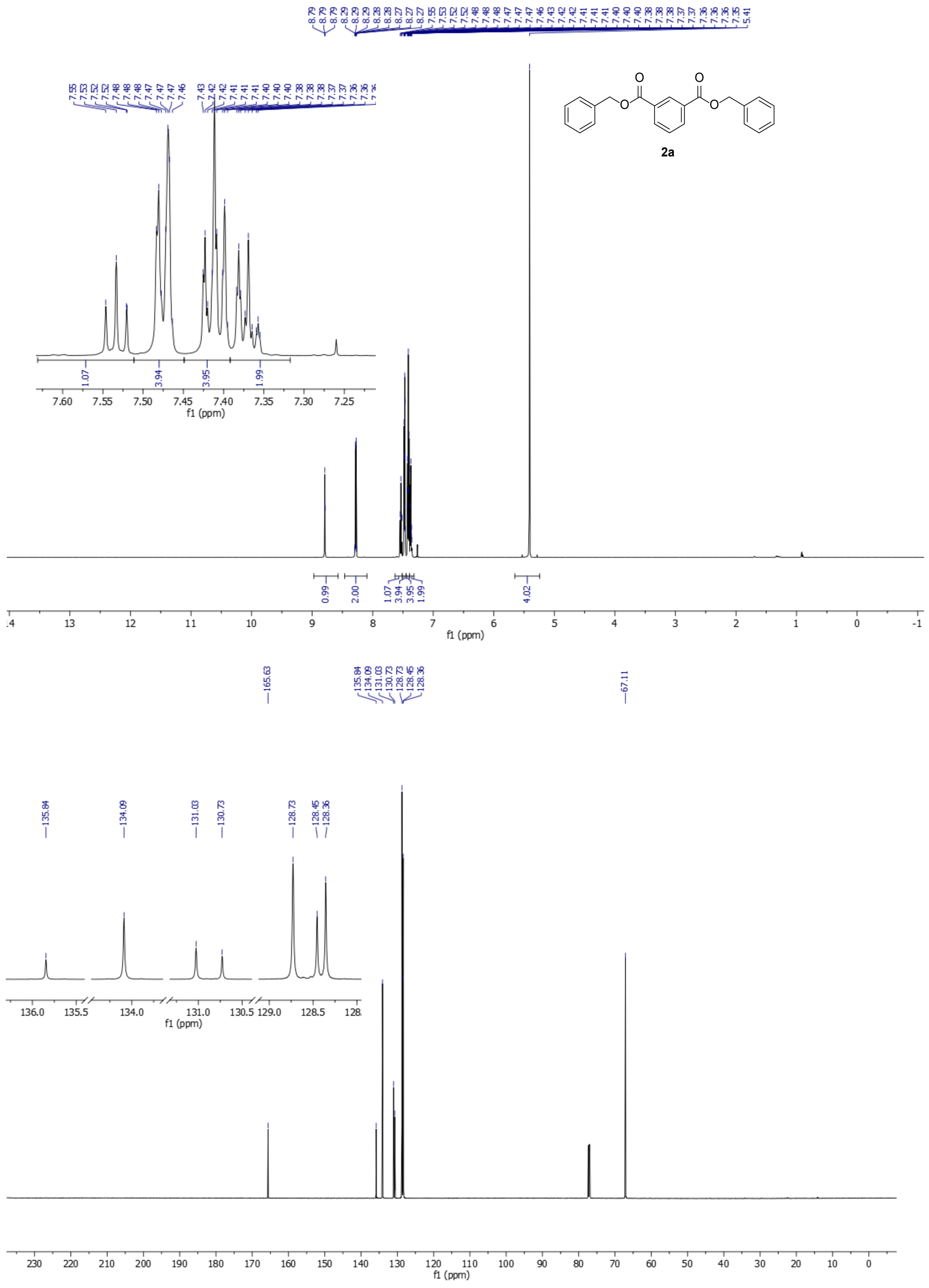

Figure S146. ^{1}H- and ^{13}C-NMR spectra of **dibenzyl isophthalate (2a)** in $CDCl_3$.

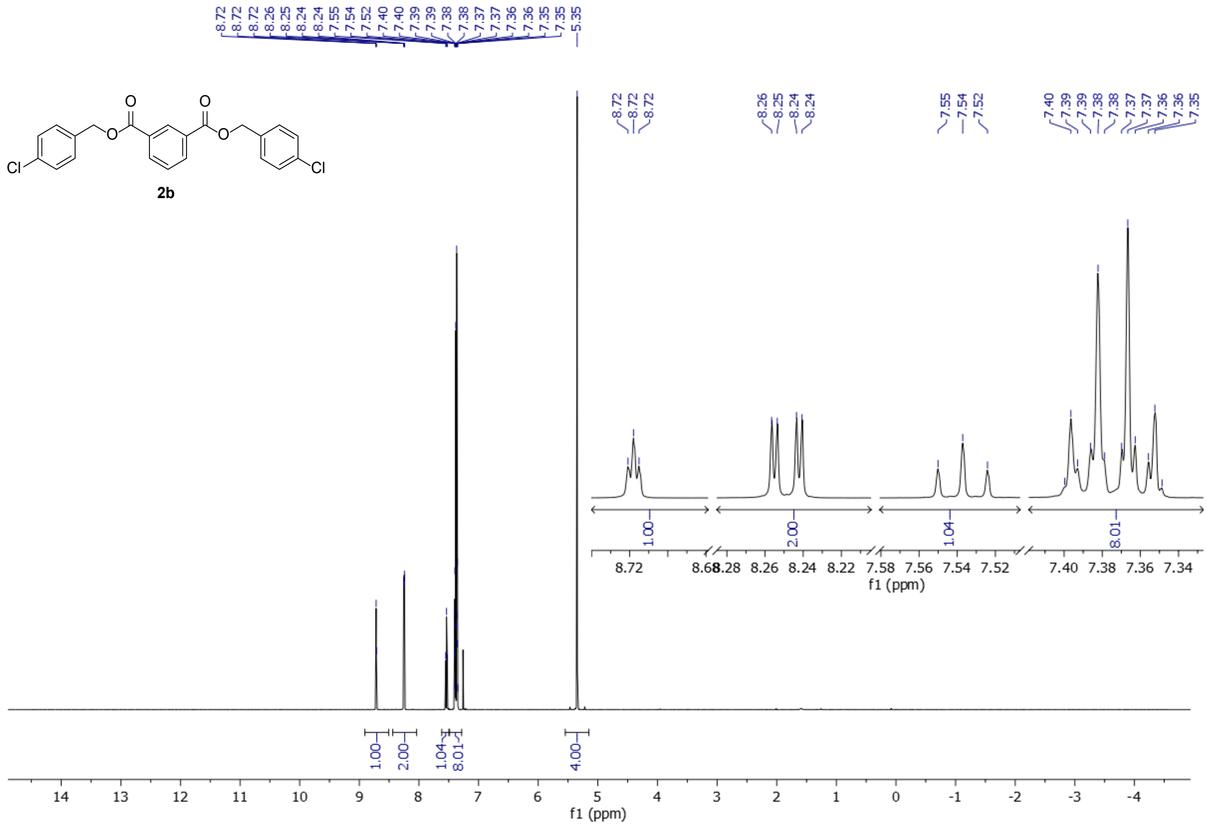

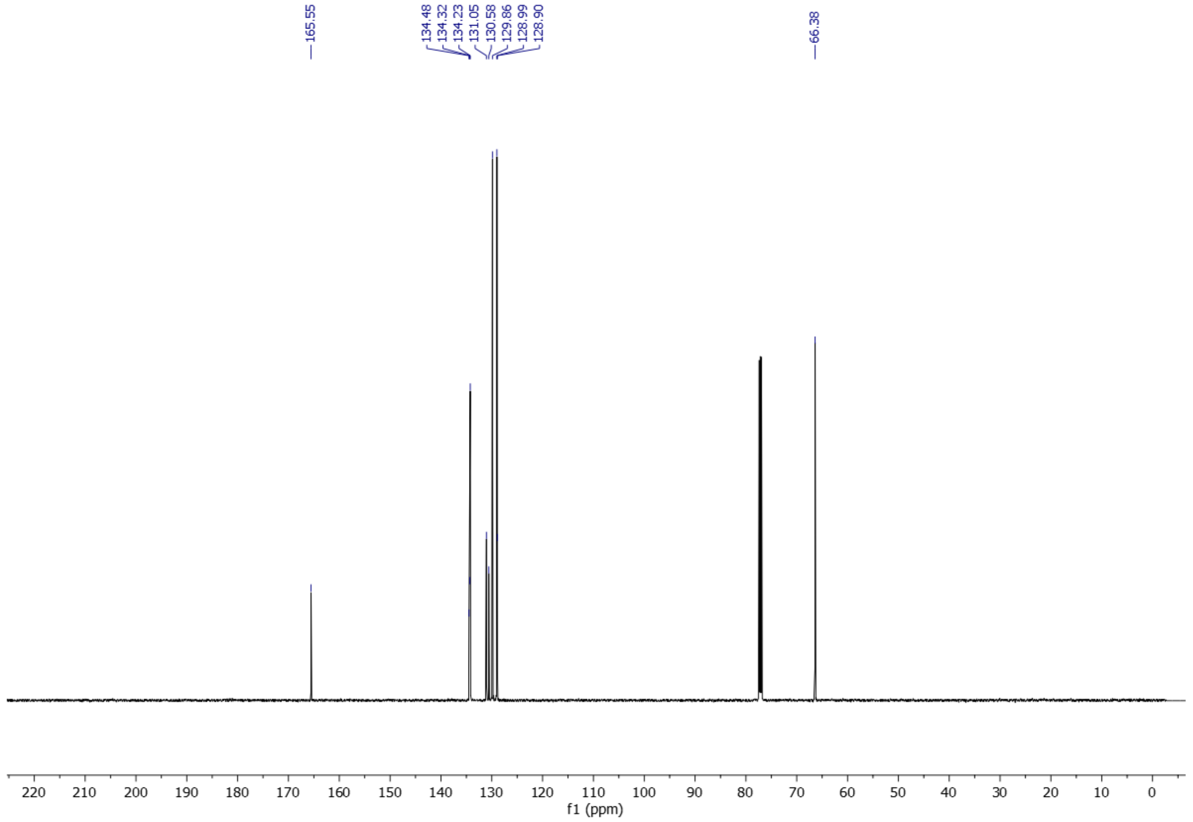

Figure S147. ^{1}H- and ^{13}C-NMR spectra of **bis(4-chlorobenzyl) isophthalate (2b)** in $CDCl_3$.

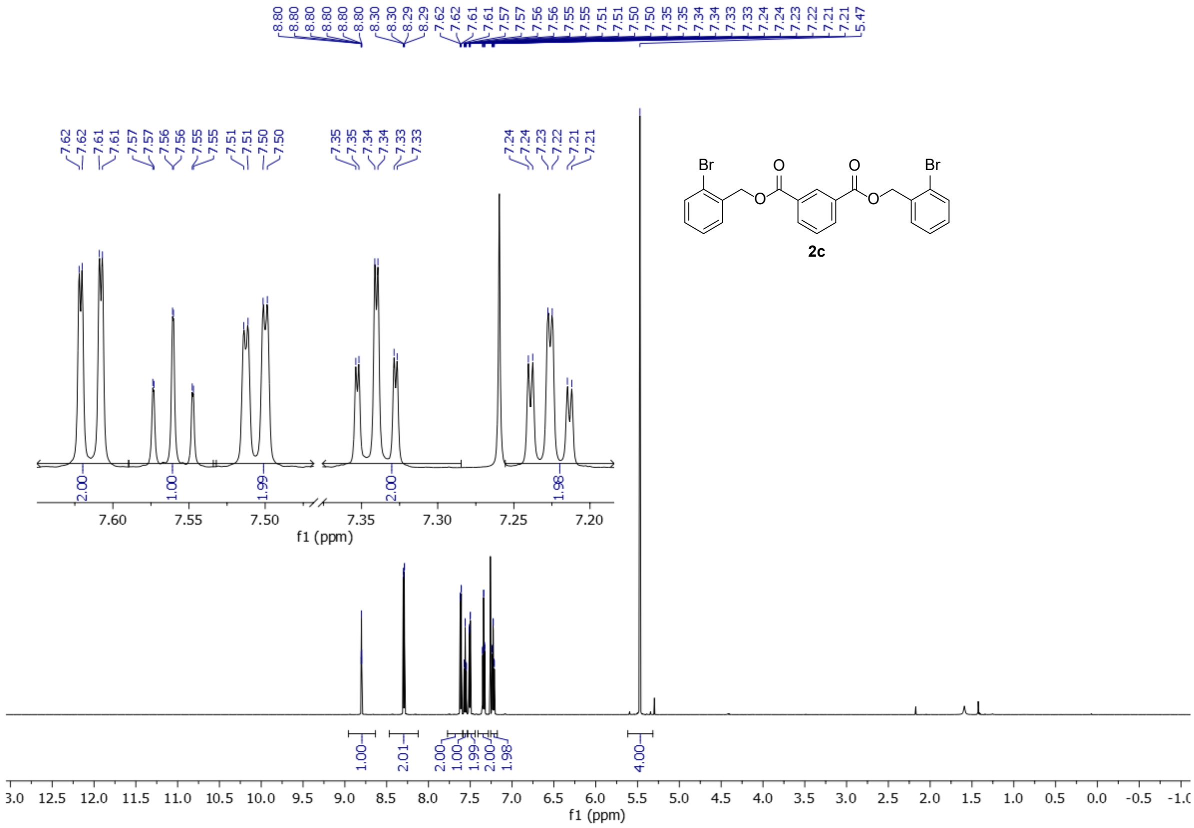

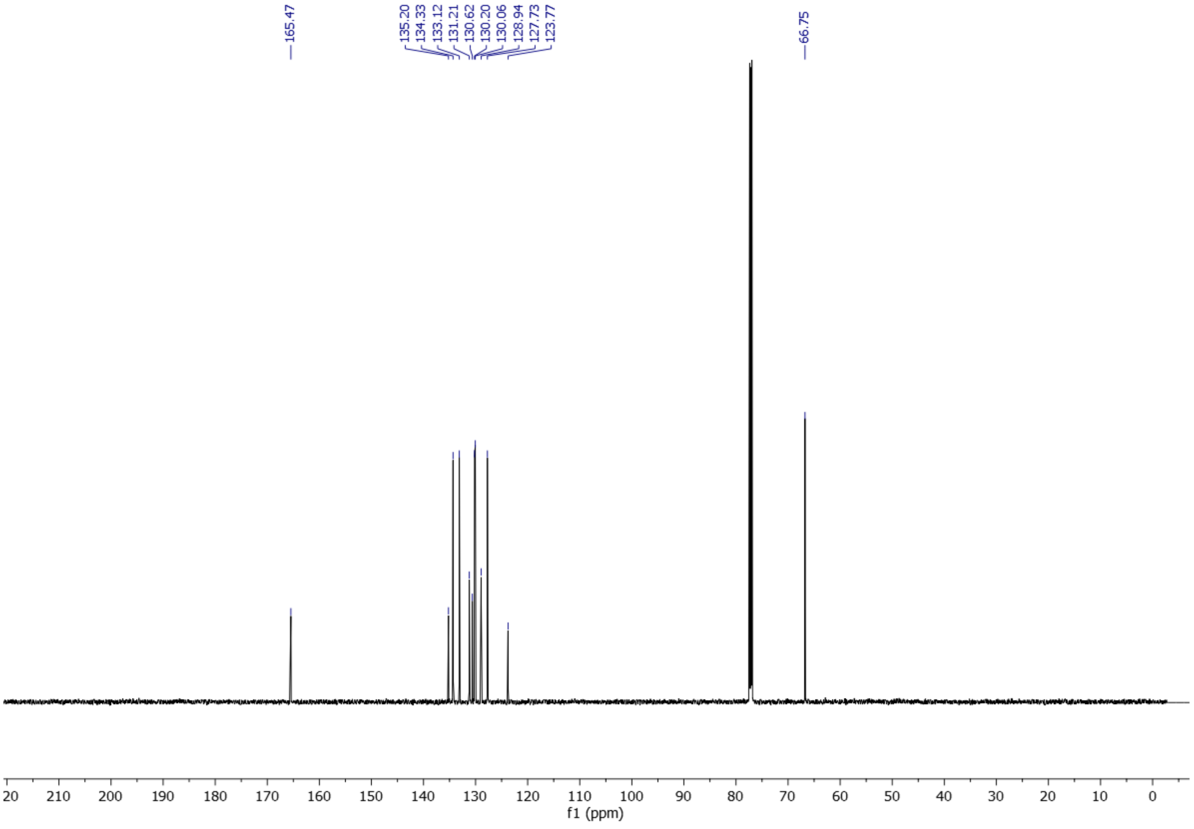

Figure S148. ^{1}H- and ^{13}C-NMR spectra of **bis(2-bromobenzyl) isophthalate (2c)** in $CDCl_3$.

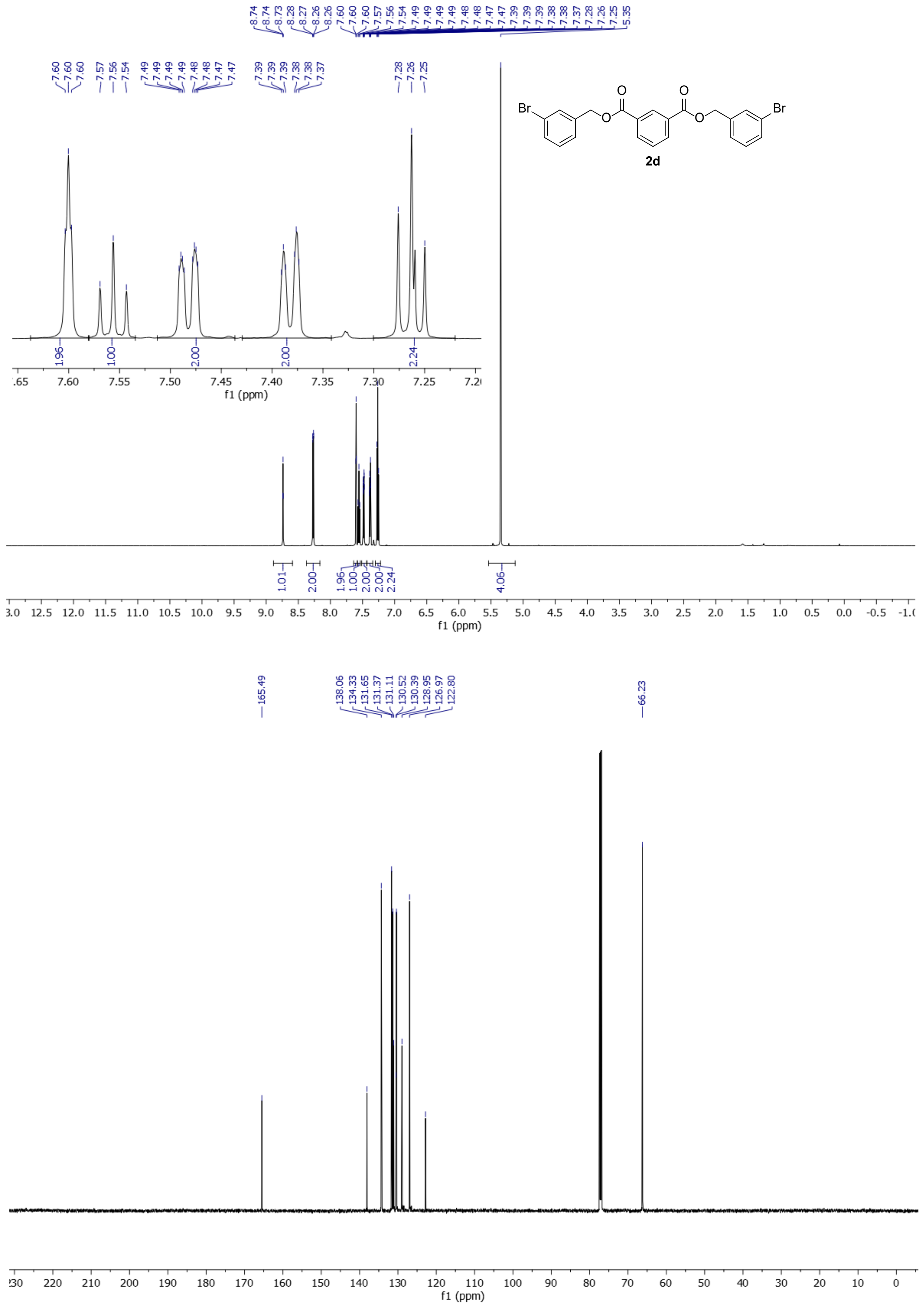

Figure S149. ^{1}H- and ^{13}C-NMR spectra of **bis(3-bromobenzyl) isophthalate (2d)** in $CDCl_3$.

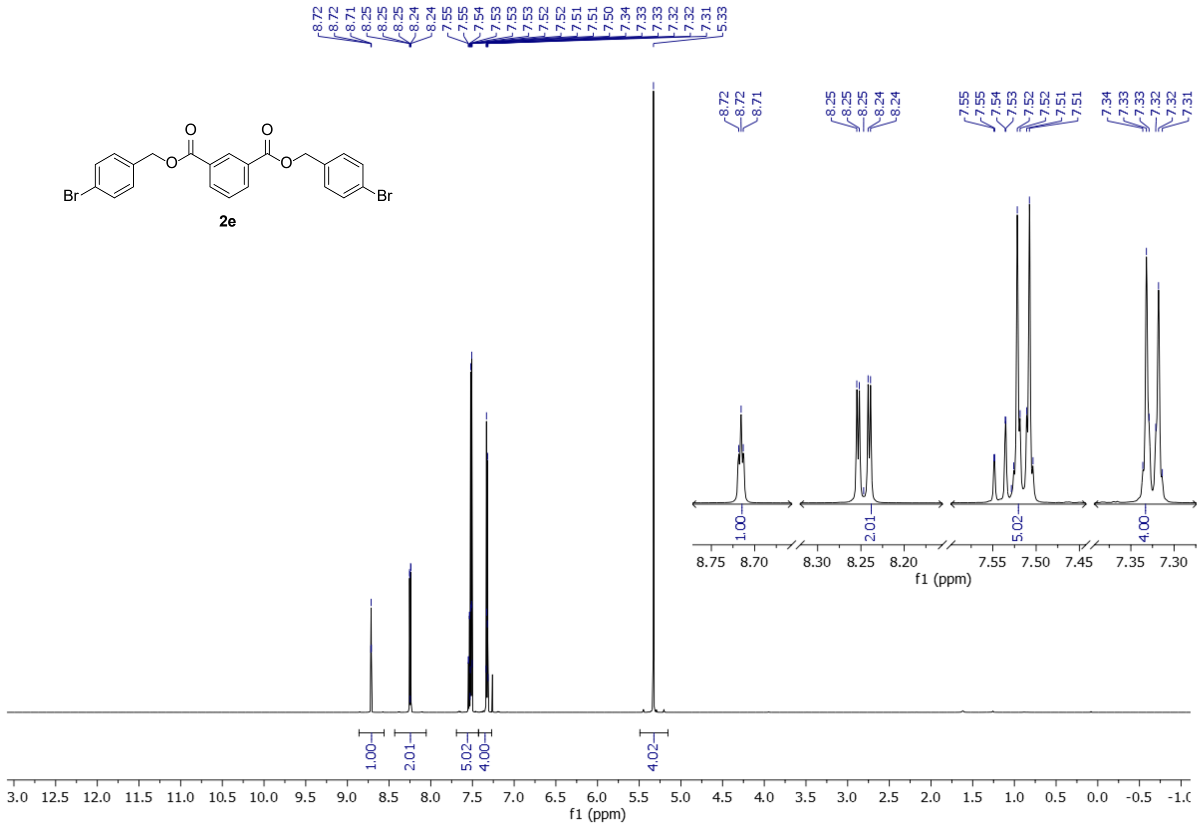

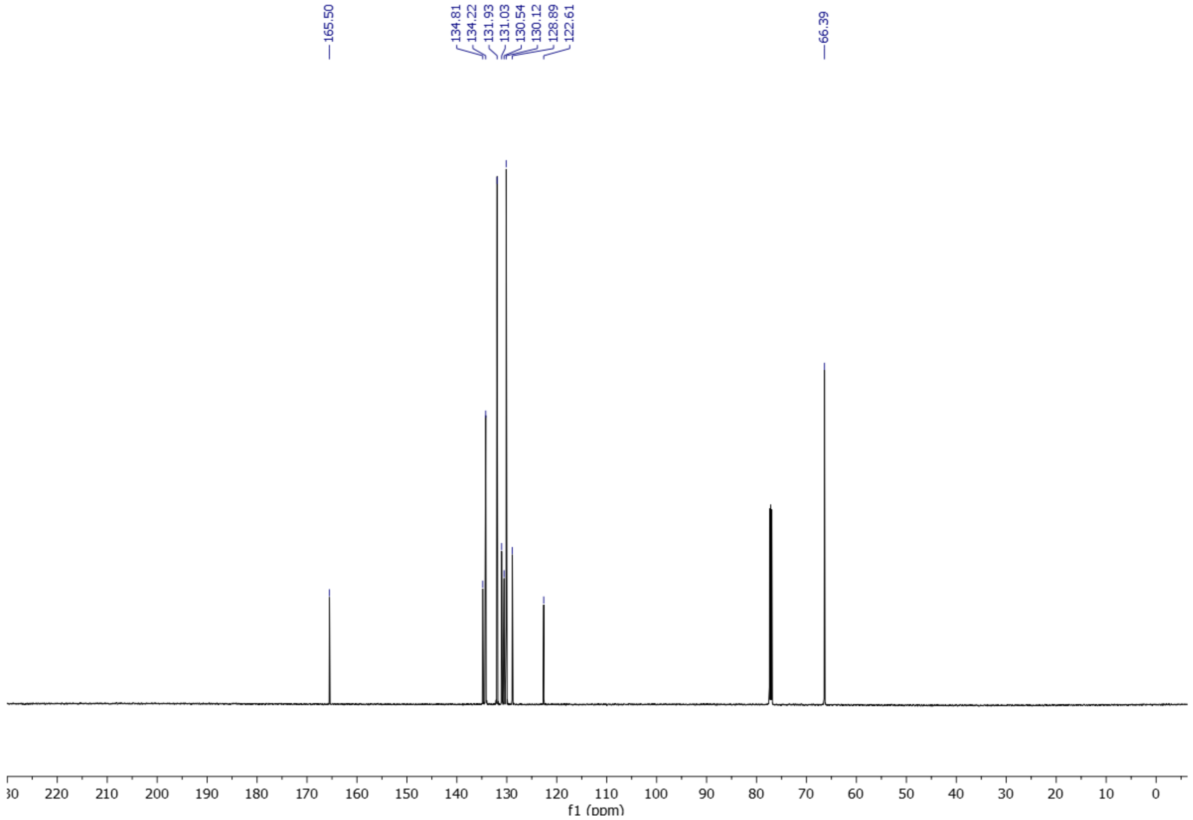

Figure S150. ^{1}H- and ^{13}C-NMR spectra of **bis(4-bromobenzyl) isophthalate (2e)** in $CDCl_3$.

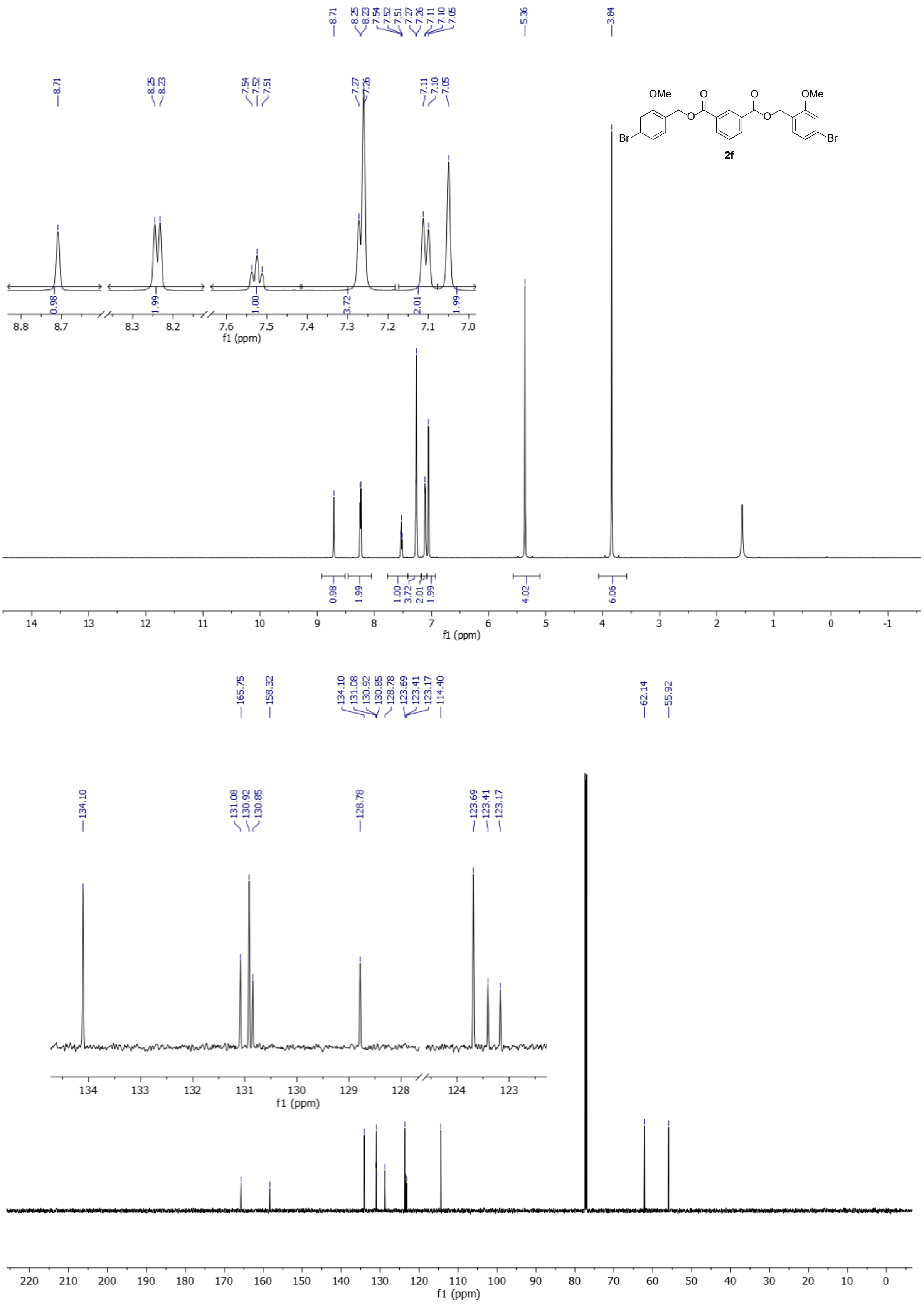

Figure S151. ^{1}H- and ^{13}C-NMR spectra of **bis(4-bromo-2-methoxybenzyl) isophthalate (2f)** in $CDCl_3$.

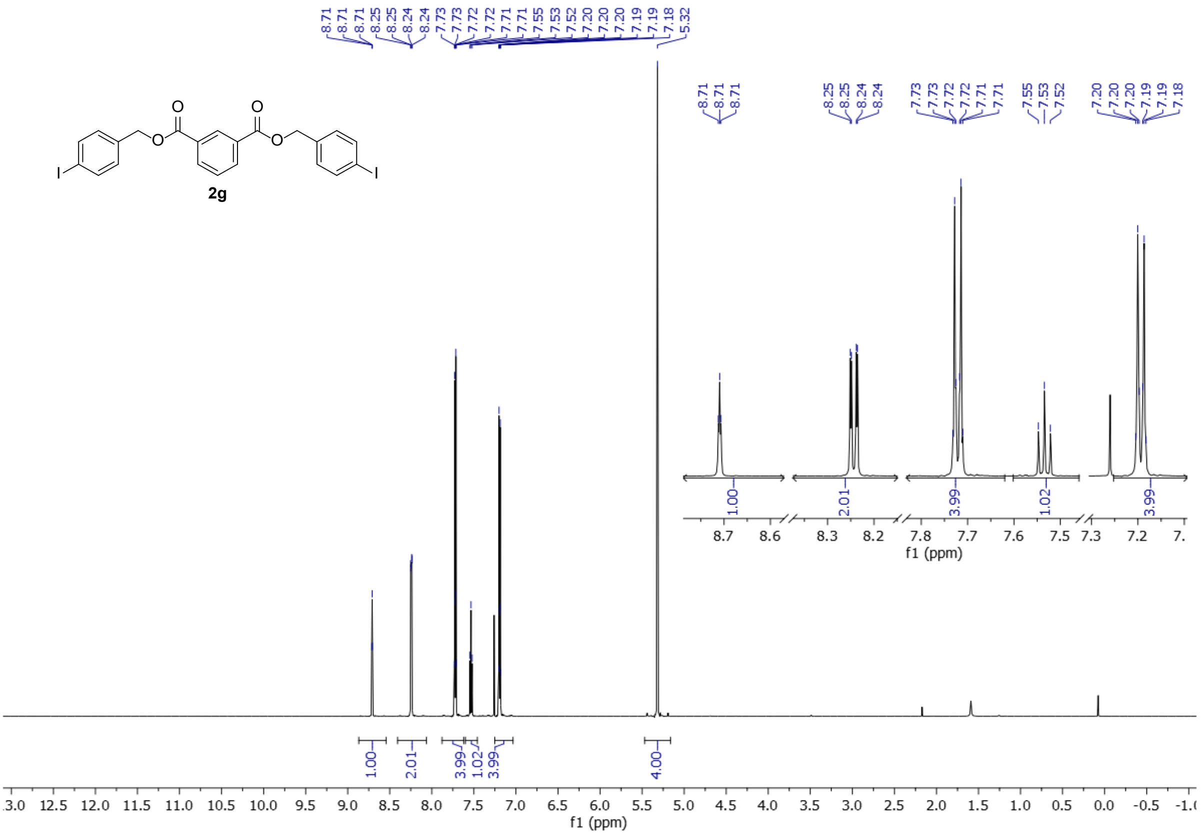

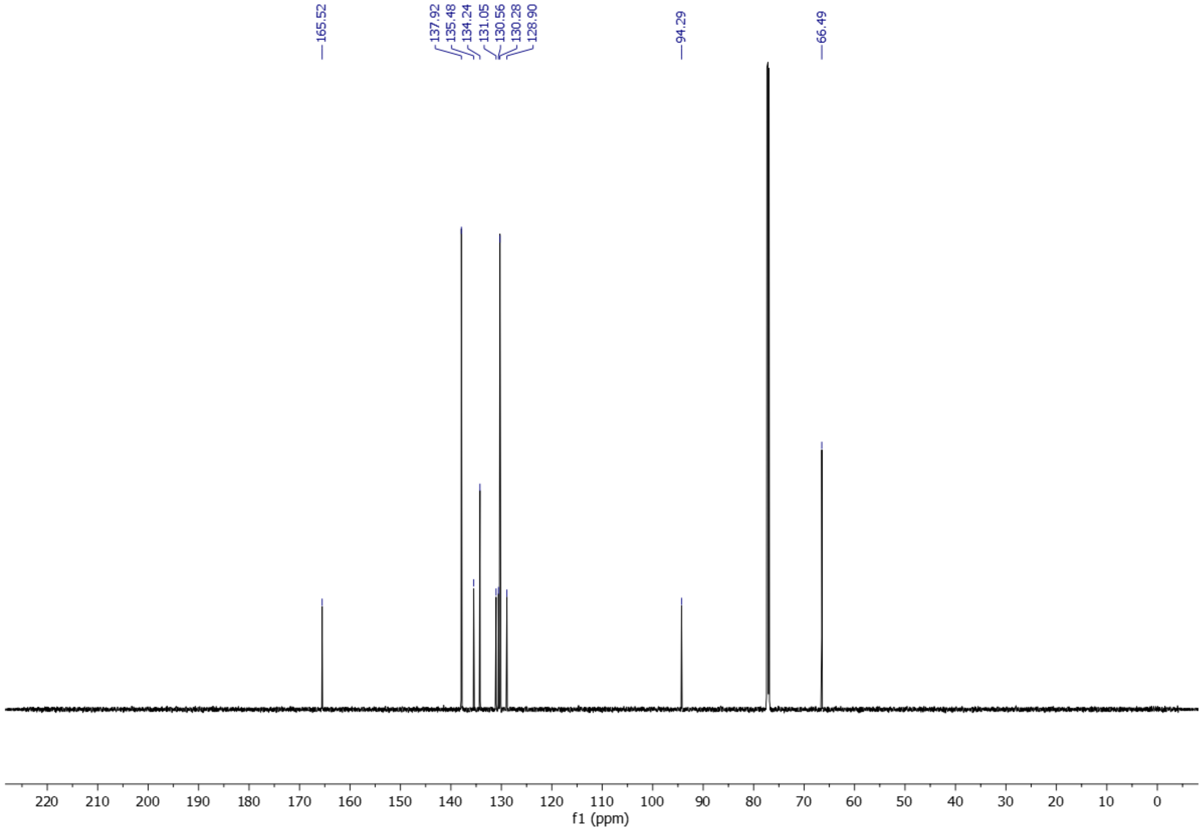

Figure S152. ^{1}H- and ^{13}C-NMR spectra of **bis(4-iodobenzyl) isophthalate (2g)** in $CDCl_3$.

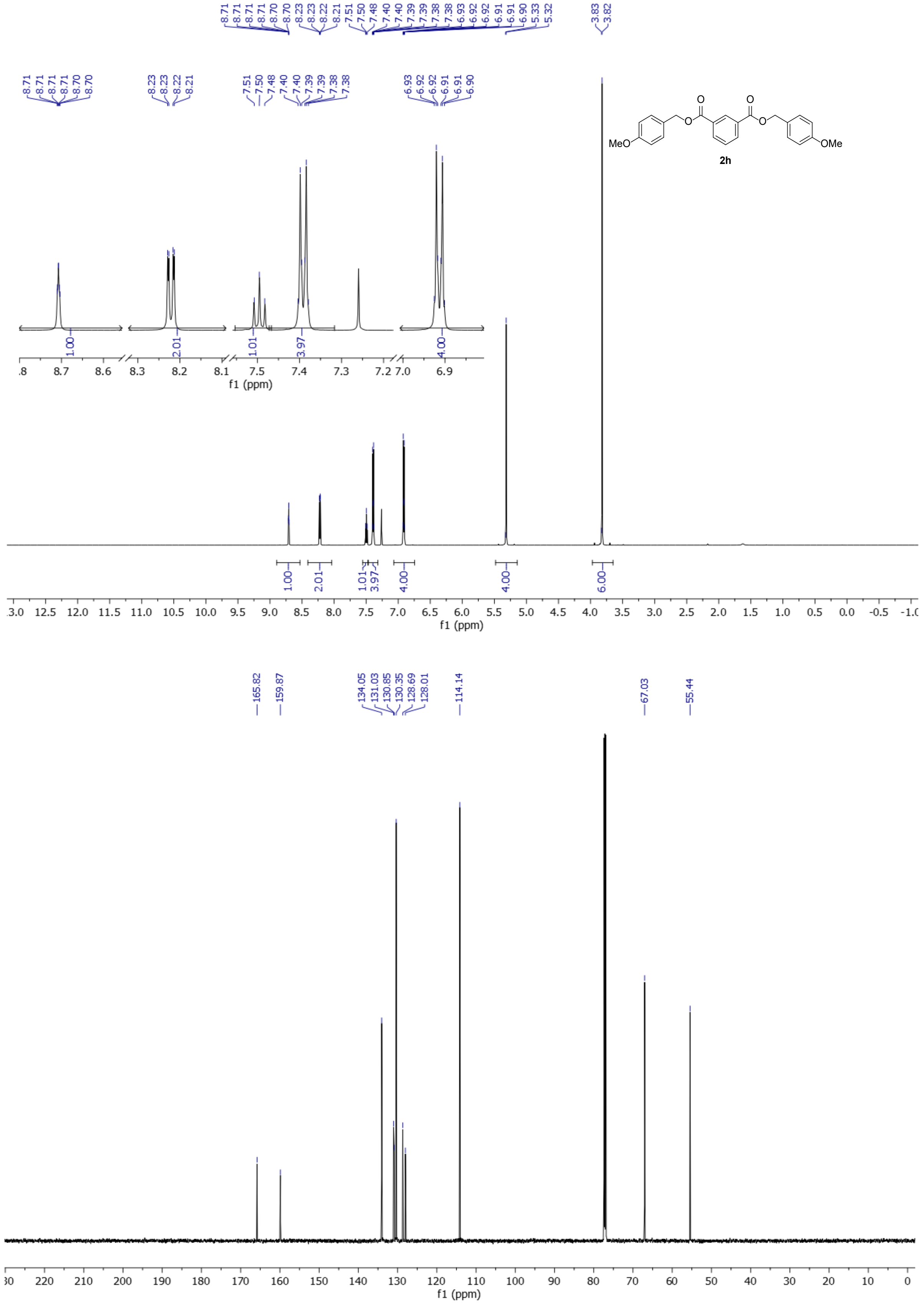

Figure S153. ^{1}H- and ^{13}C-NMR spectra of **bis(4-methoxybenzyl) isophthalate (2h)** in $CDCl_3$.

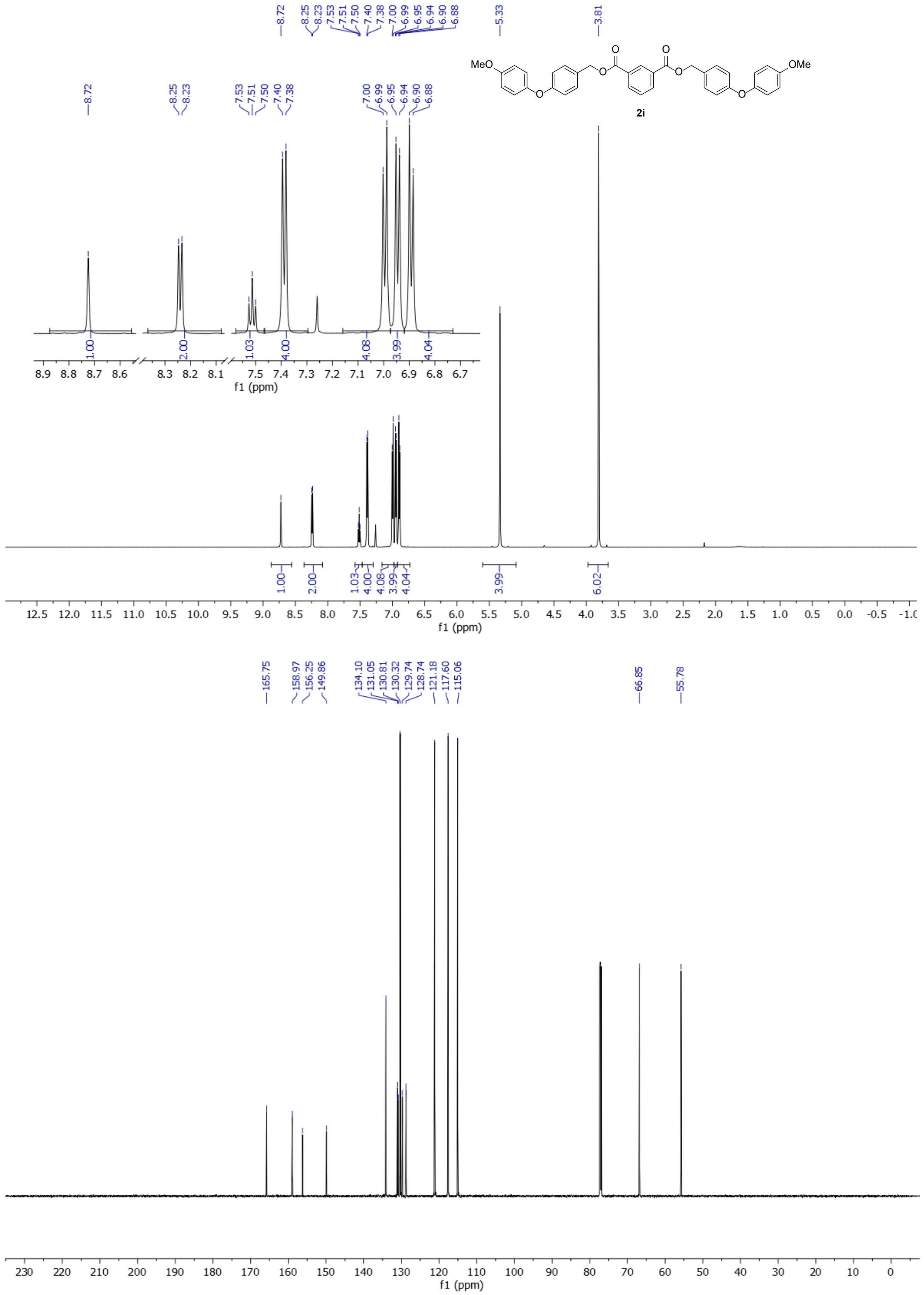

Figure S154. ^{1}H- and ^{13}C-NMR spectra of **bis(4-(4-methoxyphenoxy)benzyl) isophthalate (2i)** in $CDCl_3$.

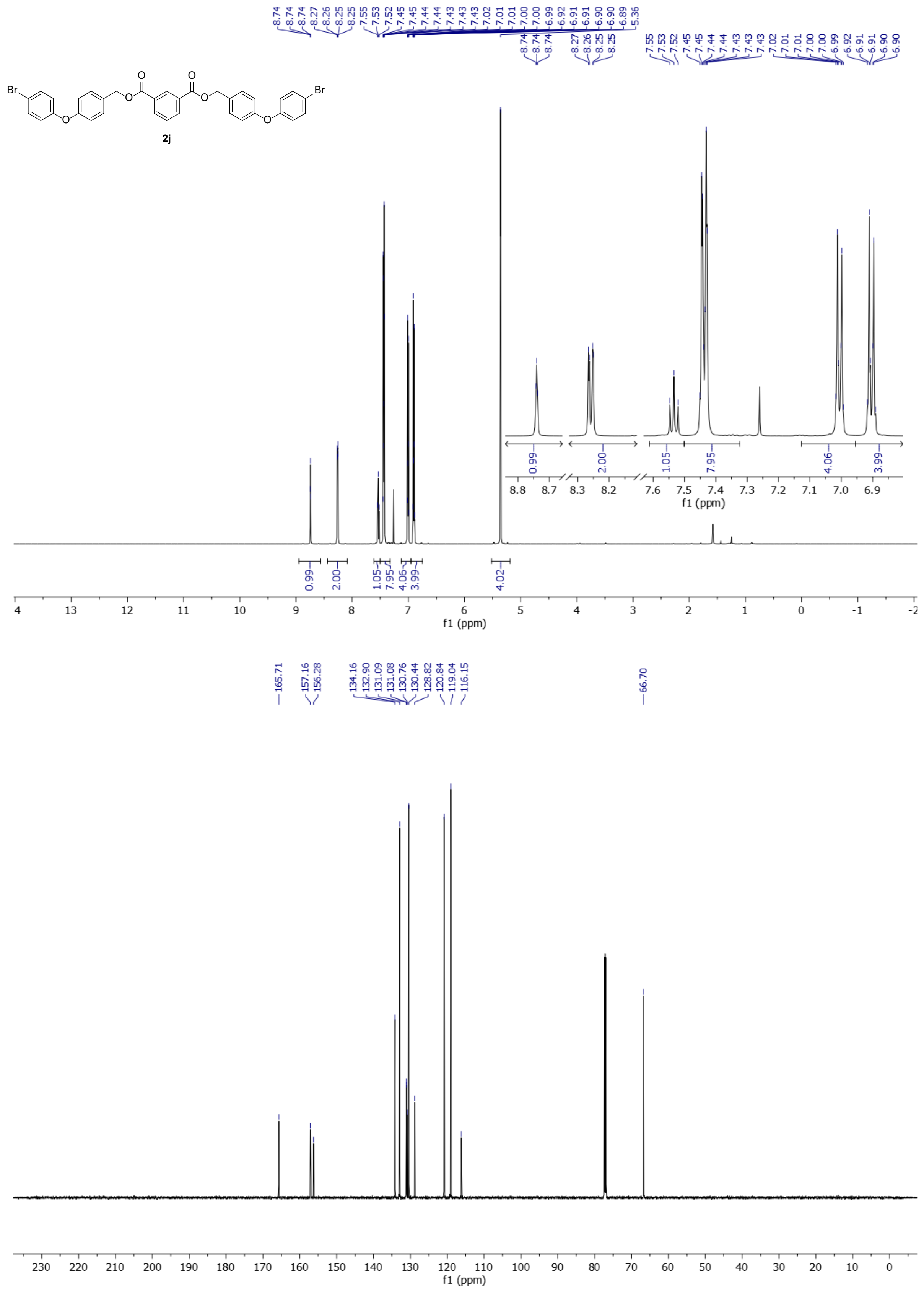

Figure S155. ^{1}H- and ^{13}C-NMR spectra of **bis(4-(4-bromophenoxy)benzyl) isophthalate (2j)** in $CDCl_3$.

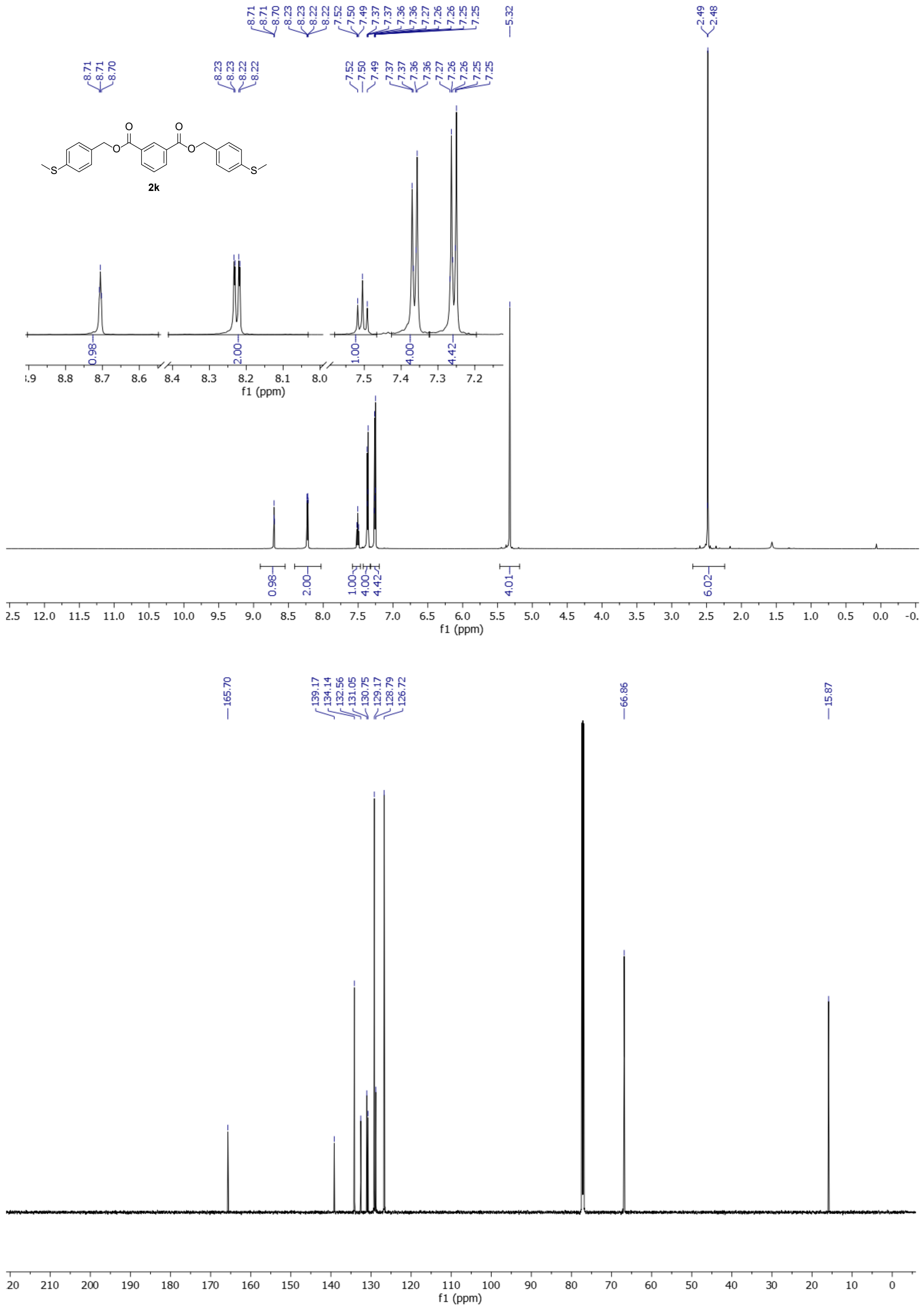

Figure S156. ^{1}H- and ^{13}C-NMR spectra of **bis(4-(methylthio)benzyl) isophthalate (2k)** in $CDCl_3$.

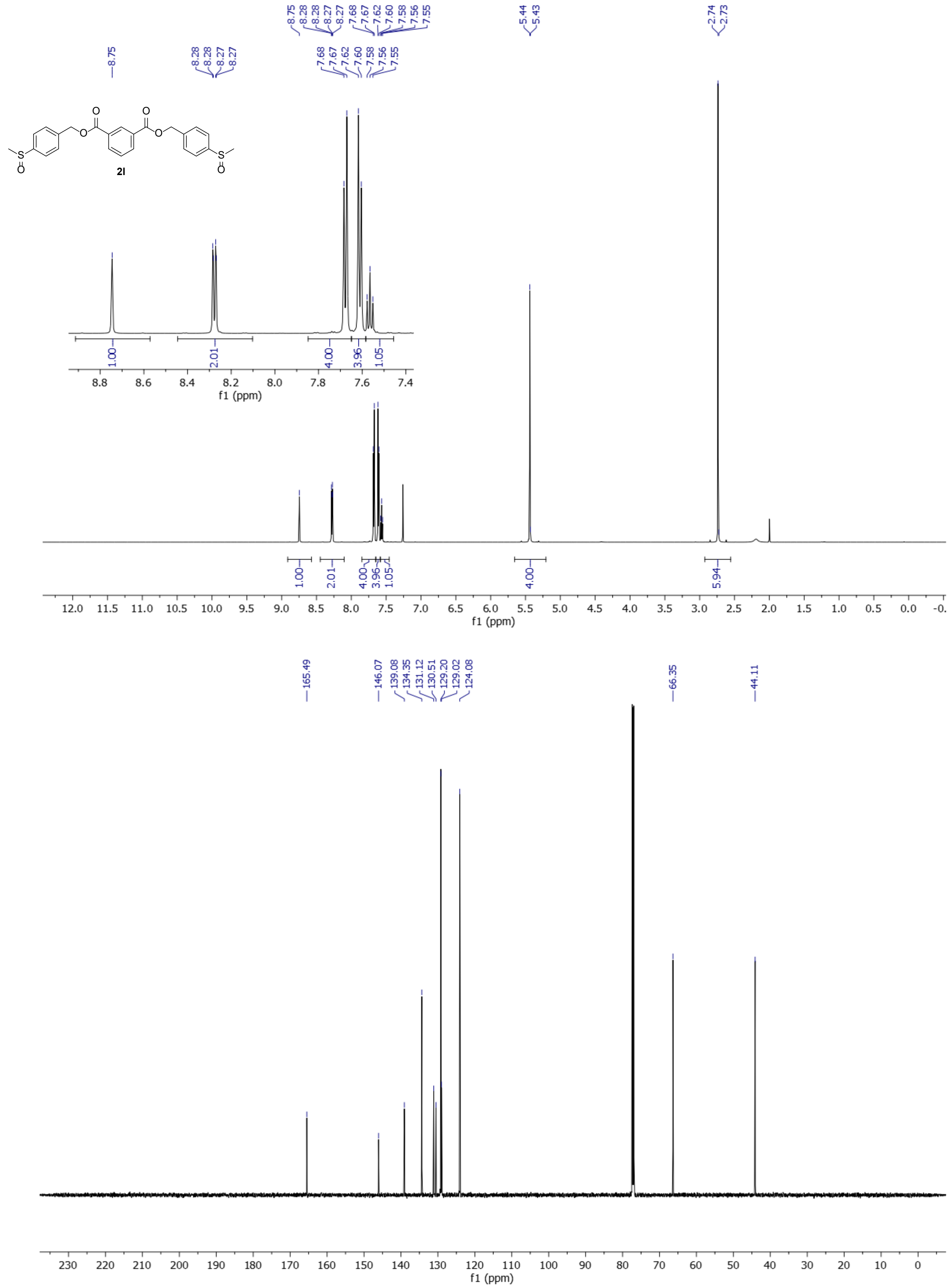

Figure S157. ^{1}H- and ^{13}C-NMR spectra of **bis(4-(methylsulfinyl)benzyl) isophthalate (2l)** in $CDCl_3$.

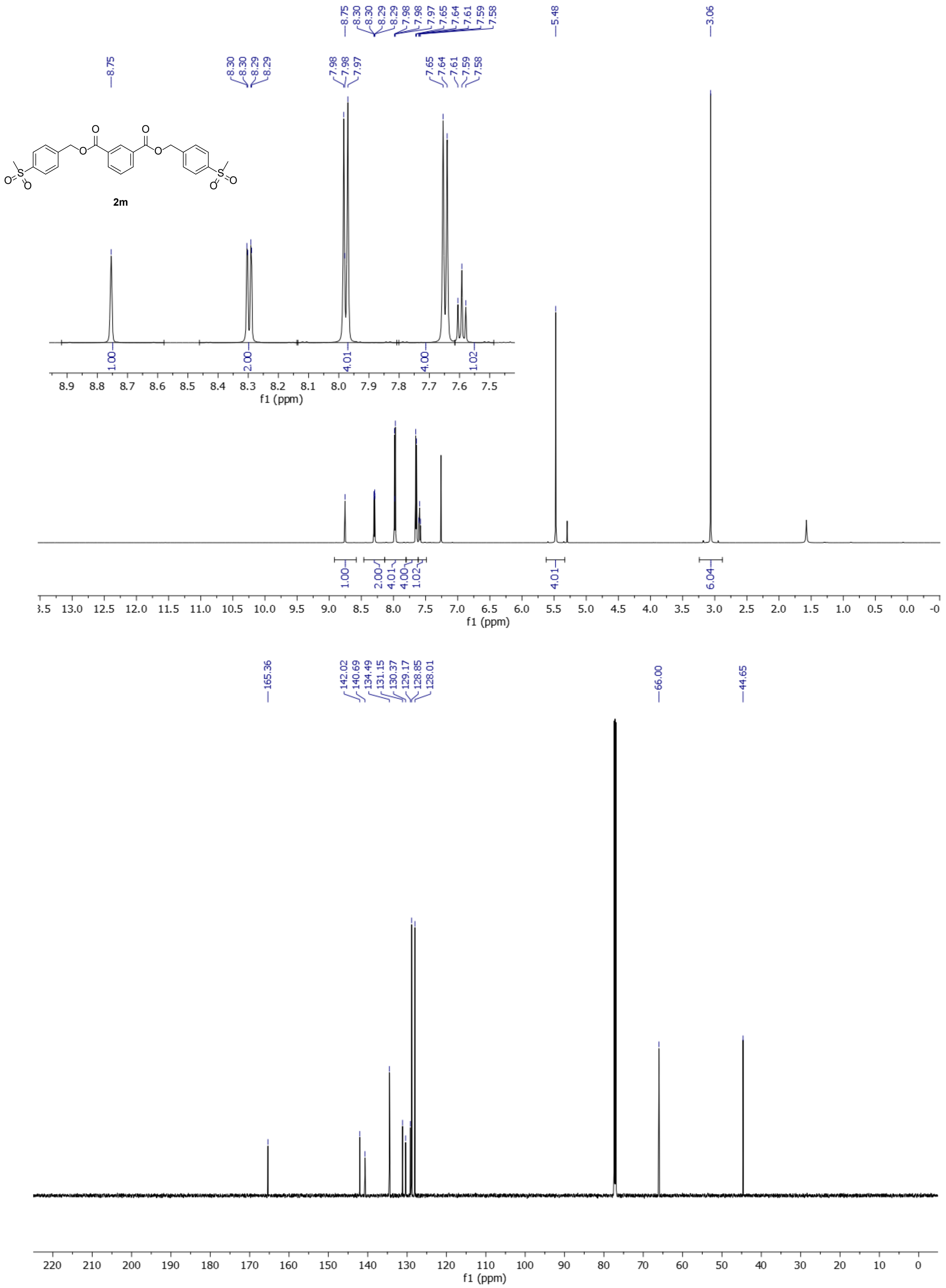

Figure S158. ^{1}H- and ^{13}C-NMR spectra of **bis(4-(methylsulfonyl)benzyl) isophthalate (2m)** in $CDCl_3$.

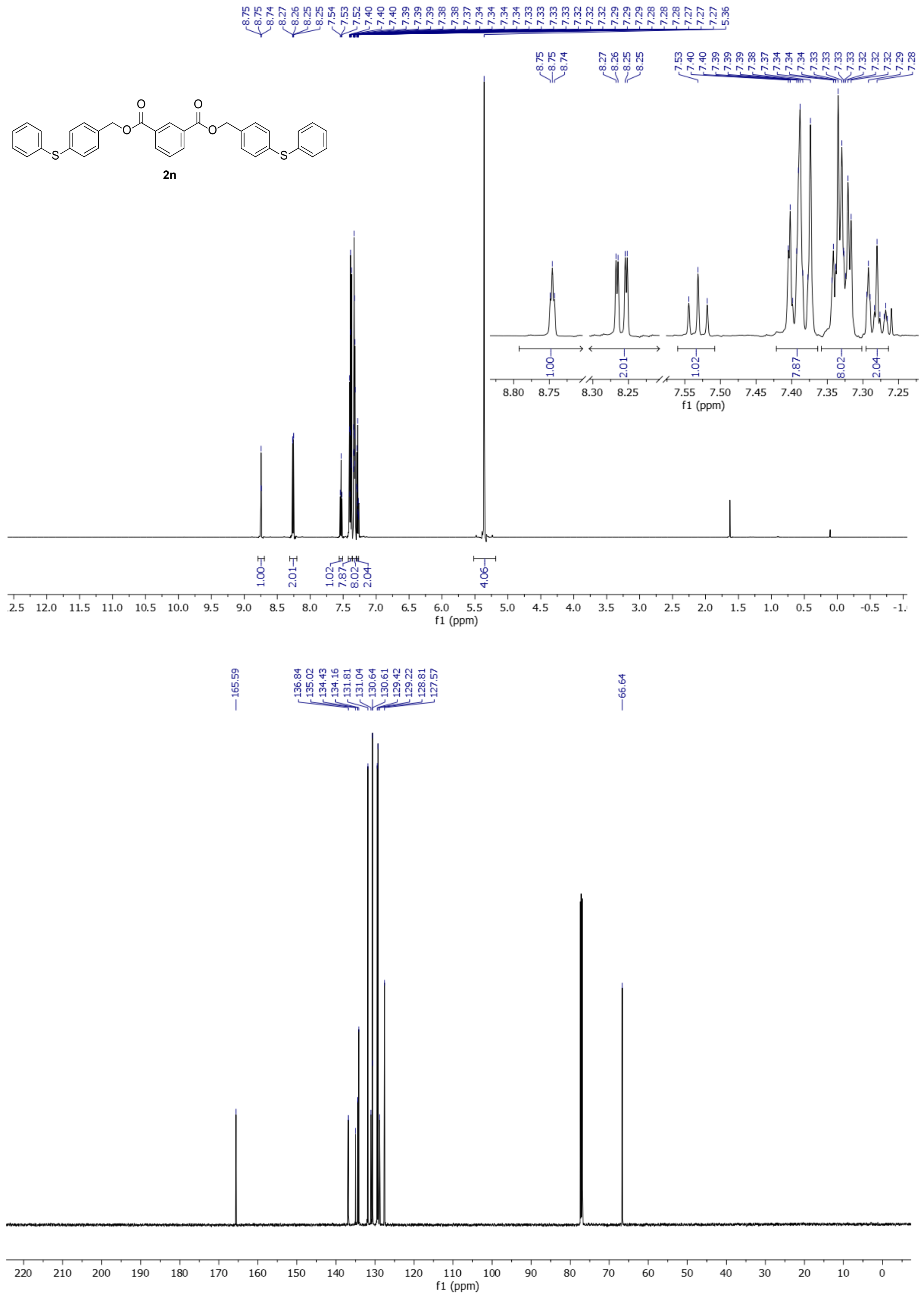

Figure S159. ^{1}H- and ^{13}C-NMR spectra of **bis(4-(phenylthio)benzyl) isophthalate (2n)** in $CDCl_3$.

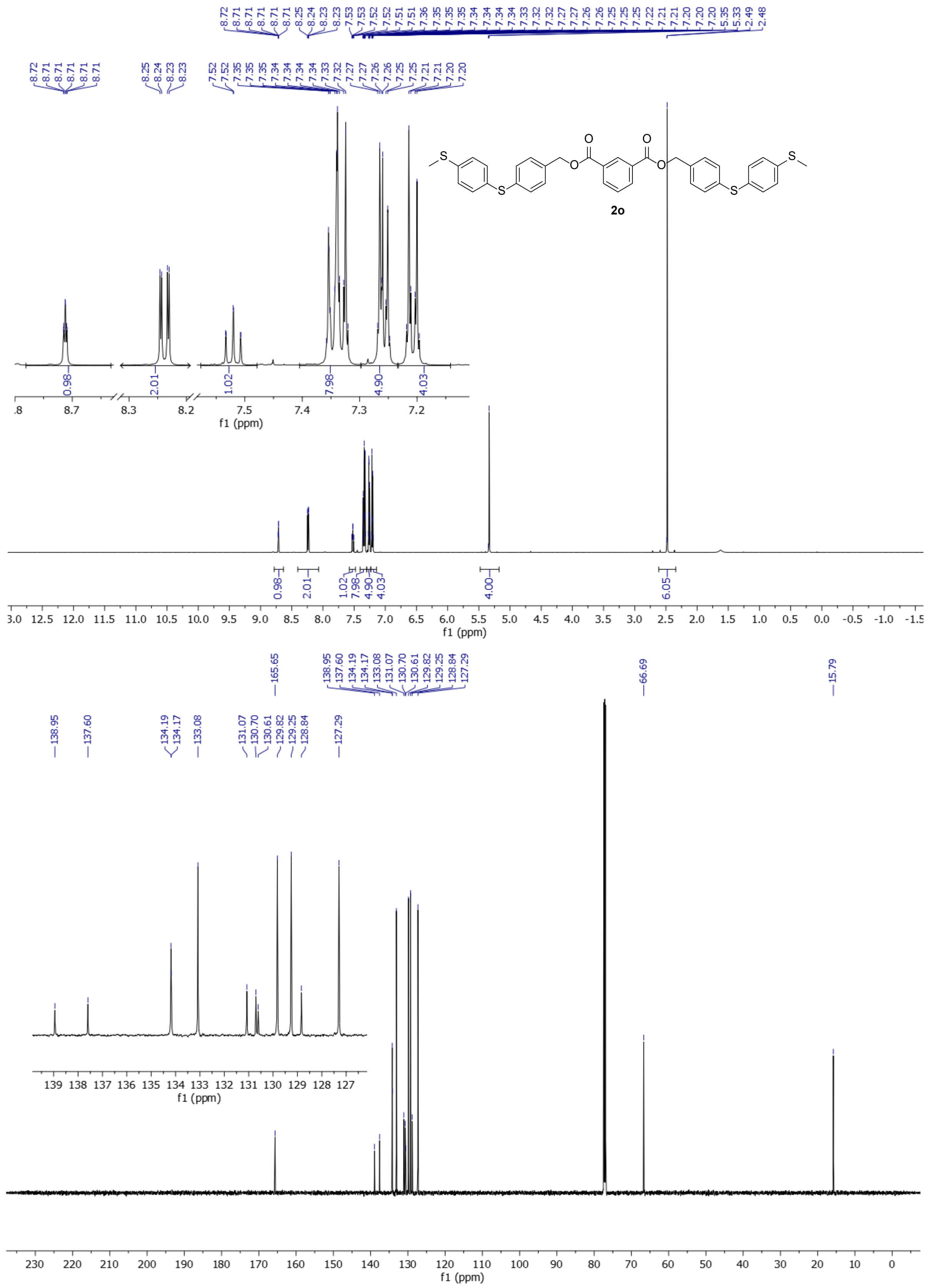

Figure S160. ^{1}H- and ^{13}C-NMR spectra of **bis(4-((4-methylthiophenyl)thio)benzyl) isophthalate (2o)** in $CDCl_3$. The solvent signal in the middle of the signal at 7.26 ppm (1-H NMR spectrum) overlaps with one signal of the compound.

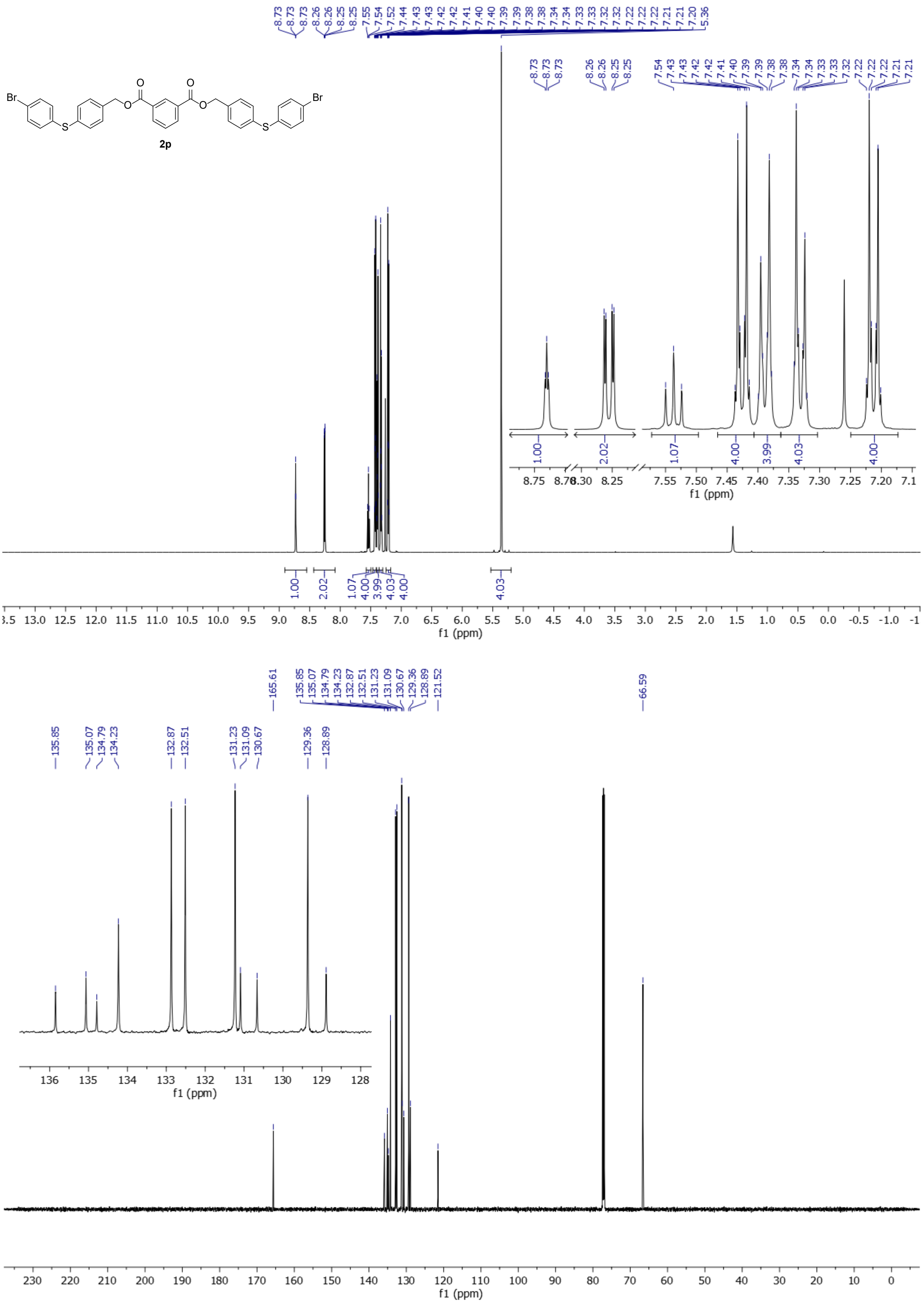

Figure S161. ^{1}H- and ^{13}C-NMR spectra of **bis(4-((4-bromophenyl)thio)benzyl) isophthalate (2p)** in $CDCl_3$

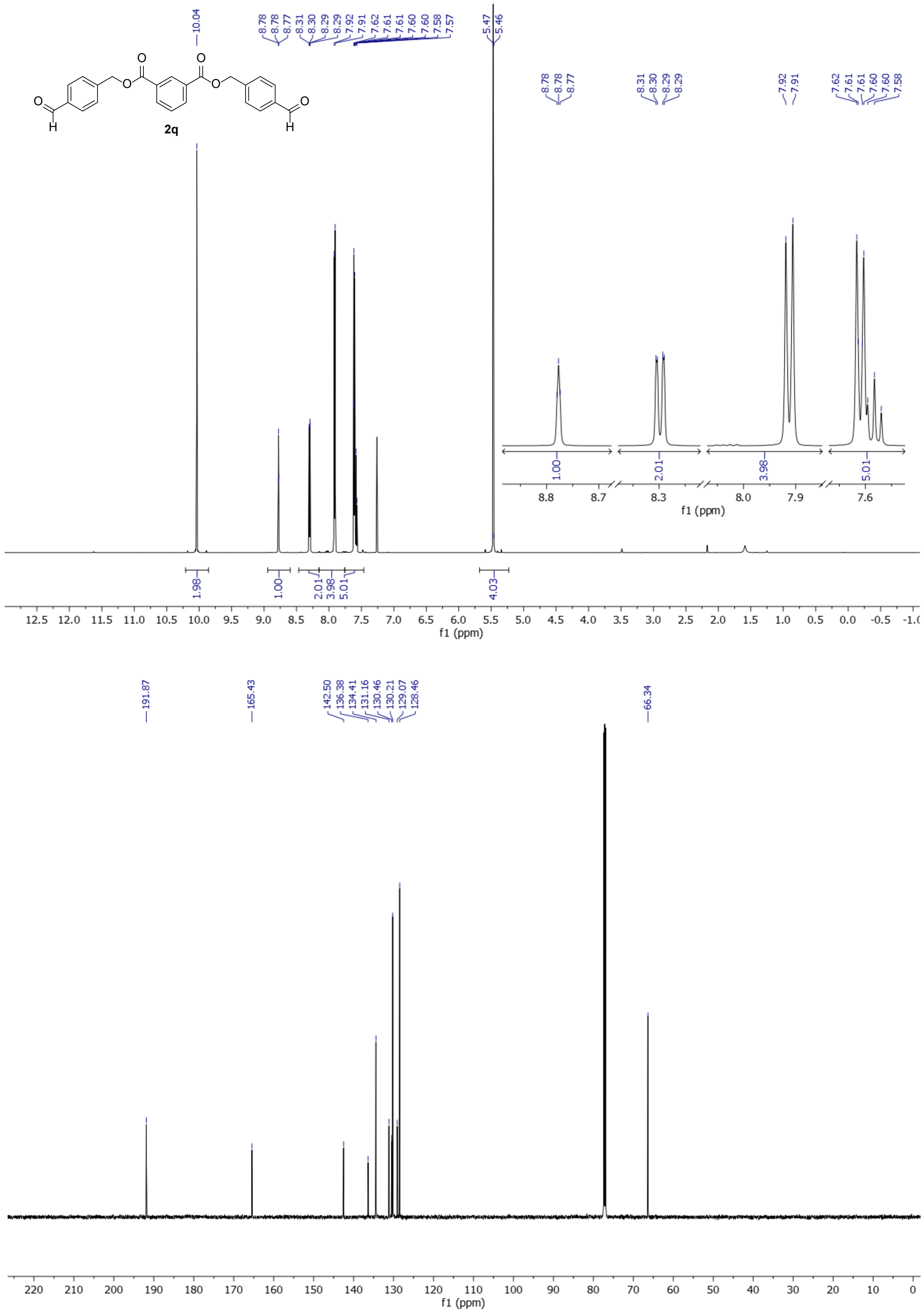

Figure S162. ^{1}H- and ^{13}C-NMR spectra of **bis(4-formylbenzyl) isophthalate (2q)** in $CDCl_3$.

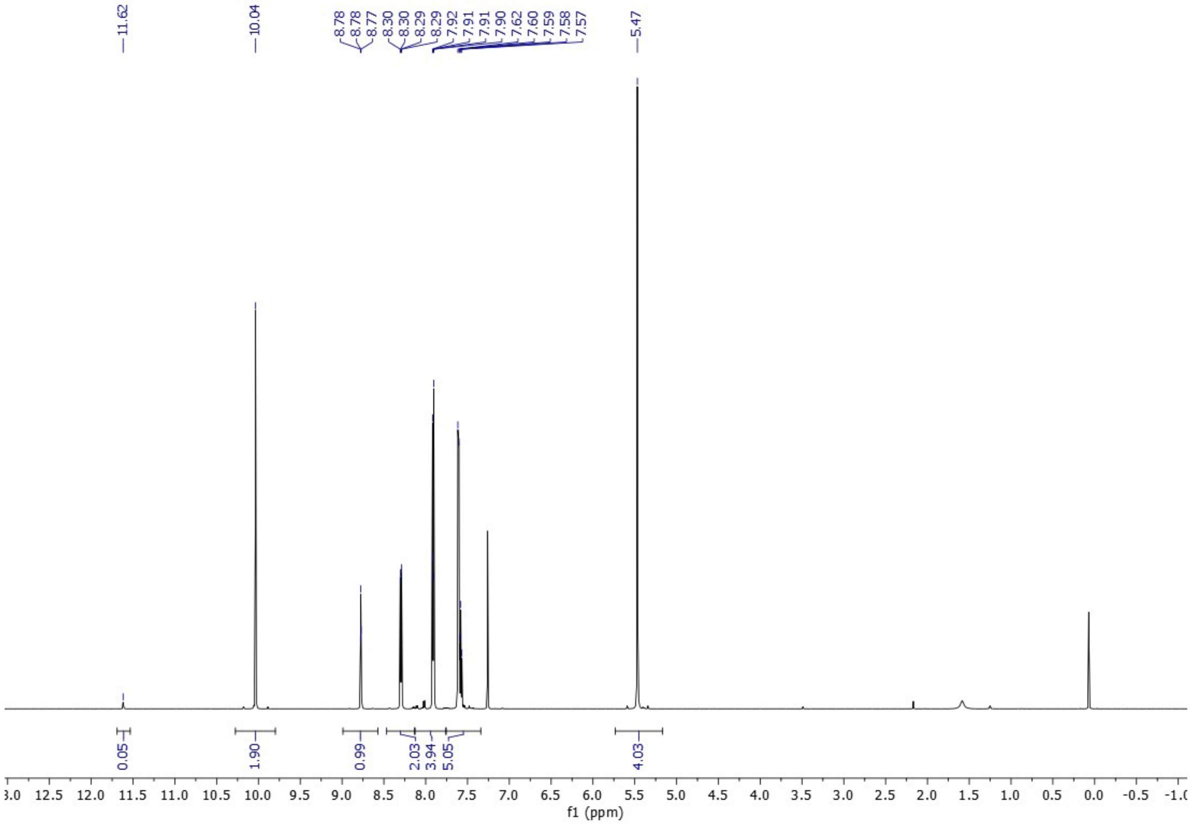

Figure S163. ^{1}H-NMR spectrum of **bis(4-formylbenzyl) isophthalate (2q)** in $CDCl_3$, after the solid had been stored in a closed vial under an air atmosphere at 4 °C for 17 months. Ca. 4 % of the aldehyde groups degraded over time. Please note that the new signal at ca. 0.1 ppm is due to grease from the cap of the nmr tube and not from the sample.

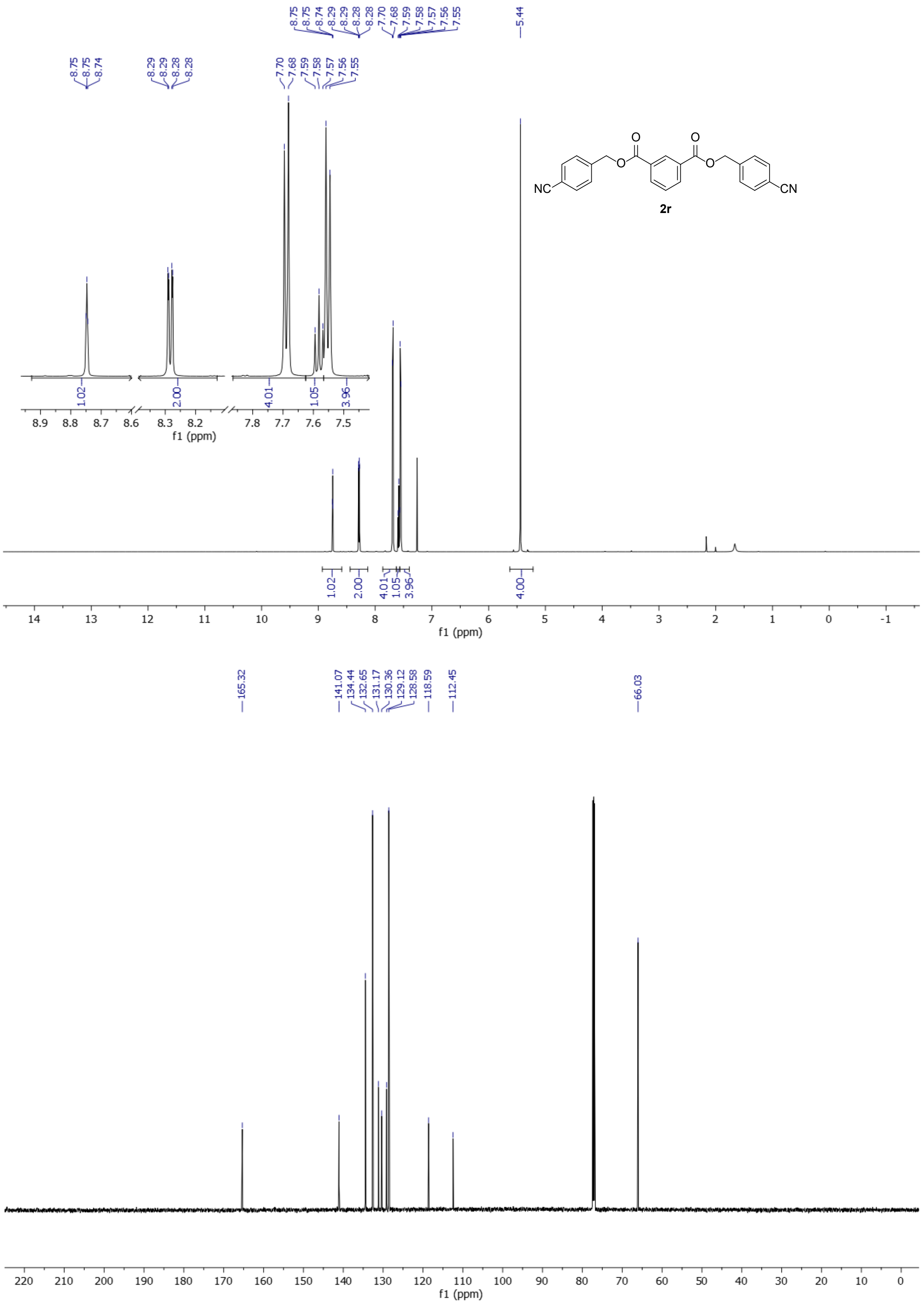

Figure S164. ^{1}H- and ^{13}C-NMR spectra of **bis(4-cyanobenzyl) isophthalate (2r)** in $CDCl_3$.

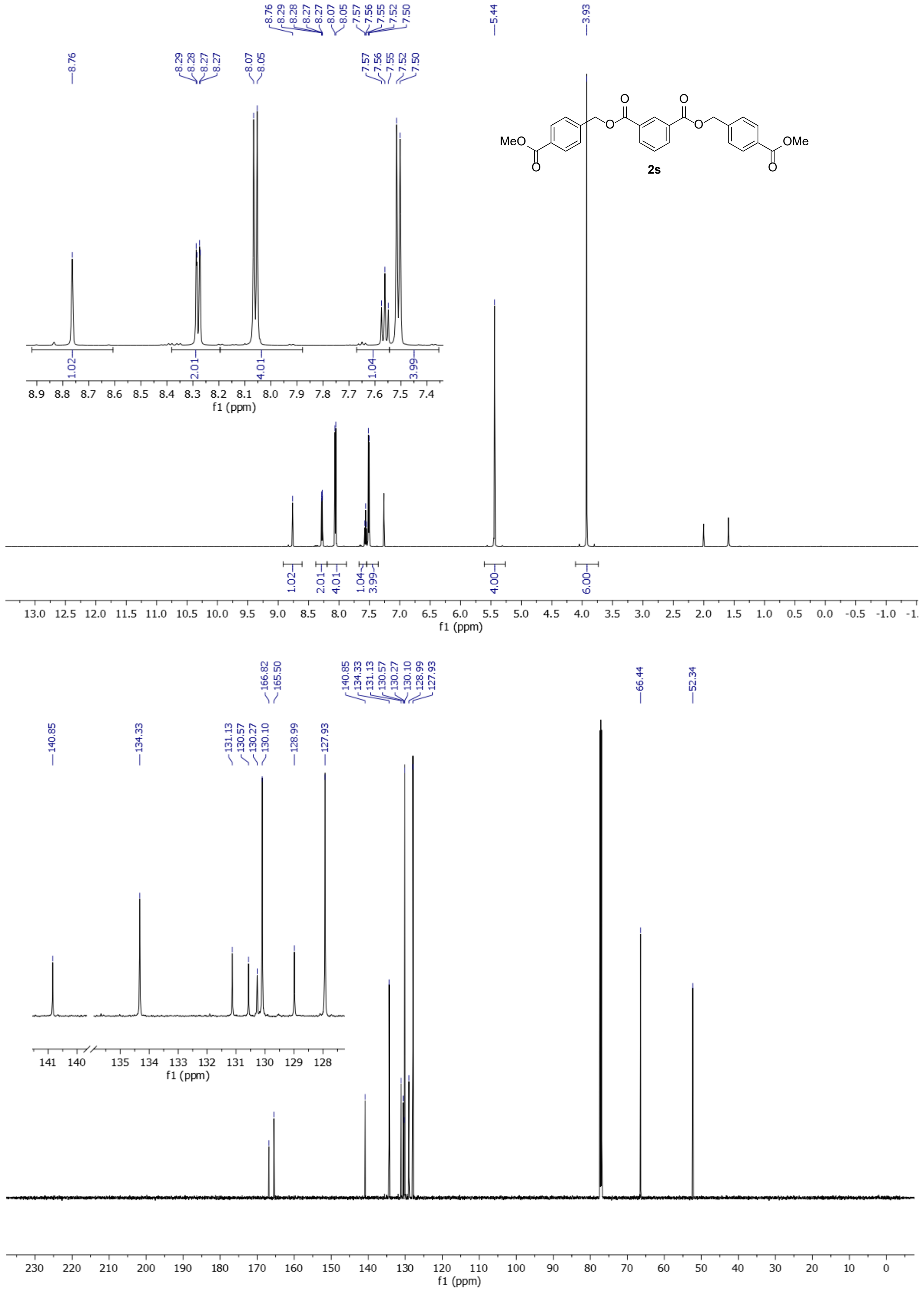

Figure S165. ^{1}H- and ^{13}C-NMR spectra of **bis(4-(methoxycarbonyl)benzyl) isophthalate (2s)** in $CDCl_3$.

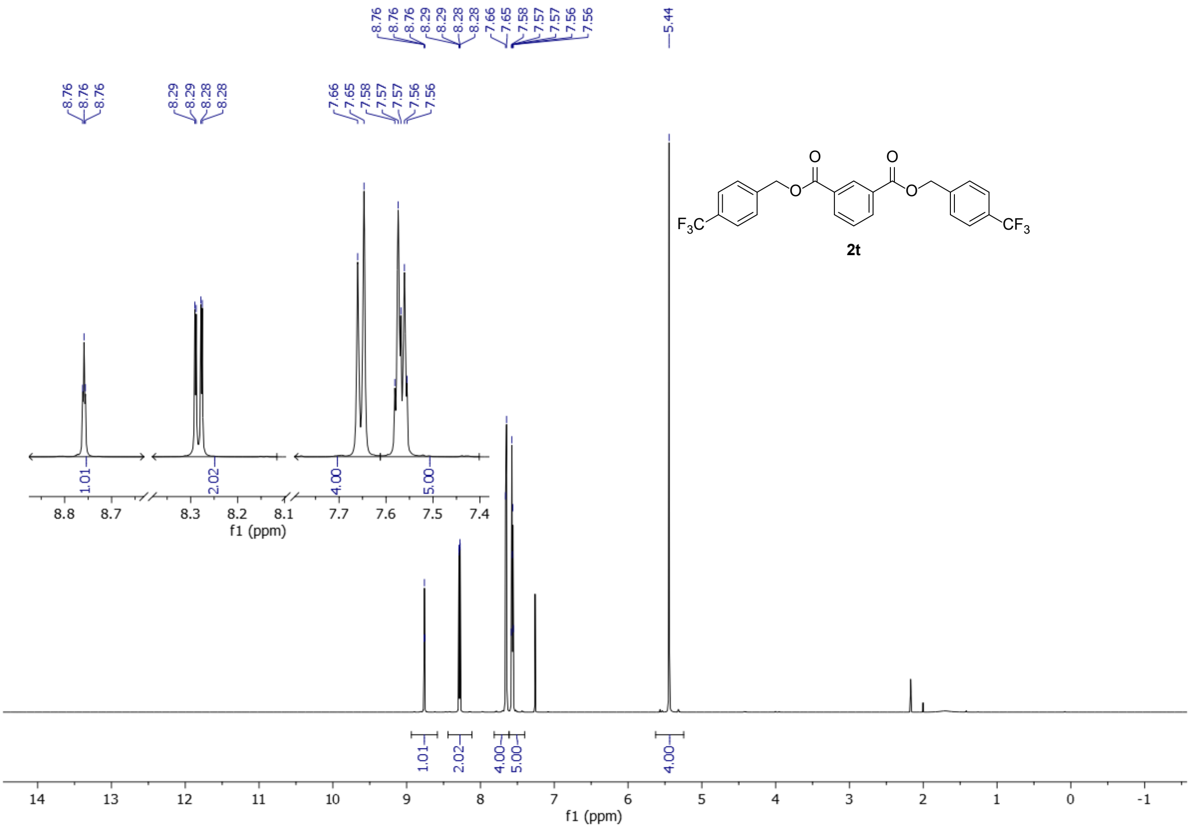
8.76
8.76
8.76
8.29
8.29
8.28
8.28
7.66
7.65
7.58
7.57
7.57
7.56
7.56
5.44
O
O
O
O
F3C
CF3
2t
1.01
2.02
4.00
5.00
f1 (ppm)
4.00

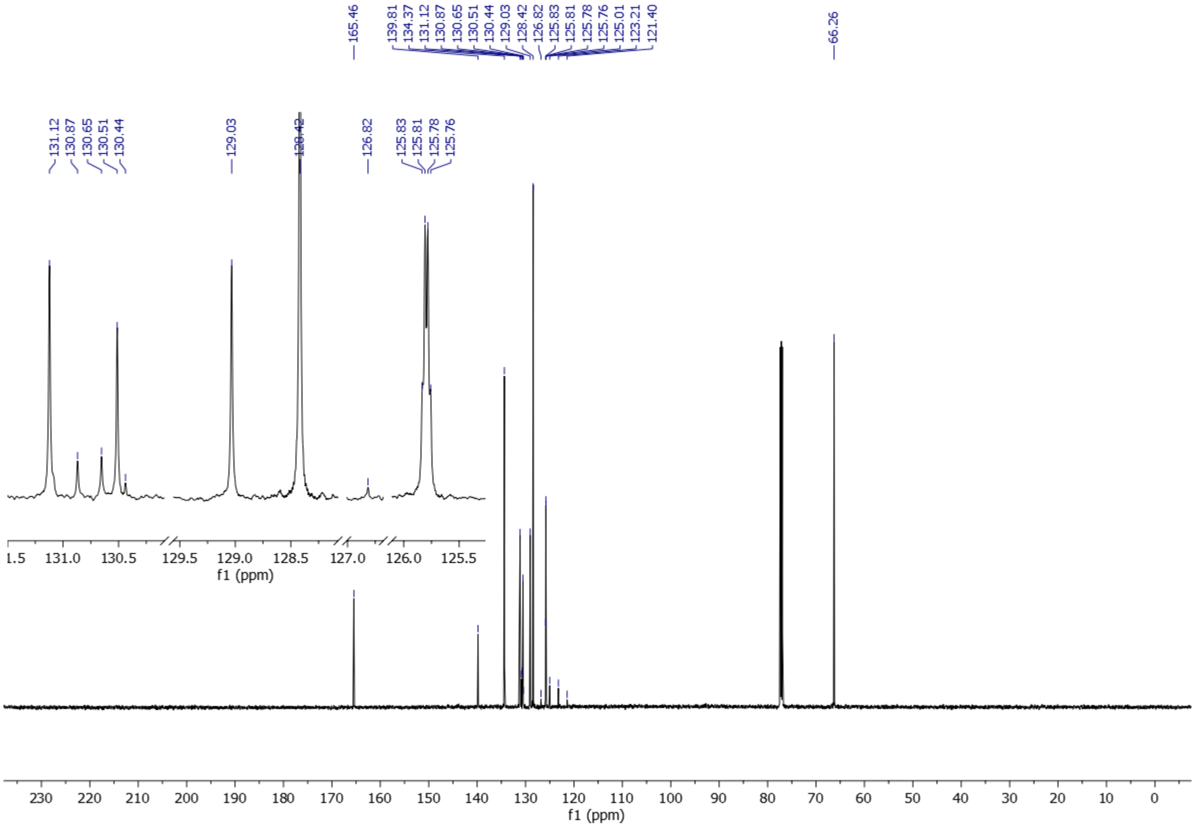
165.46
139.81
134.37
131.12
130.87
130.65
130.51
130.44
129.03
128.42
126.82
125.83
125.81
125.78
125.76
125.01
123.21
121.40
66.26
f1 (ppm)

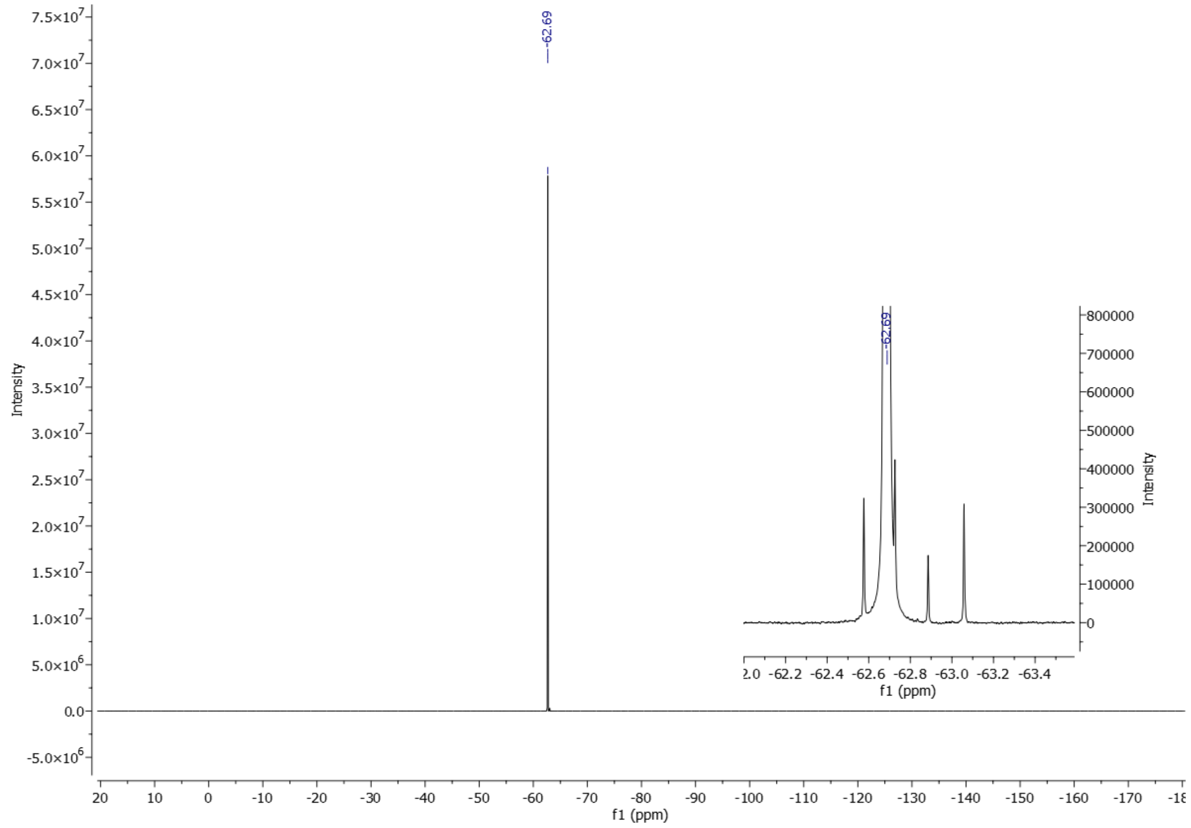

Figure S166. ^{1}H-, ^{13}C and ^{19}F-NMR spectra of **bis(4-trifluoromethylbenzyl) isophthalate (2t)** in $CDCl_3$.

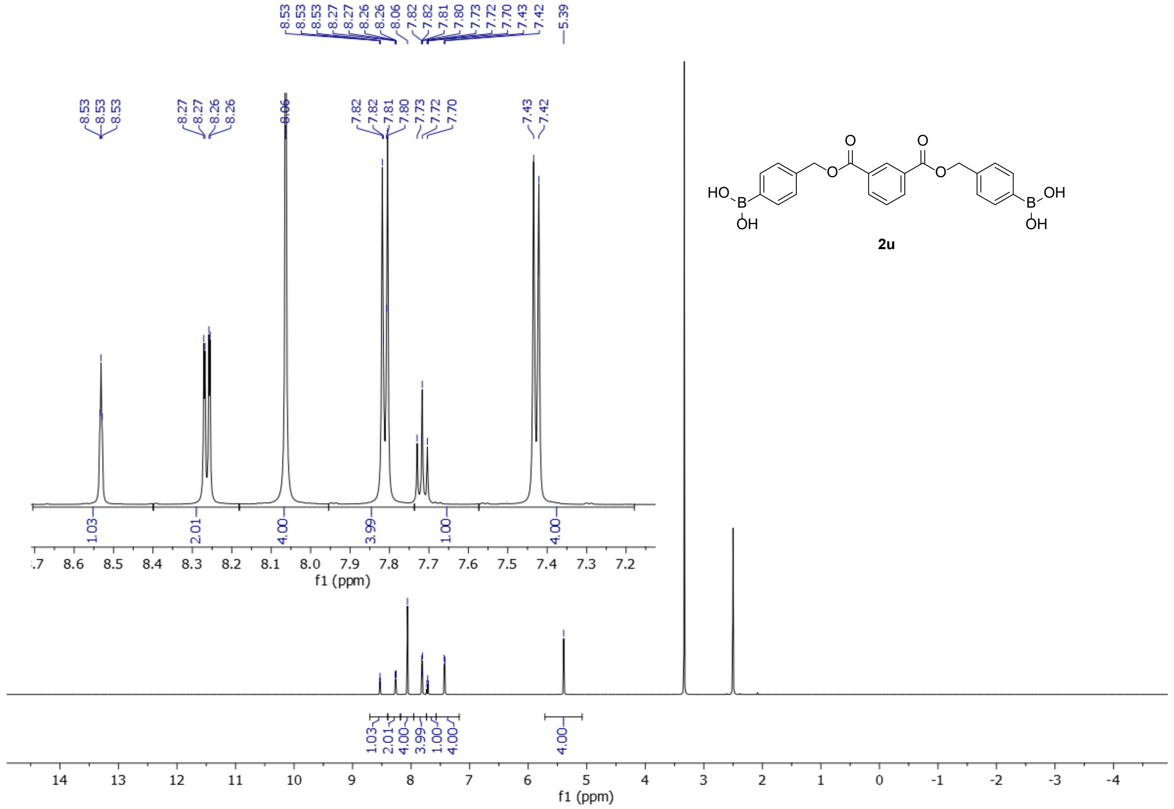

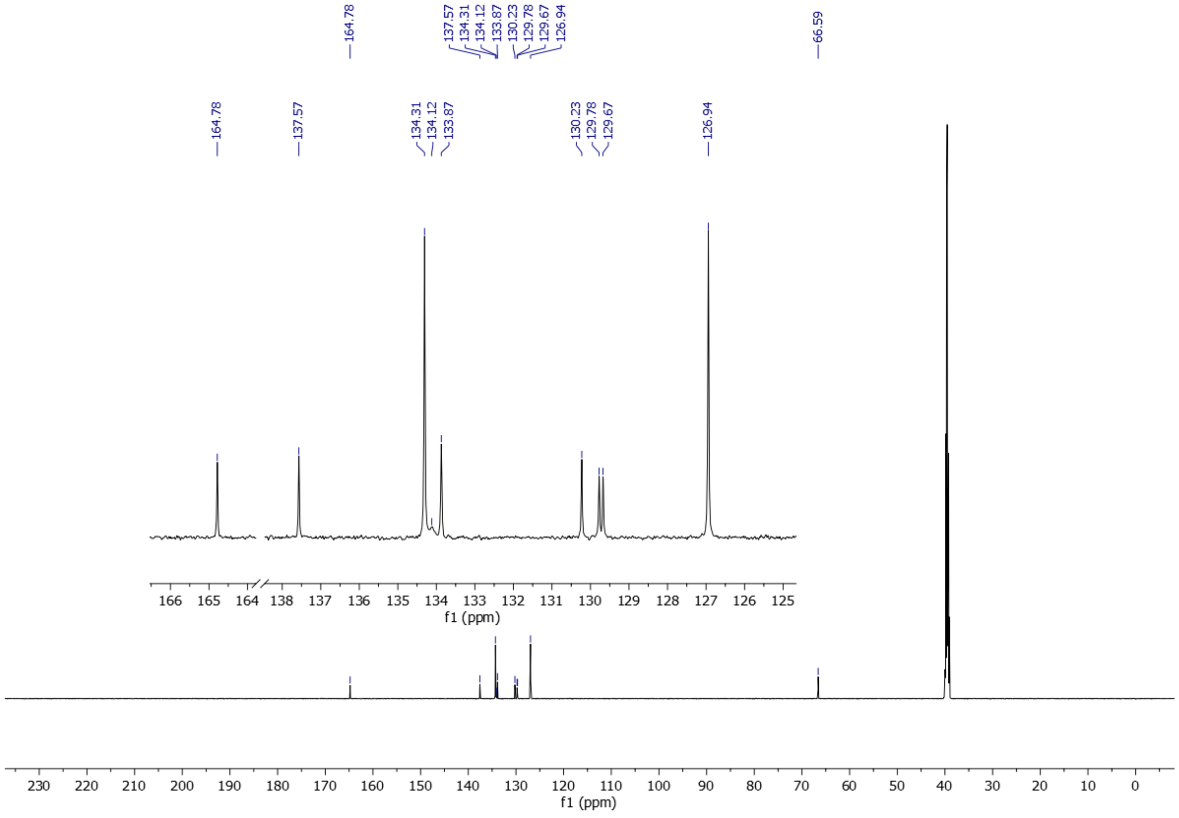

Figure S167. ^{1}H- and ^{13}C-NMR spectra of **(((isophthaloylbis(oxy))bis(methylene))bis(4,1-phenylene))diboronic acid (2u)** in DMSO-d_6.

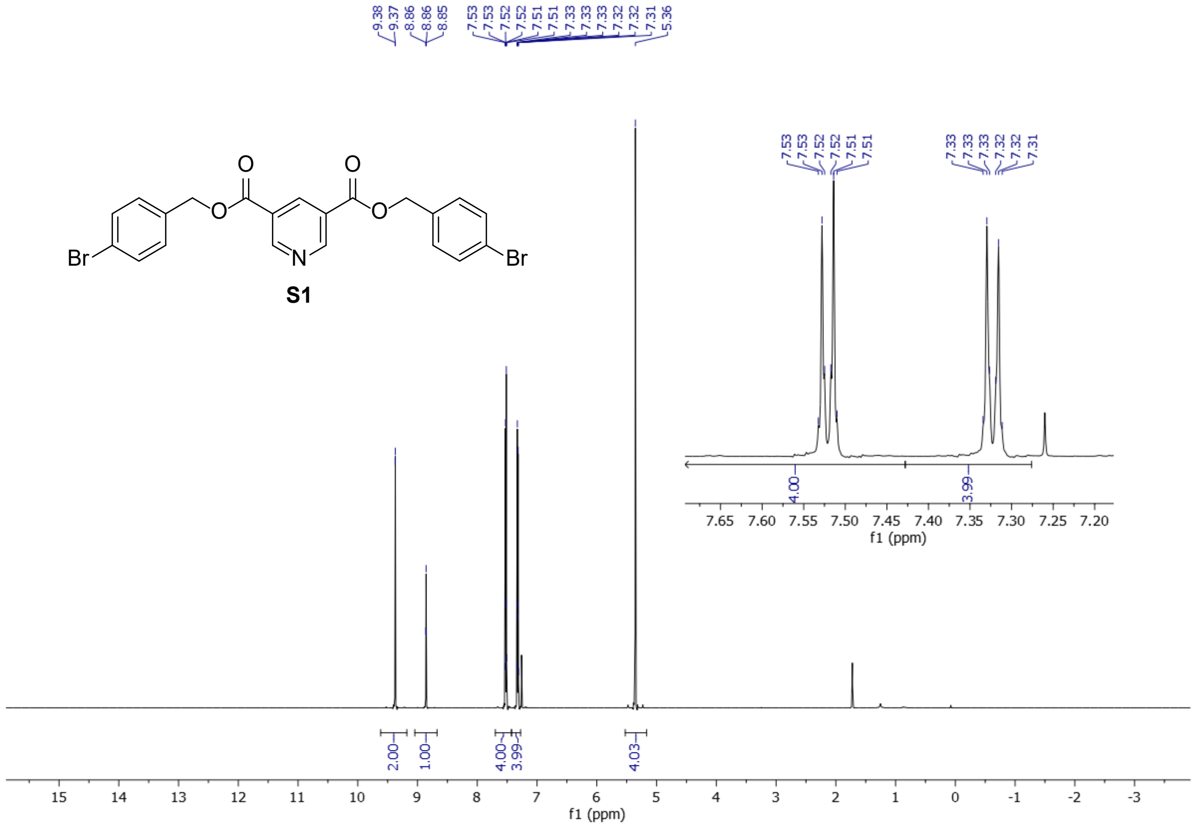

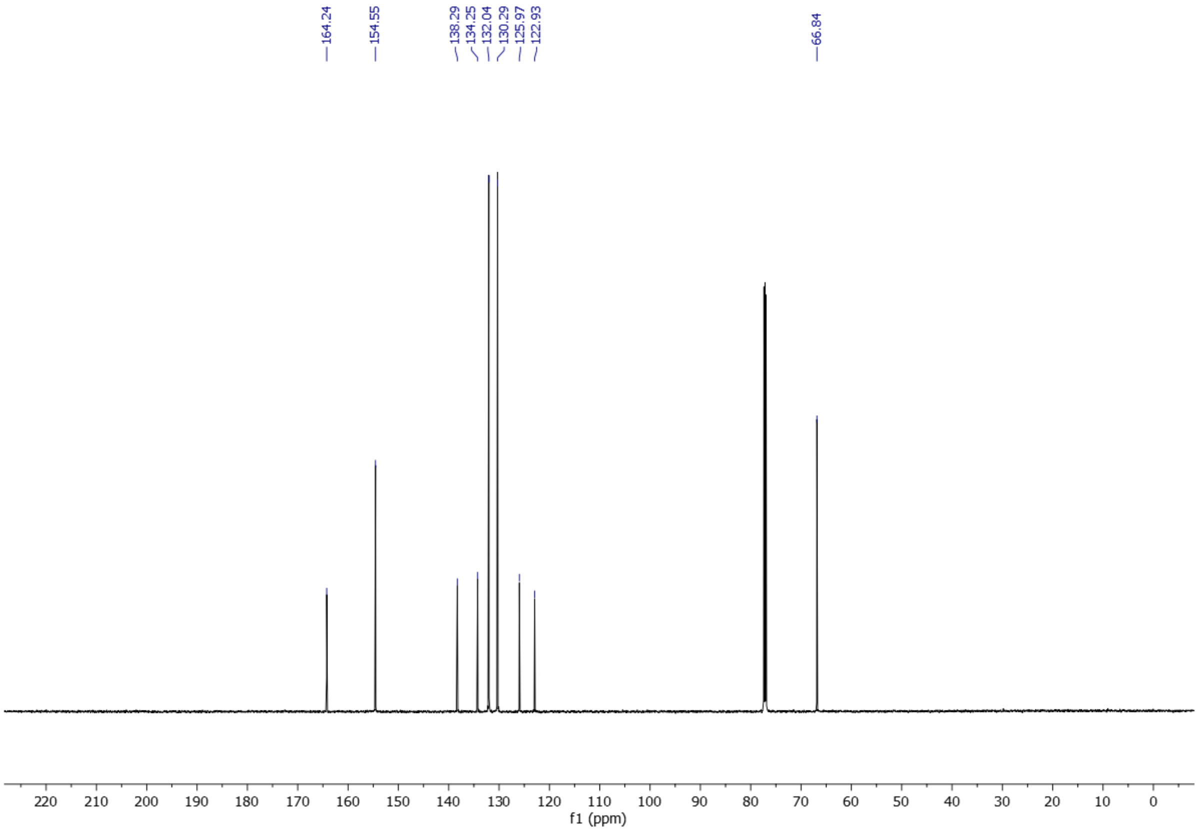

Figure S168. ^{1}H- and ^{13}C-NMR spectra of **bis(4-bromobenzyl) pyridine-3,5-dicarboxylate (S1)** in $CDCl_3$.

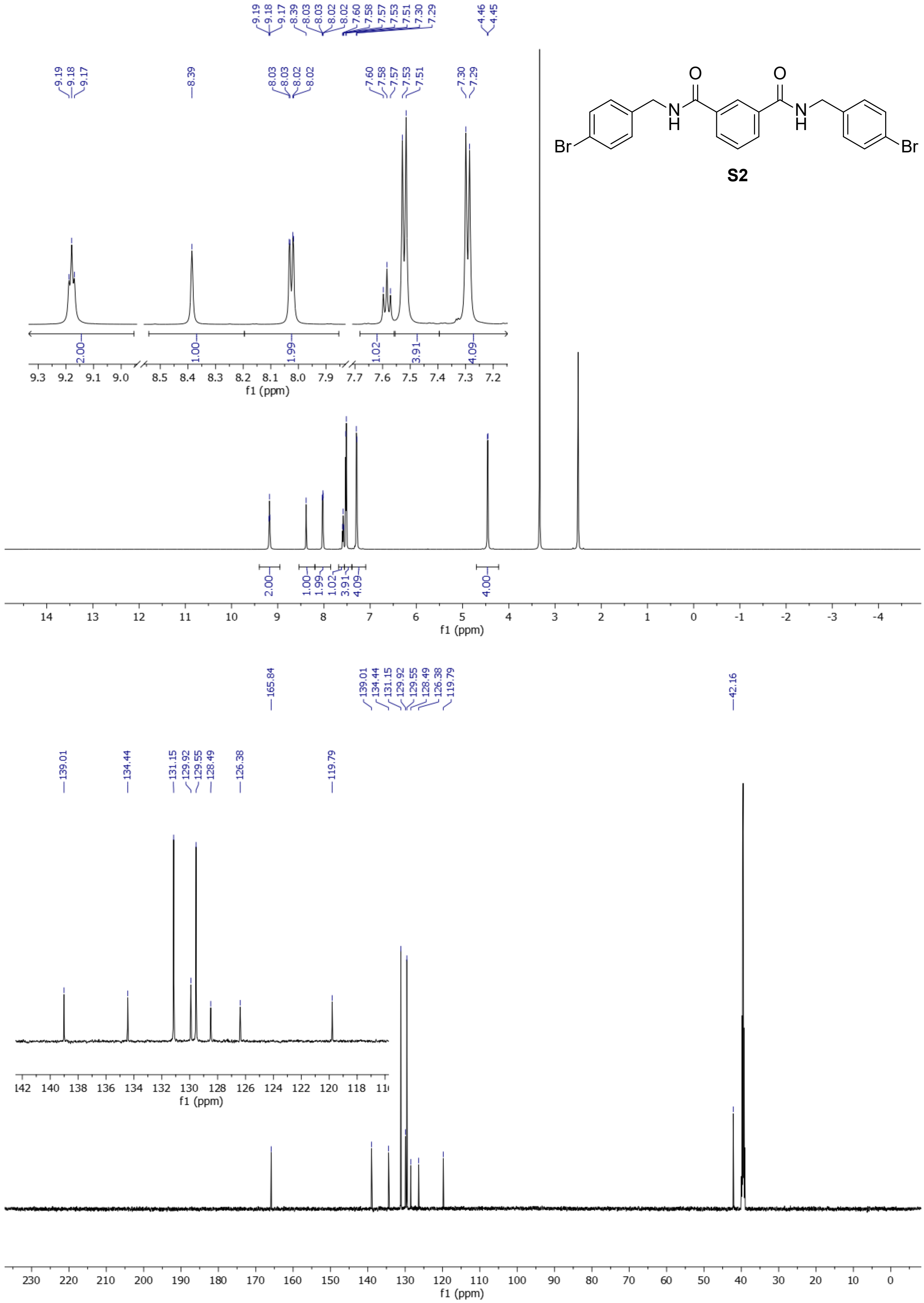

Figure S169. ^{1}H- and ^{13}C-NMR spectra of ***N*[1],*N*[3]-bis(4-bromobenzyl)isophthalamideate (S2)** in DMSO-d_6.

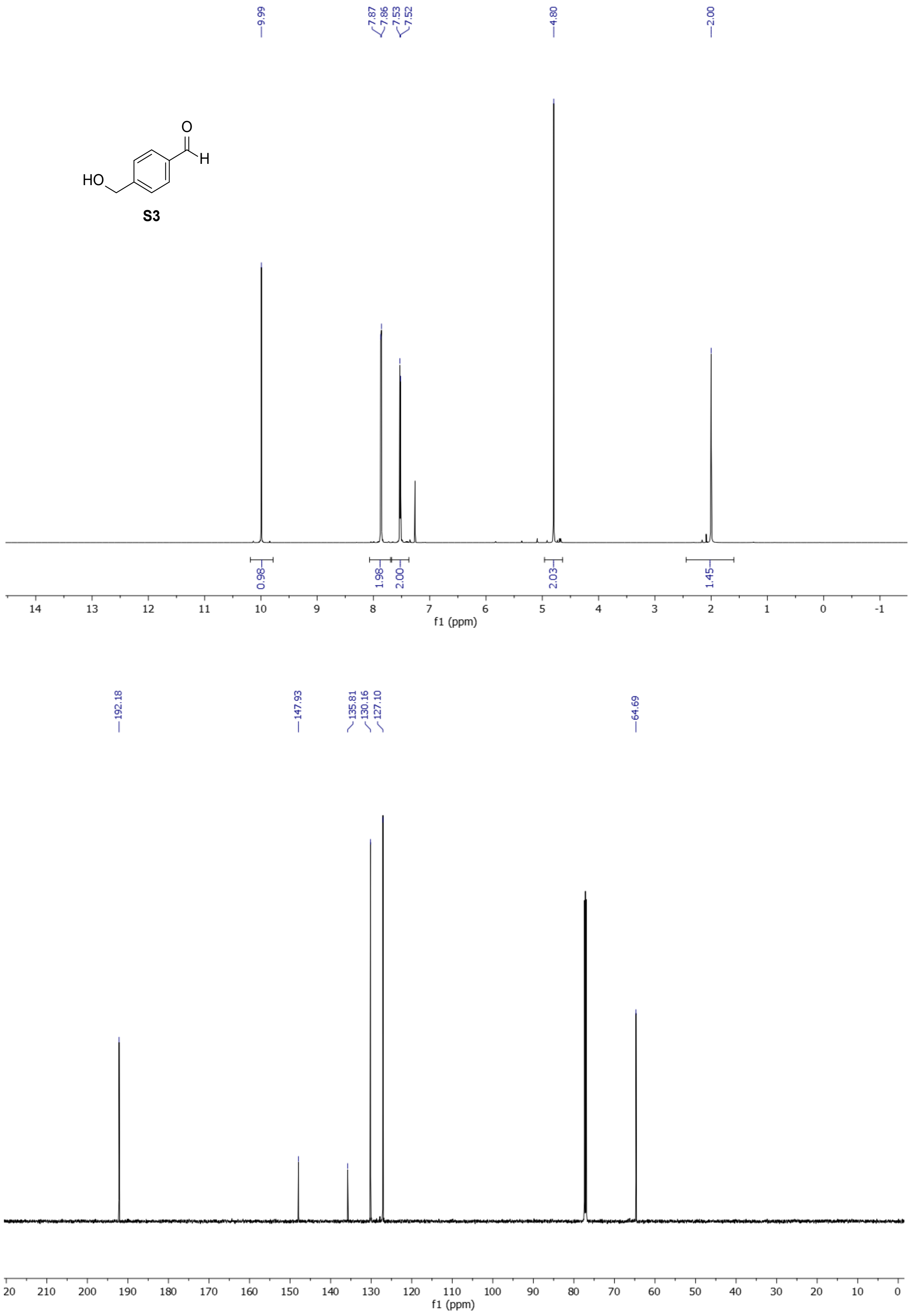

Figure S170. ^{1}H- and ^{13}C-NMR spectra of **4-(hydroxymethyl)benzaldehyde (S3)** in $CDCl_3$.

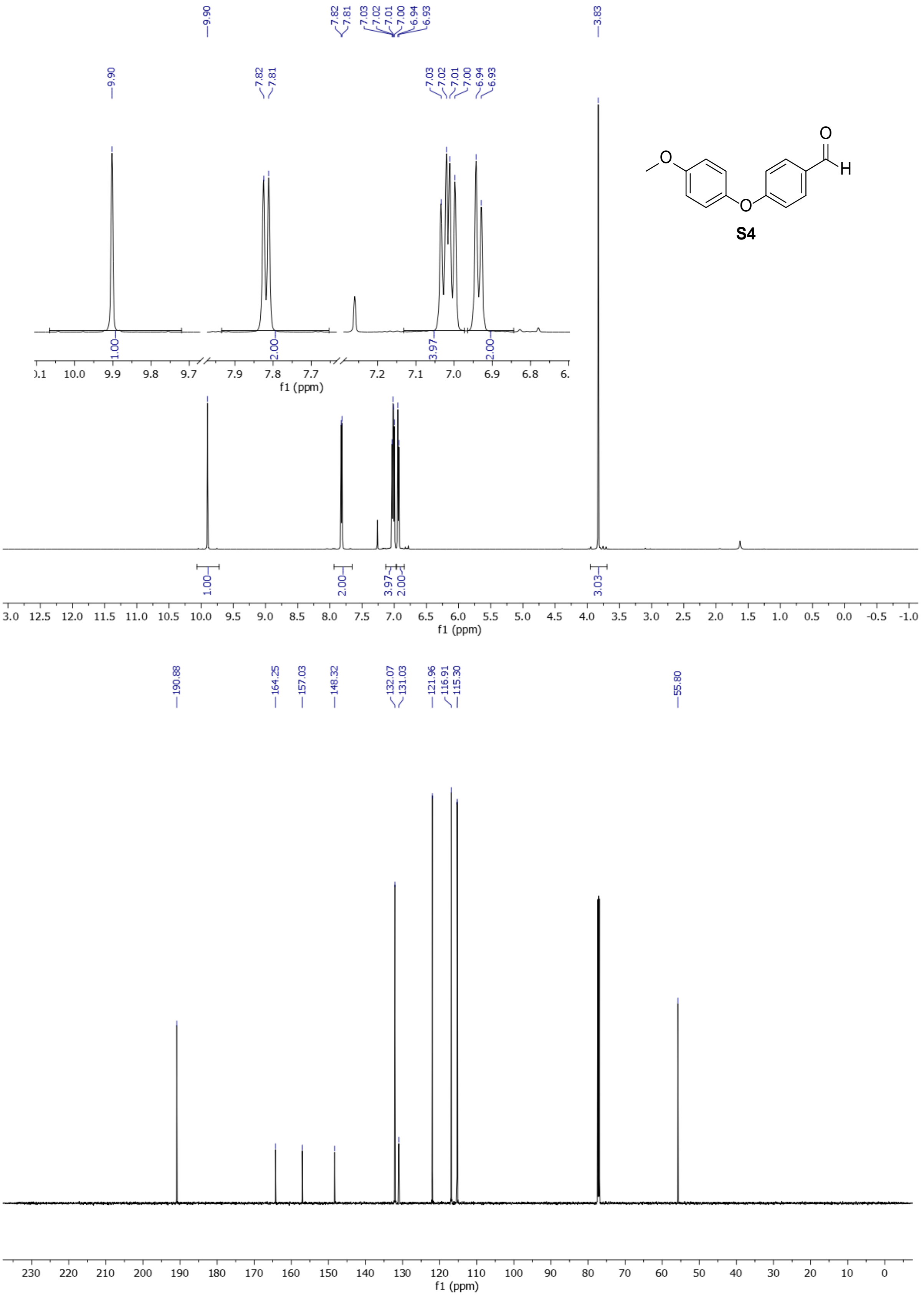

Figure S171. ^{1}H- and ^{13}C-NMR spectra of **4-(4-methoxyphenoxy)benzaldehyde (S4)** in $CDCl_3$.

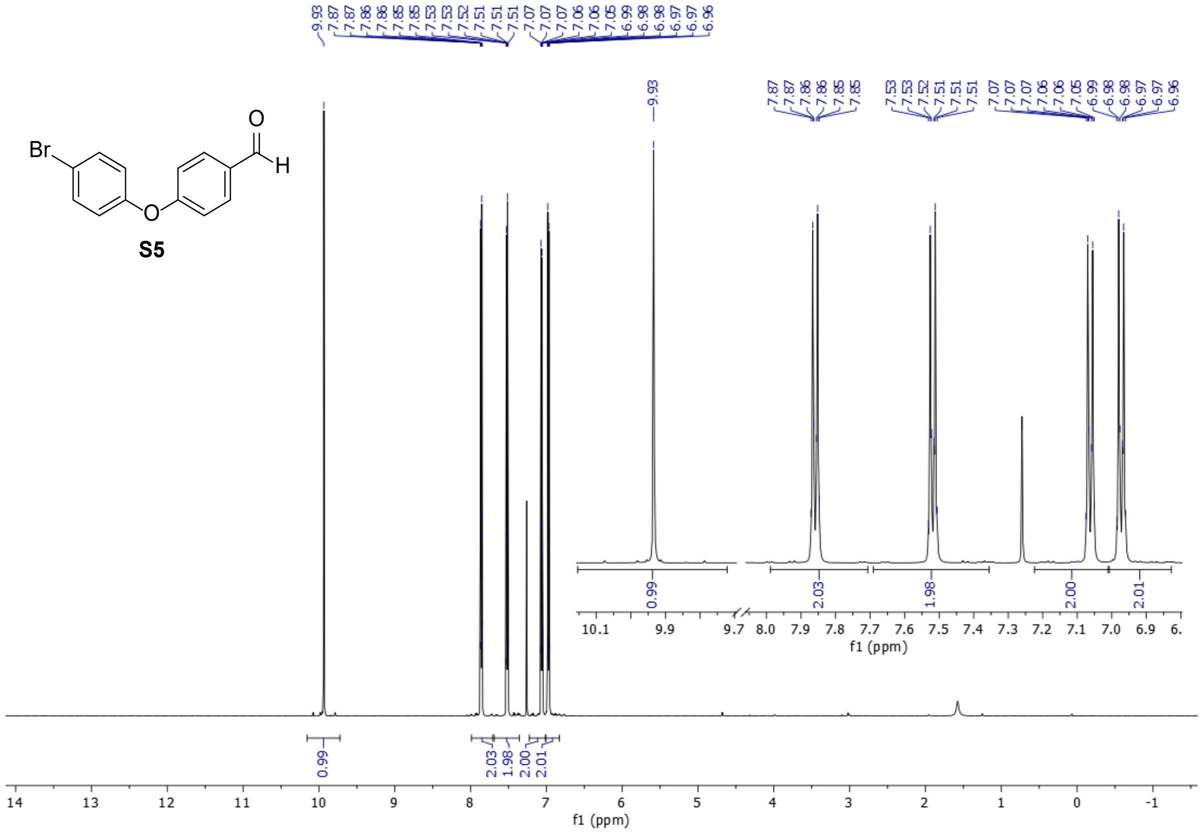

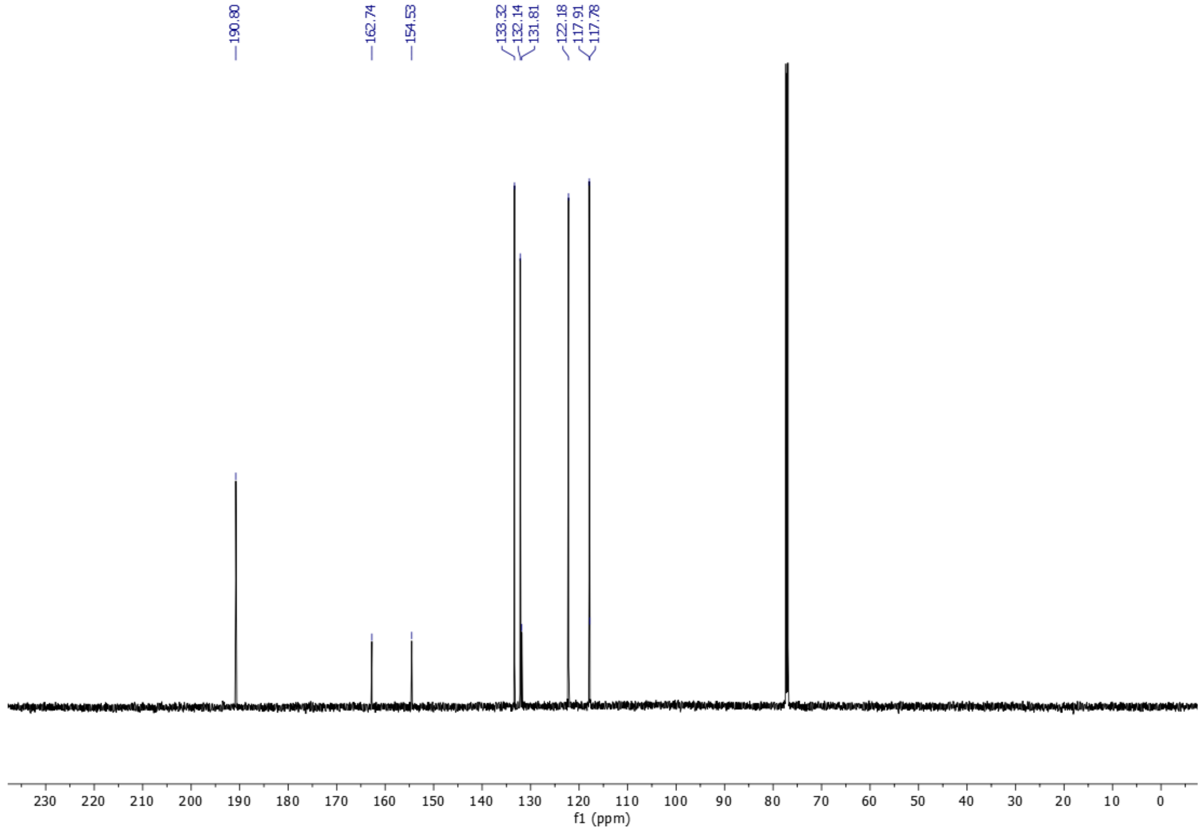

Figure S172. ^{1}H- and ^{13}C-NMR spectra of **4-(4-bromophenoxy)benzaldehyde (S5)** in $CDCl_3$.

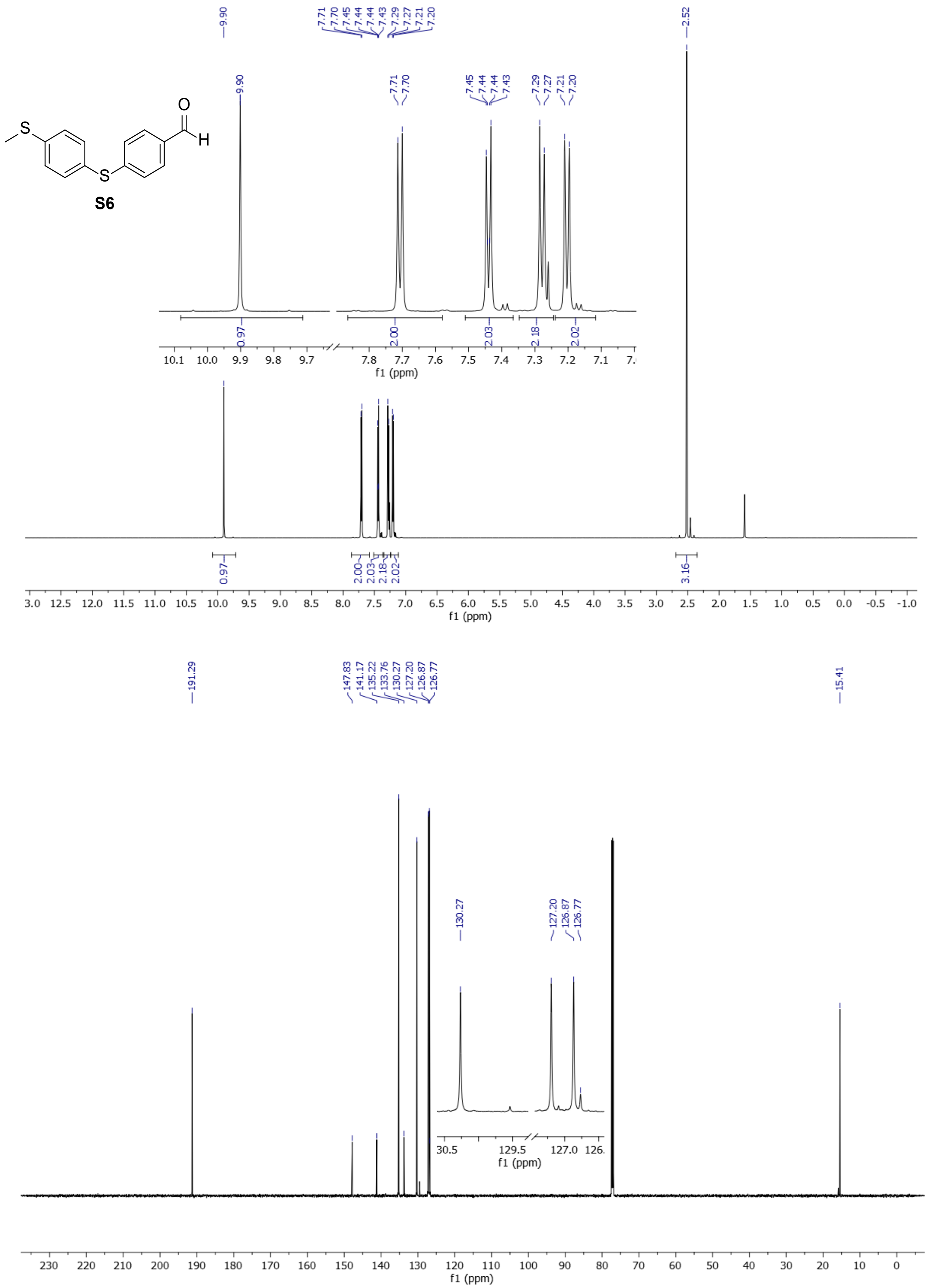

Figure S173. ^{1}H- and ^{13}C-NMR spectra of **4-((4-(methylthio)phenyl)thio)benzaldehyde (S6)** in $CDCl_3$.

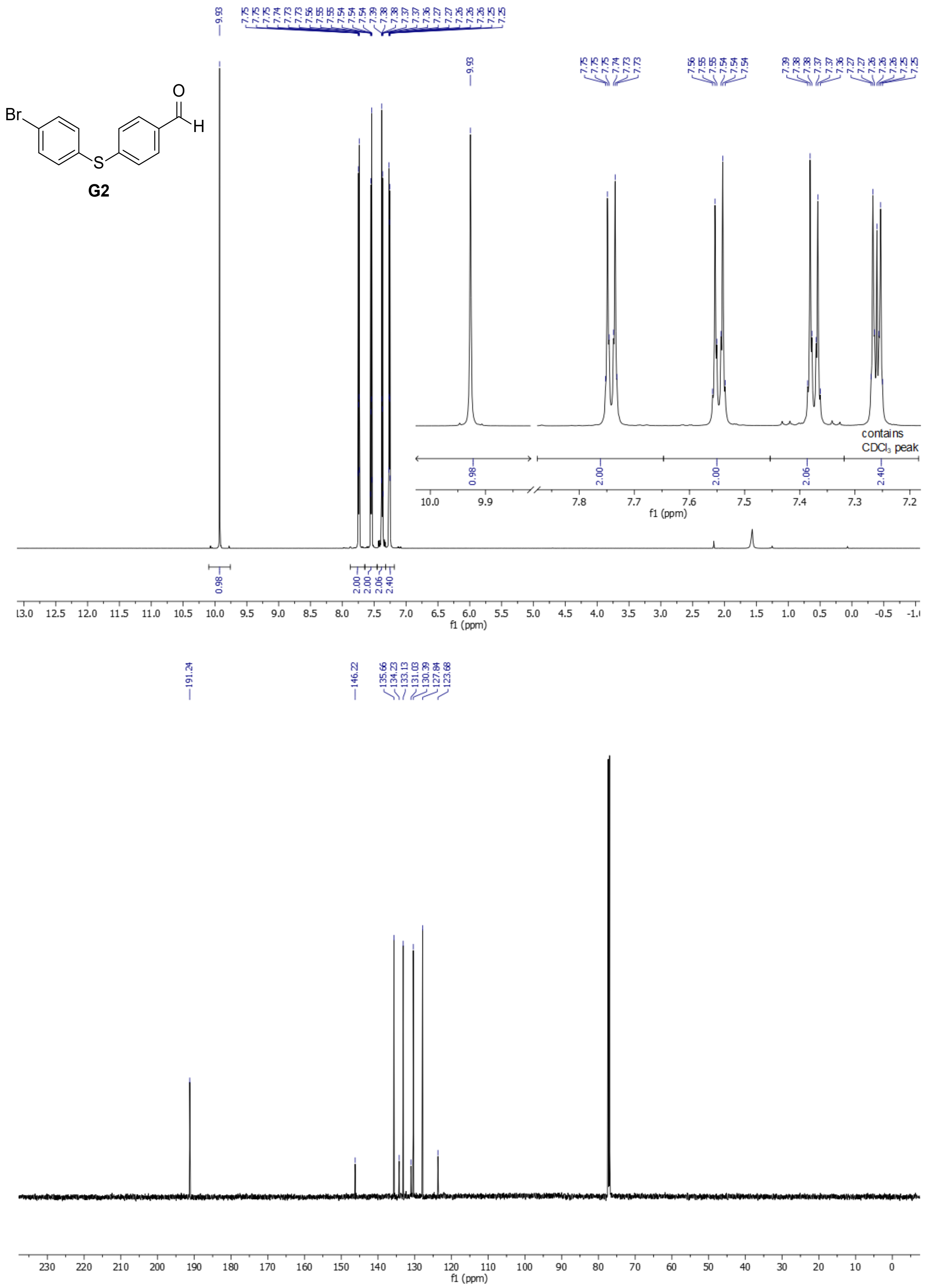

Figure S174. ^{1}H- and ^{13}C-NMR spectra of **4-((4-bromophenyl)thio)benzaldehyde (G2)** in $CDCl_3$.

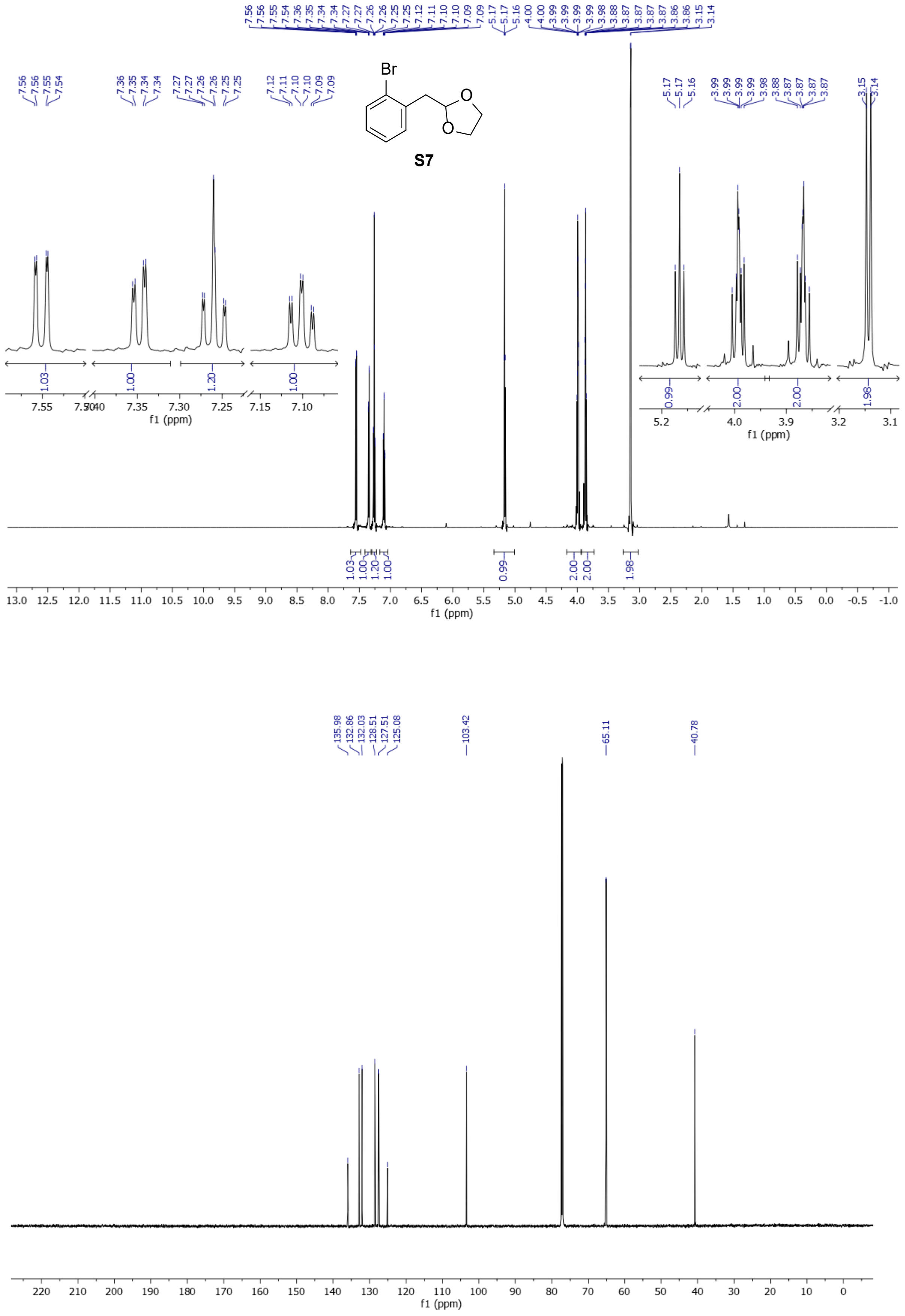

Figure S175. ^{1}H- and ^{13}C-NMR spectra of **2-(2-bromobenzyl)-1,3-dioxolane (S7)** in $CDCl_3$.

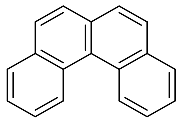

G10

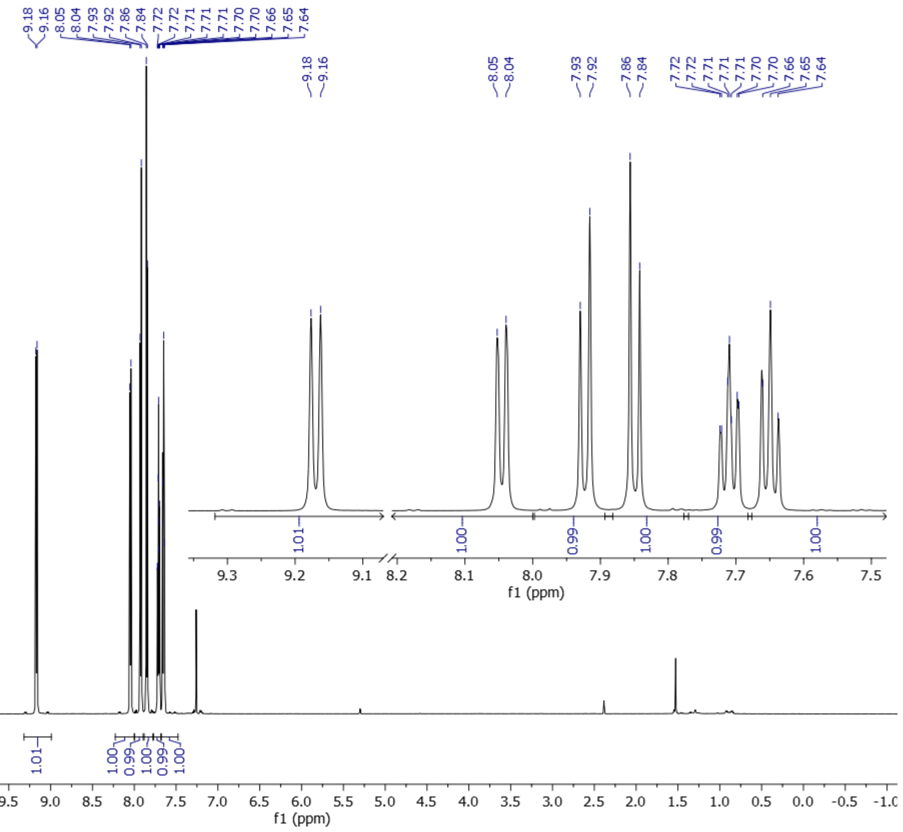

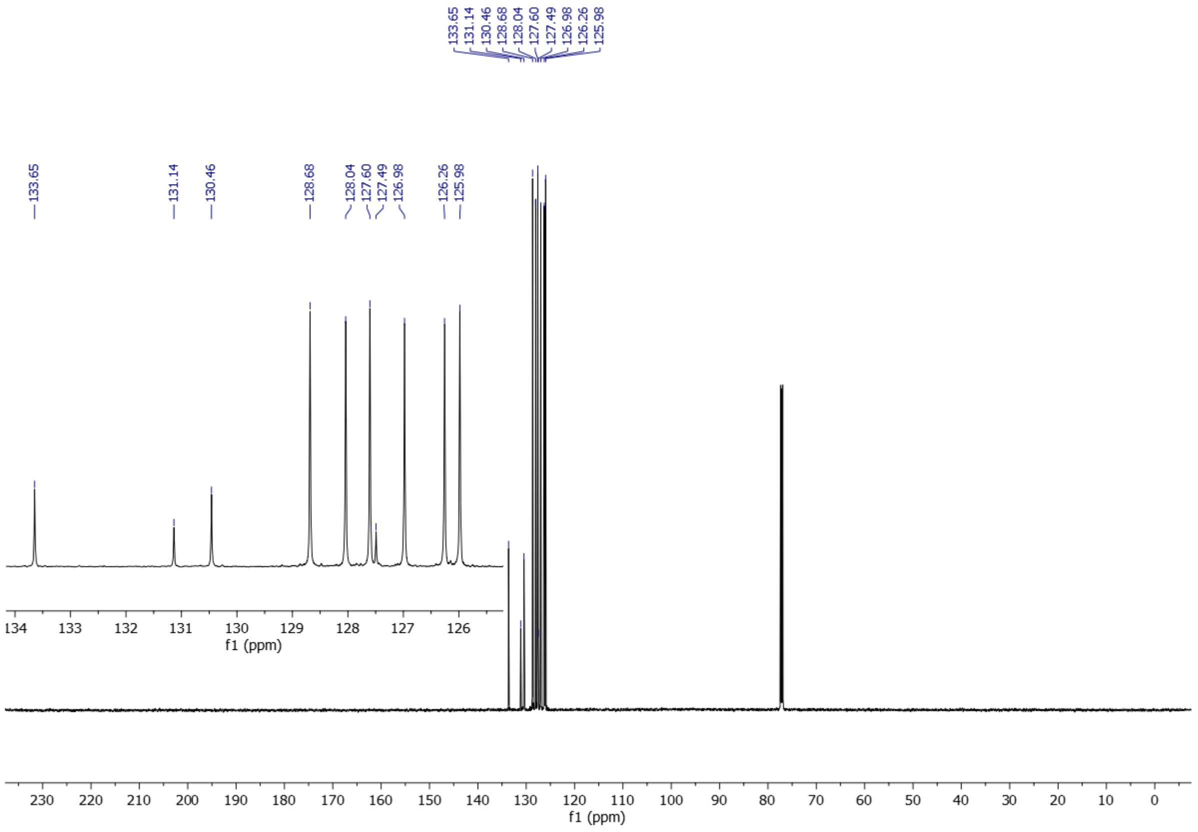

Figure S176. ^{1}H- and ^{13}C-NMR spectra of **benzo[*c*]phenanthrene (G10)** in $CDCl_3$.

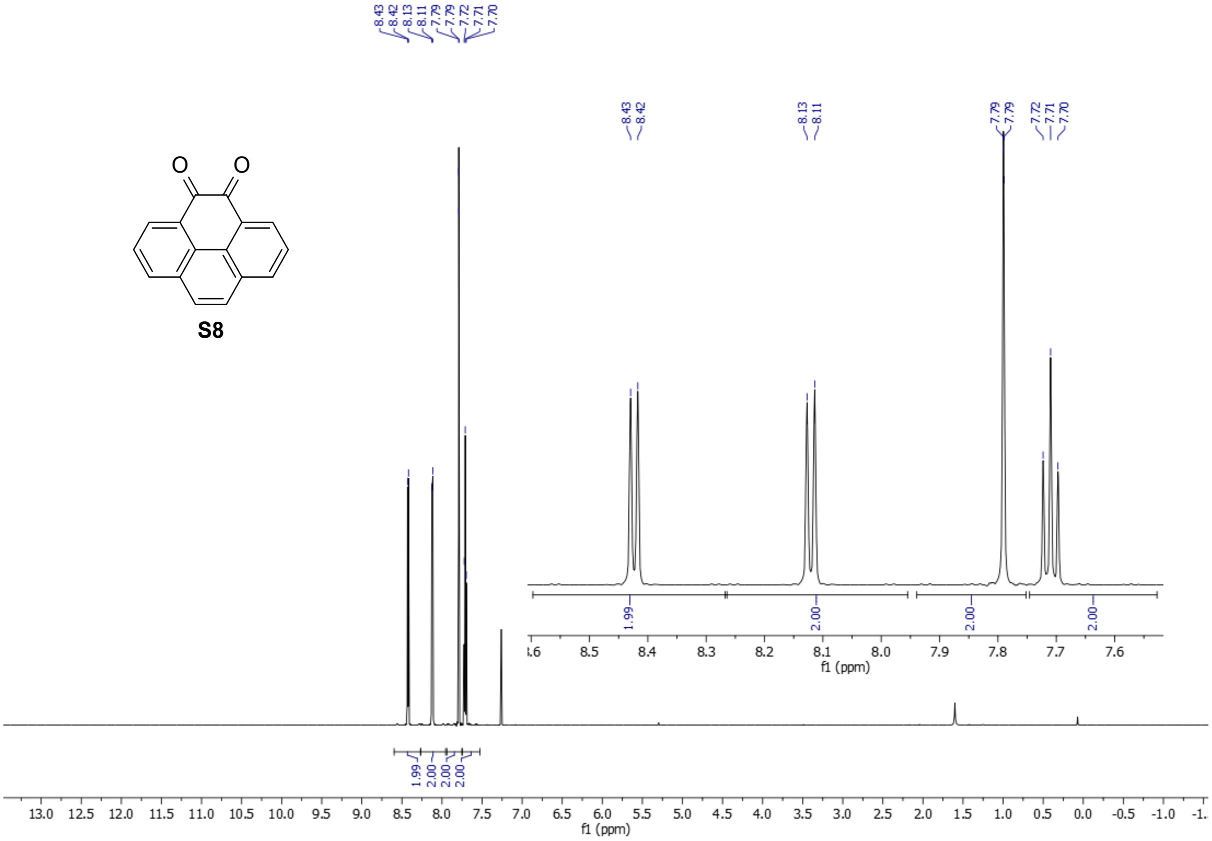

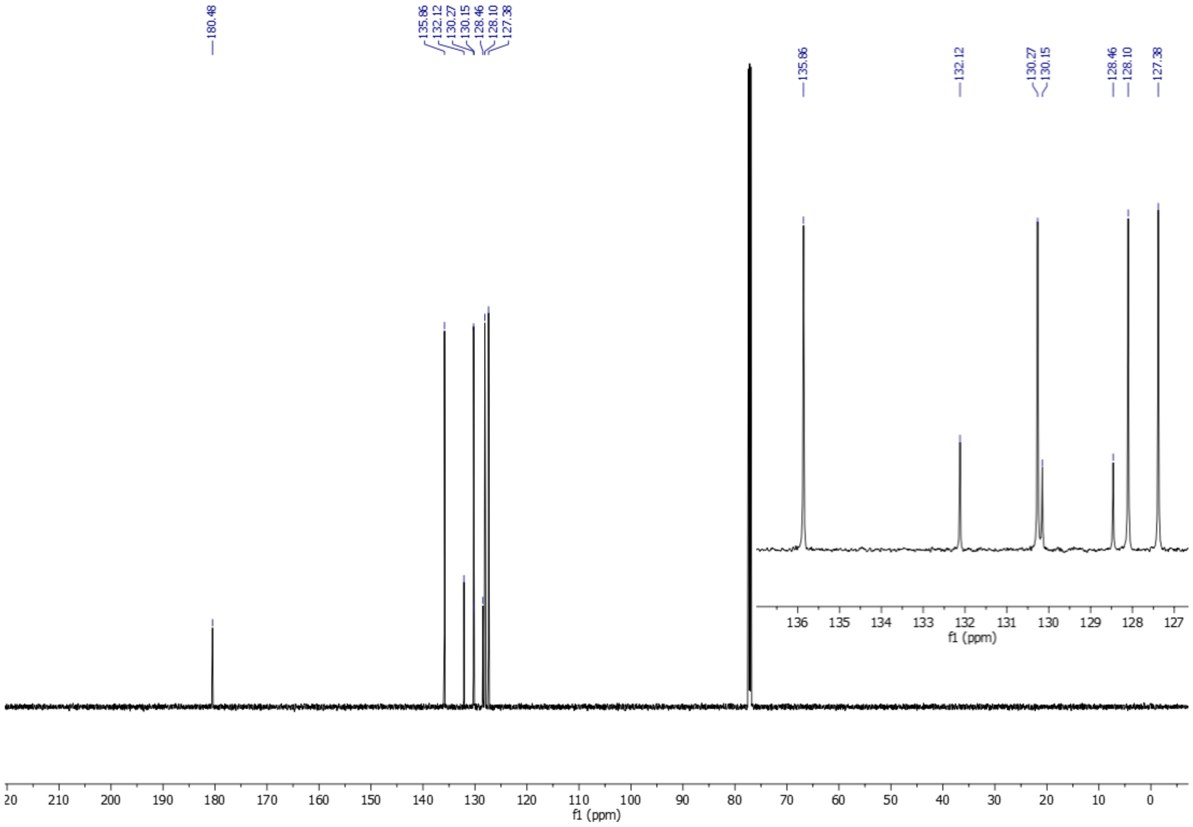

Figure S177. ^{1}H- and ^{13}C-NMR spectra of **pyrene-4,5-dione (S8)** in $CDCl_3$.

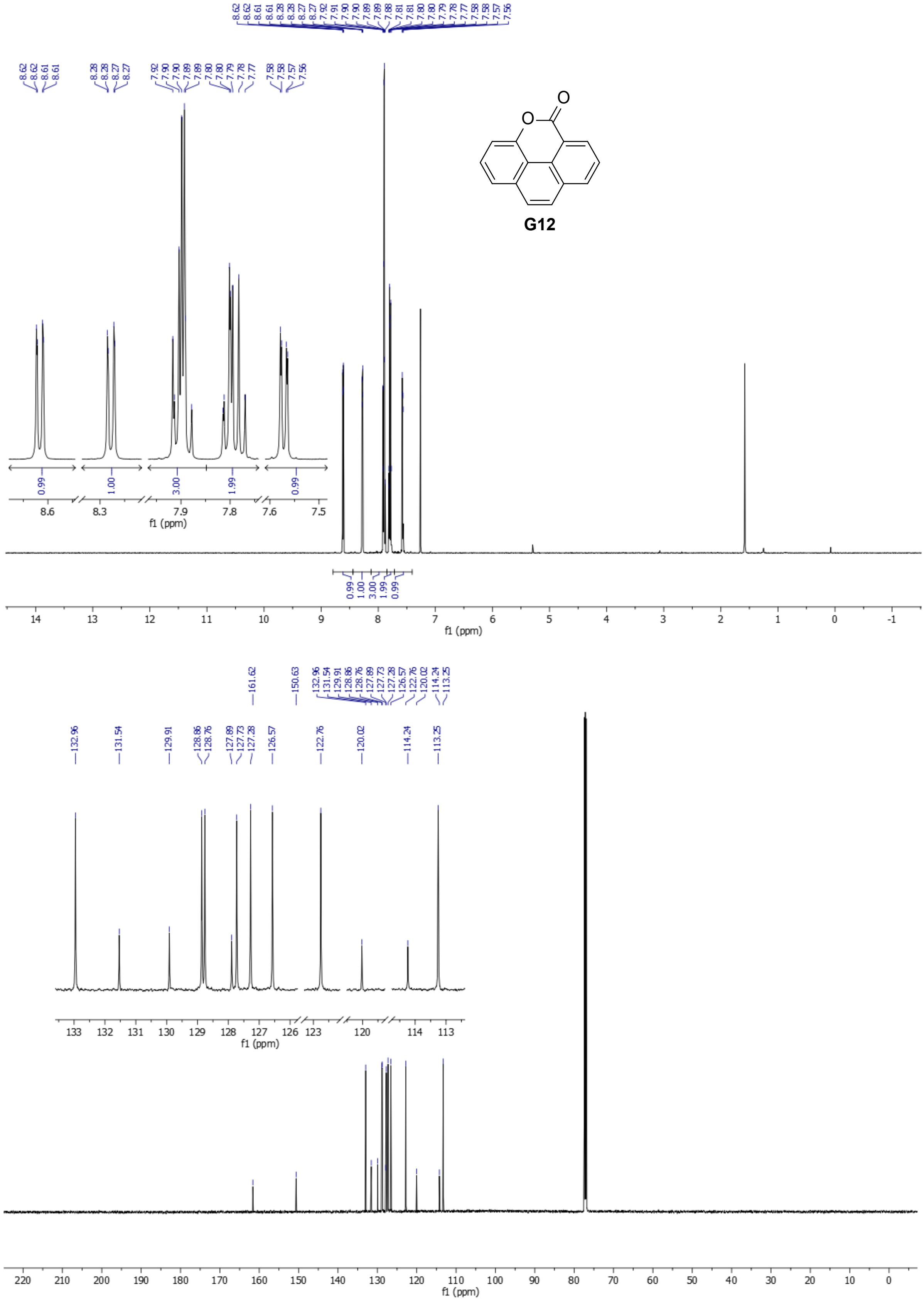

Figure S178. ^{1}H- and ^{13}C-NMR spectra of ***5H*-naphtho[8,1,2-*cde*]chromen-5-one (G12)** in $CDCl_3$.

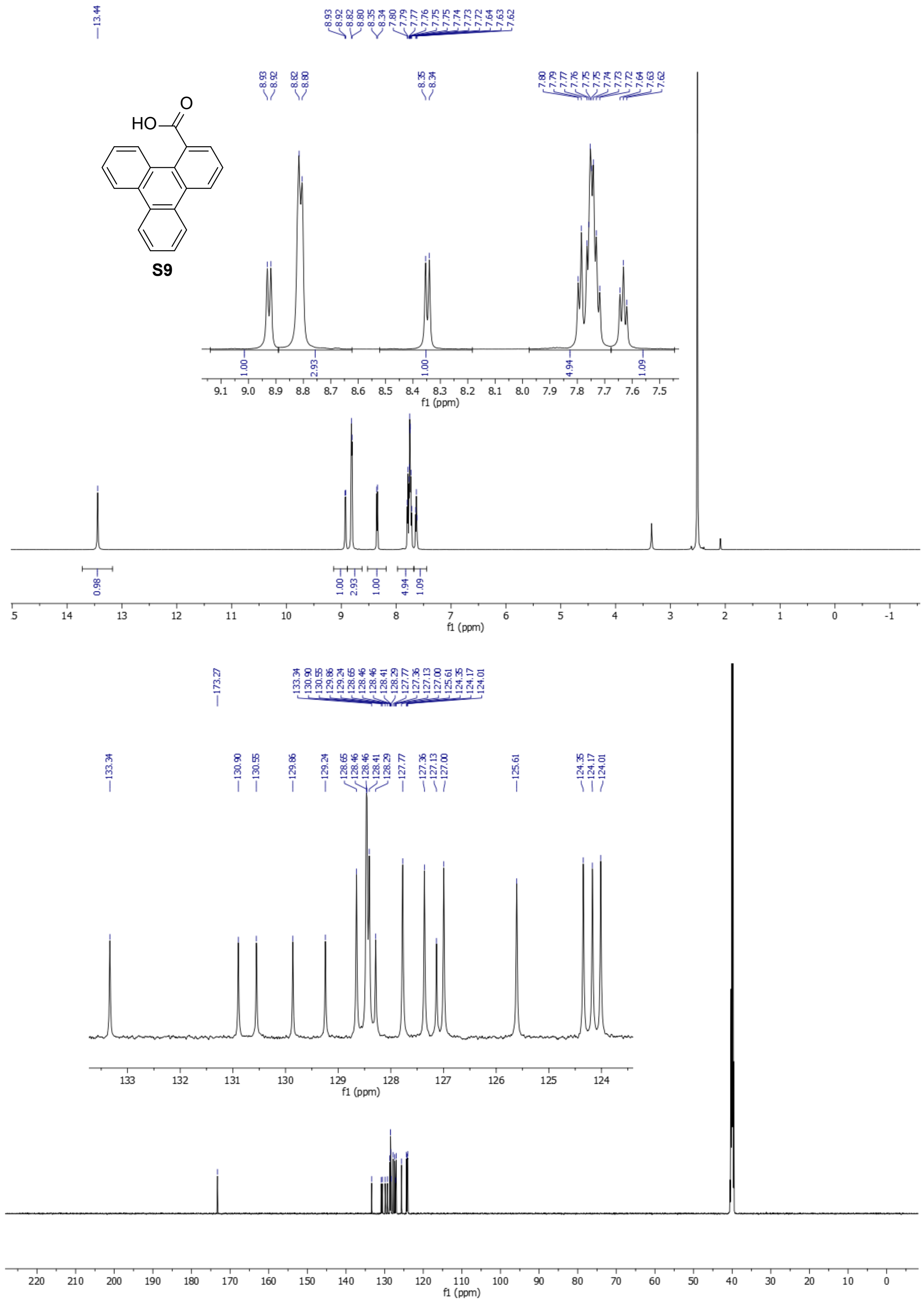

Figure S179. ^{1}H- and ^{13}C-NMR spectra of **triphenylene-1-carboxylic acid (S9)** in DMSO-d_6.

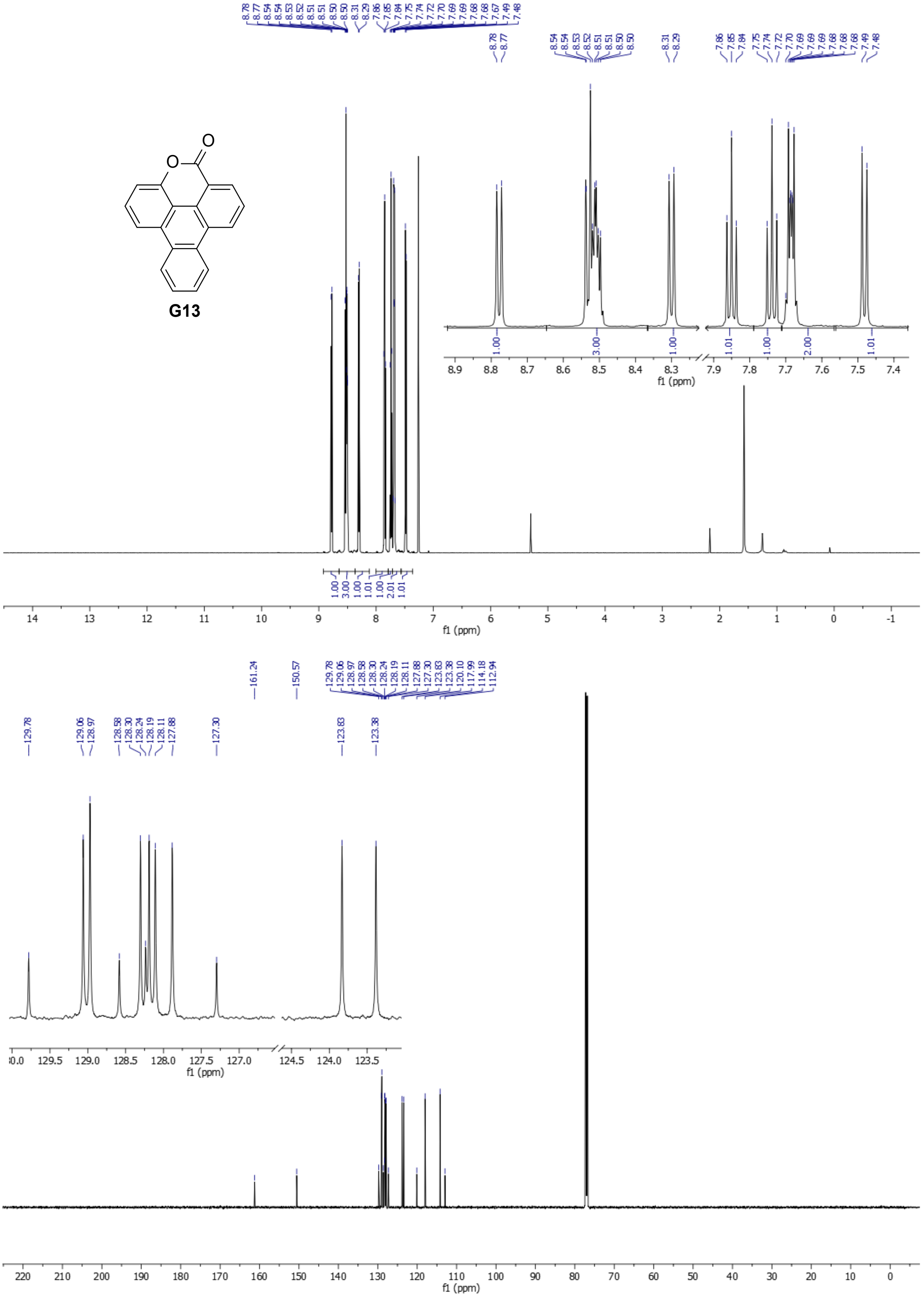

Figure S180. ^{1}H- and ^{13}C-NMR spectra of **5*H*-phenanthro[1,10,9-*cde*]chromen-5-one (G13)** in $CDCl_3$.

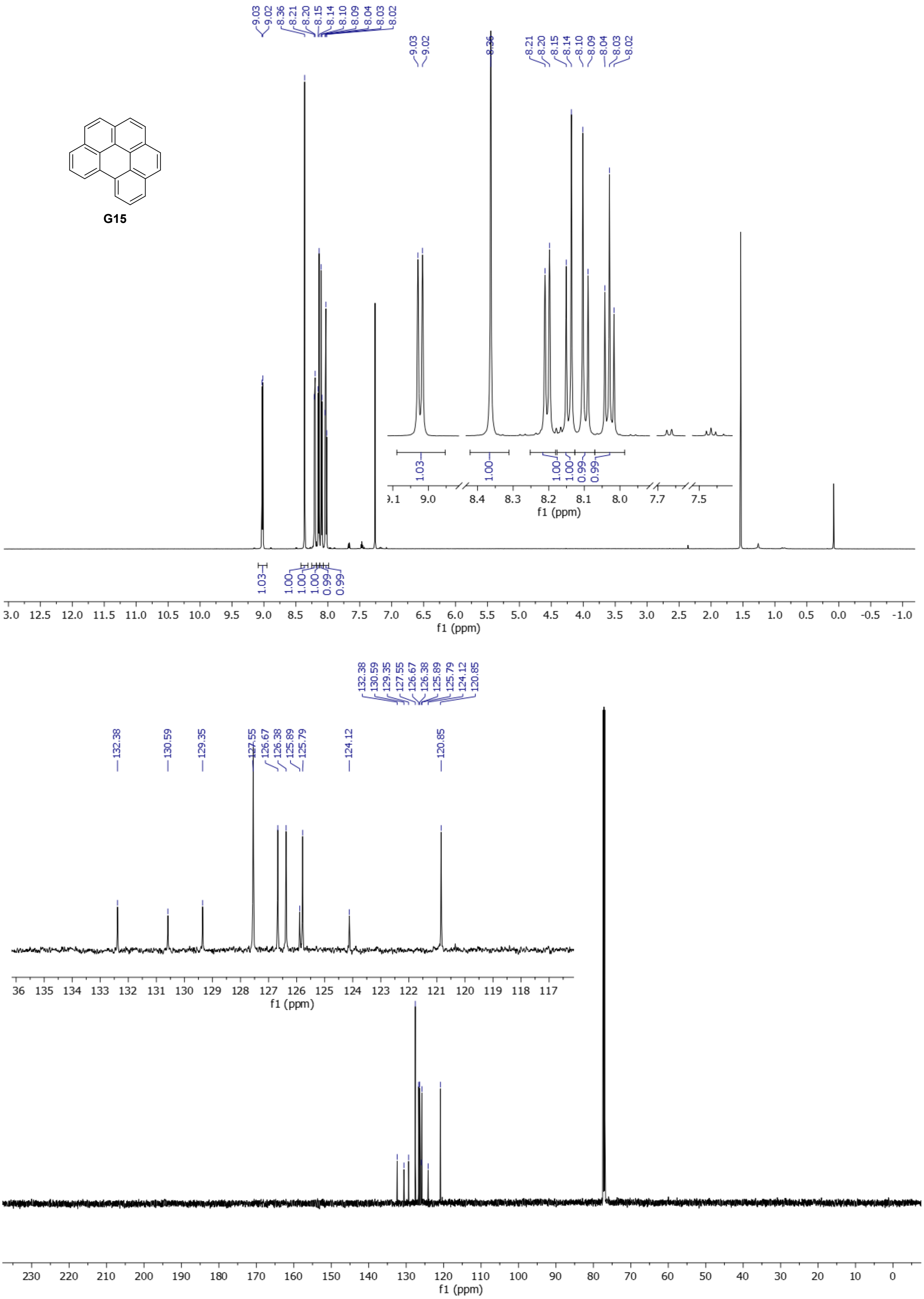

Figure S181. ^{1}H- and ^{13}C-NMR spectra of **benzo[*ghi*]perylene (G15)** in $CDCl_3$.

7 TD-DFT Computations

7.1 Computational Methodology

Geometry optimizations of the gas-phase structures of **guest**@4-Br DBI (**2e**) (guest = coronene (**3**), **G11**, **G12** and **G13**, Figure S182) were performed using density functional theory using the TPSSTPSS[32] functional and cc-pVTZ[33,34] basis set. Dispersion effects were modeled using Grimme's GD3BJ parameters.[35] Subsequent frequency analysis confirmed all obtained structures to be local minima on the potential energy surface. To calculate phosphorescence energies, the geometries of the lowest lying triplet states (T_1) were optimized by using UTPSSTPSS (spin-unrestricted TPSSTPSS) with the same basis sets as specified above. The vertical excitation energies of the first ten singlet and triplet states have been predicted by TD-DFT computations using the TPSSTPSS functional as well as the cc-pVTZ basis sets using the respective optimized gas-phase S_0 geometry. Phosphorescence energies have been calculated as the difference of the energies at the UTPSSTPSS-optimized T_1 geometry and the TPSSTPSS-optimized S_0 geometry. All computations have been carried out with the Gaussian16 software.[36] The presented molecular orbitals (MOs) were extracted from the Gaussian16 checkpoint-files and are visualized with VMD.[37]

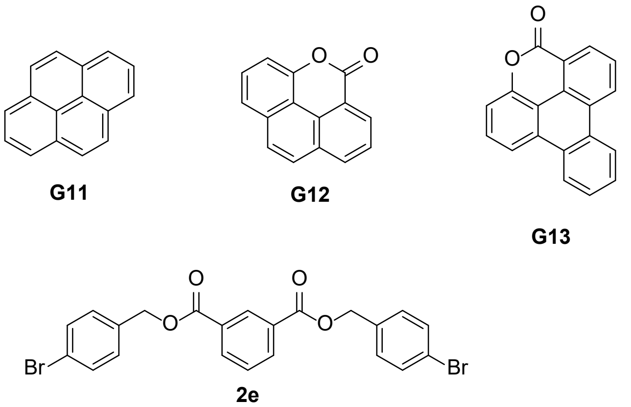

G11 G12 G13

2e

Figure S182. Guests and host used in TD-DFT calculations.

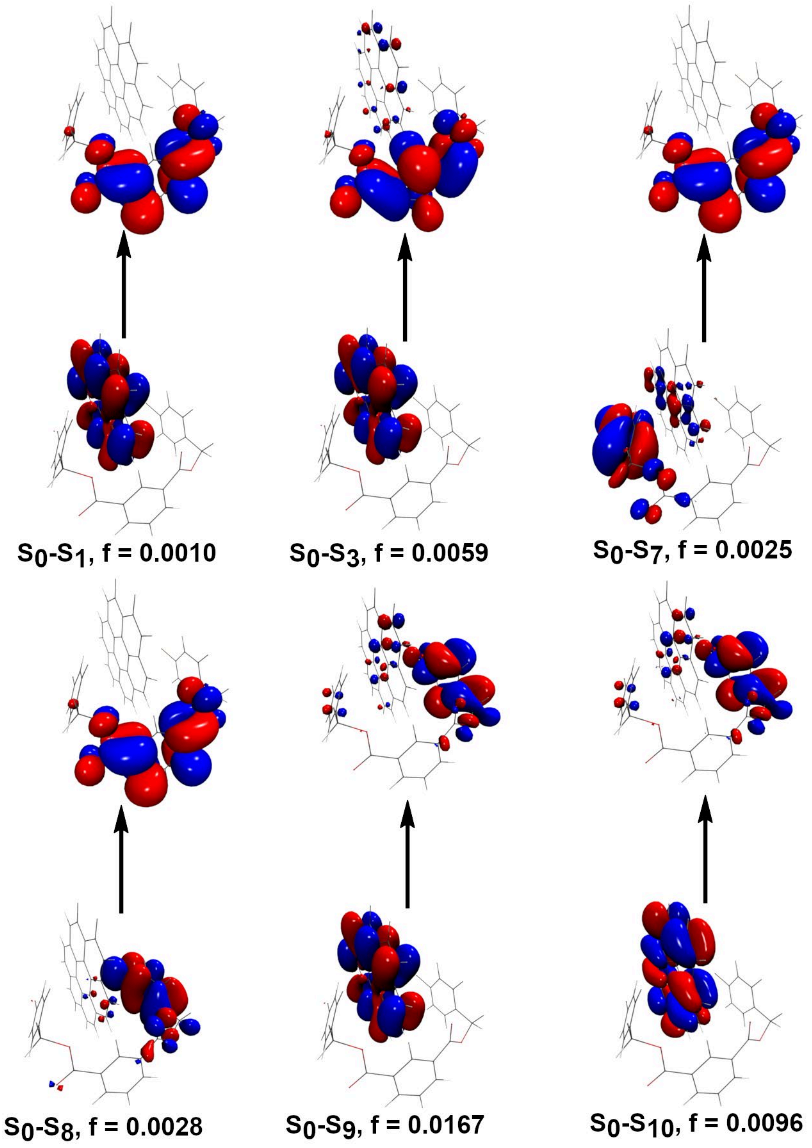

Figure S183. Natural transition orbitals (NTO) for TD-DFT computed $S_0 \rightarrow S_n$ transitions with oscillator strengths f ≥ 0.0010 of coronene (**3**)@4-Br DBI (**2e**).

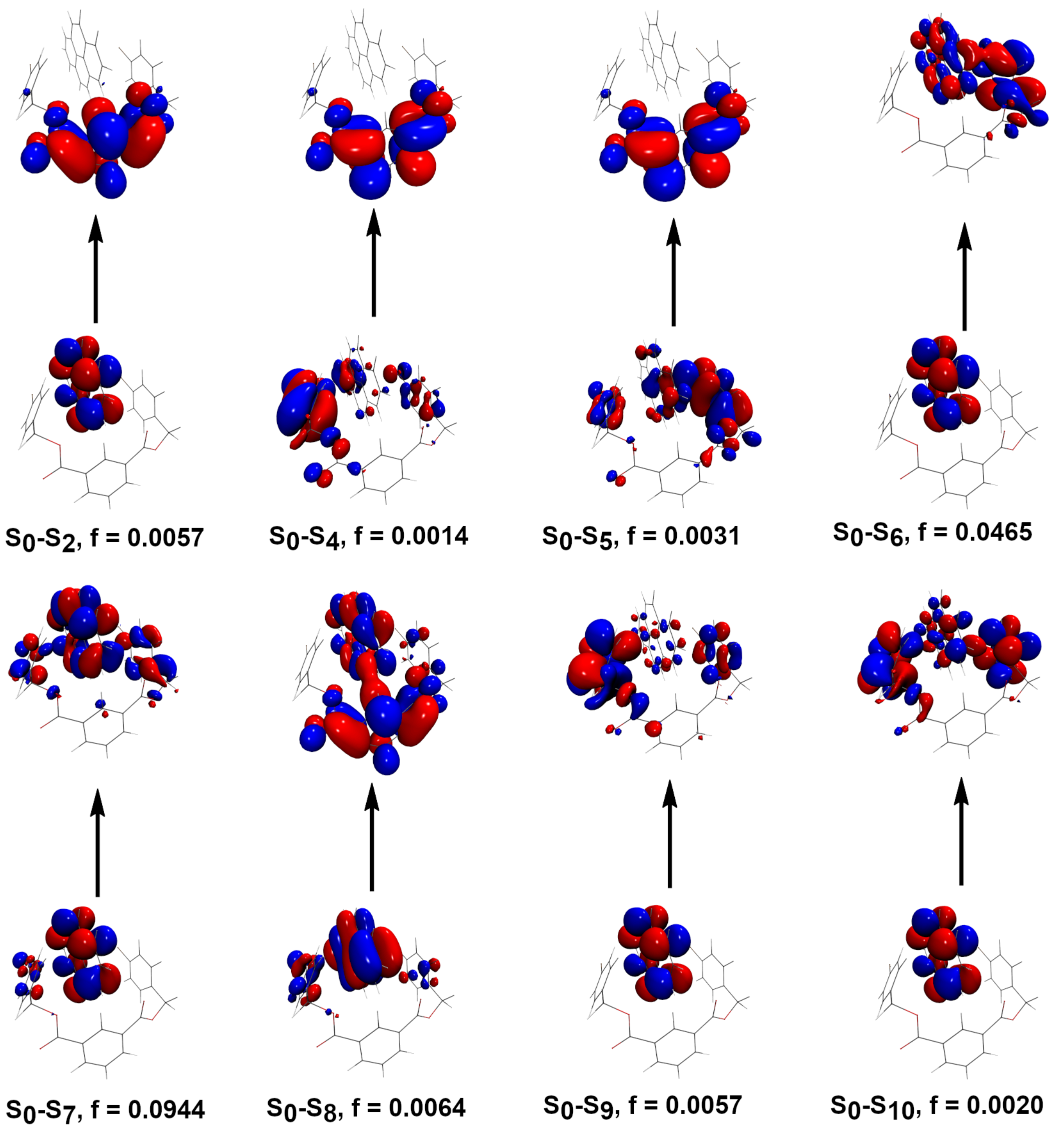

Figure S184. Natural transition orbitals (NTO) for TD-DFT computed $S_0 \rightarrow S_n$ transitions with oscillator strengths $f \geq 0.0010$ of pyrene (**G11**)@4-Br DBI (**2e**).

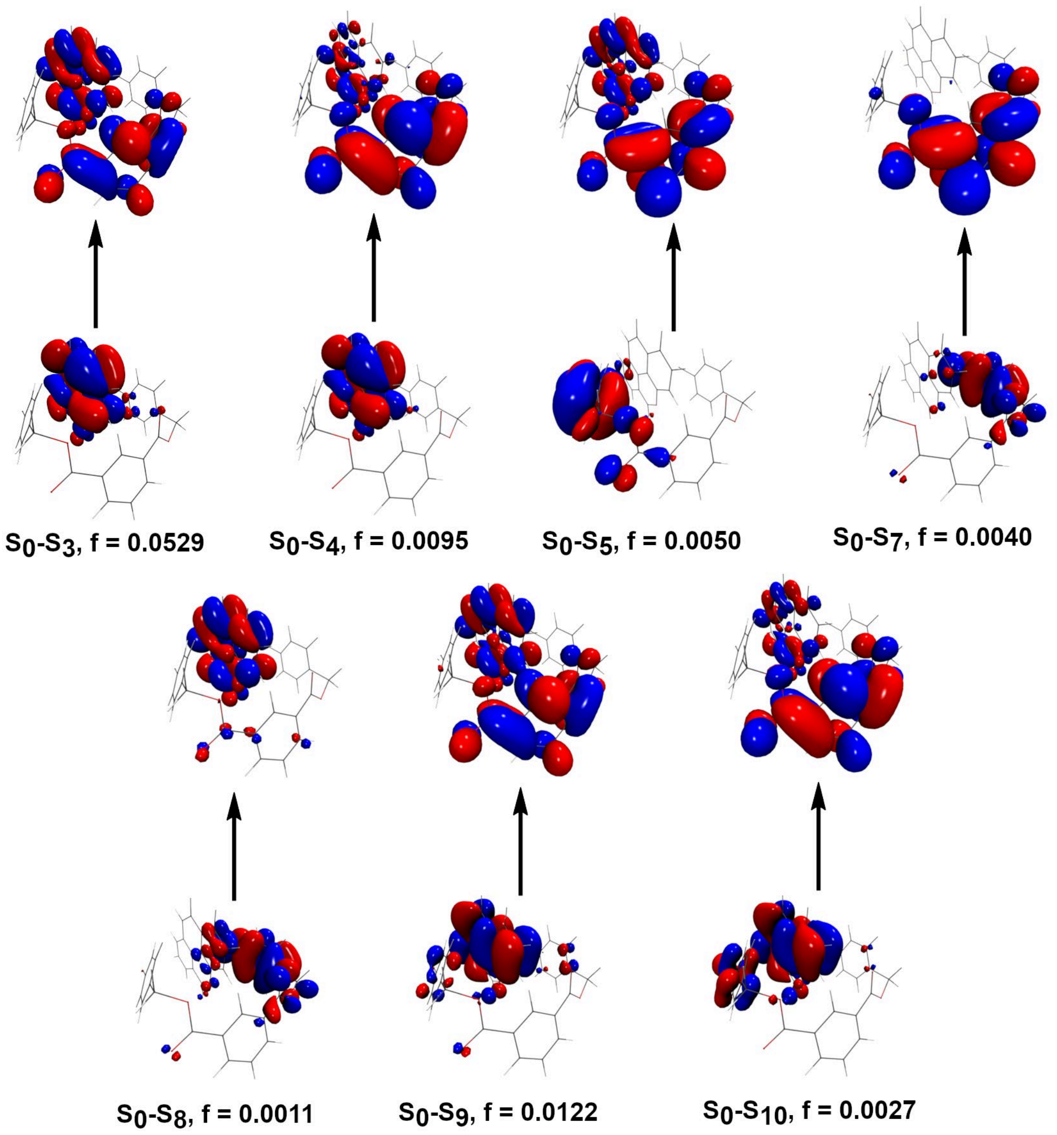

Figure S185. Natural transition orbitals (NTO) for TD-DFT computed $S_0 \rightarrow S_n$ transitions with oscillator strengths f ≥ 0.0010 of **G12**@4-Br DBI (**2e**).

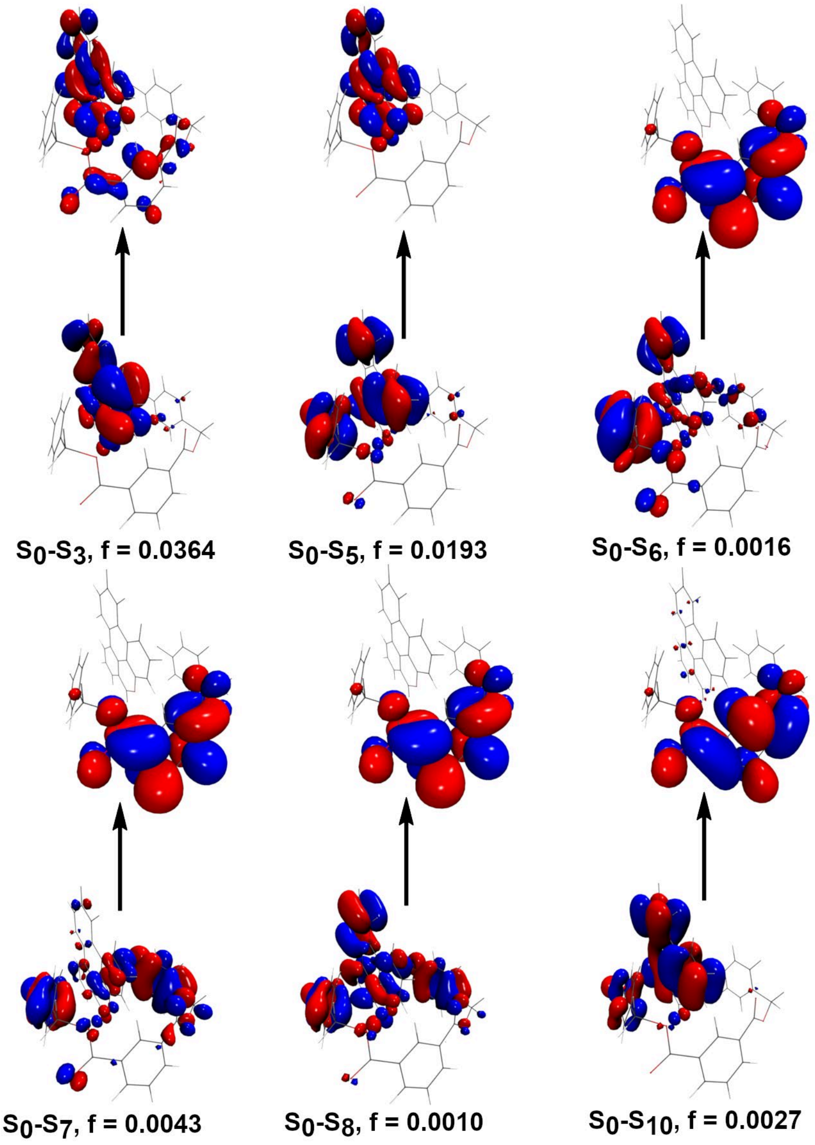

Figure S186. Natural transition orbitals (NTO) for TD-DFT computed $S_0 \rightarrow S_n$ transitions with oscillator strengths f ≥ 0.0010 of **G13**@4-Br DBI (**2e**).

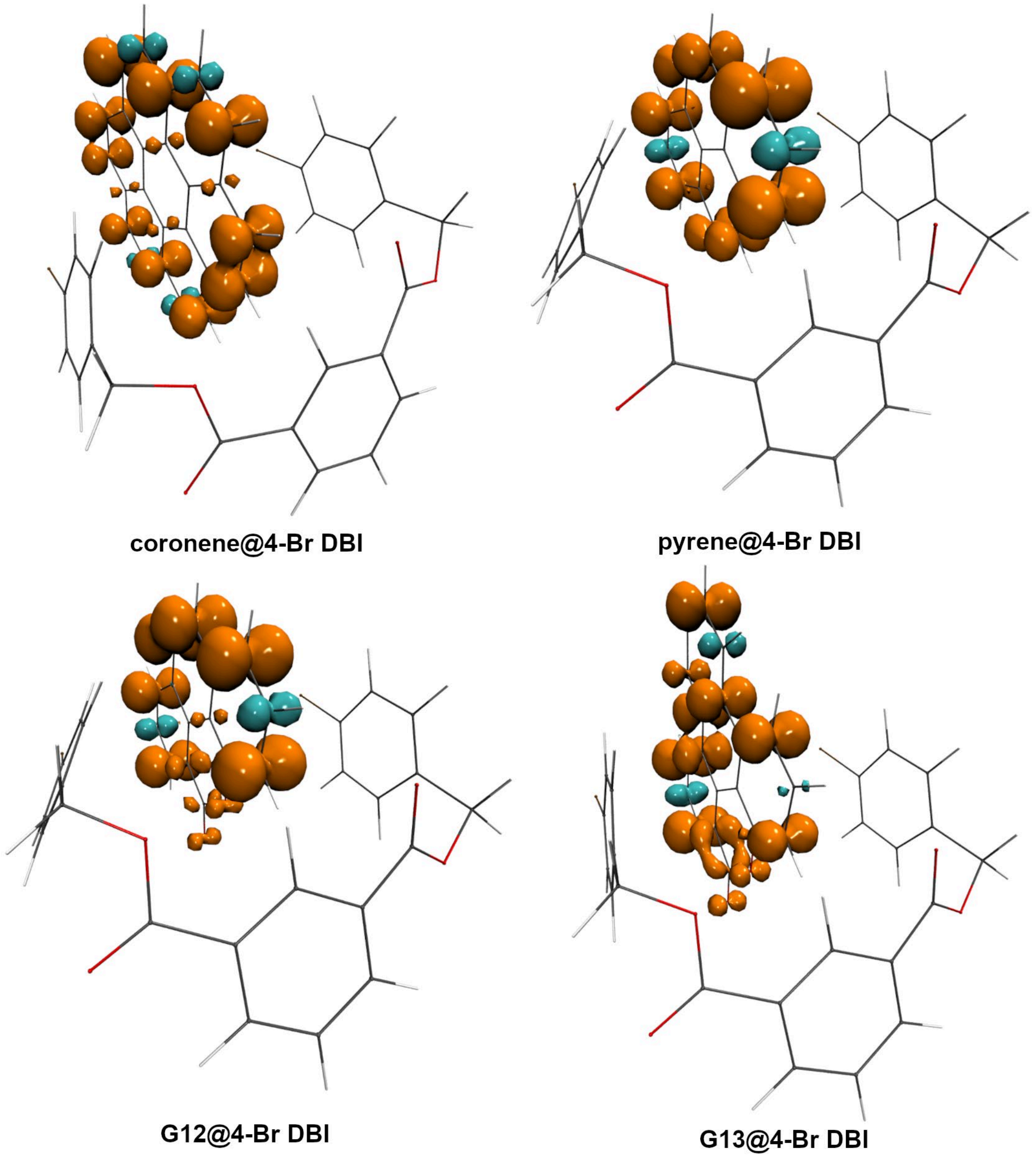

coronene@4-Br DBI **pyrene@4-Br DBI**

G12@4-Br DBI **G13@4-Br DBI**

Figure S187. Spin density of the T_1 states of **guest**@4-Br DBI (**2e**) (guest = coronene (**3**, top left), pyrene (**G11**, top right), **G12** (bottom left) and **G13** (bottom right)), representation at +0.005/–0.005 e bohr^{-3} (orange/cyan).

Table S8. TD-DFT computed excited states of for **guest**@4-Br DBI (**2e**) (**guest** = coronene (**3**), pyrene (**G11**), **G12** and **G13**) using the TPSSTPSS/cc-pVTZ level of theory.

**Coronene (3)**		**G11 (pyrene)**		**G12**		**G13**	
Ex. States	E [eV] and f	Ex. States	E [eV] and f	Ex. States	E [eV] and f	Ex. States	E [eV] and f
T_1	2.3390 0.0000	T_1	2.1192 0.0000	T_1	2.4992 0.0000	T_1	2.7024 0.0000
T_2	2.4454 0.0000	T_2	2.3260 0.0000	T_2	2.8712 0.0000	T_2	2.9168 0.0000
S_1	2.4456 0.0010	S_1	2.3272 0.0004	S_1	2.8735 0.0003	S_1	2.9215 0.0004
T_3	2.4569 0.0000	S_2	2.7645 0.0057	T_3	2.9538 0.0000	T_3	2.9615 0.0000
S_2	2.4581 0.0003	T_3	2.7692 0.0000	T_4	3.1588 0.0000	T_4	3.1181 0.0000
T_4	2.7419 0.0000	T_4	3.0993 0.0000	T_5	3.2172 0.0000	S_2	3.1253 0.0006
T_5	2.7433 0.0000	S_3	3.1027 0.0003	S_2	3.2217 0.0006	T_5	3.1445 0.0000
T_6	2.8478 0.0000	T_5	3.2180 0.0000	S_3	3.2618 0.0529	T_6	3.1997 0.0000
S_3	2.8855 0.0059	T_6	3.2683 0.0000	T_6	3.3050 0.0000	S_3	3.2920 0.0364
S_4	2.9018 0.0004	S_4	3.2879 0.0014	S_4	3.3182 0.0095	T_7	3.3305 0.0000
T_7	2.9028 0.0000	T_7	3.2982 0.0000	T_7	3.3502 0.0000	T_8	3.3489 0.0000
T_8	2.9154 0.0000	T_8	3.3229 0.0000	T_8	3.4101 0.0000	S_4	3.3490 0.0000
S_5	2.9784 0.0002	S_5	3.3367 0.0031	S_5	3.4259 0.0050	T_9	3.3650 0.0000
S_6	3.1501 0.0001	S_6	3.3618 0.0465	S_6	3.4388 0.0005	T_{10}	3.3826 0.0000
T_9	3.2149 0.0000	S_7	3.4031 0.0944	S_7	3.4443 0.0040	S_5	3.3867 0.0193
S_7	3.2347 0.0025	T_9	3.4041 0.0000	T_9	3.4552 0.0000	S_6	3.3999 0.0016
T_{10}	3.3016 0.0000	T_{10}	3.4114 0.0000	T_{10}	3.4569 0.0000	S_7	3.4487 0.0043
S_8	3.3382 0.0028	S_8	3.5028 0.0064	S_8	3.5108 0.0011	S_8	3.4757 0.0010
S_9	3.5205 0.0167	S_9	3.5411 0.0057	S_9	3.6290 0.0122	S_9	3.5468 0.0008
S_{10}	3.5367 0.0096	S_{10}	3.5683 0.0020	S_{10}	3.6698 0.0027	S_{10}	3.5541 0.0027

Table S9. Calculated adiabatic (E_{adia}) and zero-point energy corrected adiabatic energies (E_{0-0}) for **guest**@4-Br DBI (**2e**) (guest = coronene (**3**), pyrene (**G11**), **G12** and **G13**).

Energy [eV]	Coronene	G11 (pyrene)	G12	G13
S_0	–7220.2569 a.u.	–6913.9500 a.u.	–7025.1460 a.u.	–7178.8834 a.u.
ZPE (S_0)	0.5991 a.u.	0.5276 a.u.	0.5079 a.u.	0.5540 a.u.
T_1	–7220.1678 a.u.	–6913.876 a.u.	–7025.0579 a.u.	–7178.7858
ZPE (T_1)	0.5927 a.u.	0.5235 a.u.	0.5030 a.u.	0.547992
E_{adia}	2.42 eV	2.02 eV	2.40 eV	2.66 eV
E_{0-0}	2.25 eV	1.91 eV	2.26 eV	2.49 eV
E_{exp}	2.37 eV	2.08 eV	2.47 eV	2.72 eV

Table S10. Calculated adiabatic (E_{adia}) and zero-point energy corrected adiabatic energies (E_{0-0}) for **guests** without DBI (guest = coronene (**3**), pyrene (**G11**), **G12** and **G13**) and 4-Br DBI (**2e**) without guest.

Energy[eV]	Coronene	G11 (pyrene)	G12	G13	4-Br DBI
S_0	–922.4345 a.u.	–616.1316 a.u.	–727.3265 a.u.	–881.0627 a.u.	–6297.7938 a.u.
ZPE (S_0)	0.2753 a.u.	0.2038 a.u.	0.1841 a.u.	0.2302 a.u.	0.3228 a.u.
T_1	–922.3445 a.u.	–616.0569 a.u.	–727.2379 a.u.	–880.9644 a.u.	–6297.6745 a.u.
ZPE (T_1)	0.2690 a.u.	0.1997 a.u.	0.1792 a.u.	0.2243 a.u.	0.3164
E_{adia}	2.45 eV	2.03 eV	2.41 eV	2.68 eV	3.25 eV
E_{0-0}	2.28 eV	1.92	2.28 eV	2.52 eV	3.07 eV

8 Stability tests

8.1 TGA/DSC measurements

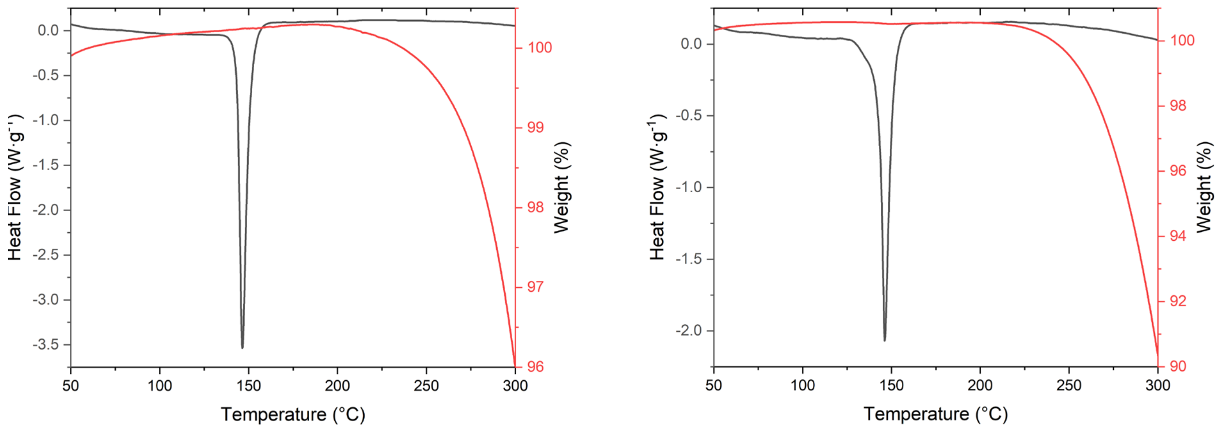

Figure S188. TGA/DSC curves of 4-Cl DBI (**2b**, left) and 4-Br DBI (**2e**, right).

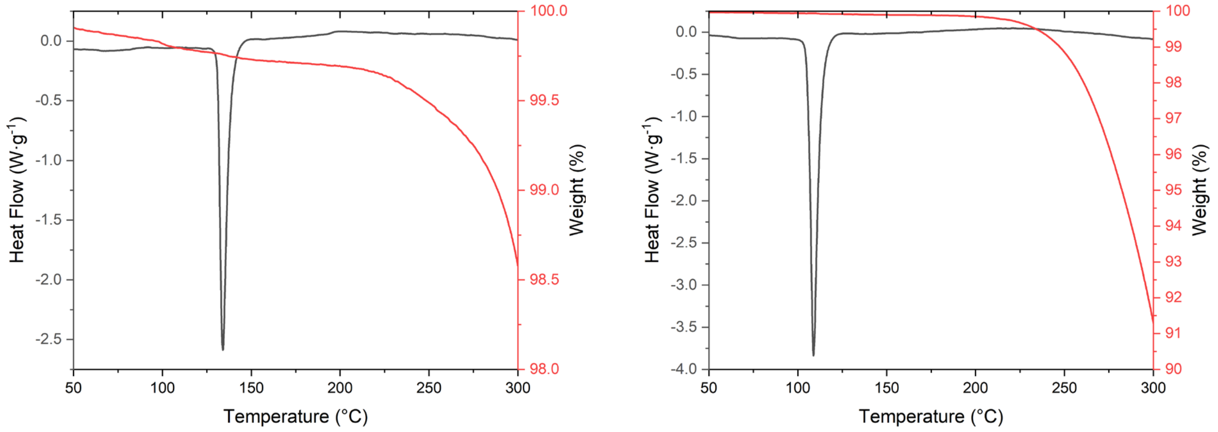

Figure S189. TGA/DSC curves of 4-I DBI (**2g**, left) and 4-OMe DBI (**2h**, right).

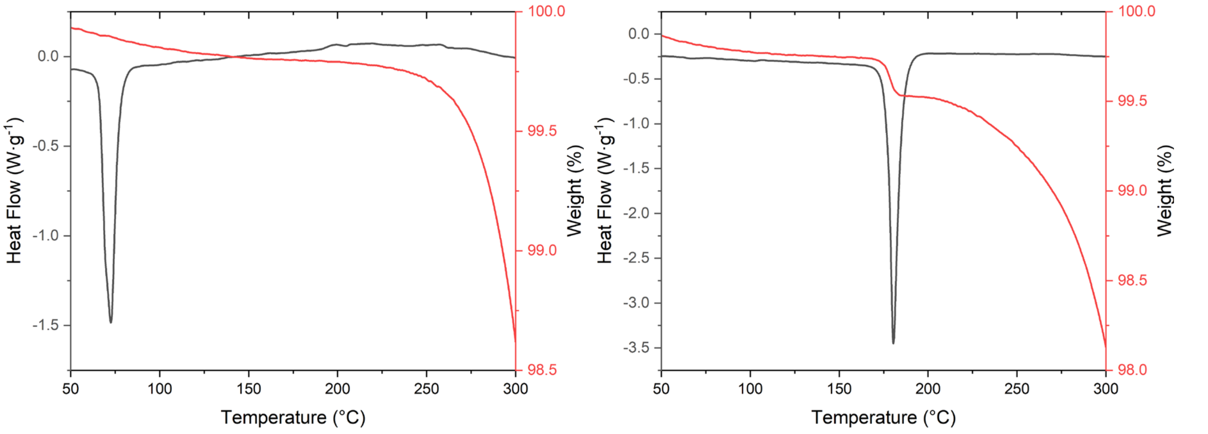

Figure S190. TGA/DSC curves of 4-SPh DBI (**2n**, left) and 4-CN DBI (**2r**, right).

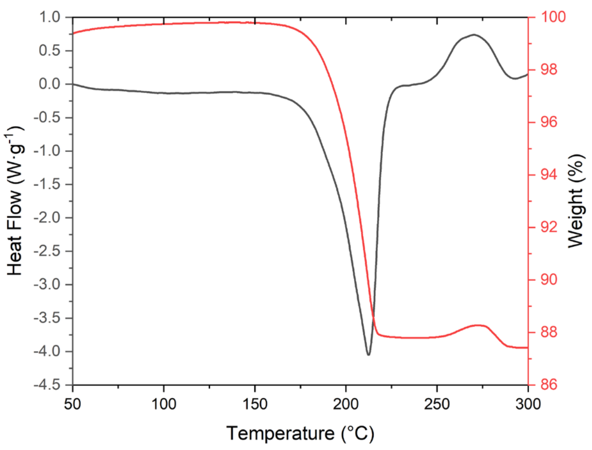

Figure S191. TGA/DSC curves of 4-B(OH)$_2$ DBI (**2u**).

8.2 Photostability tests

8.2.1 UV stability of the 4-Br DBI (2e)

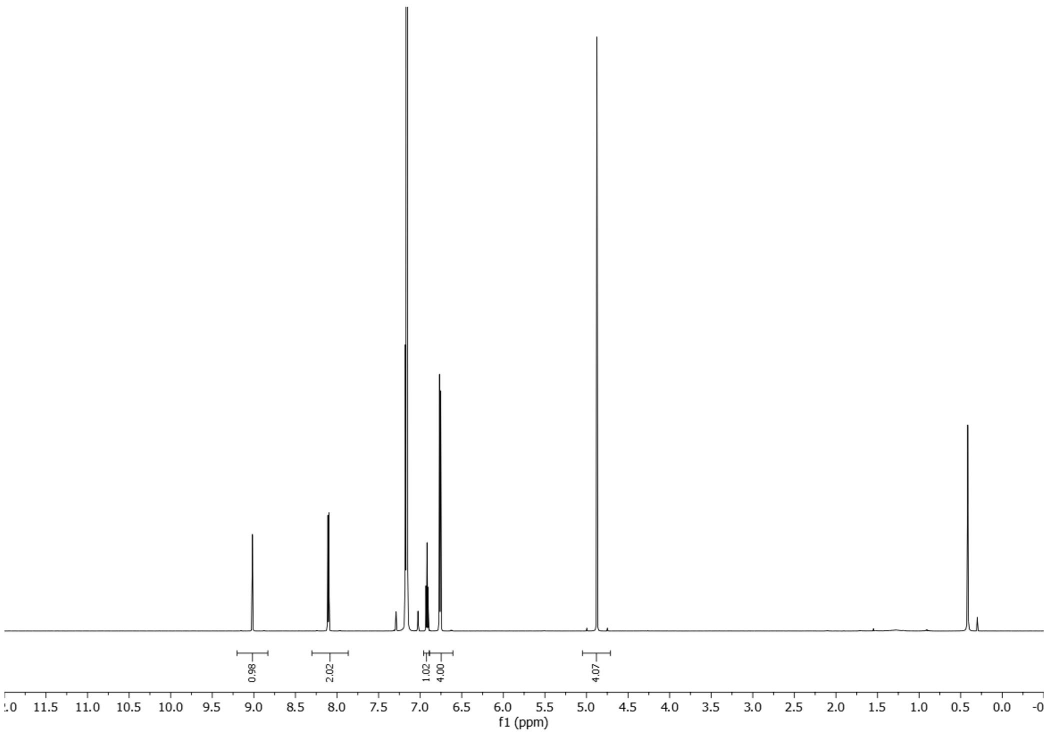

Figure S192. ^{1}H-NMR spectrum of 4-Br DBI (**2e**) in C_6D_6 (c = 5 mg/mL) before UV-irradiation. An integral of 5 H is missing due to an overlap with the benzene signal. (See 6.1 for a ^{1}H-NMR spectrum in $CDCl_3$ with all signals resolved. C_6D_6 was chosen as a solvent due to its light stability).

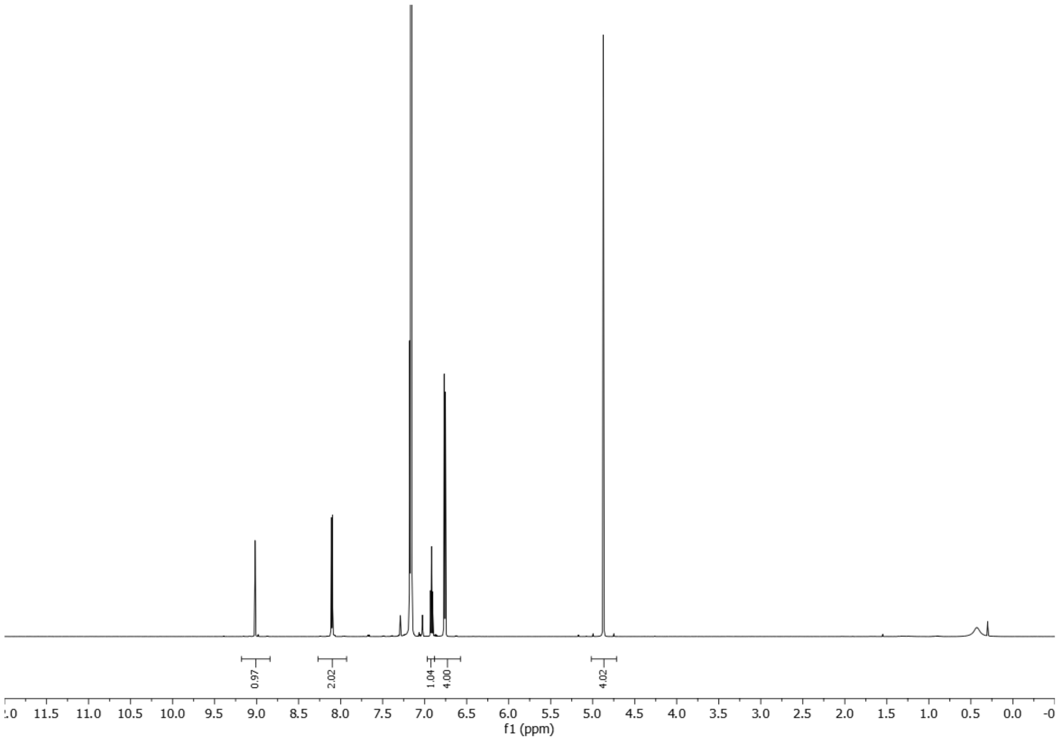

Figure S193. ^{1}H-NMR spectrum of 4-Br DBI (**2e**) in C_6D_6 after 7.5 h of continuous UV-irradiation at 345 nm in the spectrometer (the excitation bandwidth was set to 20 nm for this experiment). The sample was irradiated in a 10x10 mm quartz cuvette as a solution in C_6D_6 (2.0 mL, c = 5 mg/mL). An integral of 5 H is missing due to an overlap with the benzene signal.

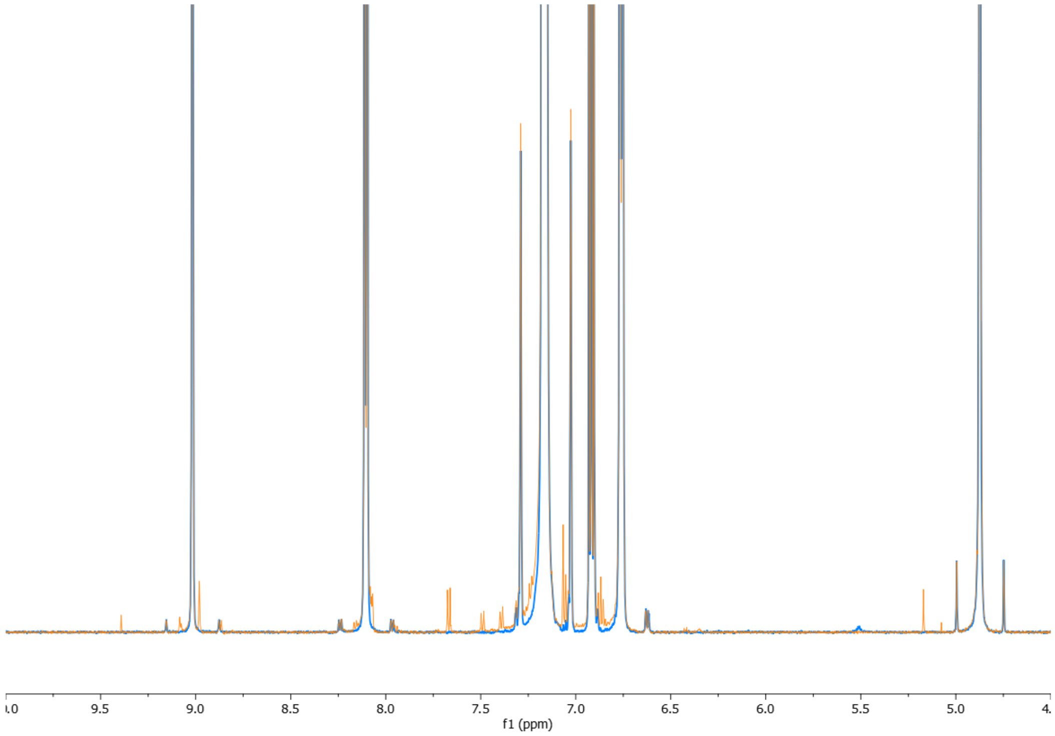

Figure S194. Magnified comparison of ^{1}H-NMR spectra of 4-Br DBI (**2e**) in C_6D_6 before (blue) and after (orange) UV-irradiation showing only minor degradation.

8.2.2 Photostability of selected host/guest-systems

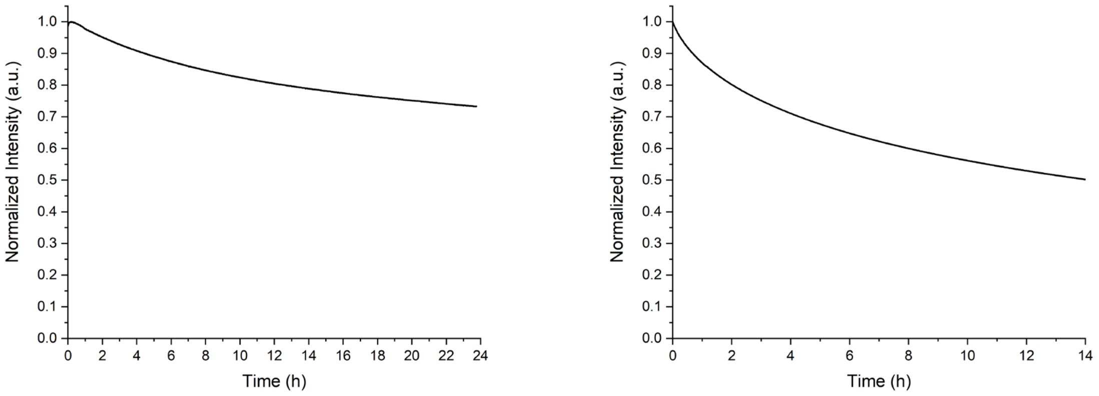

Figure S195. Normalized evolution of phosphorescence intensity at 571 nm over time during continuous irradiation. (Left) Coronene (**3**) in 4-Cl DBI (**2b**) (0.1 wt%). (Right) Coronene (**3**) in 4-I DBI (**2g**) (0.1 wt%). Excitation at 345 nm for both.

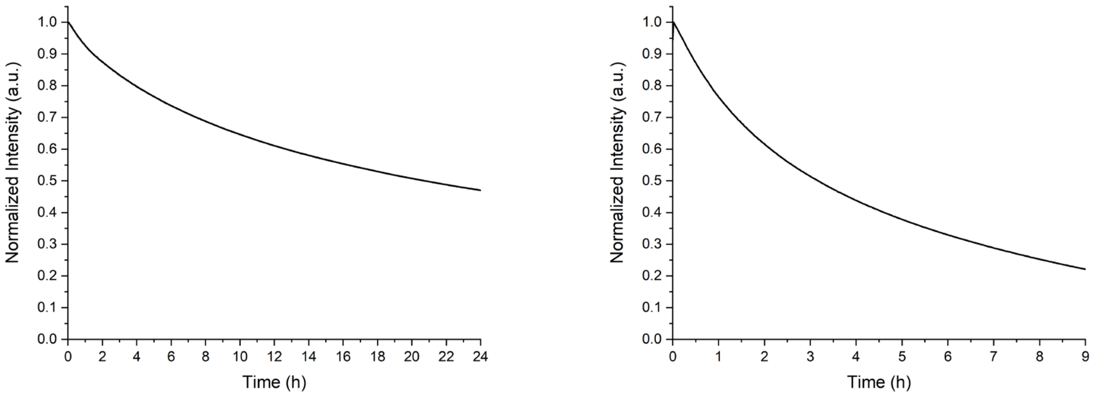

Figure S196. (Left) Normalized evolution of phosphorescence intensity at 571 nm over time during continuous irradiation. Coronene (**3**) in 4-Br DBI (**2e**) (0.1 wt%). (Right) Normalized evolution of phosphorescence intensity at 595 nm over time during continuous irradiation. Pyrene-d_{10} in 4-Br DBI (**2e**) (0.1 wt%). Excitation at 345 nm for both.

9 Spectra used for quantum yield determinations

9.1 Color scheme

The following colors were used for the integrating sphere spectra: Black – Incident light spectra (no sample). Red – Indirect light spectra of the samples. Blue – Direct light spectra of the samples. Light blue – Direct light spectra of the samples with subtracted phosphorescence spectra (fluorescence only).

In some cases, the signal-to-noise ratio of the measured direct light spectra was insufficient for determining very low RTP quantum yields. In these cases, parts of the spectra were replaced by scaled steady-state spectra measured in the PPH-150 sample holder, which had a much higher quality. In these cases, the original direct light spectra of the samples were colored green, while the replaced spectra were colored blue. The fluorescence spectra (light blue) were then obtained from the scaled steady-state replacements.

9.2 Quantum yield spectra for DBIs + coronene (0.1 wt%)

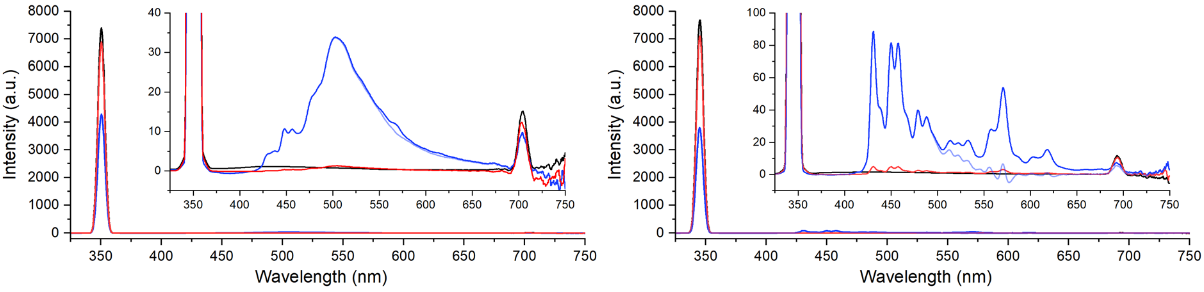

Figure S197. Integrating sphere spectra of 0.1 wt% of coronene in 4-H DBI (**2a**, left) and 4-Cl DBI (**2b**, right). λ_{ex} = 350 nm (left), 345 nm (right). For coloring details, see 9.1.

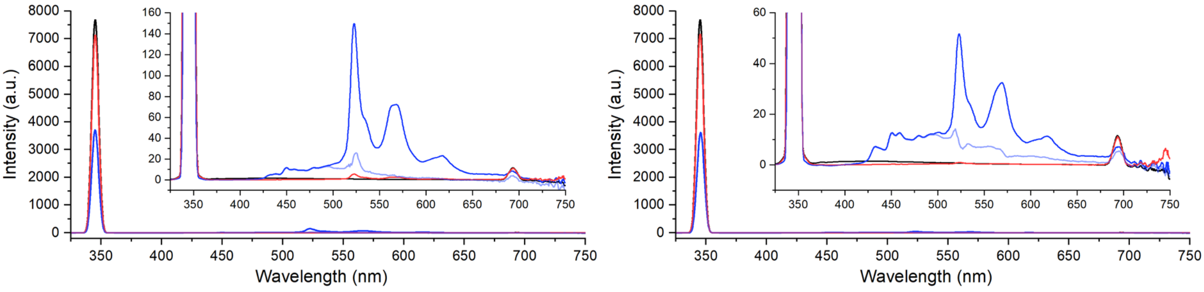

Figure S198. Integrating sphere spectra of 0.1 wt% of coronene in 2-Br DBI (**2c**, left) and 3-Br DBI (**2d**, right). λ_{ex} = 345 nm. For coloring details, see 9.1.

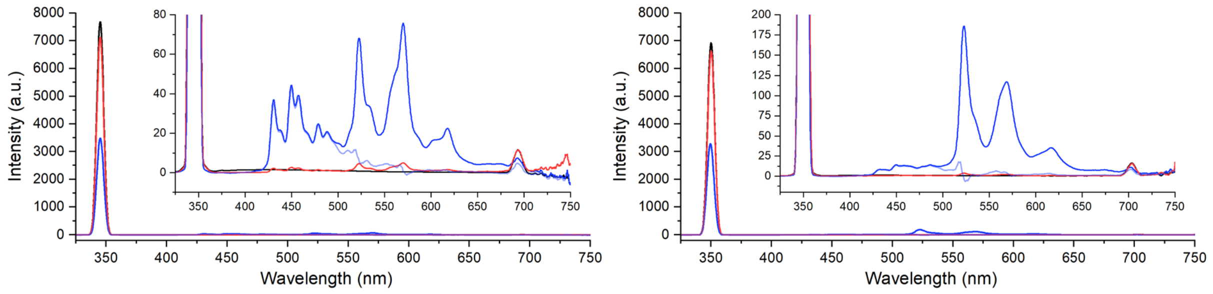

Figure S199. Integrating sphere spectra of 0.1 wt% of coronene in 4-Br DBI (**2e**, left) and 4-Br-2-OMe DBI (**2f**, right). λ_{ex} = 345 nm (left), 350 nm (right). For coloring details, see 9.1.

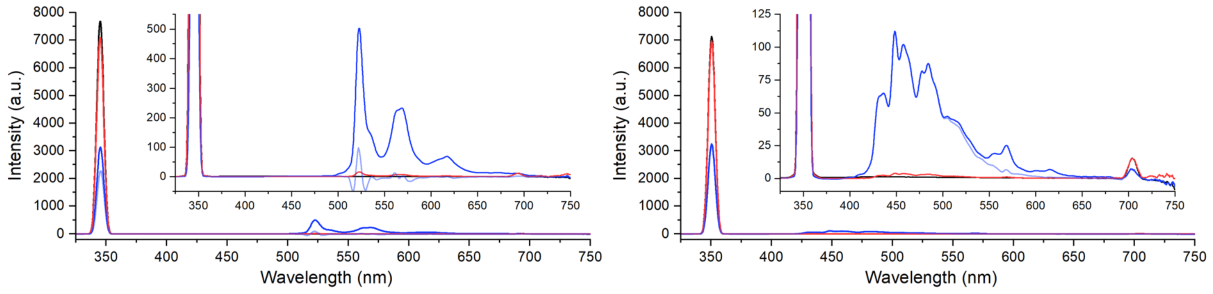

Figure S200. Integrating sphere spectra of 0.1 wt% of coronene in 4-I DBI (**2g**, left) and 4-OMe DBI (**2h**, right). λ_{ex} = 345 nm (left), 350 nm (right). For coloring details, see 9.1.

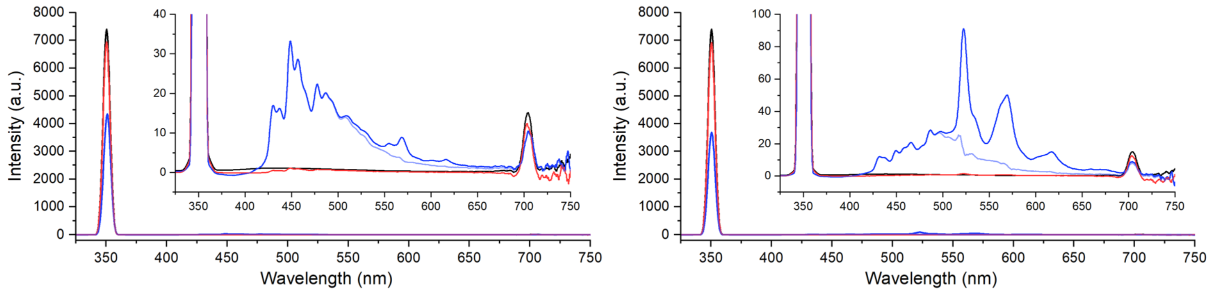

Figure S201. Integrating sphere spectra of 0.1 wt% of coronene in 4-(OPh(4-OMe)) DBI (**2i**, left) and 4-(OPh(4-Br)) DBI (**2j**, right). λ_{ex} = 350 nm. For coloring details, see 9.1.

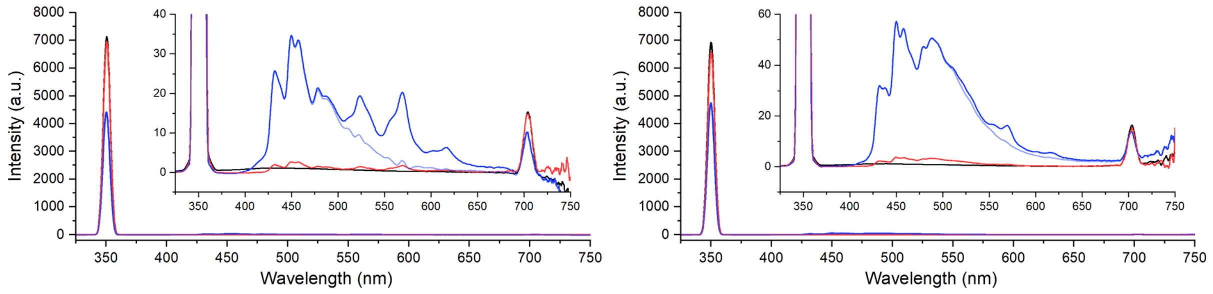

Figure S202. Integrating sphere spectra of 0.1 wt% of coronene in 4-SMe DBI (**2k**, left) and 4-S(O)Me DBI (**2l**, right). λ_{ex} = 350 nm. For coloring details, see 9.1.

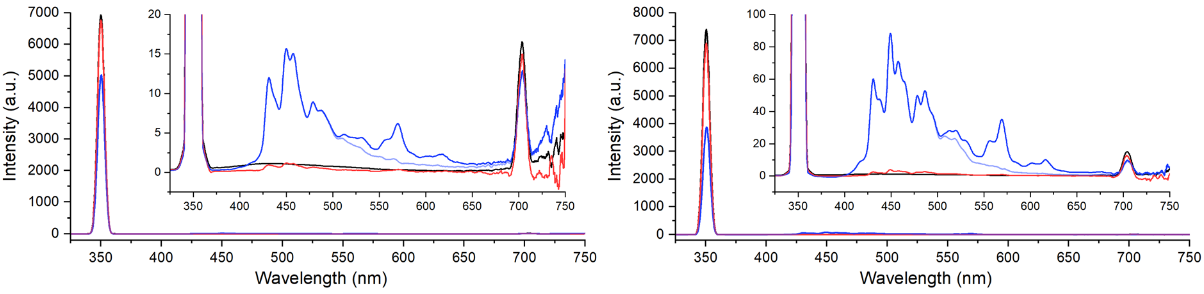

Figure S203. Integrating sphere spectra of 0.1 wt% of coronene in 4-SO_2Me DBI (**2m**, left) and 4-SPh DBI (**2n**, right). λ_{ex} = 350 nm. For coloring details, see 9.1.

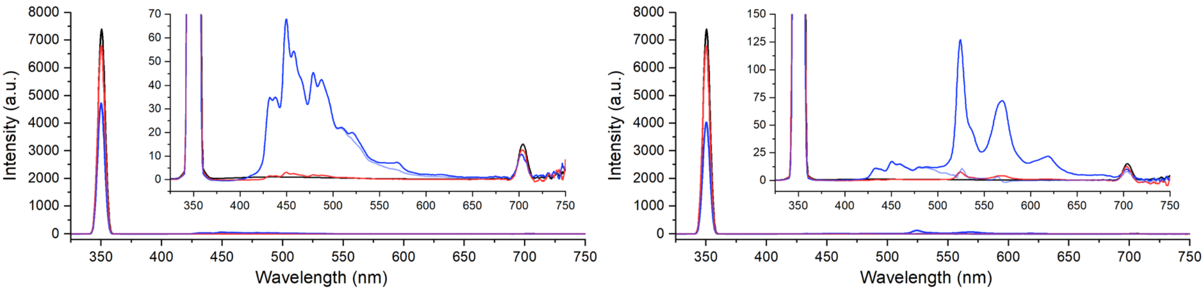

Figure S204. Integrating sphere spectra of 0.1 wt% of coronene in 4-(SPh(4-SMe)) DBI (**2o**, left) and 4-(SPh(4-Br)) DBI (**2p**, right). λ_{ex} = 350 nm. For coloring details, see 9.1.

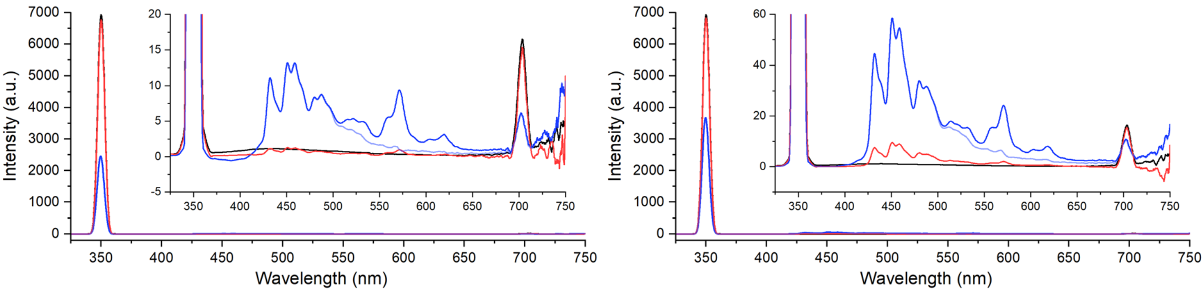

Figure S205. Integrating sphere spectra of 0.1 wt% of coronene in 4-CHO DBI (**2q**, left) and 4-CN DBI (**2r**, right). λ_{ex} = 350 nm. For coloring details, see 9.1.

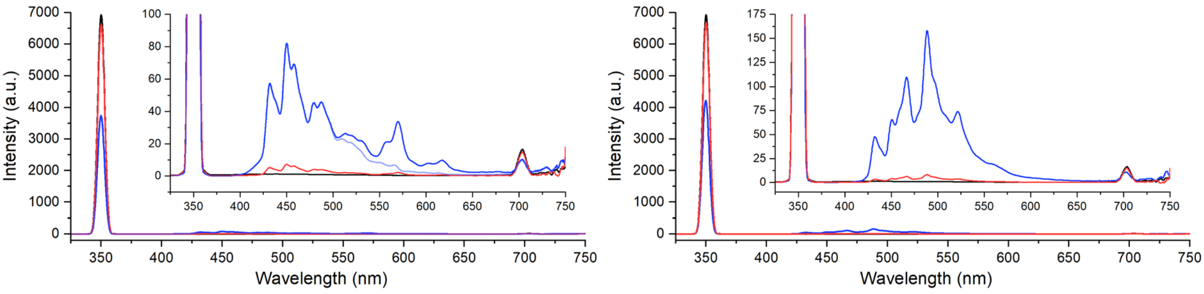

Figure S206. Integrating sphere spectra of 0.1 wt% of coronene in 4-CO_2Me DBI (**2s**, left) and 4-CF3 DBI (**2t**, right). λ_{ex} = 350 nm. For coloring details, see 9.1.

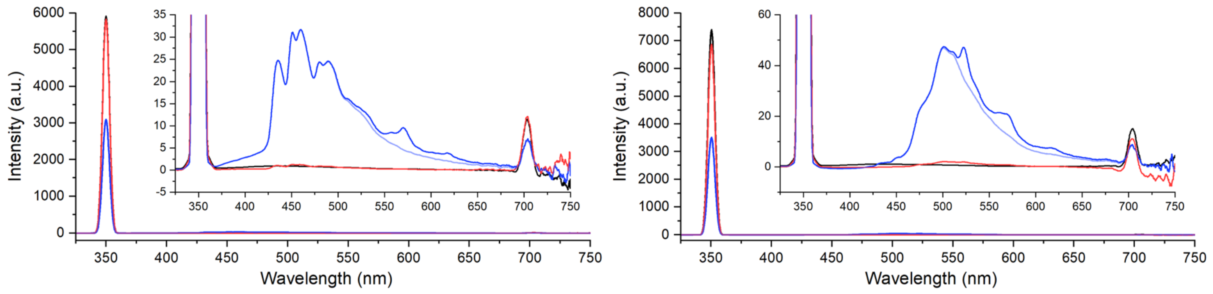

Figure S207. Integrating sphere spectra of 0.1 wt% of coronene in 4-B(OH)$_2$ DBI (**2u**, left) and **S1** (right). λ_{ex} = 350 nm. For coloring details, see 9.1.

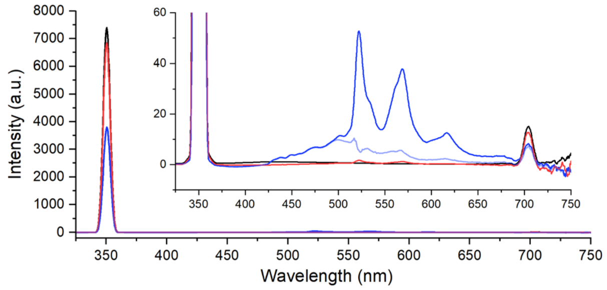

Figure S208. Integrating sphere spectra of 0.1 wt% of coronene in **S2**. λ_{ex} = 350 nm. For coloring details, see 9.1.

9.3 Quantum yield spectra for DBIs + coronene-d_{12} (0.1 wt%)

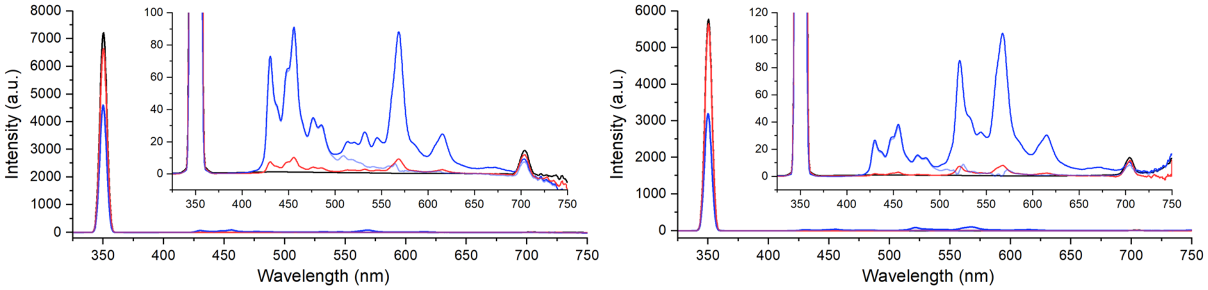

Figure S209. Integrating sphere spectra of 0.1 wt% of coronene-d_{12} in 4-Cl DBI (**2b**, left) and 4-Br DBI (**2e**, right). λ_{ex} = 350 nm. For coloring details, see 9.1.

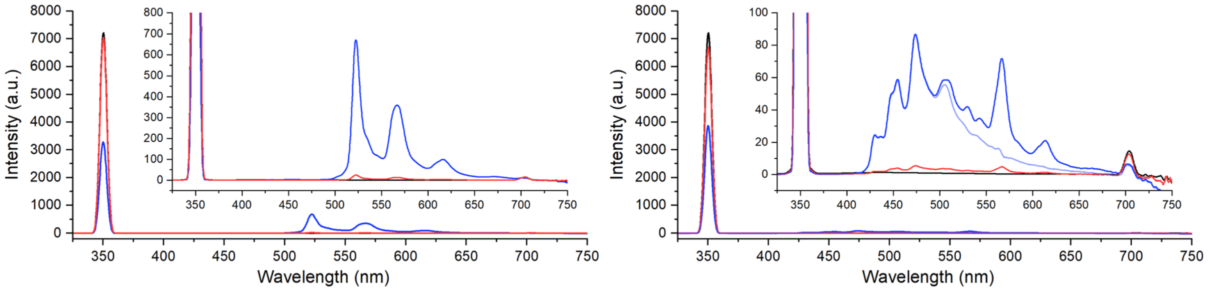

Figure S210. Integrating sphere spectra of 0.1 wt% of coronene-d_{12} in 4-I DBI (**2g**, left) and 4-OMe DBI (**2h**, right). λ_{ex} = 350 nm. For coloring details, see 9.1.

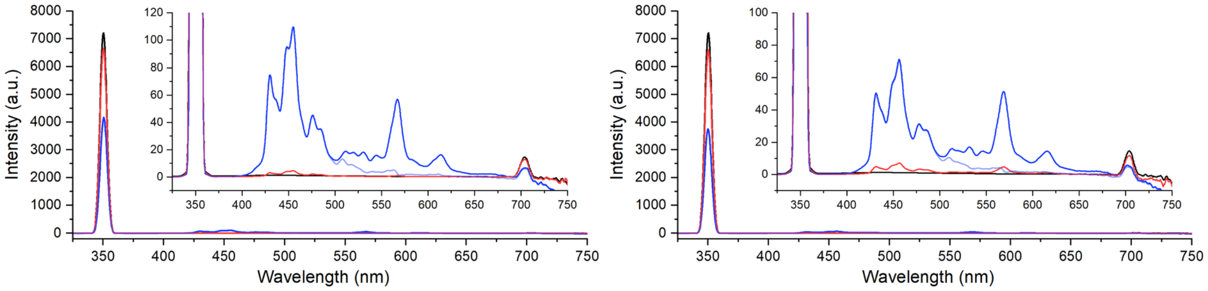

Figure S211. Integrating sphere spectra of 0.1 wt% of coronene-d_{12} in 4-SPh DBI (**2n**, left) and 4-CN DBI (**2r**, right). λ_{ex} = 350 nm. For coloring details, see 9.1.

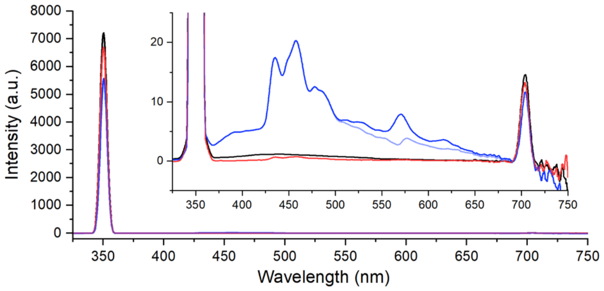

Figure S212. Integrating sphere spectra of 0.1 wt% of coronene-d_{12} in 4-$B(OH)_2$ DBI (**2u**). λ_{ex} = 350 nm. For coloring details, see 9.1.

9.4 Quantum yield spectra for 4-Br DBI (2e) + different guests (0.1 wt%)

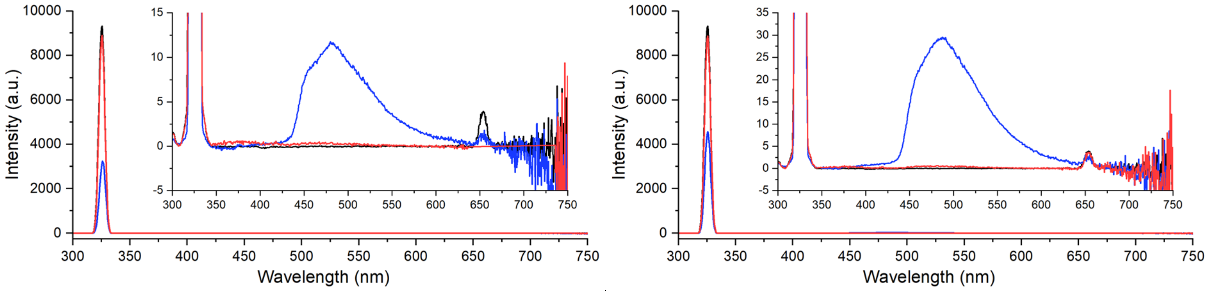

Figure S213. Integrating sphere spectra of 4-Br DBI host with **G1** (left) and **G2** (right) (0.1 wt%). λ_{ex} = 325 nm. For coloring details, see 9.1.

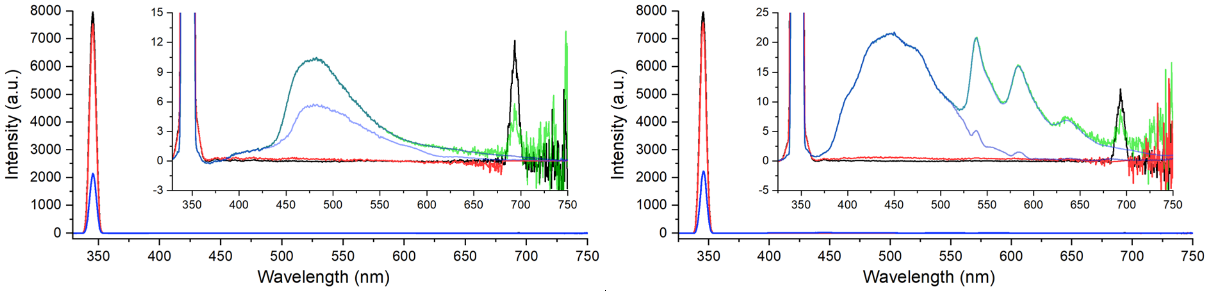

Figure S214. Integrating sphere spectra of 4-Br DBI host with **G13** (left) and **G4** (right) (0.1 wt%). λ_{ex} = 345 nm. For coloring details, see 9.1.

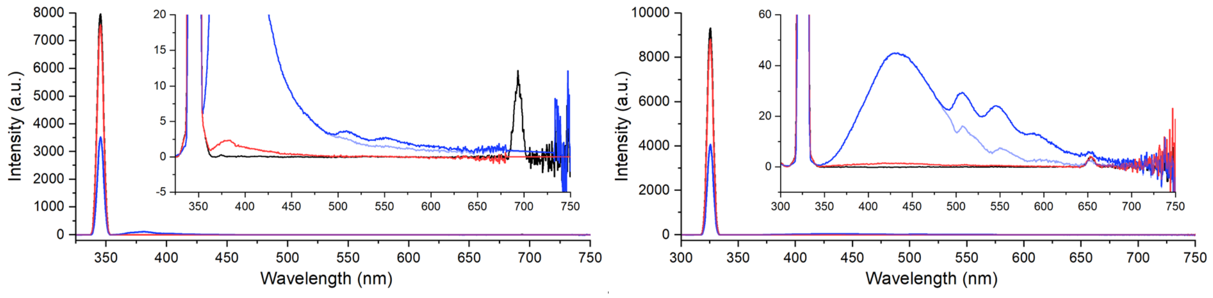

Figure S215. Integrating sphere spectra of 4-Br DBI host with **G5** (left) and **G6** (right) (0.1 wt%). λ_{ex} = 345 nm (left), 325 nm (right). For coloring details, see 9.1.

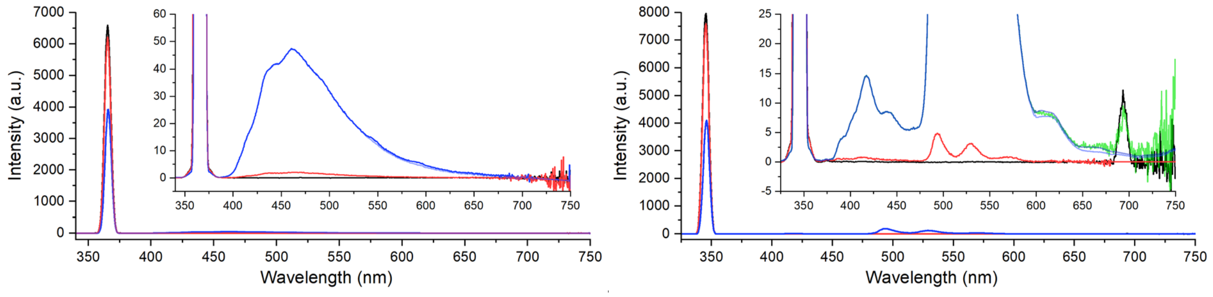

Figure S216. Integrating sphere spectra of 4-Br DBI host with **G7** (left) and **G8** (right) (0.1 wt%). λ_{ex} = 365 nm (left), 345 nm (right). For coloring details, see 9.1.

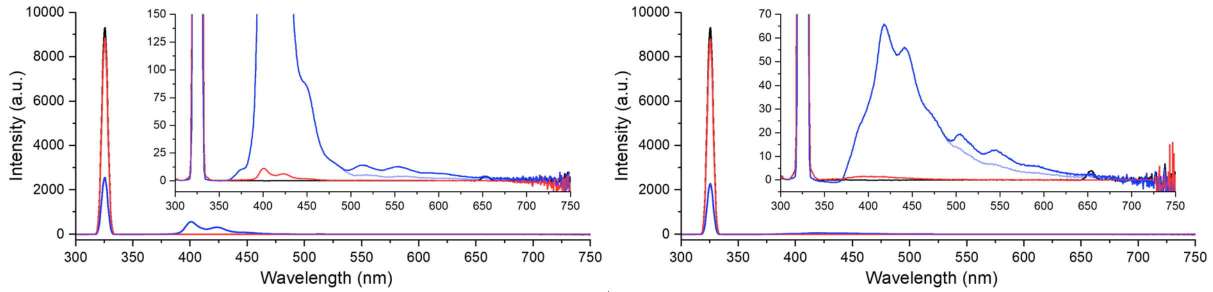

Figure S217. Integrating sphere spectra of 4-Br DBI host with **G9** (left) and **G10** (right) (0.1 wt%). λ_{ex} = 325 nm. For coloring details, see 9.1.

–

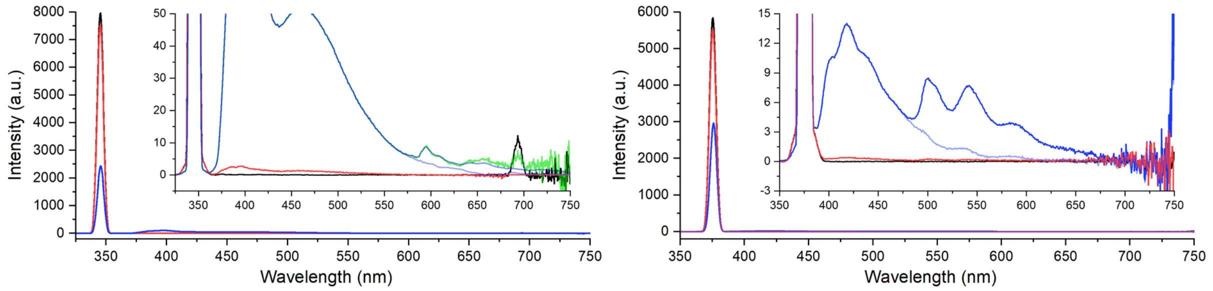

Figure S218. Integrating sphere spectra of 4-Br DBI host with **G11** (left) and **G12** (right) (0.1 wt%). λ_{ex} = 345 nm (left), 375 nm (right). For coloring details, see 9.1.

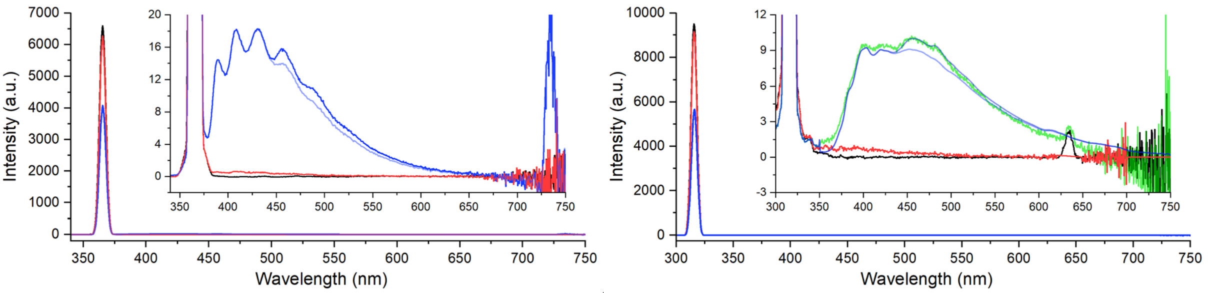

Figure S219. Integrating sphere spectra of 4-Br DBI host with **G13** (left) and **G14** (right) (0.1 wt%). λ_{ex} = 365 nm (left), 315 nm (right). For coloring details, see 9.1.

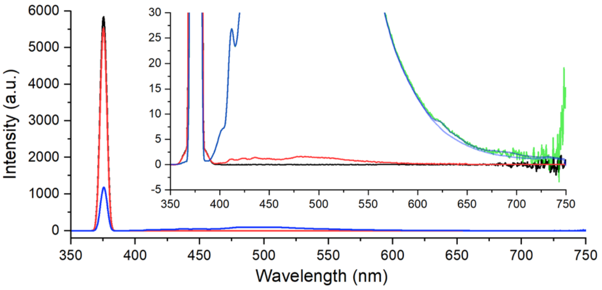

Figure S220. Integrating sphere spectra of 4-Br DBI host with **G15** (0.1 wt%). λ_{ex} = 375 nm. For coloring details, see 9.1.

9.5 Other quantum yield spectra

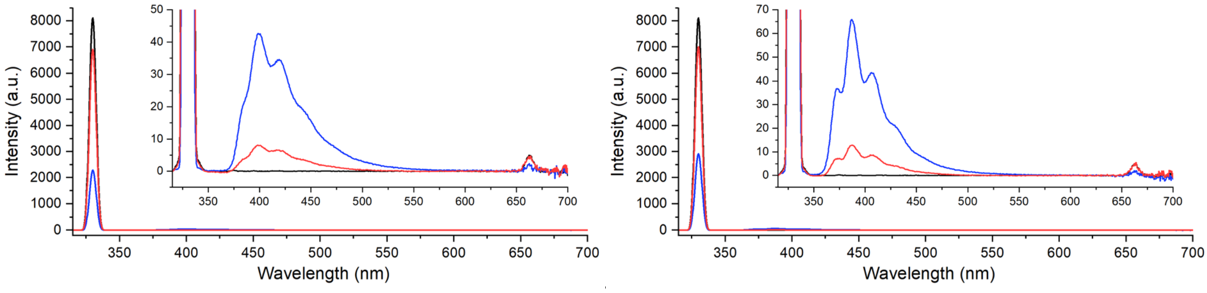

Figure S221. Integrating sphere spectra of **G12** (left) and **G13** (right) in DCM (c = 3.0 μg/mL). Measured in 10x10 mm quartz cuvettes. λ_{ex} = 330 nm. For coloring details, see 9.1.

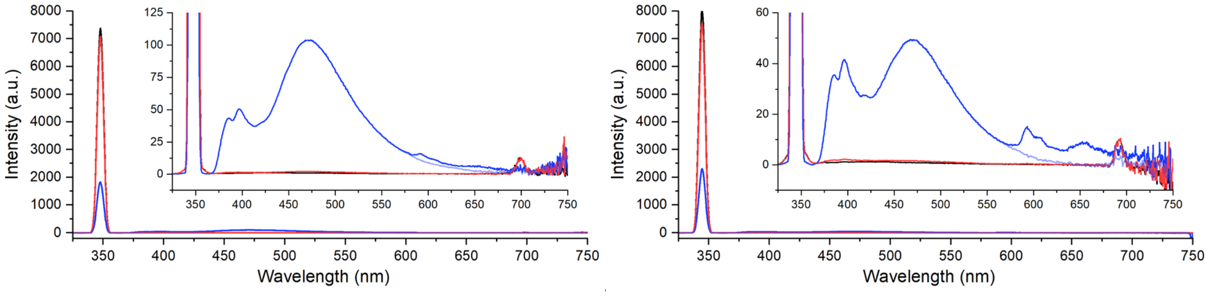

Figure S222. Integrating sphere spectra of 4-Cl DBI (**2b**, left) and 4-Br DBI (**2e**, right) with **pyrene-*d*$_{10}$** (0.1 wt%). λ_{ex} = 345 nm. For coloring details, see 9.1.

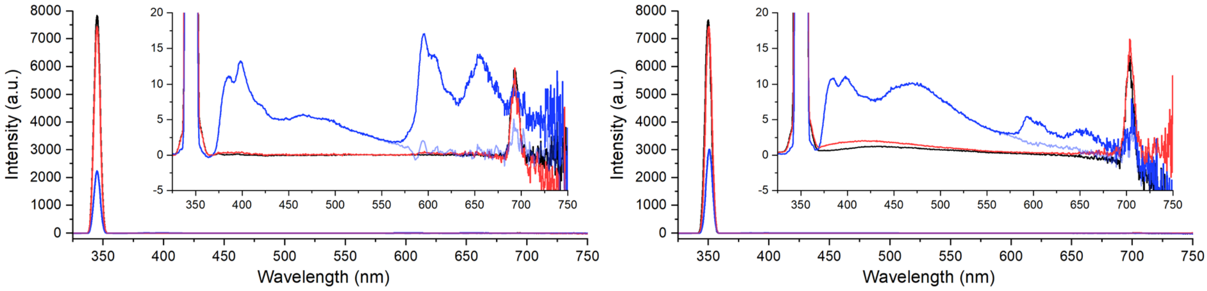

Figure S223. Integrating sphere spectra of 4-I DBI (**2g**) with **pyrene-*d*$_{10}$** (0.1 wt%) (left) and a mixture of *p*-toluic acid (98.9 wt%) and 4-Br DBI (**2e**, 1 wt%) with **pyrene-*d*$_{10}$** (0.1 wt%) (right). λ_{ex} = 345 nm (left), 350 nm (right). For coloring details, see 9.1.

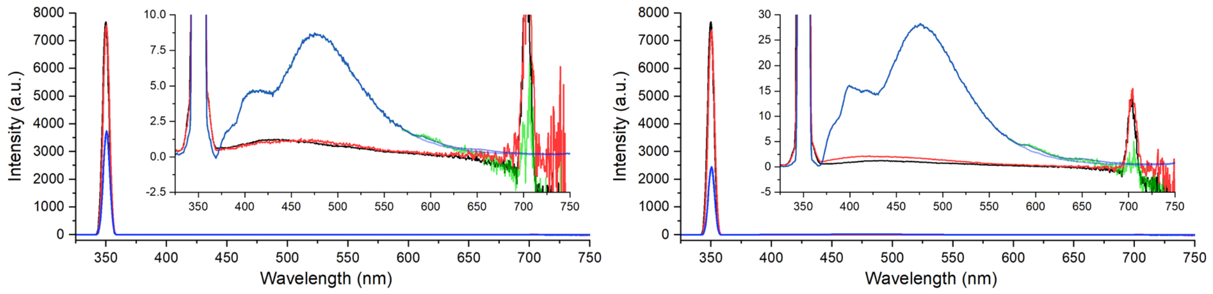

Figure S224. Integrating sphere spectra of 4-H DBI (**2a**) with **pyrene-*d*$_{10}$** (0.1 wt%) (left) and a mixture of 4-H DBI (**2a**, 98.9 wt%) and 4-Br DBI (**2e**, 1 wt%) with **pyrene-*d*$_{10}$** (0.1 wt%) (right). λ_{ex} = 350 nm. For coloring details, see 9.1.

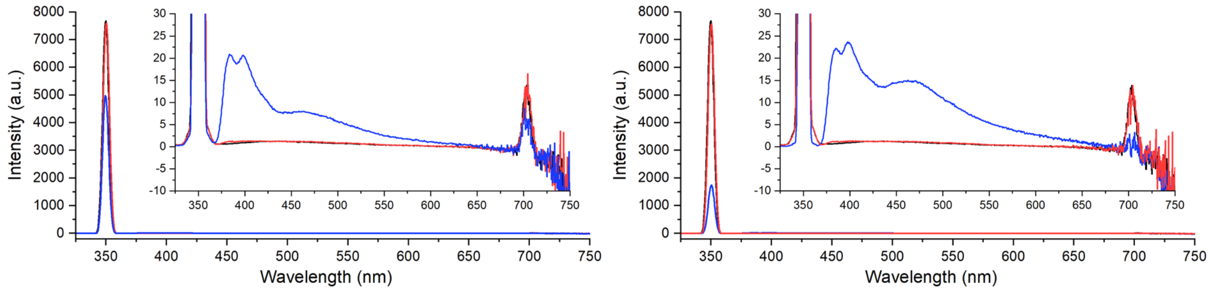

Figure S225. Integrating sphere spectra of 4-CF_3 DBI (**2t**) with **pyrene-*d*$_{10}$** (0.1 wt%) (left) and a mixture of 4-CF_3 DBI (**2t**, 98.9 wt%) and 4-Br DBI (**2e**, 1 wt%) with **pyrene-*d*$_{10}$** (0.1 wt%) (right). λ_{ex} = 350 nm. For coloring details, see 9.1.

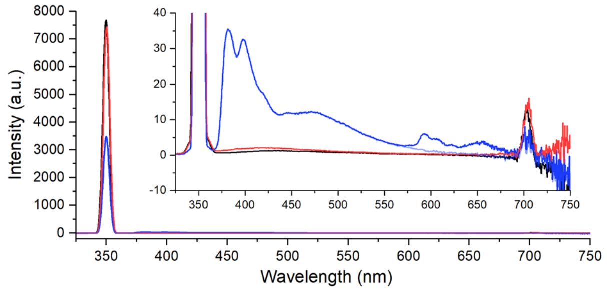

Figure S226. Integrating sphere spectra of a mixture of 4-Br DBI (**2e**, 98.9 wt%) and 4-CF_3 DBI (**2t**, 1 wt%) with **pyrene-*d*$_{10}$** (0.1 wt%) (right). λ_{ex} = 350 nm. For coloring details, see 9.1.

10 References

1 L. Gu, H. Shi, M. Gu, K. Ling, H. Ma, S. Cai, L. Song, C. Ma, H. Li, G. Xing, X. Hang, J. Li, Y. Gao, W. Yao, Z. Shuai, Z. An, X. Liu and W. Huang, Dynamic Ultralong Organic Phosphorescence by Photoactivation, *Angew. Chem. Int. Ed.*, 2018, **57**, 8425–8431.

2 H. Mieno, R. Kabe, N. Notsuka, M. D. Allendorf and C. Adachi, Long-Lived Room-Temperature Phosphorescence of Coronene in Zeolitic Imidazolate Framework ZIF-8, *Adv. Opt. Mater.*, 2016, **4**, 1015–1021.

3 J. L. Kropp and W. R. Dawson, Radiationless deactivation of triplet coronene in plastics, *J. Phys. Chem.*, 1967, **71**, 4499–4506.

4 I. Bhattacharjee, K. Hayashi and S. Hirata, Key of Suppressed Triplet Nonradiative Transition-Dependent Chemical Backbone for Spatial Self-Tunable Afterglow, *JACS Au*, 2021, **1**, 945–954.

5 Y. Liang, P. Hu, H. Zhang, Q. Yang, H. Wei, R. Chen, J. Yu, C. Liu, Y. Wang, S. Luo, G. Shi, Z. Chi and B. Xu, Enabling Highly Robust Full-Color Ultralong Room-Temperature Phosphorescence and Stable White Organic Afterglow from Polycyclic Aromatic Hydrocarbons, *Angew. Chem. Int. Ed.*, 2024, **63**, e202318516.

6 M. Wu, X. Wang, Y. Pan, J. Li, X. Li, Y. Sun, Y. Zou, H. Zhang and K. Zhang, Two-Component Design Strategy: Achieving Intense Organic Afterglow and Diverse Functions in Coronene-Matrix Systems, *J. Phys. Chem. C*, 2021, **125**, 26986–26998.

7 S. Hirata and M. Vacha, Large Reverse Saturable Absorption at the Sunlight Power Level Using the Ultralong Lifetime of Triplet Excitons, *J. Phys. Chem. Lett.*, 2017, **8**, 3683–3689.

8 B. Ding, L. Ma, Z. Huang, X. Ma and H. Tian, Engendering persistent organic room temperature phosphorescence by trace ingredient incorporation, *Sci. Adv.*, 2021, **7**, eabf9668.

9 X. Zhang, L. Du, W. Zhao, Z. Zhao, Y. Xiong, X. He, P. F. Gao, P. Alam, C. Wang, Z. Li, J. Leng, J. Liu, C. Zhou, J. W. Y. Lam, D. L. Phillips, G. Zhang and B. Z.

Tang, Ultralong UV/mechano-excited room temperature phosphorescence from purely organic cluster excitons, *Nat. Commun.*, 2019, **10**, 5161.

10 Y. Wang, W. Ye, T. Cao, C. Wang, H. Meng, Z. Gao and C. Wang, Metal-Free organic polymeric room temperature phosphorescence system with Multi-Colour and ultralong lifetime, *Chem. Eng. J.*, 2024, **481**, 148642.

11 H. Wu, L. Gu, G. V. Baryshnikov, H. Wang, B. F. Minaev, H. Ågren and Y. Zhao, Molecular Phosphorescence in Polymer Matrix with Reversible Sensitivity, *ACS Appl. Mater. Interfaces*, 2020, **12**, 20765–20774.

12 R. Gahlaut, H. C. Joshi, N. K. Joshi, N. Pandey, P. Arora, R. Rautela, K. Suyal and S. Pant, Luminescence characteristics and room temperature phosphorescence of naphthoic acids in polymers, *J. Lumin.*, 2013, **138**, 122–128.

13 R. D. Burkhart, Kinetics of the delayed luminescence decay of 1,2-benzanthracene in polystyrene films, *Chem. Phys.*, 1980, **46**, 11–21.

14 S. Hirata, Intrinsic Analysis of Radiative and Room-Temperature Nonradiative Processes Based on Triplet State Intramolecular Vibrations of Heavy Atom-Free Conjugated Molecules toward Efficient Persistent Room-Temperature Phosphorescence, *J. Phys. Chem. Lett.*, 2018, **9**, 4251–4259.

15 L. Ma, Y. Liu, T. Jiang, L. Zhou, Q. Wang, H. Tian and X. Ma, Ion-Radical Mediated Multi-Color Ultra-Long Afterglow Materials, *ChemRxiv [preprint]*, 2024, DOI:10.26434/chemrxiv-2024-35nc4.

16 W. Qiu, X. Cai, M. Li, Z. Chen, L. Wang, W. Xie, K. Liu, M. Liu and S.-J. Su, Achieving Purely Organic Room-Temperature Phosphorescence Mediated by a Host-Guest Charge Transfer State, *J. Phys. Chem. Lett.*, 2021, **12**, 4600–4608.

17 Y. Xia, C. Zhu, F. Cao, Y. Shen, M. Ouyang and Y. Zhang, Host-Guest Doping in Flexible Organic Crystals for Room-Temperature Phosphorescence, *Angew. Chem. Int. Ed.*, 2022, **62**, e202217547.

18 H. Wu, D. Wang, Z. Zhao, D. Wang, Y. Xiong and B. Z. Tang, Tailoring Noncovalent Interactions to Activate Persistent Room-Temperature Phosphorescence from Doped Polyacrylonitrile Films, *Adv. Funct. Mater.*, 2021, **31**, 2101656.

19 A. Tanaka, T. Terasawa, H. Hagihara, Y. Sakuma, N. Ishibe, M. Sawada, H. Takasugi and H. Tanaka, Inhibitors of acyl-CoA: cholesterol O-acyltransferase (ACAT). Part 1: Identification and structure-activity relationships of a novel series of substituted N-alkyl-N-biphenylylmethyl-N′-arylureas, *Bioorg. Med. Chem.*, 1998, **6**, 15–30.

20 K. S. Ravikumar, J.-P. Bégué and D. Bonnet-Delpon, A selective conversion of sulfide to sulfoxide in hexafluoro-2-propanol, *Tetrahedron Lett.*, 1998, **39**, 3141–3144.

21 J. McNulty and D. McLeod, An iterative approach toward the synthesis of discrete oligomeric p-phenylene vinylene organic dyes employing aqueous Wittig chemistry, *Tetrahedron Lett.*, 2011, **52**, 5467–5470.

22 C. Mahecha-Mahecha, F. Lecornué, S. Akinari, T. Charote, D. Gamba-Sánchez, T. Ohwada and S. Thibaudeau, Sequential Suzuki-Miyaura Coupling/Lewis Acid-Catalyzed Cyclization: An Entry to Functionalized Cycloalkane-Fused Naphthalenes, *Org. Lett.*, 2020, **22**, 6267–6271.

23 G. Manolikakes and P. Knochel, Radical catalysis of Kumada cross-coupling reactions using functionalized Grignard reagents, *Angew. Chem. Int. Ed.*, 2009, **48**, 205–209.

24 T. Fujita, N. Shoji, N. Yoshikawa and J. Ichikawa, Helicene synthesis by Brønsted acid-catalyzed cycloaromatization in HFIP (CF3)2CHOH, *Beilstein J. Org. Chem.*, 2021, **17**, 396–403.

25 A. Ham, H. S. Overkleeft, D. V. Filippov and G. F. Schneider, A Three-Step Synthesis of 4 H -Cyclopenta[def]phenanthrene from Pyrene, *Eur. J. Org. Chem.*, 2021, **2021**, 2013–2017.

26 J. Yan, B. R. Travis and B. Borhan, Direct oxidative cleavage of alpha- and beta-dicarbonyls and alpha-hydroxyketones to diesters with KHSO5, *J. Org. Chem.*, 2004, **69**, 9299–9302.

27 R. G. Gillis and Q. N. Porter, 5-Methoxyphenanthrene-4-carboxylic Acid, *Aust. J. Chem.*, 1989, **42**, 1007.

28 N. Saha, H. Wang, S. Zhang, Y. Du, D. Zhu, Y. Hu, P. Huang and S. Wen, Domino Carbopalladation/C-H Activation as a Quick Access to Polycyclic Frameworks, *Org. Lett.*, 2018, **20**, 712–715.

29 F. W. Wassmundt and W. F. Kiesman, Soluble Catalysts for Improved Pschorr Cyclizations, *J. Org. Chem.*, 1995, **60**, 196–201.

30 J. Gallardo-Donaire and R. Martin, Cu-catalyzed mild C(sp2)-H functionalization assisted by carboxylic acids en route to hydroxylated arenes, *J. Am. Chem. Soc.*, 2013, **135**, 9350–9353.

31 J. T. M. van Dijk, A. Hartwijk, A. C. Bleeker, J. Lugtenburg and J. Cornelisse, Gram Scale Synthesis of Benzo[ghi]perylene and Coronene, *J. Org. Chem.*, 1996, **61**, 1136–1139.

32 J. Tao, J. P. Perdew, V. N. Staroverov and G. E. Scuseria, Climbing the density functional ladder: nonempirical meta-generalized gradient approximation designed for molecules and solids, *Phys. Rev. Lett.*, 2003, **91**, 146401.

33 A. K. Wilson, D. E. Woon, K. A. Peterson and T. H. Dunning, Gaussian basis sets for use in correlated molecular calculations. IX. The atoms gallium through krypton, *J. Phys. Chem. Phys.*, 1999, **110**, 7667–7676.

34 T. H. Dunning, Gaussian basis sets for use in correlated molecular calculations. I. The atoms boron through neon and hydrogen, *J. Phys. Chem. Phys.*, 1989, **90**, 1007–1023.

35 S. Grimme, S. Ehrlich and L. Goerigk, Effect of the damping function in dispersion corrected density functional theory, *J. Comput. Chem.*, 2011, **32**, 1456–1465.

36 Gaussian 16, Revision C.01, M. J. Frisch, G. W. Trucks, H. B. Schlegel, G. E. Scuseria, M. A. Robb, J. R. Cheeseman, G. Scalmani, V. Barone, G. A. Petersson, H. Nakatsuji, X. Li, M. Caricato, A. V. Marenich, J. Bloino, B. G. Janesko, R. Gomperts, B. Mennucci, H. P. Hratchian, J. V. Ortiz, A. F. Izmaylov, J. L. Sonnenberg, D. Williams-Young, F. Ding, F. Lipparini, F. Egidi, J. Goings, B. Peng, A. Petrone, T. Henderson, D. Ranasinghe, V. G. Zakrzewski, J. Gao, N. Rega, G. Zheng, W. Liang, M. Hada, M. Ehara, K. Toyota, R. Fukuda, J. Hasegawa, M. Ishida, T. Nakajima, Y. Honda, O. Kitao, H. Nakai, T. Vreven, K. Throssell, Montgomery, J. A., Jr., J. E. Peralta, F. Ogliaro, M. J. Bearpark, J. J. Heyd, E. N. Brothers, K. N. Kudin, V. N. Staroverov, T. A. Keith, R. Kobayashi, J.

Normand, K. Raghavachari, A. P. Rendell, J. C. Burant, S. S. Iyengar, J. Tomasi, M. Cossi, J. M. Millam, M. Klene, C. Adamo, R. Cammi, J. W. Ochterski, R. L. Martin, K. Morokuma, O. Farkas, J. B. Foresman and D. J. Fox, Gaussian Inc., Wallingford CT, 2016.

37 W. Humphrey, A. Dalke and K. Schulten, VMD: visual molecular dynamics, *J. Mol. Graph.*, 1996, **14**, 33-8, 27-8.
